# Trinucleotide cap analogs with triphosphate chain modifications: synthesis, properties, and evaluation as mRNA capping reagents

**DOI:** 10.1093/nar/gkae763

**Published:** 2024-09-09

**Authors:** Marcin Warminski, Anais Depaix, Kamil Ziemkiewicz, Tomasz Spiewla, Joanna Zuberek, Karolina Drazkowska, Hanna Kedzierska, Agnieszka Popielec, Marek R Baranowski, Marta Sklucka, Marcelina Bednarczyk, Miroslaw Smietanski, Karol Wolosewicz, Bartosz Majewski, Remigiusz A Serwa, Dominika Nowis, Jakub Golab, Joanna Kowalska, Jacek Jemielity

**Affiliations:** Division of Biophysics, Institute of Experimental Physics, Faculty of Physics, University of Warsaw, Pasteura 5, 02-093 Warsaw, Poland; Centre of New Technologies, University of Warsaw, Banacha 2C, 02-097 Warsaw, Poland; Centre of New Technologies, University of Warsaw, Banacha 2C, 02-097 Warsaw, Poland; Division of Biophysics, Institute of Experimental Physics, Faculty of Physics, University of Warsaw, Pasteura 5, 02-093 Warsaw, Poland; Explorna Therapeutics sp. z o.o, Zwirki i Wigury 93/2157, 02-089 Warsaw, Poland; Division of Biophysics, Institute of Experimental Physics, Faculty of Physics, University of Warsaw, Pasteura 5, 02-093 Warsaw, Poland; Centre of New Technologies, University of Warsaw, Banacha 2C, 02-097 Warsaw, Poland; Explorna Therapeutics sp. z o.o, Zwirki i Wigury 93/2157, 02-089 Warsaw, Poland; Explorna Therapeutics sp. z o.o, Zwirki i Wigury 93/2157, 02-089 Warsaw, Poland; Explorna Therapeutics sp. z o.o, Zwirki i Wigury 93/2157, 02-089 Warsaw, Poland; Explorna Therapeutics sp. z o.o, Zwirki i Wigury 93/2157, 02-089 Warsaw, Poland; Explorna Therapeutics sp. z o.o, Zwirki i Wigury 93/2157, 02-089 Warsaw, Poland; Explorna Therapeutics sp. z o.o, Zwirki i Wigury 93/2157, 02-089 Warsaw, Poland; Explorna Therapeutics sp. z o.o, Zwirki i Wigury 93/2157, 02-089 Warsaw, Poland; Explorna Therapeutics sp. z o.o, Zwirki i Wigury 93/2157, 02-089 Warsaw, Poland; Proteomics Core Facility, IMol Polish Academy of Sciences, 02-247 Warsaw, Poland; Explorna Therapeutics sp. z o.o, Zwirki i Wigury 93/2157, 02-089 Warsaw, Poland; Laboratory of Experimental Medicine, Faculty of Medicine, Medical University of Warsaw, Nielubowicza 5, 02-097 Warsaw, Poland; Explorna Therapeutics sp. z o.o, Zwirki i Wigury 93/2157, 02-089 Warsaw, Poland; Department of Immunology, Medical University of Warsaw, Nielubowicza 5, 02-097 Warsaw, Poland; Division of Biophysics, Institute of Experimental Physics, Faculty of Physics, University of Warsaw, Pasteura 5, 02-093 Warsaw, Poland; Explorna Therapeutics sp. z o.o, Zwirki i Wigury 93/2157, 02-089 Warsaw, Poland; Centre of New Technologies, University of Warsaw, Banacha 2C, 02-097 Warsaw, Poland; Explorna Therapeutics sp. z o.o, Zwirki i Wigury 93/2157, 02-089 Warsaw, Poland

## Abstract

The recent COVID-19 pandemics have demonstrated the great therapeutic potential of *in vitro* transcribed (IVT) mRNAs, but improvements in their biochemical properties, such as cellular stability, reactogenicity and translational activity, are critical for further practical applications in gene replacement therapy and anticancer immunotherapy. One of the strategies to overcome these limitations is the chemical modification of a unique mRNA 5′-end structure, the 5′-cap, which is responsible for regulating translation at multiple levels. This could be achieved by priming the *in vitro* transcription reaction with synthetic cap analogs. In this study, we combined a highly efficient trinucleotide IVT capping technology with several modifications of the 5′ cap triphosphate bridge to synthesize a series of 16 new cap analogs. We also combined these modifications with epigenetic marks (2′-O-methylation and m^6^A_m_) characteristic of mRNA 5′-ends in higher eukaryotes, which was not possible with dinucleotide caps. All analogs were compared for their effect on the interactions with eIF4E protein, IVT priming, susceptibility to decapping, and mRNA translation efficiency in model cell lines. The most promising α-phosphorothiolate modification was also evaluated in an *in vivo* mouse model. Unexpected differences between some of the analogs were analyzed using a protein cell extract pull-down assay.

## Graphical Abstract

**Graphical Abstract:**
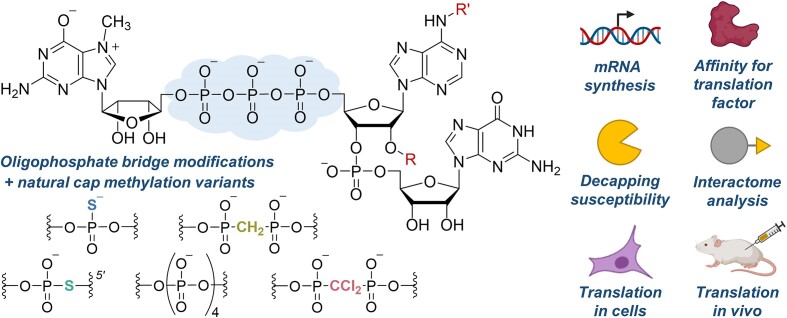

## Introduction

Cellular mRNAs encode information about the sequence of proteins and serve as molecular templates during translation. This phenomenon can be harnessed for therapeutic purposes by delivering into the patient's body an exogenous mRNA encoding an immunizing or therapeutic protein. For this purpose, mRNAs are typically obtained by *in vitro* transcription reaction (1–3). *In vitro* transcribed (IVT) mRNAs are thus an emerging class of therapeutics with increasing number of existing and potential applications (4–8). To increase stability and translational potential, and avoid unwanted reactogenicity, IVT mRNAs synthesized for therapeutic purposes need to be highly purified (9,10) and are usually equipped with stabilizing elements along with additional chemical modifications (11–16). Chemically modified mRNA has tremendous potential in the context of therapeutic applications, of which the SARS-CoV-2 vaccines were a spectacular example. The potential applications of mRNA are much broader than antiviral vaccines and include anti-cancer applications, treatment of rare genetic diseases, cell therapies, regenerative medicine, therapeutic approaches involving precise genome editing and others. However, in order for mRNA to be successfully applied in these more challenging contexts, it is still necessary to improve mRNA technology and to develop new molecular tools to better understand mRNA metabolism.

One of the key structural elements necessary for sufficient mRNA stability and efficient translation is the 5′ cap—a structure present at the 5′ end of all eukaryotic mRNAs consisting of 7-methylguanosine linked to the first transcribed ribonucleotide by a 5′,5′-triphosphate chain. During translation initiation, the cap is bound by the eukaryotic translation initiation factor 4E to facilitate ribosome recruitment. Cap also plays a role as a stabilizing element for the mRNA 5′ end, and needs to be removed by a specialized decapping enzyme, Dcp1/Dcp2 complex, before 5′-to-3′ mRNA degradation by exonucleases is triggered. The presence of the 5′ cap, along with additional epitranscriptomic methylations within the first transcribed nucleotides (17–24), is also one of the key structural features required for discrimination between self and non-self RNAs by the innate immune system (25,26). In the cells, the cap structure is introduced into mRNA by a sequence of three enzymatic reactions, whereas IVT mRNA is usually capped co-transcriptionally by adding a properly designed di- or tri-nucleotide capping reagent. The latter strategy allows the preparation of therapeutic mRNAs equipped with cap structures either identical to the natural structure or their chemically modified analogs (27,28).

An important group of chemically modified caps are those modified in the 5′,5′-triphosphate chain. Miscellaneous dinucleotide capping reagents carrying phosphate-chain modifications have been explored to date as mRNA capping reagents (29). These modifications include bridging phosphate modifications such as elongation of the oligophosphate chain, a substitution at one of the bridging positions of the oligophosphate chain (e.g. O-to-CH_2_ (30,31), O-to-CF_2_ (31), O-to CCl_2_ (31), O-to-NH (32) or 5′-O-to-S (33)), a substitution at one or more of the non-bridging positions of the oligophosphate chain (single O-to-S and O-to-BH_3_ substitutions (34–37), multiple O-to-S (38)) or more bulky modifications such as phosphotriazole cap analogs (39–41). These studies have revealed that phosphate modifications may substantially affect the biochemical properties of the 5′ cap such as affinity for eIF4E, incorporation efficiency by RNA polymerase, and susceptibility to decapping, which in turn affect the properties of mRNA, including stability and translational efficiency. In general, increased affinity for eIF4E and decreased susceptibility to decapping by Dcp1/Dcp2 complex are desirable features in mRNA cap design, although some studies suggest that other, so far unidentified factors, may also play a significant role in determining overall translational properties of mRNAs carrying modified 5′ caps. Despite extensive work done so far, drawing more general conclusions on the influence of phosphate modifications within the cap is impaired by the fact that these modifications have been studied independently from each other, and manufacturing, purification and evaluation protocols in various model systems have been evolving over the years. Coincidently, most recent studies strongly suggest that these factors may significantly affect the outcome of translational studies on IVT mRNA.

Recently, trinucleotide mRNA capping reagents have been developed (3,12,41–44), which enabled both improving the incorporation efficiencies of native 5′ cap structures by T7 RNA polymerase (capping efficiencies) and incorporation of natural, epigenetic marks: 2′-*O*-methylation of the first and the second transcribed nucleotides and *N*6-methylation of 5′-terminal adenosine (which was not possible with the dinucleotide technology without further enzymatic processing of the samples).

**Figure 1.**
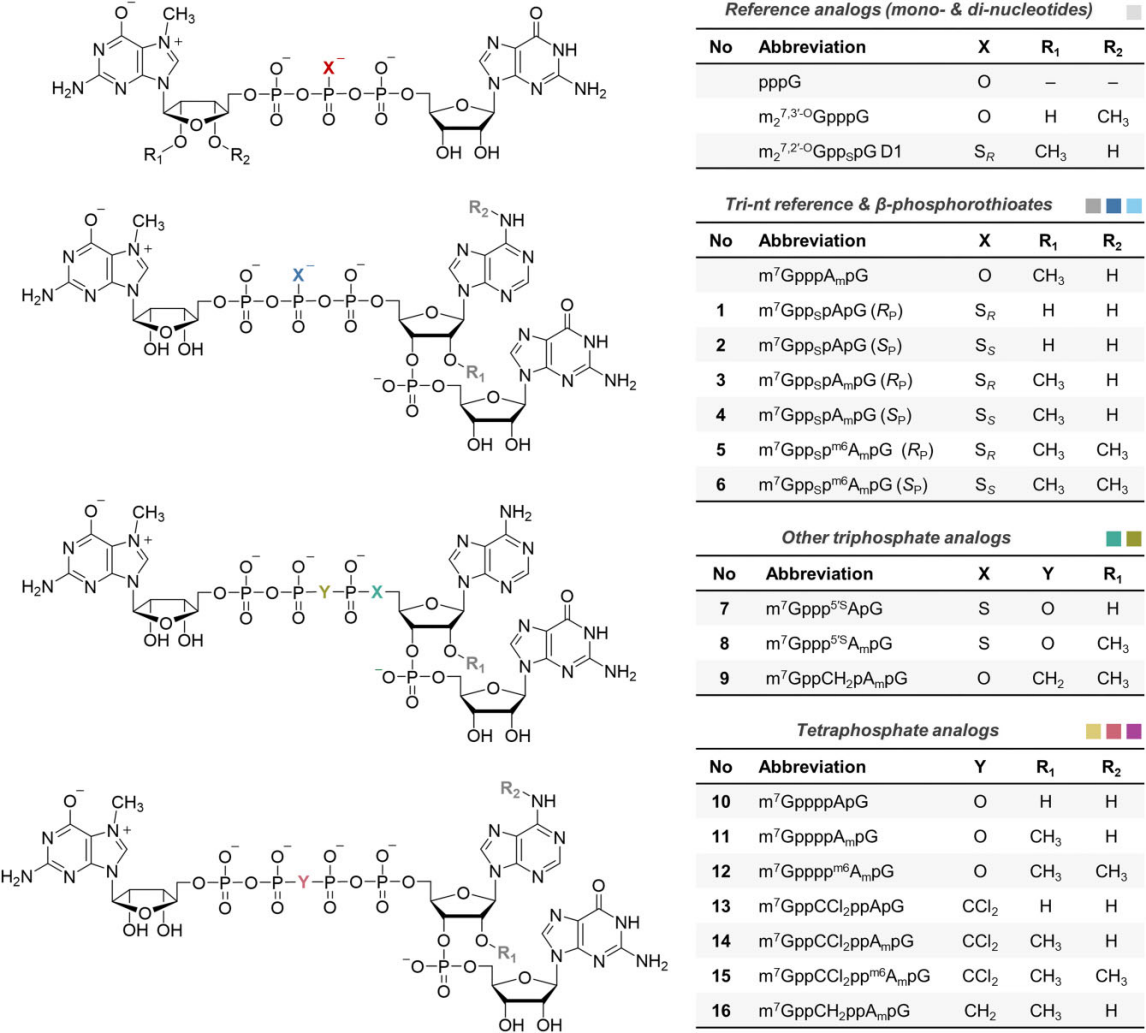
Structures of cap analogs synthesized in this work. The analogs were color-coded through-out the whole manuscript as follows: light-grey—mono- and dinucleotide reference analogs, grey—trinucleotide reference analogs, dark-blue—β-phosphorothioate analogs D1 (*R*_P_) diastereomers, light-blue—β-phosphorothioate analogs D2 (*S*_P_) diastereomers, green—5′-phosphorothiolate (PSL) analogs, olive-green—α,β-methylenebisphosphonate analogs, yellow—tetraphosphate analogs, pink—β,γ-dichloromethylenebisphosphonate analogs, purple—β,γ-methylenebisphosphonate analog.

Here, we have combined the trinucleotide technology with various modifications of the 5′,5′-triphosphate chain to back-to-back compare these modifications as mRNA capping reagents and evaluate the effects of combining them with epigenetic marks present at the 5′ end of mRNA. To that end, we have synthesized a series of 16 novel trinucleotide cap analogs (Figure 1, Supplementary Table S1) and evaluated their properties, including affinity for eIF4E, incorporation into RNA by T7 polymerase, susceptibility to decapping by recombinant hDcp2 and translational properties of mRNAs carrying these modified caps in cultured cells. To get insight into the possible mechanisms underlying unexpected differences observed for some of the caps, we have performed pulldown assays combined with proteomic analyses. Finally, the mRNA containing one of the most promising α-phosphorothiolate modification was formulated into lipid nanoparticles and evaluated in an *in vivo* mouse model.

## Materials and methods

### Synthesis of trinucleotide cap analogs

#### General information

Solvents, chemical reagents, and starting materials, including guanosine 5′-*O*-diphosphate (GDP), guanosine 5′-*O*-triphosphate (GTP), adenosine and 2′-*O*-Me phosphoramidites were purchased from commercial sources. The m^6^A_m_ phosphoramidite was synthesized as described earlier (18,45). The following modified mononucleotides were synthesized according to the previously published procedures: *N*7-methylguanosine 5′-*O*-monophosphate (m^7^GMP) (35), *N*7-methylguanosine 5′-*O*-diphosphate (m^7^GDP) (35), *N*7-methylguanosine 5′-*O*-diphosphate *P*-imidazolide (m^7^GDP-Im) (35), *N*7-methylguanosine 5′-*O*-(2-thiodiphosphate) (m^7^GDP-β-S) (35), guanosine 5′-*O*-(2,3-methylenetriphosphate) (GppCH_2_p) (46) and guanosine 5′-*O*-(2,3-dichloromethylenetriphosphate) (GppCCl_2_p) (31).

All the synthesized compounds were isolated from reaction mixtures by ion-exchange chromatography on DEAE Sephadex A-25 (HCO_3_^–^ form). The column was washed with water, loaded with the reaction mixture, and then washed thoroughly with water until the eluate did not form a visible precipitate with 1% AgNO_3_ solution. Nucleotides were eluted using a linear gradient of triethylammonium bicarbonate (TEAB) in deionized water and the fractions were collected based on the absorption at 260 nm. After evaporation of the eluate under reduced pressure with repeated additions of 96% ethanol and then acetonitrile, the nucleotides were isolated as triethylammonium salts. The trinucleotide cap analogs **1**–**16** were additionally purified using semi-preparative RP-HPLC with Gemini® NX-C18 (Phenomenex) RP-HPLC column (150 × 10 mm, 5 μm, 110 Å, flow rate 5.0 ml/min) with UV detection at 254 nm and isolated from the eluate by repeated freeze-drying.

The synthesis yields were calculated based on the optical density (mOD = absorbance of the solution × volume in ml) of combined fractions measured in 0.1 M phosphate buffer pH 6 (for 7-methylguanosine mononucleotides) or pH 7 (for all the other compounds) at 260 nm. The following extinction coefficient values were assumed in the calculations: ϵ = 32.0 l/mmol/cm for all trinucleotide cap analogs (compounds **1–19**); ϵ = 27.1 l/mmol/cm for all dinucleotides containing A and G nucleobases (compounds **20–30**); ϵ = 11.4 l/mmol/cm for all 7-methylguanosine mononucleotides. The structure and homogeneity of each compound was confirmed by re-chromatography by RP-HPLC and high resolution mass spectrometry with electrospray ionization (HRMS-ESI) in negative ions mode. Cap analogs that were synthesized in sufficient amounts were additionally characterized by Nuclear Magnetic Resonance spectroscopy (^1^H, ^31^P, COSY, ^13^C HSQC and ^31^P HSQC NMR). Analytical HPLC was performed using a Gemini® NX-C18 HPLC column (4.6 × 150 mm, 3 μm, 110 Å, flow rate 1.0 ml/min) with a 0–50% linear gradient of methanol in 0.05 M ammonium acetate buffer (pH 5.9) in 30 min (conditions A) or in 15 min (conditions B) and UV detection at 254 nm. Mass spectra were recorded with LTQ Orbitrap Velos (Thermo Scientific, high resolution). NMR spectra were recorded at 25°C with a Bruker Avance III 500 MHz spectrometer equipped with 5 mm PABBO BB/19F-1H/D Z-GRD probe.

#### Synthesis of 7-methylguanosine triphosphate analogs (m^7^GppYp) – general procedure

The synthesis of m^7^GppYp (Y = O, CCl_2_ or CH_2_) was described earlier (31), but we decided to take a different approach to facilitate ion-exchange purification. Briefly, GppYp (Y = O, CCl_2_ or CH_2_) was dissolved in water (0.1 M) and the solution was acidified to pH = 4 with 80% acetic acid. Then, dimethyl sulfate (10 equivalents) was added in 10 portions over a period of one hour and the pH was kept at ca. 3 by adding 1 M NaOH (in 100 uL portions). The reaction was quenched after 3 h by dilution with water (10 volumes) and the mixture was washed with ethyl acetate. The residual ethyl acetate was removed from the aqueous phase under reduced pressure and the product was isolated by ion-exchange chromatography on DEAE Sephadex (gradient elution 0–0.7 M TEAB) to afford—after evaporation—triethylammonium salt of m^7^GppYp. RP-HPLC profiles and mass spectra of all three products matched those reported earlier (31). Yields: m^7^Gppp—12 060 mOD (1.06 mmol, 66%), m^7^GppCH_2_p—3090 mOD (0.271 mmol, 60%), m^7^GppCCl_2_p—3710 mOD (0.325 mmol, 87%).

#### Synthesis of 5′-phosphorylated dinucleotides 20–22 (pNpG)—general procedure

Synthesis of dinucleotides was performed using ÄKTA Oligopilot plus 10 synthesizer (GE Healthcare) on a 5′-*O*-DMT-2′-*O*-TBDMS-rG^iBu^ 3′-lcaa PrimerSupport 5G (308 μmol/g) solid support (GE Healthcare). In the coupling step, 2.0 equivalents of 5′-*O*-DMT-2′-*O*-TBDMS/2′-*O*-Me-3′-*O*-phosphoramidite (rA^Ac^, rA_m_^Pac^, ^m6^A^Ac^ or ^m6^A_m_^Pac^) (12,45) or biscyanoethyl phosphoramidite and 0.30 M 5-(benzylthio)-1-*H*-tetrazole in acetonitrile were recirculated through the column for 15 min. A solution of 3% (v/v) dichloroacetic acid in toluene was used as a detritylation reagent, 0.05 M iodine in pyridine/water (9:1) for oxidation, 20% (v/v) *N*-methylimidazole in acetonitrile as Cap A and a mixture of 40% (v/v) acetic anhydride and 40% (v/v) pyridine in acetonitrile as Cap B. After the last cycle of synthesis, dinucleotides, still attached to the solid support, were treated with 20% (v/v) diethylamine in acetonitrile to remove 2-cyanoethyl protecting groups. Finally, the solid support was washed with acetonitrile and dried with argon. The product was cleaved from the solid support and deprotected with AMA (methylamine/ammonium hydroxide 1:1 (v/v), 55°C, 1 h), evaporated to dryness, and redissolved in DMSO (200 μl). The TBDMS groups were removed using triethylammonium trihydrofluoride (TEA·3HF; 250 μl, 65°C, 3 h), and then the mixture was cooled down and diluted with 0.25 M NaHCO_3_ in water (20 ml). The product was isolated by ion-exchange chromatography on DEAE Sephadex (gradient elution 0–0.9 M TEAB) to afford after evaporation triethylammonium salt of dinucleotide **20–22** (pNpG). The synthesis scales, yields, HPLC and HRMS data for particular dinucleotides are summarized in Supplementary Table S3.

#### Synthesis of dinucleotide 5′-phosphorothiolates 26–27 (p^5′S^NpG)—general procedure

Synthesis of 5′-OH-NpG dinucleotides was performed using ÄKTA Oligopilot plus 10 synthesizer (GE Healthcare) on a 5′-*O*-DMT-2′-*O*-TBDMS-rG^iBu^ 3′-lcaa PrimerSupport 5G (308 μmol/g) solid support (GE Healthcare). In the coupling step, 2.5 equivalents of adenosine 3′-*O*-phosphoramidite (5′-*O*-DMT-2′-*O*-PivOM-rA^Pac^ or 5′-*O*-DMT-rA_m_^Pac^) and 0.30 M 5-(benzylthio)-1-*H*-tetrazole (BTT) in acetonitrile were recirculated through the column for 15 min. A solution of 3% (v/v) dichloroacetic acid in toluene was used as a detritylation reagent, 0.05 M iodine in pyridine for oxidation, 20% (v/v) *N*-methylimidazole in acetonitrile as Cap A and a mixture of 40% (v/v) acetic anhydride and 40% (v/v) pyridine in acetonitrile as Cap B. After the last cycle of synthesis, the support was treated with 20% (v/v) diethylamine in acetonitrile to remove 2-cyanoethyl protecting groups, washed with acetonitrile and dried with argon. Dinucleotide, still on a solid support, was then converted into 5′-iodo derivative by pushing back and forth (using two syringes attached to the column) a solution of triphenoxymethylphosphonium iodide (1.5 g, 3.32 mmol) in DMF (5 ml) for 15 min. The resin was then washed with DMF (10 ml) and acetonitrile (10 ml), dried and transferred to a flask containing a cold solution of triethylammonium thiophosphate (ca. 0.16 M) and triethylamine (0.64 M) in DMF (1 ml). The slurry was stirred at 4°C overnight, filtered, and washed with acetonitrile. The product was cleaved and deprotected using AMA (40% methylamine/33% ammonium hydroxide 1:1 (v/v); 55°C, 1 h) and isolated by ion-exchange chromatography on DEAE Sephadex (gradient elution 0–0.9 M TEAB) to afford after evaporation triethylammonium salt of dinucleotide **26–27** (p^5′S^NpG). The synthesis scales, yields, HPLC and HRMS data for particular dinucleotides are summarized in Supplementary Table S3.

#### Synthesis of dinucleotide 5′-methylenebisphosphonate 28 (pCH_2_pA_m_pG)—general procedure

Synthesis of 5′-OH-A_m_pG dinucleotide was performed using ÄKTA Oligopilot plus 10 synthesizer (GE Healthcare) on a 5′-*O*-DMT-2′-*O*-TBDMS-rG^iBu^ 3′-lcaa PrimerSupport 5G (308 μmol/g) solid support (GE Healthcare). In the coupling step, 5.0 equivalents of rA^Pac^-5′-*O*-DMT-2′-*O*-Me-3′-*O*-phosphoramidite and 0.30 M 5-(benzylthio)-1-*H*-tetrazole in acetonitrile were recirculated through the column for 15 min. A solution of 3% (v/v) dichloroacetic acid in toluene was used as a detritylation reagent, 0.05 M iodine in pyridine for oxidation, 20% (v/v) *N*-methylimidazole in acetonitrile as Cap A and a mixture of 40% (v/v) acetic anhydride and 40% (v/v) pyridine in acetonitrile as Cap B. After the synthesis, the support was washed with acetonitrile and dried with argon. A solution of methylenebis(phosphonic dichloride) (500 mg, 2 mmol) in trimethyl phosphate (5 ml) cooled to −18°C was applied to the column and left for 7 h at 2°C. Then the solution was removed and the support was washed with trimethyl phosphate (5 ml) and acetonitrile (10 ml) and dried with argon. The column was washed with 5 ml of 0.9 M TEAB and the resin was incubated with fresh portion of TEAB at 2°C overnight. The product was cleaved from the solid support and deprotected with AMA (methylamine/ammonium hydroxide 1:1 (v/v); 55°C, 1 h), evaporated to dryness and redissolved in DMSO (200 μl). The TBDMS groups were removed using triethylammonium trihydrofluoride (TEA·3HF; 250 μl, 65°C, 3 h), then the mixture was cooled down, diluted with water and pH was adjusted to 1 using hydrogen chloride, and left for 7 days at room temperature to hydrolyze fluorobisphosphonate. The product was isolated by ion-exchange chromatography on DEAE Sephadex (gradient elution 0–0.9 M TEAB) to afford after evaporation triethylammonium salt of dinucleotide **28** (pCH_2_pA_m_pG). The synthesis scale, yield, HPLC and HRMS data are summarized in Supplementary Table S3.

#### Activation of pNpG into P-imidazolides 23–25 (Im-pNpG)—general procedure

Dinucleotide 5′-phosphate **20–22** (pNpG) was dissolved in DMF (to obtain a 0.05 M solution) followed by addition of imidazole (16 equivalents), 2,2′-dithiodipiridine (6 equivalents), triethylamine (3 equivalents) and triphenylphosphine (6 equivalents). The mixture was stirred at room temperature for 48 h. The product was precipitated by the addition of a 0.05 M solution of sodium perchlorate (10 equivalents) in acetonitrile (10 times the volume of DMF). The precipitate was centrifuged at 4°C, washed with cold acetonitrile 3 times, and dried under reduced pressure to give a sodium salt of dinucleotide *P*-imidazolide **23**–**25** (Im-pNpG). The compounds were immediately used for further reactions.

#### Synthesis of β-phosphorothioate trinucleotide cap analogs 1–6 (m^7^Gpp_S_pNpG)—general procedure

7-Methylguanosine β-thiodiphosphate (m^7^GDP-β-S; obtained as described earlier and stored in TEAB at −20°C) (35) was evaporated to an oil and redissolved in DMF (to obtain a 0.05 M solution). Then ZnCl_2_ (8 equivalents) and Im-pNpG (**23–25**, 0.5 equivalent) were added and the solution was stirred at room temperature for 2 h. The reaction was quenched by the addition of a solution of Na_2_EDTA (20 mg/ml; 8 equivalents) and NaHCO_3_ (10 mg/ml) in water and the product was isolated by ion-exchange chromatography on DEAE Sephadex (gradient elution 0–1.2 M TEAB) to afford, after evaporation, triethylammonium salt of m^7^Gpp_S_pNpG. The diastereomers were separated by RP-HPLC (C18) using a linear gradient of acetonitrile in aqueous CH_3_COONH_4_ buffer pH 5.9 to give—after repeated freeze-drying from water—ammonium salts of pure diastereomers of m^7^Gpp_S_pNpG (**1–6**). The synthesis scales, yields, HPLC and HRMS data for particular cap analogs are summarized in Supplementary Table S4.

#### Synthesis of phosphorothiolate trinucleotide cap analogs 7 and 8 (m^7^Gppp^5′S^NpG)—general procedure

Dinucleotide 5′-phosphorothiolate **26–27** (p^5′S^NpG), 7-methylguanosine-5′-diphosphate *P*^2^-imidazolide m^7^GDP-Im (2 equivalents) and ZnCl_2_ (20 equivalents) were dissolved in DMSO (to obtain 0.05 M of p^5′S^NpG) and the solution was stirred at room temperature for 3 days. The reaction was quenched by the addition of a solution of Na_2_EDTA (20 mg/ml; 20 equivalents) and NaHCO_3_ (10 mg/ml) in water and the product was isolated by ion-exchange chromatography on DEAE Sephadex (gradient elution 0–1.2 M TEAB) to afford—after evaporation—triethylammonium salt of m^7^Gppp^5′S^NpG. Additional purification by RP-HPLC (C18) using a linear gradient of acetonitrile in aqueous CH_3_COONH_4_ buffer pH 5.9 provided—after repeated freeze-drying from water—ammonium salts of m^7^Gppp^5′S^NpG (**7–8**). The synthesis scales, yields, HPLC and HRMS data for particular cap analogs are summarized in Supplementary Table S4.

#### Synthesis of methylenebisphosphonate trinucleotide cap analog 9 (m^7^GppCH_2_pA_m_pG)—general procedure

Dinucleotide 5′-methylenebisphosphonate **28** (pCH_2_pA_m_pG), 7-methylguanosine-5′-monophosphate *P*-imidazolide m^7^GMP-Im (5 equivalents) and ZnCl_2_ (20 equivalents) were dissolved in DMSO (to obtain 0.05 M of pCH_2_pA_m_pG) and the solution was stirred at room temperature for 24 h. The reaction was quenched by the addition of a solution of Na_2_EDTA (20 mg/ml; 20 equivalents) and NaHCO_3_ (10 mg/ml) in water and the product was isolated by ion-exchange chromatography on DEAE Sephadex (gradient elution 0–1.2 M TEAB) to afford—after evaporation—triethylammonium salt of m^7^GppCH_2_pA_m_pG. Additional purification by RP-HPLC (C18) using a linear gradient of acetonitrile in aqueous CH_3_COONH_4_ buffer pH 5.9 provided—after repeated freeze-drying from water – ammonium salts of m^7^GppCH_2_pA_m_pG (**9**). The synthesis scales, yields, HPLC and HRMS data for particular cap analogs are summarized in Supplementary Table S4.

#### Synthesis of tetraphosphate cap analogs 10–16 (m^7^GppYppNpG)—general procedure

The appropriate 7-methylguanosine triphosphate analog (m^7^GppYp; Y = O, CCl_2_ or CH_2_, 1.5 equivalents) and ZnCl_2_ (8.0 equivalents) were dissolved in DMF (to obtain a 0.075 M solution of m^7^GppYp). Then the dinucleotide Im-pNpG (**23–25**, 1.0 equivalent) was added and the solution was stirred at room temperature for 2–9 h. The reaction was quenched by the addition of a solution of Na_2_EDTA (20 mg/ml; 9 equivalents) and NaHCO_3_ (10 mg/ml) in water and the product was isolated by ion-exchange chromatography on DEAE Sephadex (gradient elution 0–1.2 M TEAB) to afford after evaporation triethylammonium salt of m^7^GppYppNpG. The diastereomers were separated by RP-HPLC (C18) using a linear gradient of acetonitrile in aqueous CH_3_COONH_4_ buffer pH 5.9 to give—after repeated freeze-drying from water—ammonium salts of pure m^7^GppYppNpG (**10–16**). The synthesis scales, yields, HPLC and HRMS data for particular cap analogs are summarized in Supplementary Table S4.

#### Synthesis of functionalized trinucleotide cap analogs 17–19 (m^7^GppYp_n_A_m_pG-L13_N_)—general procedure

The triethylammonium salt of **21 (**pA_m_pG) was dissolved in DMSO (to 0.05M solution) and 1,1′-carbonyldiimidazole (CDI, 10 equivalents) was added. The mixture was stirred at room temperature for 2 h and then water (16 equivalents) was added to hydrolyze an excess of CDI. After 20 min, 4,7,10-trioxatridecane-1,13-diamine (3 equivalents) and 1,8-Diazabicyclo[5.4.0]undec-7-ene (DBU, 1 equivalent) were added and the mixture was stirred at room temperature until all the dinucleotide 2′,3′-*O*,*O*-carbonate was consumed (as evidenced by RP-HPLC). The crude product **28**—a regioisomeric mixture of 2′-*O*/3′-*O*-carbamoyl dinucleotide *P*-imidazolide (Im-pA_m_pG-L13_N_)—was isolated as a sodium salt by the addition of a NaClO_4_ (6 equivalents) solution in cold acetonitrile (10 times volume of DMSO). The white precipitate was centrifuged, washed with cold acetonitrile three times and dried under reduced pressure. The crude **28** (Im-pA_m_pG-L13_N_), m^7^GppYp (2 equivalents) and ZnCl_2_ (16 equivalents) were suspended in DMSO (to 0.025 M concentration of **28**) and the mixture was stirred at room temperature for a week. The reaction was quenched by the addition of a solution of Na_2_EDTA (20 mg/ml; 20 equivalents) and NaHCO_3_ (10 mg/ml) in water and the product was isolated by ion-exchange chromatography on DEAE Sephadex (gradient elution 0–1.2 M TEAB) to afford—after evaporation—triethylammonium salt of m^7^GppYp_n_A_m_pG-L13_N_ (**17–19**). The products were contaminated with m^7^G mononucleotide, but since it lacks primary amine groups, it does not interfere with subsequent immobilization on the Sepharose. The synthesis scales, yields, HPLC and HRMS data for particular cap analogs are summarized in Supplementary Table S4.

#### Synthesis of affinity resins AR-1–AR-3

The affinity resins were synthesized based on the previously published protocol (47). Briefly, a suspension of Sepharose™ CL-4B (3 ml of settled resin) in water (6 ml total volume) was cooled down in an ice-water bath and a solution of BrCN (386 mg) in acetonitrile (386 μl) was added. The mixture was stirred in an ice-water bath and the pH was kept at the level of 11 by addition of 1 M NaOH (ca. 200 μl every 1–5 min). After 2 h the slurry was filtered on a Buchner funnel (not letting the resin get dry) and washed with cold water (200 ml), and then with 0.1 M carbonate buffer pH 9 (200 ml). The resin was transferred into a vial and suspended in carbonate buffer pH 9 (3 ml). A solution of trinucleotide cap analog **17**, **18** or **19** (1.0 μmol) in water (100 μl) was added, and the mixture was stirred gently at 4°C overnight. The slurry was filtered, washed with water (150 ml) and 20% ethanol (150 ml). The flow-through was analyzed by RP-HPLC to estimate the resin loading (usually 0.8–1.0 μmol/ml of the settled resin). The resins were stored in 20% ethanol (3 ml—total volume of ca. 6 ml) at 4°C.

### Expression and purification of human eIF4E1a

The human eIF4E1a protein was expressed without affinity tag in *E. coli* Rosseta 2 (DE3)pLysS strain (Novagen) under conditions leading to accumulation of the produced protein in inclusion bodies (48). The protein was extracted from inclusion bodies with buffer containing 6 M guanidine hydrochloride, refolded by one-step dialysis against 50 mM HEPES/KOH (pH 7.2), 100 mM KCl, 0.5 mM EDTA, 2 mM DTT, and purified by ion exchange chromatography on HiTrap SP HP 5 ml column (GE Healthcare) using salts gradient. Finally, protein was filtered using Ultrafree MC-HV Centrifugal PVDF filters with 0.45 μm membrane pore (Millipore) to remove protein aggregates and its concentration was determined spectrophotometrically assuming the theoretical extinction coefficient ϵ_280nm_ = 52 940 L mol^−1^cm^−1^—calculated from amino acid composition using algorithm on ExPASy Server.

### Fluorescence binding assay

Fluorescence titration measurements were carried out on a LS-55 spectrofluorometer (Perkin Elmer Co., Norwalk, CT, USA) in a quartz semi-micro cuvette (Hellma, Germany) with optical lengths 4 mm and 10 mm for absorption and emission, respectively, which was thermostated at 20.0 ± 0.2 °C. The titrations were performed in 50 mM Hepes/KOH pH 7.2, 100 mM KCl, 0.5 mM EDTA and 1 mM DTT by adding 1 μl aliquots of increasing concentrations of the cap solutions to 1.4 ml of 0.1 μM eIF4E1a solution. The eIF4E1a fluorescence was excited at 280 nm and the fluorescence intensity was monitored at a single wavelength 340 nm. The measured fluorescence intensities were corrected for dilution (<4%) and for the *inner filter* effect. Association equilibrium constants (*K*_as_) were obtained by fitting the theoretical curve for fluorescence intensity (F) upon total concentration of cap analogs ([L]) to the titration data according to the equation:


\begin{equation*}{\mathrm{F}} = {\mathrm{F}}\left( 0 \right) - \left[ {{\mathrm{cx}}} \right] \cdot \left( {{\mathrm{\Delta \Phi }} + {{{\mathrm{\Phi }}}_{{\mathrm{lig}} - {\mathrm{free}}}}} \right) + \left[ {\mathrm{L}} \right] \cdot {{{\mathrm{\Phi }}}_{{\mathrm{lig}} - {\mathrm{free}}}}\end{equation*}

with the concentration of the cap analogs–eIF4E1a complex [cx] given by:


\begin{eqnarray*} [{cx}] &=& \frac{{[L] + [{{{P}_{act}}}]}}{2}\nonumber\\ && +\, \frac{{1 - \sqrt {{{{( {{{K}_{as}}( {[L] - [{{{P}_{act}}}]}) + 1})}}^2} + 4{{K}_{as}} \cdot [{{{P}_{act}}}]} }}{{2{{K}_{as}}}}\end{eqnarray*}

where Δϕ stands for the difference between the fluorescence efficiencies of the apo- and cap analog bound proteins; ϕ_lig-free_ is the fluorescence efficiency of the free cap analog; F(0) is the initial fluorescence, and P_act_ is the total concentration of the active protein (49). The final *K*_AS_ values were calculated as a weighted average from three independent titrations and represented as the *K*_D_ values. Numerical least-squares nonlinear regression analysis was performed using ORIGIN 2021 from Microcal Software Inc., USA.

### T7 RNA polymerase expression and purification

*Escherichia* phage T7 RNA polymerase (Gene ID: 1 261 050) sequence was obtained as a plasmid vector (pT7-911Q) from Marcin Nowotny (Laboratory of Protein Structure, International Institute of Molecular and Cell Biology in Warsaw, Poland). T7 RNA Polymerase protein (wt, ≈99 kDa) with Histidine tag at 5′-end was overexpressed in BL21 (DE3) RIL E.coli (Invitrogene) procaryotic expression system. 6xHis-T7 RNA polymerase protein was induced with 0.6 mM IPTG (Isopropyl-*β*-d-thiogalactoside) solution in bacterial culture optical density ≈ 0.75 and further cultured for 16 h at 18°C. Harvested cells were lysed in buffer containing: 50 mM Tris pH 7.5, 300 mM NaCl, 20 mM imidazole, 1 mM DTT (dithiothreitol), 10% glycerol, 0.1 mg/ml lysozyme and mixture of protease inhibitors (aprotinin, leupeptin, pepstatin, PMSF). The lysate was sonicated (20 min, 50% power, 15 s on/off) and centrifuged. The supernatant was loaded on 2 × 5 ml HisTrap FFTM column (Cytiva) equilibrated with buffer: 50 mM Tris pH 7.5, 250 mM NaCl, 20 mM imidazole, 1 mM DTT, 10% glycerol. 6× His-T7 RNA polymerase protein was washed with buffer containing 1 M NaCl and eluted with buffer: 50 mM Tris pH 7.5, 250 mM NaCl, 300 mM imidazole, 1 mM DTT, 10% glycerol. The protein was further purified on the heparin column to remove nucleic acid impurities. T7 RNA polymerase fractions were diluted in buffer: 50 mM Tris pH 7.5, 1 mM DTT to reach a salt concentration of 100 mM in the protein sample and then loaded on 5 ml HiTrap HeparinTM column (Cytiva) equilibrated with buffer: 50 mM Tris pH 7.5, 100 mM NaCl, 1 mM DTT, 5% glycerol. The elution was carried out with a high salt concentration buffer containing: 50 mM Tris pH = 7.5, 1 M NaCl, 1 mM DTT, 5% glycerol. Finally, a gel filtration was performed on a Superdex 200 pg HiLoad 26/600 column (Cytiva). The samples containing T7 RNA polymerase protein were concentrated to 3 mg/ml, flash *frozen and st*ored at −80°C in a buffer containing 10 mM Tris pH 7.5, 300 mM NaCl, 1 mM DTT, 10% glycerol.

### Preparation of DNA plasmid vectors

**Step 1**—sticky-end cloning of Luciferase gene: Luciferase gene (FLuc) from Firefly (Lampyridae), containing restriction sites for AdeI and BamHI endonucleases, was purchased from Invitrogen and cloned into pJet1.2 (Thermo) plasmid vector using the sticky-end cloning method.

#### Insert formation

The ordered FLuc plasmid (2 μg) was digested (15 min, 37°C) with AdeI (Thermo) and BamHI (Thermo) restriction enzymes and 10 × Fast Digest Buffer (Thermo). The digested DNA was then resolved on 1% agarose gel (1 × TAE) and the band containing the FLuc sequence was excised and purified using a commercial DNA purification kit (Macherey-Nagel) according to the manufacturer protocol affording the pure insert.

#### Vector linearization

The pJet1.2 plasmid vector (2 μg, Thermo) was linearized (50 μl reaction volume) by digestion with AarI restriction enzyme (6 μl, Thermo) in 10 × AarI buffer (Thermo) and 50 × Oligonucleotide (Thermo) for 16 h at 37°C, followed by purification using a commercial DNA purification kit (Macherey-Nagel).

#### Ligation

The resulting pJET1.2 vector solution containing 100 ng of DNA was mixed with the insert (FLuc sequence) at a molar ratio of 1:3 (vector:insert). T4 DNA Ligase (1 μl, 5 U, Thermo) and 10 × Ligase buffer (Thermo) were added and the solution (total volume 20 μl) was incubated for 1 h at 25°C, followed by incubation at 4°C for an additional 1 h.

#### Plasmid amplification and sequencing

Half of the ligation mixture (10 μl, other half kept as a backup) was mixed on ice with pre-melted commercially available chemically competent bacteria Top10 (50 μl, Thermo) and incubated on ice for 30 min. The mixture was transferred to 42°C (30 sec, heat shock) and cooled on ice for 2 min. SOC (Super Optimal broth with Catabolite repression) outgrowth medium (500 μl, Thermo) was added and the solution was incubated at 37°C for 1 h (300 RPM). 200 μl of the resulting transformation mixture was spread on LB-agar plate (Roth) with 100 mg/ml ampicillin (Roth) and incubated for 16 h at 37°C. Then, single colonies were selected and inoculated in liquid LB medium (5 ml, Roth) supplemented with ampicillin (100 mg/ml, Roth), and the cultures were incubated for 16 h at 37°C. The bacterial cultures were then centrifuged (4000g, 10 min), and the plasmids were purified using the commercial GeneJET Plasmid Miniprep Kit (Thermo). The concentration was measured by spectrophotometer (Nanodrop 2000c Thermo) and plasmids were sent for DNA sequencing using the Sanger method (Genomed). The resulting pJet1.2_FLuc plasmid contained a short form of the polyA tail (∼ 30 adenine nucleotide).

**Step 2**—blunt end cloning of polyA tail: in order to obtain a DNA template with a 90 adenine nucleotides polyA tail, a multi-step procedure was carried out to insert an adenine oligonucleotide into the 3′ end of FLuc sequence. The insert was designed as a double-stranded DNA oligonucleotide and added to the pJet plasmid vector that encodes Firefly luciferase using a blunt-end cloning method.

#### Insert formation

The two solutions of DNA oligonucleotides (Genomed) with the following sequences: A_60_ (coding strand) and T_60_ (template strand) were mixed in a 1:1 ratio (final 50 μM of each DNA strand). T4 Polynucleotide Kinase (2 μl, 20 U, NEB) and 10 × buffer for T4 Polynucleotide Kinase (NEB) were added to the DNAs solution, followed by incubation for 30 min at 37°C, allowing the 5′-end phosphorylation of the oligonucleotides. The strands were then directly hybridized by heating the solution to 95°C for 2 min and slowly cooling to 25°C for 2 h (step gradient ∼2°C/∼ 3 min). The resulting DNA insert was purified using a commercial DNA purification kit (Macherey-Nagel) according to the protocol.

#### Vector linearization

The circular plasmid pJET1.2_Fluc (of ∼30 adenine nucleotide tail) encoding Firefly Luciferase (2 μg) was linearized (50 μl reaction volume) by digestion with AarI restriction enzyme (6 μl, Thermo) in 10 × AarI buffer (Thermo) and 50 × Oligonucleotide (Thermo) for 16 h at 37°C, followed by purification using a commercial DNA purification kit (Macherey-Nagel).

#### Blunt ends vector formation

The solution of linearized plasmid was incubated (50 μl) with DNA Polymerase I Large (Klenow) Fragment (15 U), 10 × buffer 3 (NEB), and 10 mM NTP (Thermo) at 25°C for 15 min, to remove 3′ overhangs and filling in 5′ overhangs to form blunt ends. The resulting pJET1.2_Fluc vector was purified using a commercial DNA purification kit (Macherey-Nagel).

#### Vector dephosphorylation

Finally, the dephosphorylation reaction (50 μl) was performed by incubation of the pJET1.2_Fluc vector using FastAP Thermosensitive Alkaline Phosphatase (5 μl, 5 U, Thermo) in 10 × Fast AP buffer (Thermo) at 37°C. for 10 min. The final pJET1.2_Fluc vector was purified using a commercial DNA purification kit (Macherey-Nagel).

#### Ligation

The resulting vector pJET1.2_Fluc solution containing 100 ng of DNA was mixed with the DNA insert at a molar ratio of 1:3 (vector:insert). T4 DNA Ligase (1 μl, 5 U, Thermo), 10 × Ligase buffer (Thermo), PEG 2000 (2 μl Thermo) and 100 mM ATP (1 μl, Thermo) were added and the solution (20 μl) was incubated at 25°C for 1 h, cooled to 4°C and incubated an additional 1 h.

Then transformation, clonal selection, and DNA sequencing were carried out analogous to the procedure described above (amplification and sequencing, step 1). The resulting sequence is shown in Supplementary Table S2.

### RNA synthesis and purification

*Short RNA (35 nt)*. The DNA template was generated by annealing single-strand oligonucleotides (CAGTAATACGACTCACTATAGGGGAAGCGGGCATGCGGCCAGCCATAGCCGATCA and TGATCGGCTATGGCTGGCCGCATGCCCGCTTCCCCTATAGTGAGTCGTATTACTG, Genomed, HPLC purified), which contains T7 promoter sequence (TAATACGACTCACTATA) and encodes 35-nt sequence GGGGAAGCGGGCATGCGGCCAGCCATAGCCGATCA. Typical *in vitro* transcription reaction (100 μl) was performed at 37°C for 2 h and contained: RNA polymerase buffer (40 mM Tris-HCl pH 7.9, 10 mM MgCl_2_, 1 mM DTT, 2 mM spermidine), 50 ng/μl T7 RNA polymerase (expressed and purified as described above), 1 U/μl RiboLock RNase inhibitor (ThermoFisher Scientific), 0.5 mM ATP/CTP/UTP, 0.125 mM GTP, 0.375 mM cap analog and 0.25 μM DNA template. Following 2 h of incubation at 37°C, an additional portion of T7 polymerase (5 μg) was added and the reaction was incubated for 2 h. Then, 1 U/μl DNase I (ThermoFisher Scientific) was added and the incubation was pursued for 30 min at 37°C. The crude RNAs were purified using NucleoSpin RNA Clean-up XS (Macherey Nagel). The quality of transcripts was verified by electrophoresis on 15% acrylamide/7 M urea/1 × TBE gels, whereas the concentration was determined spectrophotometrically (NanoDrop 2000c Thermo Scientific^®^). To remove *in vitro* transcription by-products of unintended size, RNA samples were purified by HPLC (Shimadzu apparatus), using Phenomenex^®^ Clarity Oligo-RP column at 1 ml/min, 50°C, and a linear gradient over 30 min from 10% to 30% of 200 mM TEAAc pH 7/ACN 1/1 in 100 mM TEAAc pH 7. Finally, to generate homogenous 3′-ends, the RNA transcripts (1 μM) were incubated with 1 μM DNAzyme 10–23 (TGATCGGCTAGGCTAGCTACAACGAGG-CTGGCCGC) (50) in 50 mM MgCl_2_ and 50 mM Tris–HCl pH 8.0 for 1 h at 37°C, producing 3′-homogenous 27-nt RNAs. The transcripts were purified by NucleoSpin RNA Clean-up XS (Macherey Nagel) and analyzed on 15% acrylamide/7 M urea/1 × TBE gels. The capping efficiencies were estimated by densitometry analysis of the resulting gels, as ratios between the most intense band corresponding to the capped RNA and the sum of the most intense bands of capped and uncapped RNAs.

#### Gaussia luciferase mRNA

mRNAs encoding *Gaussia* luciferase were generated on the template of pJET_T7_Gluc_128A plasmid. The plasmid was obtained by cloning the T7 promoter sequence and coding sequence of Gaussia luciferase into pJET_luc_128A (51). The plasmid was then linearized using the restriction enzyme AarI (ThermoFisher Scientific). Typical digestion reaction (40 μl) was performed for 16 h at 37°C and contained AarI buffer (10 mM Bis–Tris propane–HCl pH 6.5, 10 mM MgCl_2_,100 mM KCl, 0.1 mg/ml BSA), 10 μg of DNA plasmid, 0.5 μM of oligonucleotide (ThermoFisher Scientific) and 0.2 U/μl AarI (4 μl). The linearized DNA was purified by Nucleospin Gel & PCR Clean-up (Macherey Nagel). Typical *in vitro* transcription reaction (40 μl) was performed at 37°C for 2 h and contained RNA polymerase buffer (40 mM Tris–HCl pH 7.9, 10 mM MgCl_2_, 1 mM DTT, 2 mM spermidine), 400 ng/μl T7 RNA polymerase, 1 U/μl RiboLock RNase inhibitor (ThermoFisher Scientific), 2 mM ATP/CTP/UTP, 0.5 mM GTP, 1.5 mM trinucleotide cap analog and 50 ng/μl of digested plasmid as a template. Following 2 h of incubation, an additional portion of T7 polymerase (4 μg) was added and the reaction was incubated for 2 h. Then, 1 U/μl DNase I was added and incubation was pursued for 30 min at 37°C. When using dinucleotide analogs (ARCA and β-*S*-ARCA), a similar *in vitro* reaction was performed using RNA polymerase buffer (40 mM Tris–HCl pH 7.9, 6 mM MgCl_2_, 1 mM DTT, 2 mM spermidine) and 4 mM cap analog (i.e. a lower Mg^2+^ concentration to limit dsRNA formation and a higher cap excess compared to GTP to enhance capping efficiency). The reactions were quenched by the addition of EDTA 20 mM (50 μl) followed by precipitation with 7.5 M LiCl (100 μl). After 30 min at –20°C, the mRNA pellets were centrifuged and washed with 80% EtOH. To deplete dsRNA by-products of *in vitro* transcription, the mRNAs were then purified by HPLC (Agilent Technologies 1260), using ADS Biotec column (1 ml/min, 50°C, linear gradient 18%–26% of 200 mM TEAAc pH 7/ACN 1/1 in 100 mM TEAAc pH 7 in 20 min. The collected mRNAs were precipitated from *i*PrOH (1 μg glycogen/50 μl solution, 0.1 Volume 3 M NaOAc pH 5.2, 1 V *i*PrOH) overnight at –80°C. The pellet was then dissolved in RNase-free water (31 μl). The concentration of the transcripts was determined spectrophotometrically (1 μl, NanoDrop 2000c Thermo Scientific^®^) whereas their quality was further verified by electrophoresis on 1.2% 1 × TBE agarose gel. In order to remove the uncapped RNA from the samples, the transcripts were treated with 5′-polyphosphatase (Epicentre) and Xrn1 (New England Biolabs). During the first step (40 μl reaction volume), mRNA was incubated with 5′-polyphosphatase (1 μl/10 μg mRNA) for 30 min at 37°C in reaction buffer (500 mM HEPES–KOH pH 7.5, 0.1 M NaCl, 1 mM EDTA, 0.1% β-mercaptoethanol, and 0.01% Triton® X-100; 4 μl of commercial 10 × Buffer) in presence of 1 U/μl of RiboLock RNase inhibitor. The samples were purified by NucleoSpin RNA Clean-up XS (Macherey Nagel). In the second step (30 μl reaction volume), mRNA was incubated with Xrn1 (5 μl/10 μg mRNA) for 1 h at 37°C in NEB 3 buffer (50 mM Tris–HCl, 100 mM NaCl, 10 mM MgCl_2_, 1 mM DTT, pH 7.9; 3 μl of commercial 10 × Buffer), in presence of 1 U/μl of RiboLock RNase inhibitor. The samples were again purified by NucleoSpin RNA Clean-up XS (Macherey Nagel).

### RNA decapping assay

Human Dcp2 was expressed in *E. coli* and purified as described previously (52). Short 27 nt-capped RNAs (24.2 ng) were diluted with decapping buffer (50 mM Tris–HCl pH 8.0, 50 mM NH_4_Cl, 0.01% NP-40, 1 mM DTT, 5 mM MgCl_2_ and 2 mM MnCl_2_; 2.9 μl of freshly prepared 10 × Buffer) and the final volume was adjusted to 29 μl with ultrapure RNase free water. 4.2 ng (5 μl) of the solution was taken out as time zero reference and diluted with 5 μl loading dye (5 M urea, 44% formamide, 20 mM EDTA, 0.03% bromophenol blue, 0.03% xylene cyanol). The remaining RNAs (20 ng/24 μl) were subjected to 10 nM *h*Dcp2 (250 nM, 1 μl) and the mixtures were incubated at 37°C for 1 h. At defined time points (5, 15, 30 and 60 min), 4 ng of RNA (5 μl) were taken out and the reactions were quenched by adding an equal volume of loading dye. RNAs were resolved electrophoretically on denaturing 15% acrylamide:bisacrylamide 19:1/7 M urea/1 × TBE gel, stained with SYBR Gold (Invitrogen) and visualized using a Typhoon FLA 9500 (GE Healthcare). The decapping progress was determined for each time point by densitometry analysis of the gels, as a ratio between the intensity of the band corresponding to capped RNA and the sum of the intensity of bands from capped and uncapped RNAs. The percentages of remaining capped RNA were then plotted as a function of time.

### Protein production assay in cell lines

A549 cells (human epithelial lung carcinoma, ATCC CCL-185) were grown in DMEM medium (Gibco) supplemented with 10% FBS (Sigma), GlutaMAX (Gibco) and 1% penicillin/streptomycin (Gibco) at 5% CO_2_ and 37°C. Murine immature dendritic cell line JAWS II (ATCC CRL-11904) was grown in RPMI 1640 (Gibco) supplemented with 10% FBS, sodium pyruvate (Gibco), 1% penicillin/streptomycin and 5 ng/ml GM-CSF (PeproTech) at 5% CO_2_ and 37°C. For every experimental repetition cells within 5 and 25 passages were used. The A549 and JAWS II cells (10^4^ per well) were seeded in 96-well plate 4 h before transfection in 100 μl medium without antibiotics. Cells in each well were transfected using a mixture of 0.3 μl Lipofectamine MessengerMAX transfection reagent (Invitrogen) and 25 ng of mRNA encoding *Gaussia* luciferase in 10 μl Opti-MEM (Gibco) for each studied 5′ cap modification. The experiment was conducted for 88 h after transfection, setting 4 time points for signal collection (16 h, 40 h, 64 h and 88 h after transfection). To assess the expression of *Gaussia* luciferase, plates were centrifuged (5 min, 400 g, 25°C) and the medium was fully removed and replaced with the fresh one (100 μl) at each time point. Luminescence detection from *Gaussia* luciferase was carried out by adding 50 μl of 10 ng/ml h-coelenterazine (NanoLight) in PBS to 10 μl of cell-cultured medium for each modification and the luminescence was measured on Synergy H1 (BioTek) microplate reader. Total protein expression for each mRNA over 88 h was reported as a mean value ± SD normalized to m^7^GpppA_m_pG-RNA.

### Affinity chromatography with cellular extract

The protein extract from HEK293F cells was prepared according to the published protocol (53). The resins (100 μl of the settled resin) were loaded into 2 ml columns equipped with a filter and washed first with water (3 × 2 ml) and then with buffer A (50 mM HEPES pH 8.0, 100 mM KCl, 0.5 mM EDTA; 3 × 2 ml). The outlets of the columns were closed and 1 ml of protein extract from HEK293F cells (10.85 mg/ml stocks diluted 5-times with buffer A) with 200 μM GTP was added. The columns were tightly closed and shaken gently at 4°C overnight. The resins were washed with buffer A (5 × 2 ml) and the proteins were eluted with buffer B (buffer A + 200 μM cap analog: m^7^GpppA_m_pG for **AR-1**, **11** for **AR-2** or **14** for **AR-3**; 2 × 200 μl).

To the solutions of proteins eluted from the beads, TCEP (10 mM) and chloroacetamide (15 mM) were added, and the mixtures were subjected to overnight enzymatic digestion (0.5 μg, Sequencing Grade Modified Trypsin, Promega) at 37°C. Tryptic peptides were then desalted with the use of AttractSPE™ Disks Bio C18 (Affinisep) and TMT-labelled on the solid support (54). Individual TMT-labelled samples (3 replicates of **AR-1**, 3 replicates of **AR-2**, 3 replicates of **AR-3**) were compiled into a TMT-9 dataset and concentrated using a SpeedVac concentrator. Prior to LC-MS measurement, the sample was resuspended in 0.1% TFA, 2% acetonitrile in water. Chromatographic separation was performed on an Easy-Spray Acclaim PepMap column 50 cm long × 75 μm inner diameter (Thermo Fisher Scientific) at 55°C by applying a 120 min acetonitrile gradients in 0.1% aqueous formic acid at a flow rate of 300 nl/min. An UltiMate 3000 nano-LC system was coupled to a Q Exactive HF-X mass spectrometer via an easy-spray source (all Thermo Fisher Scientific). The spectrometer was operated in TMT mode with survey scans acquired at a resolution of 60 000 at *m*/*z* 200. Up to 15 of the most abundant isotope patterns with charges 2–5 from the survey scan were selected with an isolation window of 0.7 *m*/*z* and fragmented by higher-energy collision dissociation (HCD) with normalized collision energies of 32, while the dynamic exclusion was set to 35 s. The maximum ion injection times for the survey scan and the MS/MS scans (acquired with a resolution of 45 000 at *m*/*z* 200) were 50 and 96 ms, respectively. The ion target value for MS was set to 3e6 and for MS/MS to 1e5, and the minimum AGC target was set to 1e3. The data were processed with MaxQuant v. 1.6.17.0 (55), and the peptides were identified from the MS/MS spectra searched against the reference human proteome UP000005640 (Uniprot.org) using the built-in Andromeda search engine. Cysteine carbamidomethylation was set as a fixed modification, and methionine oxidation and protein N-terminal acetylation were set as variable modifications. For *in silico* digests of the reference proteome, cleavages of arginine or lysine followed by any amino acid were allowed (trypsin/P), and up to two missed cleavages were allowed. Reporter ion MS2 quantification was performed with the min. reporter PIF was set to 0.75. The FDR was set to 0.01 for peptides, proteins and sites. The second peptide search was disabled. Other parameters were used as pre-set in the software. Unique and razor peptides were used for quantification enabling protein grouping (razor peptides are the peptides uniquely assigned to protein groups and not to individual proteins). Data were further analysed using Perseus version 1.6.10.0 (56), and Microsoft Office Excel 2016. Reporter intensity corrected values for protein groups were loaded. Standard filtering steps were applied to clean up the dataset: reverse (matched to decoy database), only identified by site, and potential contaminants (from a list of commonly occurring contaminants included in MaxQuant) protein groups were removed. Reporter intensity values were Log2 transformed and normalized by median subtraction within TMT channels. 1302 Protein groups identified by at least 2 razor peptides and with a complete set of 9 values were subjected to further analysis. Two-sided T-tests (permutation-based FDR = 0.02, S_0_= 1) were performed for three comparisons of sample groups: **AR-3** versus **AR-1**; **AR-2** versus **AR-1**; **AR-3** vs **AR-2** to identify proteins differently bound to the resins under investigation. The raw LC–MS/MS data and the output from MaxQuant have been deposited to the ProteomeXchange Consortium (57) via the PRIDE (58) partner repository with the dataset identifier PXD053198.

### *In vivo* studies

#### mRNA synthesis and purification

Two reporters were selected to determine *in vivo* protein production: firefly luciferase (FLuc) and human erythropoietin (hEPO). The mRNA encoding each gene was obtained by IVT using T7 RNA polymerase for co-transcriptional capping with m^7^Gppp^5′S^A_m_pG and m^7^GpppA_m_pG as the reference cap structure. 500 μl or 1000 μl of the IVT mix (for obtaining mRNA encoding hEPO or FLuc, respectively) contained following components at indicated final concentration: transcription buffer with 2 mM spermidine and Bis-Tris pH 6.5, 1 U/μl RNase inhibitor (RiboLock, ThermoFisher Scientific), 5 mM ATP, CTP and UTP, 4 mM GTP, 10 mM DTT, 25 mM MgCl_2_, 0.002 U/μl inorganic pyrophosphatase (ThermoFisher Scientific), 10 mM m^7^GpppA_m_pG or m^7^Gppp^5’S^A_m_pG, 40 ng/μl linearized plasmid as the DNA template and 0.125 μg/μl T7 RNA polymerase (in-house prepared). All components were gently mixed by pipetting and incubated for one hour at 37°C. After DNA template removal, obtained mRNA was initially purified using oligo(dT)_25_ resin, as previously described, and concentrated by ultrafiltration (Amicon Ultra-15 50K or 100K, Millipore) prior loading on preparative column. To remove contaminants, such as dsRNA and short mRNA species, second purification step was applied. Each mRNA sample was purified by RP-HPLC on an Agilent Infinity 1260 II using RNASep^TM^ Semi-Prep columns (ADS Biotec). mRNA was eluted with the linear gradient (10–14.5%) of acetonitrile in 0.1 M TEAA pH 7.0 at 55°C. Eluate quality was analyzed on 1 × TBE 1.2% agarose gel, and the fractions containing mRNA of the highest purity were combined, desalted by ultrafiltration (Amicon Ultra-15 50K or 100K, Millipore), and precipitated. Prior formulation and quality control, mRNA pellets were resuspended in RNase-free water to obtain concentration of >1 μg/μl and—if necessary—stored at −80°C as single-use aliquots.

#### Lipid nanoparticle preparation in GenVoy-ILM^TM^ mix

The ionizable lipid mix GenVoy-ILM™ was purchased from Precision NanoSystems, Canada, and diluted 1:1 (v/v) with absolute ethanol to final concentration of 12.5 mM. All mRNAs were diluted in 100 mM sodium citrate buffer pH 4.0 at the final concentration of 120 ng/μl. The GenVoy-ILM^TM^ and mRNA solution were combined in a microfluidic device (NanoAssemblr^®^ Ignite™, Precision NanoSystems) equipped with NxGen Cartridge (cat#NIN0002) at a flow ratio of 3:1 (aqueous phase:ethanol) with a total flow rate of 12 ml/min. The final N/P ratio was 6, where N/P represents the ratio of ionizable nitrogen atoms to phosphate groups in the mixture.

#### Lipid nanoparticle preparation with SM-102 and MC3 lipids

The SM-102 and MC3 (DLin-MC3-DMA) lipids were purchased from BroadPharm (USA). The 1,2-dimyristoyl-rac-glycero-3-methoxypolyethylene glycol-2000 (DMG-PEG2k), cholesterol (from ovine wool) and 1,2-dioctadecanoyl-*sn*-glycero-3-phosphocholine (DSPC) were purchased from Avanti Polar Lipids (USA). The stock solutions of lipids were prepared in absolute ethanol (Thermo Fisher Scientific) at the concentration of 100 mg/ml for SM-102 and MC3 and 10 mg/ml for the rest of the lipids. The lipid mixes were prepared by combining SM-102/MC3, DSPC, cholesterol and DMG-PEG2k at molar ratio of 50:10:38.5:1.5 in absolute ethanol at total concentration of 15 mM for SM-102 lipid mix and 12.5 mM for MC3 lipid mix. Stock solution of mRNA was diluted in 100 mM sodium citrate buffer pH 4.0 at the mRNA final concentration of 102 ng/μl for SM-102 lipid mix and 120 ng/μl MC3 lipid mix. The lipid mix and mRNA solution were combined together in a microfluidic device (NanoAssemblr^®^ Ignite™, Precision NanoSystems) equipped with NxGen Cartridge (cat#NIN0002) at a flow ratio of 4:1 with a total flow rate of 12 ml/min for SM-102 lipid mix and at a flow ratio 3:1 with a total flow rate 10 ml/min for MC3 lipid mix. The final N/P ratio was 6, where N/P represents the ratio of ionizable nitrogen atoms to phosphate groups in the mixture. All resulting LNP nanoparticles (LNPs) were diluted 20–40 times in 1 × PBS sterile buffer (w/o calcium and magnesium ions) and concentrated by ultrafiltration using Amicon ultracentrifugal tubes (Merck Millipore) with 50 kDa MWCO (2000 × g, 20°C). Final LNPs were stored at 4°C and diluted in 1 × PBS (w/o calcium and magnesium ions) sterile buffer before application into mice.

#### LNPs size and encapsulation efficiency measurement

Size distribution and polydispersity index were determined using dynamic light scattering (DLS) on Malvern Zetasizer Ultra Red (Malvern, UK) in PBS buffer at 25°C in back scatter mode. Encapsulation efficiency and concentration of mRNA entrapped in LNPs was determined using the Quant-iT Ribogreen RNA assay (Thermo Fisher Scientific, USA) by compering fluorescence intensities in the presence or absence of 0.1% (w/v) Triton X-100.

#### Mice

The experiments were carried out in 10–12-week-old (∼25 g) female C57BL/6 mice, under a protocol approved by the Local Ethical Committee for Experiments on Animals in Warsaw, Poland (WAW2/063/2023) and in 10–12-week-old (∼25 g) female BALB/c mice under a protocol approved by the Local Ethical Committee for Experiments on Animals in Warsaw, Poland (WAW2/126/2021). All experiments were conducted in accordance with the Directive of the European Parliament and Council No. 2010/63/EU on the protection of animals used for scientific purposes. Mice were obtained from the Breeding Facility of the Mossakowski Institute, Polish Academy of Science, Warsaw and maintained in specific pathogen-free (SPF) environment in the individually ventilated cages (IVC) under the conditions of a 12-h day/night cycle with unrestricted access to food and drinking water.

#### Assessment of *in vivo* translation efficacy of FLuc-encoding mRNA-LNPs

mRNA coding for FLuc was administered intravenously (i.v.) into the lateral tail vein of BALB/c mice in different formulations. Each mouse was inoculated with 10 μg of LNP-formulated mRNA. The total body bioluminescent signal was recorded intravitally at 4, 8 and 24 h post mRNA administration with IVIS Spectrum imaging system (Perkin-Elmer). Briefly, mice were administered intraperitoneally 150 mg/kg d-luciferin (Sydlabs) and anesthetized by continuous inhalation of 3% isoflurane (Baxter) mixture in 100% O_2_. Imaging conditions were as follows: auto acquisition time set on Auto (max 1 min), F/Stop 1 and binning small. Bioluminescence values in the regions of interest (ROIs) were analysed using Living IMAGE Software provided by Perkin-Elmer.

#### Assessment of *in vivo* translation efficacy of hEPO-encoding mRNA-LNPs

mRNA coding for hEPO was administered i.v. into the lateral tail vein of C57BL/6 mice in different formulations. Each mouse was inoculated with 1 μg of LNP-formulated mRNA. Blood was collected at 4, 8, 24 and 48 h time points after mRNA administration. hEPO serum concentrations were measured with ELISA (ThermoFisher).

## Results and discussion

### Design and synthesis of cap analogs

The structures of the trinucleotide cap analogs were designed to be complementary to the most common T7 RNA polymerase promoter sequences (Φ6.5 and Φ2.5), thus maximizing capping efficiency during the *in vitro* transcription reaction. Our previous studies have shown that among analogs of the m^7^GpppNpG structure, the most efficient transcription initiators are m^7^GpppApG derivatives (12), therefore we focused our attention on this structure and combined it with the modifications previously characterized as most favorable in the context of dinucleotide anti-reverse cap analogs (ARCAs, m_2_^7,2′-^*^O^*GpppG derivatives). Those included β-phosphorothioates (non-bridging O-to-S), 5′-α-phosphorothiolates (5′-O-to-S), α,β-methylenebisphosphonates (O-to-CH_2_), and tetraphosphates, along with β,γ-dichloromethylenebisphosphonate (O-to-CCl_2_) which performed best in terms of translation efficiency and decapping susceptibility (Figure 1, Supplementary Table S1). Some of the trinucleotide analogs were prepared in different variants in terms of their methylation status, i.e. cap-0 (m^7^GpppApG), cap-1 (m^7^GpppA_m_pG—‘m’ subscript codes for 2′-*O*-methylation of the ribose) and m^6^A_m_ cap-1 (*N*6,2′-*O*-dimethyladenosine) derivatives, to test if the phosphate modifications affect different natural methylations of the cap in the same way.

**Figure 2.**
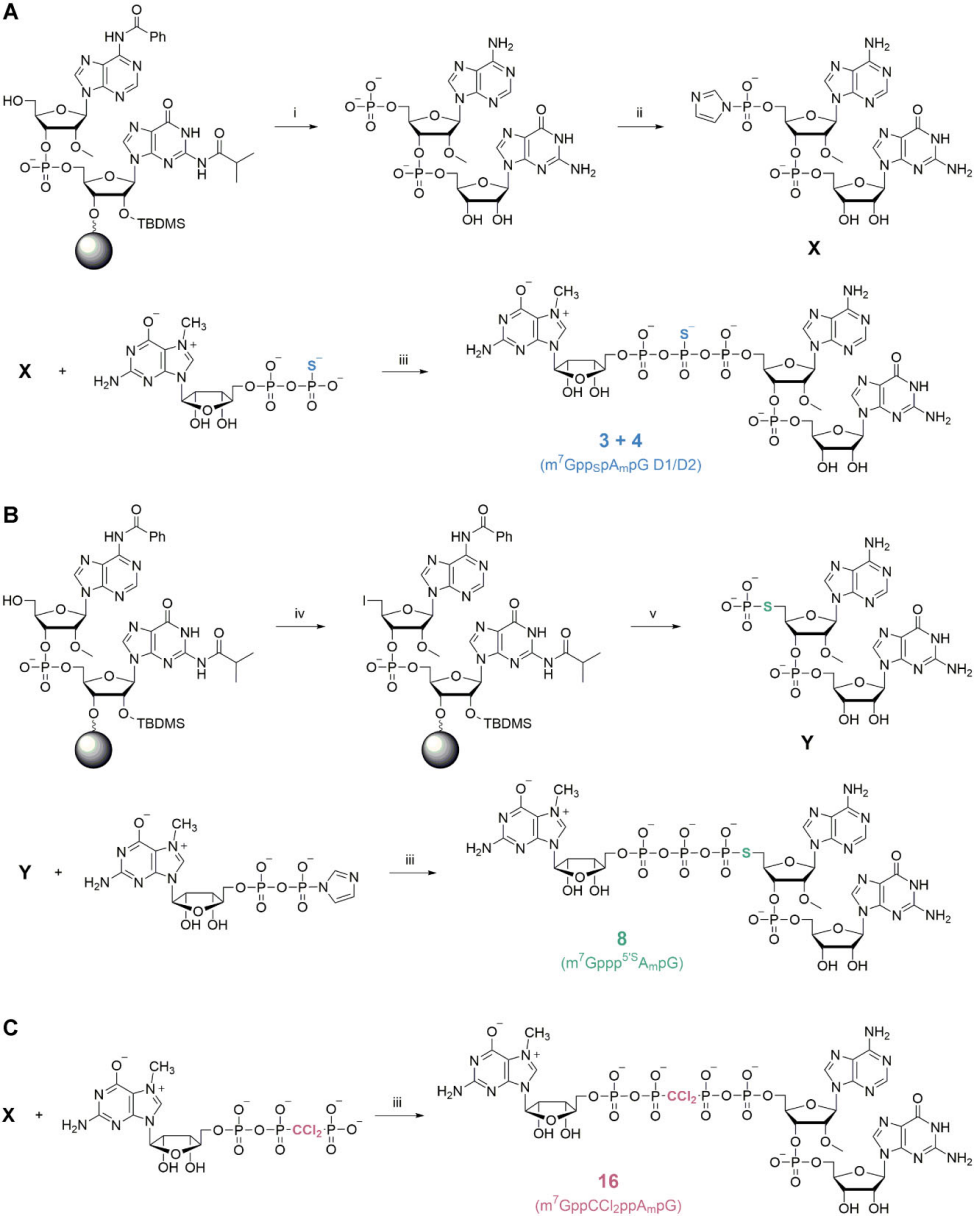
Synthesis of cap analogs 3, 4, 8 and 14. Reaction conditions: i. 1) bis-cyanoethylphosphoramidite, benzylthiotetrazole, acetonitrile; 2) I_2_, pyridine/water; 3) diethylamine, acetonitrile; 4) AMA, 37°C, 3 h; 5) TEA·3HF, DMSO, 65°C, 2 h; ii. imidazole, 2,2′-ditiodipiridine, triphenylphosphine, triethylamine, DMF; iii. ZnCl_2_, DMF; iv. triphenoxymethylphoshonium iodide, DMF; v. 1) triethylammonium thiophosphate, DMF; 2) AMA, 37°C, 3 h; 3) TEA·3HF, DMSO, 65°C, 2 h;

All the trinucleotide cap analogs **1–16** were synthesized by a combination of P(III) and P(V) chemistry. First, the dinucleotide precursors linked by the 3′,5′-phosphodiester moiety were synthesized by the phosphoramidite method on a high-loaded polystyrene support, and then the 5′,5′-tri(tetra)phosphate bridge was formed in solution in a ZnCl_2_ mediated coupling reaction using a *P*-imidazolide activation strategy (Figure 2, Supplementary Figure S1). Every modification of the triphosphate bridge required a different synthetic approach, depending on the stability and reactivity of the synthons.

To obtain β-phosphorothioate cap analogs **1**–**6** (Figure 2A, Supplementary Figure S1A), it was necessary to convert the dinucleotide 5′-phosphates **20–22** into *P*-imidazolides, as there is no straightforward synthetic route to obtain the phosphorimidazolide derivative of 7-methylguanosine 5′-*O*-(*P*2-thiodiphosphate) (m^7^GDP-β-S). Reactions of activated dinucleotides **23–25** with m^7^GDP-β-S were completed within 5–10 minutes, yielding an approximately 1:1 diastereomeric mixture of target trinucleotides **1–6**. The diastereomers were separated by RP-HPLC and their *R*_P_/*S*_P_ absolute configurations at the β-phosphorus atom were assigned based on the previous studies of cap-eIF4E complexes by X-ray crystallography (59). The synthesis of the 5′-phosphorothiolate analogs (compounds **7** and **8**) involved the conversion of the terminal 5′-OH group of the solid-supported dinucleotides to 5′-iodides, according to the previously published reports (60–62), and subsequent nucleophilic substitution with thiophosphate (Figure 2B, Supplementary Figure S1B). The cleaved and deprotected dinucleotides **26–27** were then reacted with 7-methylguanosine 5′-*O*-diphosphate *P*-imidazolide (m^7^GDP-Im) to give the desired trinucleotide analogs **7** and **8**. To obtain the α,β-methylenebisphosphonate analog **9**, we attempted the solid-phase synthesis of the dinucleotide 5′-methylenebisphosphonate (Supplementary Figure S1C). To this end, we reacted the base-protected 5′-OH dinucleotide, while still on the solid support, with methylenebis(phosphonic dichloride) followed by hydrolysis of the phosphonic chloride. We found that the reaction is not easily reproducible and often leads to a 5′-chlorodinucleotide derivative along with some other side products. Still, we were able to isolate a small amount of dinucleotide 5′-methylenebisphoshonate **28** and react it with the 7-methylguanosine 5′-*O*-monophosphate *P*-imidazolide (m^7^GMP-Im) to obtain the trinucleotide cap **9**. Finally, the trinucleotide cap analogs with unmodified or modified tetraphosphate chain (compounds **10**–**16**) were synthesized using dinucleotide *P*-imidazolides **23–25** and 7-methylguanosine 5′-*O*-triphosphate (m^7^GTP) or its *P*2,*P*3-methylenebisphosphonate (m^7^GppCH_2_p) or *P*2,*P*3-dichloromethylenebisphosphonate (m^7^GppCCl_2_p) derivatives (Figure 2C, Supplementary Figure S1D).

### Biochemical properties of trinucleotide cap analogs

#### Binding affinities for eIF4E

**Figure 3.**
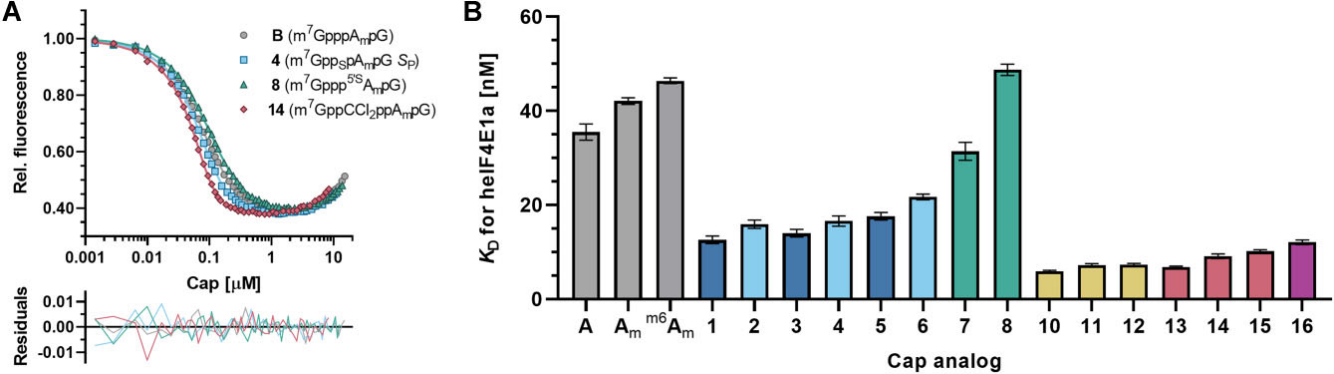
Modified trinucleotide cap analogs bind to eIF4E with high affinity. (**A**) representative binding curves; (**B**) Bar plot of *K*_D_ values for trinucleotide caps 1–8 and 10–16 along with reference compounds m^7^GpppA_m_pG (A_m_) and m^7^Gppp^m6^A_m_pG (^m6^A_m_). For color coding see Figure 1 caption.

Recognition of the mRNA 5′ cap by translation initiation factor 4E (eIF4E) is considered to be the rate-limiting step for the entire translation process (63,64), therefore we investigated the affinity of novel trinucleotide cap analogs to the canonical human eIF4E1a protein by fluorescence quenching titration (FQT; Table 1 column 1, Figure 3) (65). As a reference, we determined the dissociation constant between heIF4E1a and the trinucleotide cap-1 structure m^7^GpppA_m_pG, which was equal to 42 nM and very close to the analogous value previously determined for mouse Δ27NeIF4E (46 nM) (12). In the case of all modifications tested, the *K*_D_ values for cap-heIF4E1a complexes are consistent with the results previously obtained for dinucleotide counterparts and murine eIF4E protein, although the magnitude of the modification effect is generally lower. The most tightly bound tetraphosphate analogs (compounds **10–12**) show *K*_D_ values of 6–7 nM, which means that their affinity is about 6-fold higher compared to the triphosphate counterparts. The affinities decrease upon CH_2_ or CCl_2_ modification of the tetraphosphate bridge (compounds **13–16**), but they are still 4–5 times higher than for the triphosphate analogs with the same adenosine methylation pattern. The β-phosphorothioate modification (compounds **1–6**) results in a 2–3 fold increase in binding affinity as compared to the corresponding triphosphates with a slight preference for *R*_P_ diastereomers, whereas the 5′-α-phosphorothiolate modification (compounds **7** and **8**) has virtually no effect on binding affinity.

**Table 1.** Biochemical properties of novel cap analogs

Cap analog	heIF4E1^a^*K*_D_ (nM)	Capping efficiency^a^	Susceptibility to hDcp2 (@30 min)^b^	Normalized protein expression in JAWS II^a^	Normalized protein expression in A549^a^
**m^7^GpppApG**	**35.5 ± 1.7**	**88%** ^c^	**0.90 ± 0.04**	n.d.	n.d.
**m^7^GpppA_m_pG**	**42.1 ± 0.7**	**90%** ^c^	**0.80 ± 0.14**	**1** **± 0.43**	**1** **± 0.49**
**m^7^Gppp^m6^A_m_pG**	**46.4 ± 0.6**	**75%** ^c^	**0.67 ± 0.02**	n.d.	n.d.
**m_2_^7,3^′^-O^GpppG**	n.d.	n.d.	n.d.	0.26 ± 0.19	0.55 ± 0.15
**m_2_^7,2^′^-O^Gpp_S_pG *R*_P_ (D1)**	n.d.	n.d.	n.d.	1.36 ± 0.21	0.79 ± 0.22
**1 (m^7^Gpp_S_pApG *R*_P_)**	12.6 ± 0.8	86 ± 7%	0.81 ± 0.16	0.81 ± 0.09	0.73 ± 0.25
**2 (m^7^Gpp_S_pApG *S*_P_)**	15.9 ± 0.9	87 ± 4%	0.31 ± 0.09	0.65 ± 0.27	0.28 ± 0.05
**3 (m^7^Gpp_S_pA_m_pG *R*_P_)**	14.0 ± 0.8	88 ± 8%	0.82 ± 0.09	1.16 ± 0.21	1.17 ± 0.02
**4 (m^7^Gpp_S_pA_m_pG *S*_P_)**	16.6 ± 1.1	93 ± 3%	0.32 ± 0.10	1.26 ± 0.23	1.08 ± 0.11
**5 (m^7^Gpp_S_p^m6^A_m_pG *R*_P_)**	17.6 ± 0.8	59 ± 14%	0.62 ± 0.07	1.13 ± 0.43	1.10 ± 0.08
**6 (m^7^Gpp_S_p^m6^A_m_pG *S*_P_)**	21.7 ± 0.6	61 ± 11%	0.15 ± 0.10	1.20 ± 0.19	1.09 ± 0.15
**7 (m^7^Gppp^5’S^ApG)**	31.4 ± 1.9	80 ± 2%	0.60 ± 0.15	0.46 ± 0.24	0.64 ± 0.09
**8 (m^7^Gppp^5’S^A_m_pG)**	48.7 ± 1.2	85 ± 5%	0.64 ± 0.11	1.32 ± 0.16	1.18 ± 0.22
**9 (m^7^GppCH_2_pA_m_pG)**	n.d.	88%^d^	n.d.	0.63 ± 0.17	0.68 ± 0.20
**10 (m^7^GppppApG)**	5.9 ± 0.2	86 ± 2%	0.65 ± 0.09	0.04 ± 0.01	0.10 ± 0.03
**11 (m^7^GppppA_m_pG)**	7.2 ± 0.3	86 ± 2%	0.87 ± 0.05	0.02 ± 0.01	0.11 ± 0.02
**12 (m^7^Gpppp^m6^A_m_pG)**	7.3 ± 0.3	65 ± 15%	0.40 ± 0.11	0.04 ± 0.01	0.06 ± 0.01
**13 (m^7^GppCCl_2_ppApG)**	6.8 ± 0.2	89 ± 5%	0.03 ± 0.02	0.13 ± 0.05	0.26 ± 0.02
**14 (m^7^GppCCl_2_ppA_m_pG)**	9.1 ± 0.5	90 ± 4%	0.01 ± 0.00	0.10 ± 0.03	0.30 ± 0.01
**15 (m^7^GppCCl_2_pp^m6^A_m_pG)**	10.2 ± 0.3	74 ± 14%	0.03 ± 0.00	0.11 ± 0.01	0.23 ± 0.03
**16 (m^7^GppCH_2_ppA_m_pG)**	12.1 ± 0.5	84 ± 3%	0.27 ± 0.07	0.04 ± 0.02	0.16 ± 0.04

^a^The average values from triplicates ± SD are shown.

^b^Fraction of decapped RNA after 30 min of incubation with hDcp2. The average values from triplicates ± SD are shown.

^c^Data from Sikorski *et al.* (12).

^d^Value from one replicate only.

#### Incorporation into short RNA and capping efficiency determination

**Figure 4.**
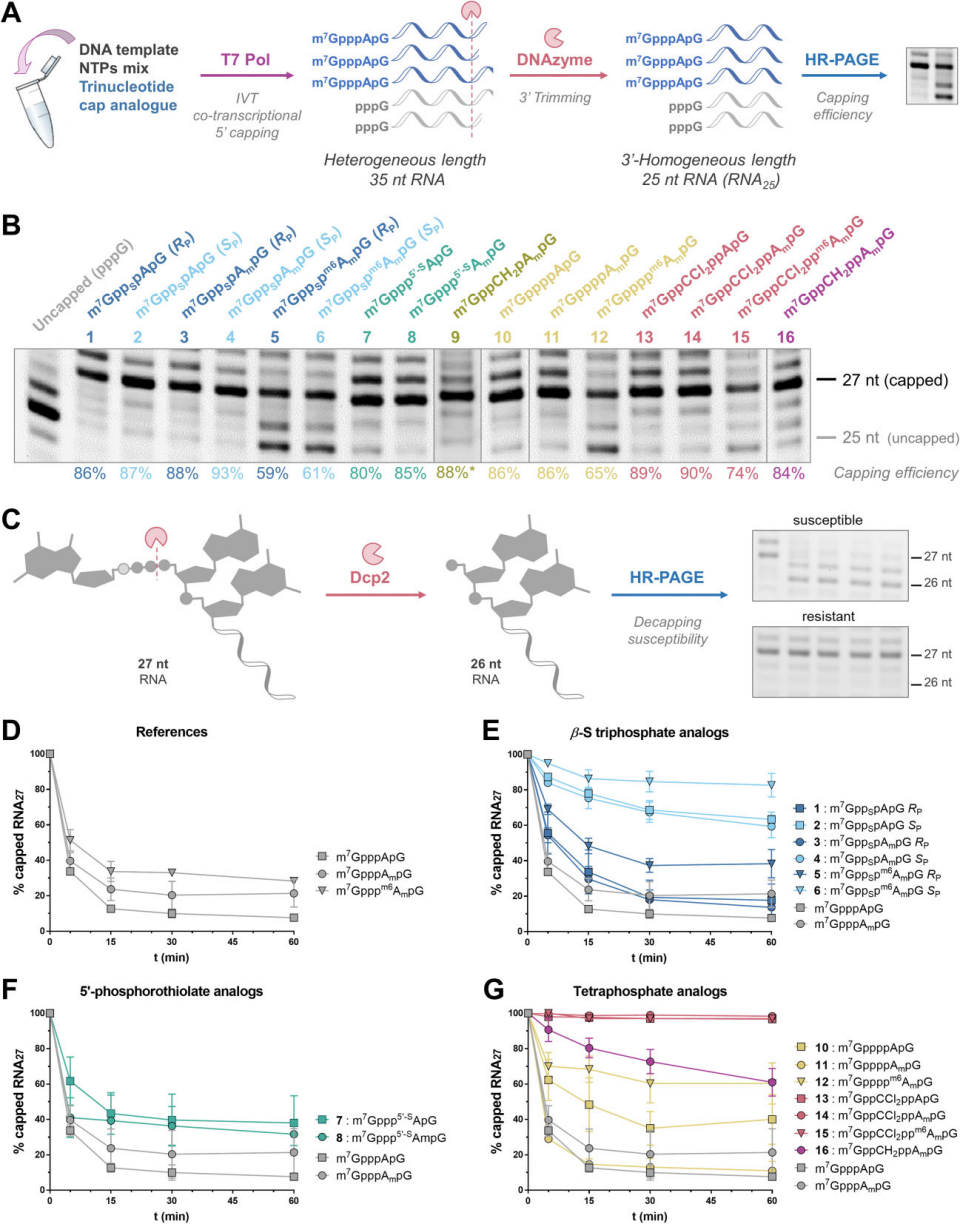
(**A**) Synthesis of capped RNA_27_ via co-transcriptional capping (IVT) and 3′-trimming (DNAzyme) for capping efficiency assessment. (**B**) HRPAGE (7 M urea, 15% PAA, 1 × TBE) of resulting capped RNA27 and capping efficiency values determined densitometrically as the average of three independent repetitions. The gel lanes are issued from 2 separated gels and have been re-ordered according to the compounds numbering. Uncut gels are shown in Supplementary Figure S3. The quantitative summary of the results is shown in Table 1. (**C**) General scheme of the decapping susceptibility assay. D–G. Relative susceptibility of capped RNA27 to recombinant hDcp2 (conditions: 20 ng RNA/ 24 μl; 50 mM Tris-HCl pH 8.0, 50 mM NH_4_Cl, 0.01% NP-40, 1 mM DTT, 5 mM MgCl_2_ and 2 mM MnCl_2_). The data displayed are the results of three independent replicates; (**D**) Unmodified capped RNAs (references); (**E**) *β*–S triphosphate capped RNAs; F. 5′-phosphorothiolate capped RNAs; (**G**) Tetraphosphate capped RNAs. Gels electrophoresis of triplicates are shown in Supplementary Figure S2. The detailed susceptibility at 30 min is displayed in Table 1. For color coding see Figure 1 caption.

Next, we used a two-step enzymatic strategy to determine how efficiently the trinucleotides were incorporated into RNA during *in vitro* transcription (IVT) with T7 polymerase and a DNA template containing a Φ6.5 promoter (with A^-1^G^+1^G^+2^G^+3^ sequence) followed by 35 nucleotides (Figure 4A). An excess of trinucleotide (5×) over GTP (the first nucleotide transcribed in the absence of a cap analog) favored transcription initiation with the trinucleotide cap analog (44). The use of a m^7^GpppApG-type trinucleotide allowed pairing with the DNA template at the −1 and + 1 positions (12), resulting in 37-nt capped RNA as the major transcription product, whereas uncapped RNA (5′-triphosphate) was 35 nt long. The RNAs were purified by RP-HPLC (necessary for efficient further processing), trimmed with a sequence-specific DNAzyme (50), to reduce RNA 3′ heterogeneity (66,67), and analyzed by high-resolution polyacrylamide gel electrophoresis (PAGE). Capping efficiencies were estimated by densitometric analysis of the gel bands as the ratio of the capped RNA band (27-nt) to total RNA (a sum of uncapped 25-nt RNA and capped 27-nt RNA; Figure 4B, Table 1).

The presence of intense bands that migrated slower than the band corresponding to uncapped RNA (25-nt) confirmed that all trinucleotides were efficiently incorporated into the RNA. For all cap analogs containing A or A_m_ as the first transcribed nucleotide, we observed high capping efficiencies ranging from 80 to 93%, regardless of the modification type. There were also no significant differences between the two *S*_P_/*R*_P_ diastereomers of the β-thiophosphate derivatives (lanes 1, 3, and 5 versus lanes 2, 4, and 6, respectively, Figure 4B). However, when m^6^A_m_ derivatives were used as transcription initiators, the capping efficiencies decreased significantly (to 60–74%), suggesting that the *N*6-methylation of adenosine interferes with base-pairing to the template at the −1 position. Interestingly, the tetraphosphate derivatives (lanes 10–16, Figure 4B) yielded RNAs that migrated slightly slower than the triphosphate analogs, most likely due to the additional negatively charged phosphate group.

### Biological properties of RNAs capped with analogs 1–16

#### Analysis of decapping susceptibility

The RNAs used to determine the capping efficiencies were also useful to study the susceptibility of capped RNA to decapping by hDcp2, the catalytic subunit of the major human mRNA decapping complex Dcp1/Dcp2, which releases m^7^GDP and 5′-monophosphorylated RNA (in this case the 26-nt pApG-RNA_24_; Figure 4C). The progress of decapping at different time points (5, 15, 30 and 60 min) was monitored by HR-PAGE followed by densitometric analysis of the gels (Figure 4C, triplicates of gels are shown in Supplementary Figure S2), and the fraction of remaining capped RNA (27-nt) was plotted as a function of time (Figure 4D-G). Unmodified trinucleotides (m^7^GpppApG, m^7^GpppA_m_pG and m^7^Gppp^m6^A_m_pG) were used as references and treated similarly, revealing little difference in decapping susceptibility between the different adenosine methylation patterns (Figure 4D). For better visualization, the data of modified capped RNAs were grouped according to the type of modification present at the 5′ end and presented in separate graphs (Figure 4E–G). To quantify the relative susceptibility to hDcp2, we compared the decapping levels at the 30 min time point (Table 1). For RNAs capped with β-S-triphosphate analogs (**1**–**6**, Figure 4E, Table 1), we observed a significant difference in susceptibility to hDcp2 between *R*_P_ and *S_P_* isomers, independent of the methylation pattern. The *S*_P_ isomers were significantly less susceptible to hDcp2 than *R*_P_ isomers, which in turn were slightly less susceptible than the corresponding unmodified caps, in agreement with previous observations made for dinucleotides (34). RNAs carrying 5′-phosphorothiolate caps (**7**–**8**) showed susceptibilities between the corresponding β-S *R*_P_ and *S*_P_ caps (Figure 4F, Table 1). Surprisingly, all three RNAs carrying unmodified tetraphosphate caps **10**–**12** (A, A_m_, m^6^A_m_) showed markedly different susceptibility to decapping (Figure 4G), with no clear correlation between the number of methyl groups and hydrolysis rate. As expected, the presence of a methylene bisphosphonate modification at the β,γ-position of the tetraphosphate chain **16** (Figure 4G, Table 1) significantly reduced the susceptibility to decapping, and the dichloromethylene bisphosphonate analogs **13**–**15** (Figure 4G, Table 1) showed complete resistance under the experimental conditions, regardless of their methylation status.

#### Translational activity in cultured cells

Next, we moved to the full-length mRNAs coding for *Gaussia* Luciferase (*G*Luc) and tested the influence of each mRNA cap modification on the protein production in cultured cells. A series of mRNAs capped with analogs **1**–**16** were synthesized by *in vitro* transcription using T7 polymerase and the appropriate DNA template containing the Φ6.5 promoter. In addition, uncapped mRNA was synthesized as a negative control and three capped mRNAs were used as well established positive controls: mRNA with unmodified cap-1 trinucleotide (m^7^GpppA_m_pG), mRNA capped with 3′-ARCA (m_2_^7,3′-O^GpppG)—a dinucleotide cap used as a reference compound in many previous studies (31,34,37)—and mRNA capped with β-S-ARCA *R*_P_ (m_2_^7,2′-O^Gpp_S_pG)—a dinucleotide with favorable translational properties leading to clinical applications in anti-cancer vaccines (36).

As demonstrated for short RNAs, IVT capping is not complete and a small amount of uncapped mRNA was expected to be present in the samples, which could not be separated by gel electrophoresis or RP HPLC for such long mRNA. To eliminate the effect of varying concentrations of uncapped RNA (along with other contaminants) on protein expression, we performed a two-step digestion of the IVT RNAs to remove the uncapped portion from all samples (39). The HPLC-purified mRNAs (to remove double-stranded RNA) were treated with polyphosphatase to selectively hydrolyze the 5′ triphosphate of uncapped RNAs. Xrn1 5′-exonuclease was then used to degrade the resulting 5′-monophosphate RNAs, leaving the capped mRNAs intact. The quality of the final mRNA samples was checked by agarose electrophoresis (1.2% in 1 × TBE, Supplementary Figure S4A, B) and dot-blot analysis using dsRNA-specific antibodies to confirm the absence of dsRNAs (Supplementary Figure S4C). The purified *G*Luc mRNAs were transfected into two different mammalian cell lines: A549 (human lung carcinoma epithelial cells) and JAWS II (mouse immortalized immature dendritic cells), which are examples of rapidly proliferating and metabolizing epithelial cells and immune cells sensitive to exogenous nucleic acids, respectively (68,69).

**Figure 5.**
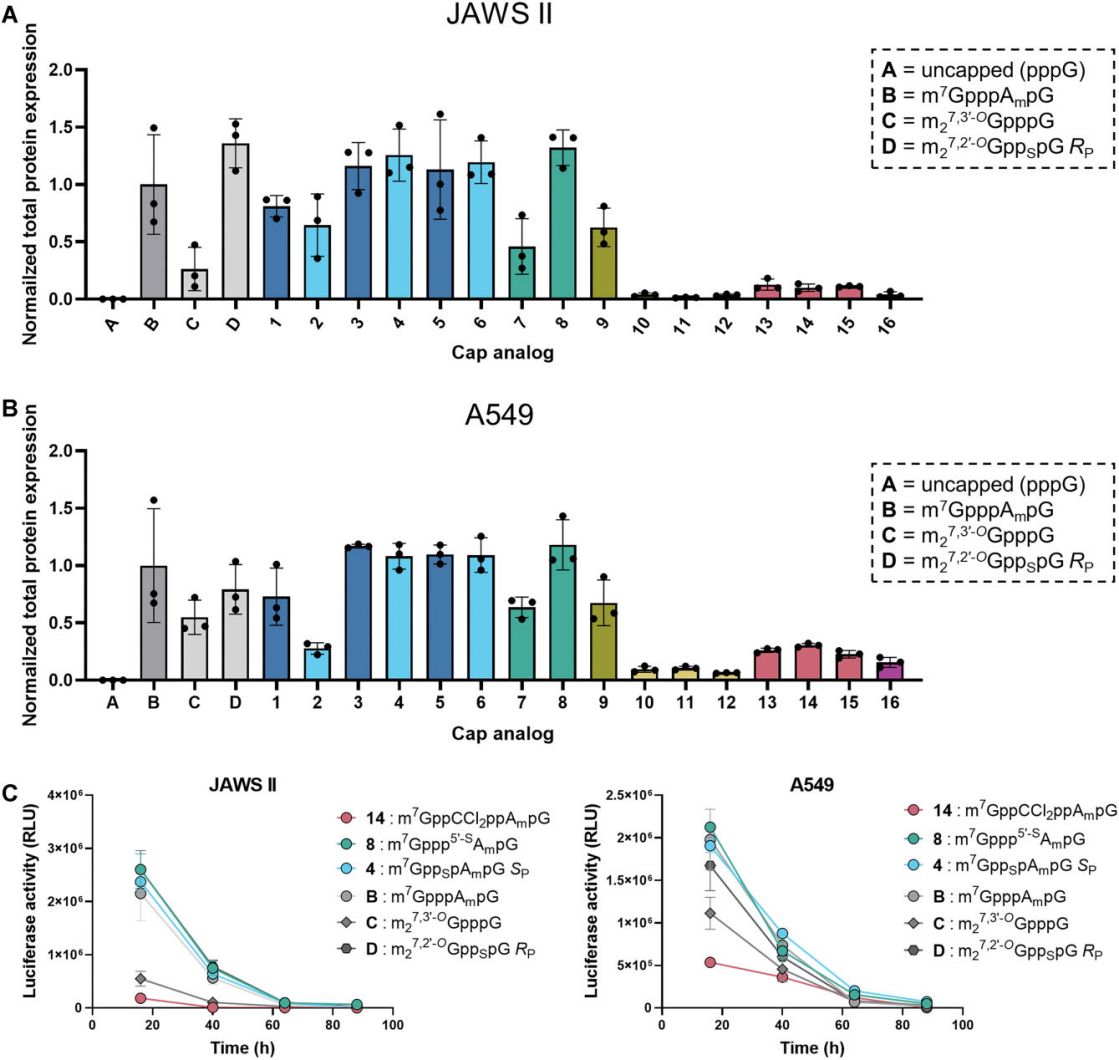
Protein expression yield in cultured cells for modified trinucleotide cap analogs. (**A**) Total protein expression over 88 h in JAWS II (cumulative luminescence). (**B**) Total protein expression over 88 h in A549. Bars represent mean value ± SD normalized to m^7^GpppA_m_pG. (**C**) Protein expression level in function of time of representative 2′-methylated mRNAs in JAWSII (left) and A549 (right) cell lines; displayed mRNAs were capped with m^7^Gpp_S_pA_m_pG *S*_P_ (4), m^7^Gppp^5’-S^A_m_pG (8) or m^7^GppCCl_2_ppA_m_pG (14), and compared to references (m^7^GpppA_m_pG B, m_2_^7,3′-^*^O^*GpppG C and m_2_^7,2′-^*^O^*Gpp_S_pG *R*_P_. For color coding see Figure 1 caption.

*G*Luc is a secretory reporter protein with a long half-life (∼6 days) that can be harvested from the cell culture medium for quantitative analysis without cell lysis (70), allowing mRNA translation to be monitored in living cells at multiple time points (16, 40, 64, 88 h). The protein levels for each mRNA in both cell lines normalized to m^7^GpppA_m_pG-RNA are shown in Figure 5 and Table 1. Consistent with previous results, the RNA capped with m^7^GpppA_m_pG (cap-1, B in Figure 5) produced approximately 4-fold more protein than the ARCA-capped RNA (C in Figure 5) in the JAWS II cell line (41), but only approximately 2-fold more in the A549 cell line. Also, the β-S-ARCA-capped RNA (D in Figure 5) was translated much more efficiently than ARCA-capped RNA in JAWS II, as observed previously in various mammalian cells (34,36,71), but the effect was much smaller in A549. For RNAs capped with β-phosphorothioate trinucleotides **3**–**6**, we observed only a small positive effect on their translation as compared to cap-1 RNA, while those capped with cap-0 structures (analogs **1** and **2**) provided significantly less protein in both cell lines. Similarly, the 5′-phosphorothiolate modification slightly increased translational activity when combined with cap-1 (analog **8**), but was not as beneficial in the context of the cap-0 structure (analog **7**). The α,β-methylenebisphosphonate modification reduced the translational activity to the level observed for mRNAs with caps-0, even though it was combined with the cap-1 methylation pattern (analog **9**). Surprisingly, all RNAs capped with tetraphosphate analogs (**10**–**16**) were very poorly translated in both cell lines, regardless of the adenosine methylation pattern. The O-to-CCl_2_ modification (analogs **13**–**15**) slightly increased the protein outputs, consistent with previous studies on dinucleotides (31), but it was still significantly lower than for all RNAs with triphosphate adenosine caps. We also observed that there were no significant differences in the temporal distribution of reporter protein level between differentially capped mRNAs that produced comparable amounts of protein (Figure 5C, Supplementary Figure S5).

### Pull-down assays with HEK protein extract

**Figure 6.**
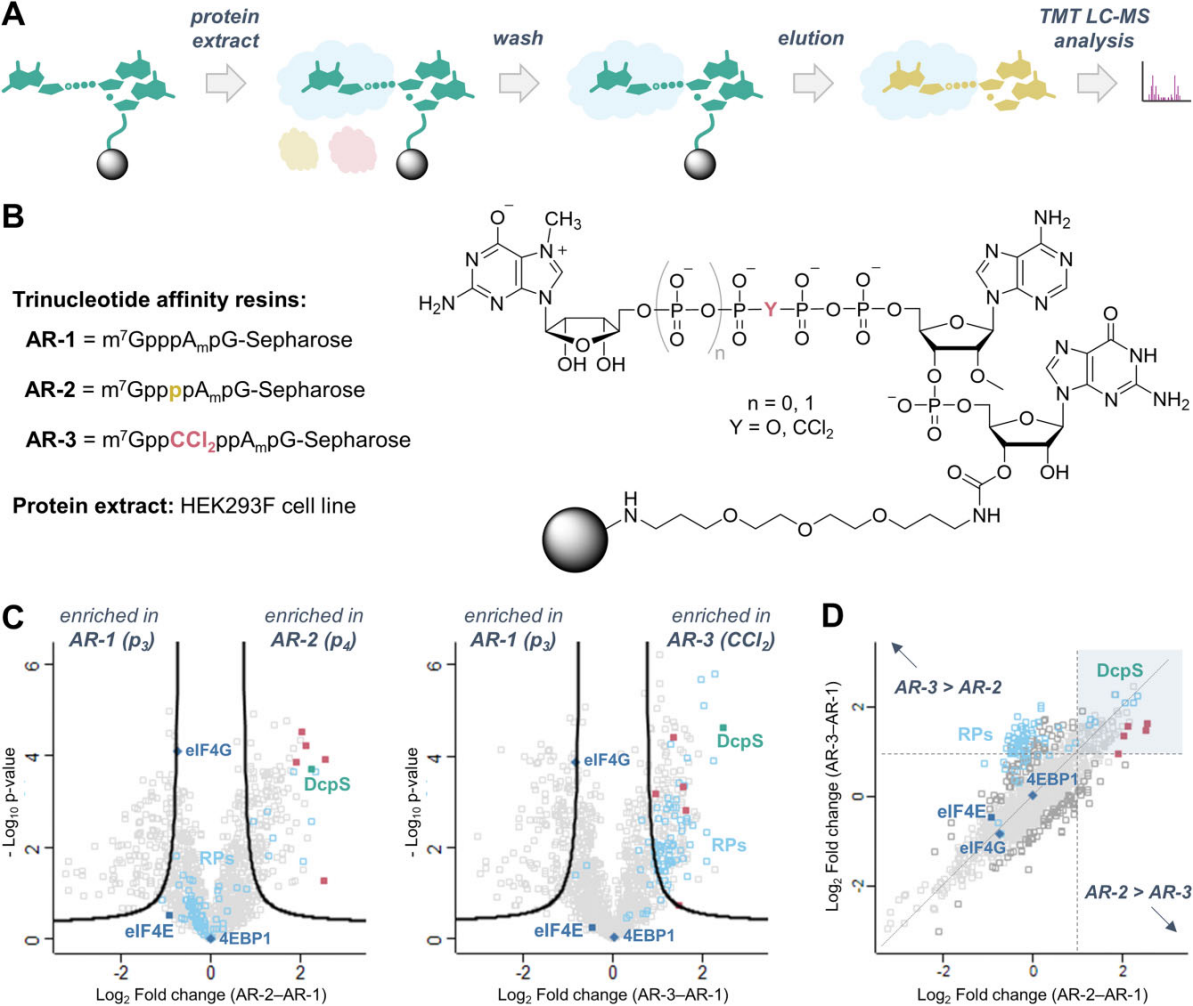
Pull-down assay with HEK293F cells protein extract. (**A**) General idea of the pull-down experiment using trinucleotide cap analogs immobilized on Sepharose beads. (**B**) Chemical structure of affinity resins used in the assay. (**C**) Volcano plots of the comparisons between protein levels detected in the elutes from trinucleotide cap affinity resins; each experiment was performed in three replicates; Student's T-test significant differences between the two samples indicated in the plot are colored dark grey; ribosomal proteins (RPs) are colored light blue; potential competitors of eIF4E protein (GNB2L1, TMA7, CMPK1, HSPA4, ARHGDIA) are colored pink; (**D**) Dot plot representations of correlation between Log_2_ Fold change **AR-3**/**AR-1** and Log_2_ Fold change **AR-2**/**AR-1**; the diagonal line represents proteins whose concentration was equal in **AR-2** and **AR-3** eluates.

The tetraphosphate capped mRNAs were found to be poorly translated despite their high affinities for eIF4E and low susceptibilities to hDcp2 *in vitro*, suggesting that other factor(s) may significantly contribute to their biological properties. To investigate the lack of correlation between the binding affinity of tetraphosphate cap analogs to eIF4E protein *in vitro* and the total protein expression of mRNAs capped with such analogs, we prepared affinity resins containing trinucleotide cap structures and performed pull-down assays of HEK293F protein extract (Figure 6A). To this end, trinucleotides **17**–**19**, which are the structural analogs of m^7^GpppA_m_pG, m^7^GppppA_m_pG (**11**) and m^7^GppCCl_2_ppA_m_pG (**14**), that are functionalized with a diamine linker at the ribose of the third nucleotide (guanosine), were synthesized and immobilized on BrCN-activated Sepharose beads (Supplementary Figure S6) (47). Thus prepared affinity resins **AR-1**, **AR-2** and **AR-3** (Figure 6B) were incubated with HEK293F protein extract in the presence of GTP to limit non-specific interactions. The pulled-down proteins were eluted with the corresponding trinucleotide cap analog (m^7^GpppA_m_pG for **AR-1**, compound **11** for **AR-2** and compound **14** for **AR-3**), digested with trypsin, labeled with isobaric tags (TMT), and analyzed by shotgun proteomics. The list of identified 1302 protein groups and their relative levels in the eluates are shown in the Supplementary Table. In summary, 91 protein groups were classified as preferential binders of **AR-2** over **AR-1** and 148 as preferential binders of **AR-3** over **AR-1**.

Interestingly, we found that eIF4E is not a preferential binder of **AR-2** and **AR-3** over **AR-1** (Figure 6C), which is in contrast to the results of the *in vitro* FQT assay. One of the possible explanations for this result is the competition for cap binding between eIF4E and other proteins. To verify this, we plotted Log_2_ fold changes of protein levels between **AR-3** and **AR-1** eluates against Log2 fold changes of protein levels between **AR-2** and **AR-1** eluates (Figure 6D) and focused on the proteins that are preferential binders of both tetraphosphate samples compared to the canonical triphosphate derivative **AR-1** (Figure 6D, blue shaded area). We found over 40 cytosolic proteins that were significantly enriched in both tetraphosphate resin eluates and narrowed them down to several hits (Figure 6C, pink squares), for which the concentrations in the samples were relatively high (top 25% as estimated by the iBAQ value; Supplementary Table) (72). These included Receptor of activated protein C kinase 1 (RACK1, GNB2L1), Translation machinery-associated protein 7 (TMA7), UMP-CMP kinase (CMPK1), Heat shock 70 kDa protein 4 (HSPA4), and Rho GDP-dissociation inhibitor 1 (ARHGDIA). Most of these proteins are known to bind nucleoside triphosphates, supporting their potential role in competing for interactions with tetraphosphate cap analogs.

Another possible explanation involves phosphorylation of eIF4E at the Ser209 residue, which has been shown to destabilize the eIF4E/m^7^GppppG complex more than the eIF4E/m^7^GpppG complex (73), but its effect on cap-dependent translation remains controversial (74). Phosphorylation of eIF4E is mainly regulated by Mnk1 (kinase) and PP2A (phosphatase) enzymes and we found the latter to be significantly enriched in samples eluted from tetraphosphate resins **AR-2** and **AR-3**. Given the fact that PP2A has a low isoelectric point, it is unlikely that it binds directly to the mRNA cap, but an indirect mechanism involving PP2A and the cap structure may be responsible for shifting the phosphorylation equilibrium of eIF4E.

Interestingly, we found that both **AR-2** and **AR-3** eluates are highly enriched in Decapping Scavenger enzyme (DcpS), which is a m^7^G cap-specific pyrophosphatase involved in the 3′-to-5′ mRNA degradation pathway (75). We speculate that this is a result of m^7^GpppA_m_pG-L13_N_ hydrolysis by DcpS on the resin, rather than the biologically relevant effect, since DcpS is known to be much more active towards dinucleotide cap structures than the capped RNAs (76).

### mRNA translation *in vivo*

**Figure 7.**
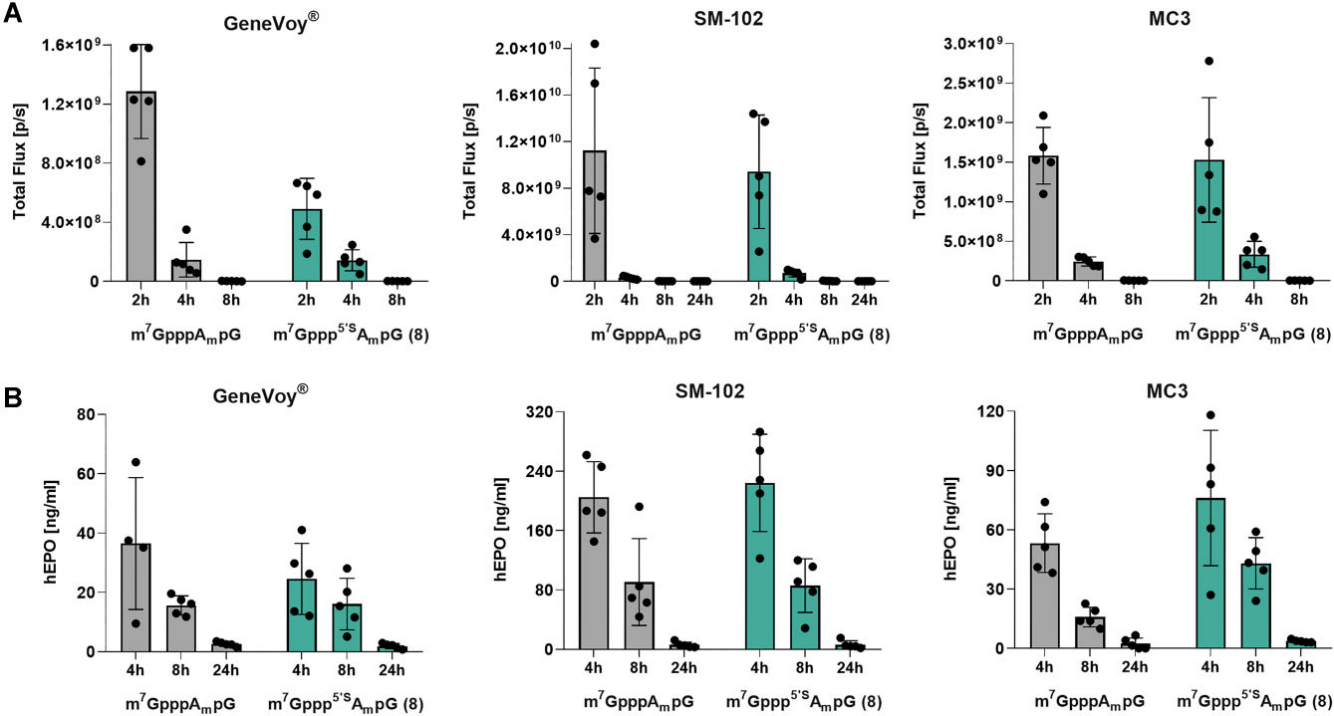
mRNAs capped with cap-1 and analog 8 yield similar protein production *in vivo*. (**A**) Intravital firefly luciferase (FLuc)-dependent bioluminescence in BALB/c mice injected intravenously (i.v.) with 10 μg of Fluc encoding mRNAs, capped with m^7^GpppA_m_pG or m^7^Gppp^5′S^A_m_pG, and formulated using different ionizable lipids (GenVoy-ILM^TM^, SM-102 and MC3). Total flux [p/s] mean values ± SD, *n* = 4–5. (**B**) Human erythropoietin (hEPO) serum concentrations in C57BL/6 mice injected i.v. with 1 μg of hEPO encoding mRNAs, capped with m^7^GpppA_m_pG or m^7^Gppp^5′S^A_m_pG, and formulated using different ionizable lipids (GenVoy-ILM™, SM-102 and MC3). Data show mean values ± SD, *n* = 4–5.

The cell culture studies revealed several promising candidates for improving the translational properties of mRNAs, including β-phosphorothioate analogs **3**–**6** and 5′-phosphorothiolate (α-PSL) analog **8**. The synthesis of β-S analogs **3**–**6** requires HPLC separation of the diastereomeric mixtures, which is problematic on a larger scale, therefore we focused our attention on analog **8** (m^7^Gppp^5’S^A_m_pG), which does not contain an additional stereogenic center, and further characterized it in *in vivo* mouse models. To this end, we used two reporter genes, namely wild-type firefly luciferase and human erythropoietin, and prepared appropriate mRNAs capped with m^7^GpppA_m_pG or analog **8**, confirming their purity and integrity before each experiment (Supplementary Figure S7). Then, mRNAs were formulated into LNPs using various ionizable lipids (GeneVoy^®^, SM-102 or MC3, Supplementary Table S5), and administered intravenously into mice. Whole body bioluminescence (as an indirect measure of firefly luciferase protein enzymatic activity) or serum hEPO concentrations were determined at several time points (Figure 7). For both reporter genes, the SM-102 formulated mRNAs provided the highest protein expression, but no significant differences were observed between the cap analogs. Interestingly, for hEPO-encoding mRNAs formulated in MC3 LNPs, we observed a slight increase in protein expression upon 5′-phosphorothiolate modification of the cap, whereas for firefly luciferase mRNAs formulated using GeneVoy^®^, the effect was the opposite.

## Discussion

Chemical modification of mRNAs has emerged as a promising strategy to improve their pharmacological properties, such as cellular stability, reactogenicity or translational activity. One of the structural elements of mRNA that is of particular interest in this context is the 5′ cap, which plays a key role in regulating the translation process. Over the past two decades, several modifications of the cap structure have been found to be beneficial for mRNA stability and translational activity. Here, we re-evaluated some of the most promising examples in the context of trinucleotide cap structures that provide the highest capping efficiency during the *in vitro* transcription reaction and allow for incorporation of the natural methylation patterns found in cap-1 and m^6^A_m_ cap. To this end, a series of 16 m^7^GpppApG analogs with a chemically modified 5′,5′-oligophosphate bridge were synthesized and characterized as *in vitro* transcription capping reagents. We found that the oligophosphate bridge modifications do not affect the capping efficiency during *in vitro* transcription with T7 RNA polymerase on DNA template containing the standard Φ6.5 promoter sequence. For A and A_m_ cap analogs, approximately 90% of IVT RNA molecules were capped, while for ^m6^A_m_ cap analogs the capping efficiency varied from 60 to 75%.

In general, the biological effects of oligophosphate modifications introduced with trinucleotide analogs were similar to those introduced with ARCAs (i.e. m_2_^7,2′-^*^O^*GpppG analogs), but in some cases the magnitude of these effects was less pronounced. The dissociation constants between translation initiation factor 4E (eIF4E) and trinucleotide caps **1**–**16** followed the same pattern as previously observed for dinucleotides: the tetraphosphates provided the highest stabilizing effect, both diastereomers of β-phosphorothioates also stabilized the complex with a slight preference for the *R*_P_ isomers, while the 5′-phosphorothiolate modification was virtually neutral. It is notable that the cap-1 modification appears to attenuate the correlation between translation efficiency and the affinity of the cap for eIF4E, a phenomenon that has been previously observed in numerous studies (27,29). The case of tetraphosphate cap analogs (as well as *N*6-benzyl analog (77)) suggests that this may be related to the specificity of cap interactions in a more complex environment, which is an area that requires further investigation.

The effect of phosphate modifications was also preserved in the context of RNA decapping by Dpc2 enzyme. Tetraphosphates (**10**–**12**), 5′-phosphorothiolates (**7**–**8**) and *R*_P_-β-phosphorothioates (**1**, **3** and **5**) were hydrolyzed slightly slower than the unmodified caps, *S*_P_-β-phosphorothioates (**2**, **4** and **6**) and β,γ-methylenebisphosphonates (**16**) were even less susceptible, while β,γ-dichloromethylenebisphosphonates (**13**–**15**) were completely resistant to Dcp2. Surprisingly, the combination of decapping resistance with ribose methylation of the first transcribed nucleotide (cap-1) did not provide the same beneficial impact on translational activity as observed in previous studies on cap-0 structures, particularly β-S-ARCA, which has been used in several clinical trials of therapeutic mRNA to date. We observed a similar effect in our latest results on mRNAs carrying an *N*6-benzylated adenosine cap analog, which produced significantly more protein (in comparison to cap-1-mRNAs), even though it was more susceptible to decapping by Dcp2 (77). This raises the question of whether decapping resistance is a crucial factor in the therapeutic efficacy of mRNAs capped with cap-1 structures, in contrast to what has been previously thought.

Despite the increased affinity for eIF4E and reduced susceptibility to decapping, we were unable to reproduce the up to 1.5-fold increase in translation efficiency of mRNAs containing β-phosphorothioate or 5′-phosphorothiolate modifications observed in HeLa cells for ARCA-type caps. Nevertheless, the mRNAs with analogs **3**, **4** and **8** still provided 1.1–1.3-fold higher protein levels in both JAWS II and A549 than the corresponding mRNA capped with unmodified cap-1 (m^7^GpppA_m_pG), which makes them promising candidates for further evaluation in different cell lines and *in vivo* models. The discrepancies in biological properties between ARCA-capped and the corresponding m^7^GpppA_m_pG-capped RNAs may result from the differences in 5′-terminal nucleobase identity and methylation pattern (cap-0 versus cap-1), but also from the method used to isolate the mRNAs, which determines their purity and integrity. In this study, we paid particular attention to the latter aspect by removing the uncapped (pppG-) RNA and including an HPLC purification step to reduce the level of dsRNA contaminants. Taken together, our study demonstrates that phosphate modifications of the cap structure can be combined with a trinucleotide capping approach to provide efficient capping reagents suitable for the production of high-quality mRNAs or for modulation of their biological properties in functional studies on e.g. mRNA decapping. The basic insights into their biological properties provided some important clues for designing new molecular tools for the investigation of mRNA metabolism as well as for optimization of therapeutic mRNAs.

## Supplementary Material

gkae763_Supplemental_File

## Data Availability

The raw LC-MS/MS data and the output from MaxQuant have been deposited to the ProteomeXchange Consortium (57) via the PRIDE (58) partner repository with the dataset identifier PXD053198.
