# Supplementary material for: Trinucleotide cap analogs with triphosphate chain modifications: synthesis, properties, and evaluation as mRNA capping reagents
Source: Nucleic Acids Res. 2024 Sep 9;52(18):10788–809. doi: 10.1093/nar/gkae763 (PMC11472058; doi:10.1093/nar/gkae763)

Trinucleotide cap analogs with triphosphate chain modifications: synthesis, properties, and evaluation as mRNA capping reagents

Marcin Warminski[1,†], Anais Depaix[2,†], Kamil Ziemkiewicz[2], Tomasz Spiewla[1,3], Joanna Zuberek[1], Karolina Drazkowska[2], Hanna Kedzierska[3], Agnieszka Popielec[3], Marek R. Baranowski[3], Marta Sklucka[3], Marcelina Bednarczyk,[3] Miroslaw Smietanski[3], Karol Wolosewicz,[3] Bartosz Majewski,[3] Remigiusz A. Serwa[4], Dominika Nowis[3,5], Jakub Golab[3,6], Joanna Kowalska[1,3*], and Jacek Jemielity[2,3*]

[1] Division of Biophysics, Institute of Experimental Physics, Faculty of Physics, University of Warsaw, Pasteura 5, 02-093 Warsaw, Poland
[2] Centre of New Technologies, University of Warsaw, Banacha 2C, 02-097 Warsaw, Poland,
[3] Explorna Therapeutics sp. z o.o, Zwirki i Wigury 93/2157, 02-089, Warsaw, Poland,
[4] Proteomics Core Facility, IMol Polish Academy of Sciences, 02-247 Warsaw, Poland
[5] Laboratory of Experimental Medicine, Faculty of Medicine, Medical University of Warsaw, Nielubowicza 5, 02-097 Warsaw, Poland,
[6] Department of Immunology, Medical University of Warsaw, Nielubowicza 5, 02-097 Warsaw, Poland

Table S1. List of the cap analogs used in this study.

No	Name	Chemical structure
1	**m^7Gpp_SpApG D1**	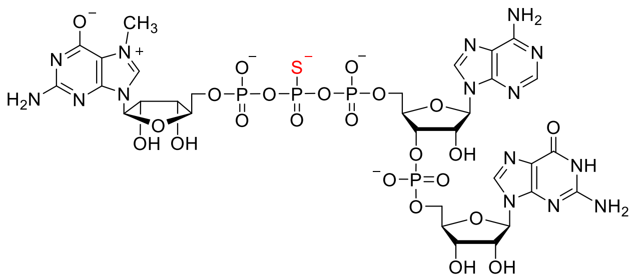
2	**m^7Gpp_SpApG D2**	
3	**$m^7Gpp_SpA_mpG$ D1**	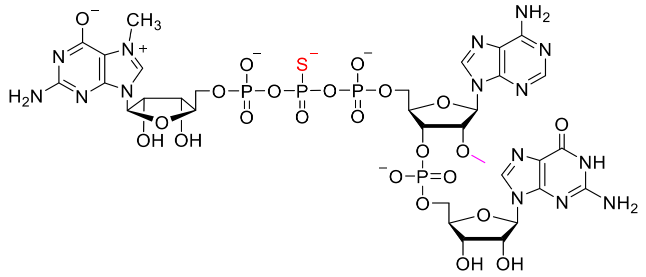
4	**$m^7Gpp_SpA_mpG$ D2**	
5	**$m^7Gpp_Sp^{m6}A_mpG$ D1**	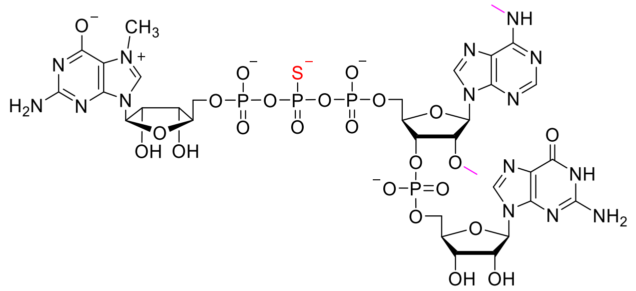
6	**$m^7Gpp_Sp^{m6}A_mpG$ D2**	
7	**$m^7Gppp^{5'S}ApG$**	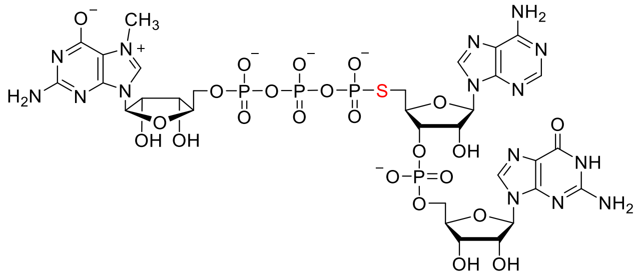
8	**$m^7Gppp^{5'S}A_mpG$**	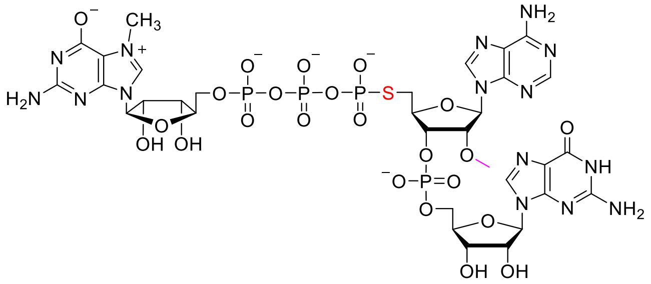
9	**$m^7GppCH_2pA_mpG$**	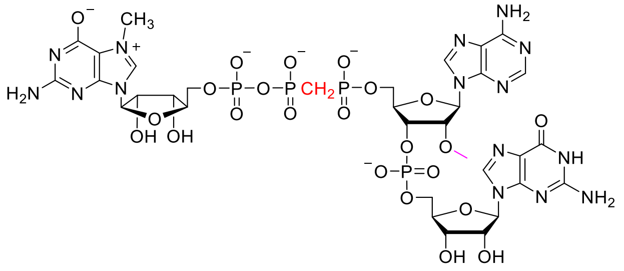

**10**	**m^{7}GppppApG**	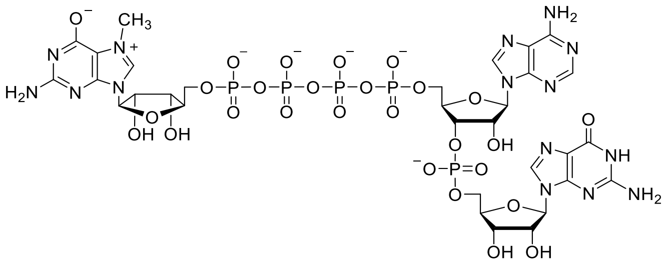
**11**	**m^{7}GppppA$_m$pG**	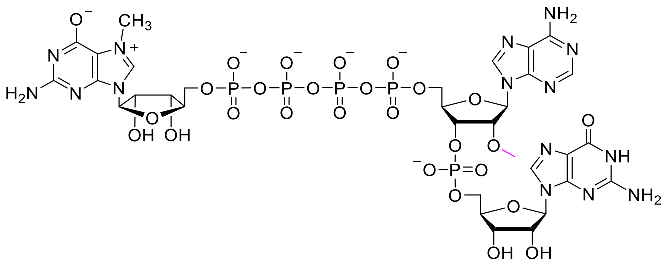
**12**	**m^{7}Gpppp^{m6}A$_m$pG**	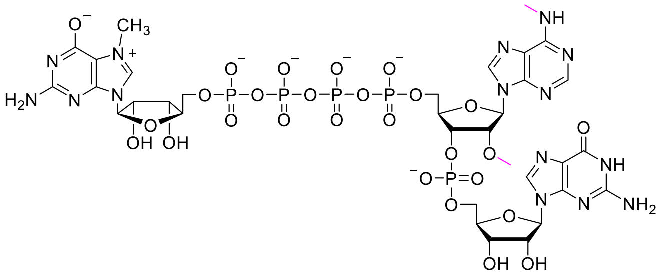
**13**	**m^{7}GppCCl$_2$ppApG**	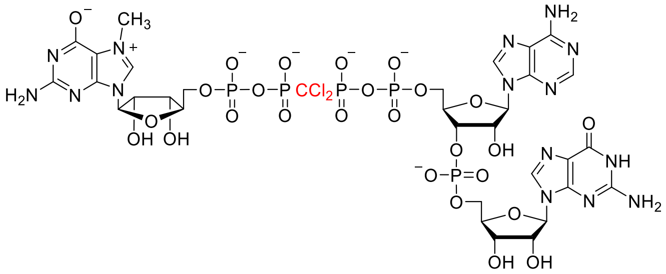
**14**	**m^{7}GppCCl$_2$ppA$_m$pG**	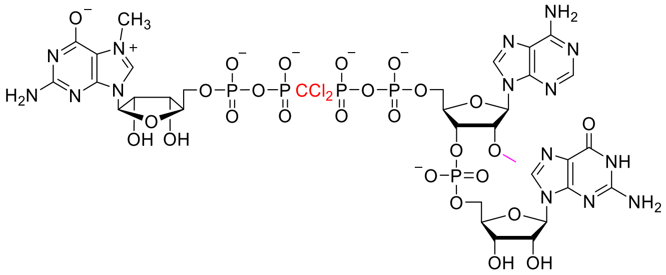
**15**	**m^{7}GppCCl$_2$pp^{m6}A$_m$pG**	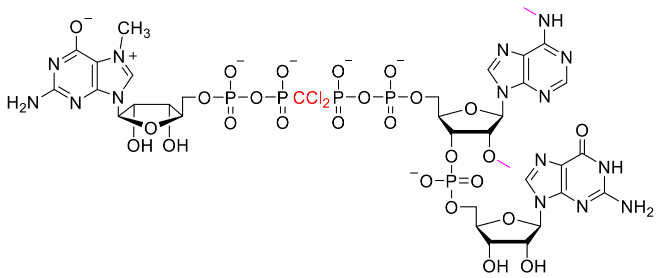
**16**	**m^{7}GppCH$_2$ppA$_m$pG**	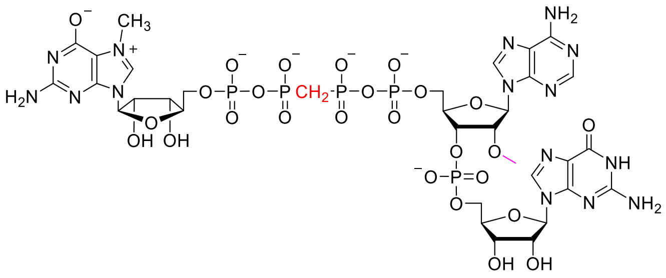

Table S2. DNA sequence for Firefly luciferase (FLuc).

Name	DNA Sequence
Firefly lucyferase (FLuc)	GGATAATCTAGACATTTGCTTCTGACACAACTGTGTTCACTAGCAACCTCAAAC<br>AGACACCATGGAAGACGCCAAAAACATAAAGAAAGGCCCGGCGCCATTCTAT<br>CCTCTAGAGGATGGAACCGCTGGAGAGCAACTGCATAAGGCTATGAAGAGAT<br>ACGCCCTGGTTCCTGGAACAATTGCTTTTACAGATGCACATATCGAGGTGAAC<br>ATCACGTACGCGGAATACTTCGAAATGTCCGTTCGGTTGGCAGAAGCTATGAA<br>ACGATATGGGCTGAATACAAATCACAGAATCGTCGTATGCAGTGAAAACTCTC<br>TTCAATTCTTTATGCCGGTGTTGGGCGCGTTATTTATCGGAGTTGCAGTTGCG<br>CCCGCGAACGACATTTATAATGAACGTGAATTGCTCAACAGTATGAACATTTCG<br>CAGCCTACCGTAGTGTTTGTTTCCAAAAAGGGGTTGCAAAAAATTTTGAACGT<br>GCAAAAAAAATTACCAATAATCCAGAAAATTATTATCATGGATTCTAAAACGGAT<br>TACCAGGGATTTCAGTCGATGTACACGTTCGTCACATCTCATCTACCTCCCGG<br>TTTTAATGAATACGATTTTGTACCAGAGTCCTTTGATCGTGACAAAACAATTGC<br>ACTGATAATGAATTCCTCTGGATCTACTGGGTTACCTAAGGGTGTGGCCCTTC<br>CGCATAGAACTGCCTGCGTCAGATTCTCGCATGCCAGAGATCCTATTTTTGGC<br>AATCAAATCATTCCGGATACTGCGATTTTAAGTGTTGTTCCATTCCATCACGGT<br>TTTGGAATGTTTACTACACTCGGATATTTGATATGTGGATTTCGAGTCGTCTTA<br>ATGTATAGATTTGAAGAAGAGCTGTTTTTACGATCCCTTCAGGATTACAAAATT<br>CAAAGTGCGTTGCTAGTACCAACCCTATTTTCATTCTTCGCCAAAAGCACTCTG<br>ATTGACAAATACGATTTATCTAATTTACACGAAATTGCTTCTGGGGGCGCACCT<br>CTTTCGAAAGAAGTCGGGGAAGCGGTTGCAAAACGCTTCCATCTTCCAGGGAT<br>ACGACAAGGATATGGGCTCACTGAGACTACATCAGCTATTCTGATTACACCCG<br>AGGGGGATGATAAACCGGGCGCGGTCGGTAAAGTTGTTCCATTTTTTGAAGC<br>GAAGGTTGTGGATCTGGATACCGGGAAAACGCTGGGCGTTAATCAGAGAGGC<br>GAATTATGTGTCAGAGGACCTATGATTATGTCCGGTTATGTAAACAATCCGGAA<br>GCGACCAACGCCTTGATTGACAAGGATGGATGGCTACATTCTGGAGACATAG<br>CTTACTGGGACGAAGACGAACACTTCTTCATAGTTGACCGCTTGAAGTCTTTAA<br>TTAAATACAAAGGATATCAGGTGGCCCCCGCTGAATTGGAATCGATATTGTTA<br>CAACACCCCAACATCTTCGACGCGGGCGTGGCAGGTCTTCCCGACGATGACG<br>CCGGTGAACTTCCCGCCGCCGTTGTTGTTTTGGAGCACGGAAAGACGATGAC<br>GGAAAAAGAGATCGTGGATTACGTCGCCAGTCAAGTAACAACCGCGAAAAAGT<br>TGCGCGGAGGAGTTGTGTTTGTGGACGAAGTACCGAAAGGTCTTACCGGAAA<br>ACTCGACGCAAGAAAAATCAGAGAGATCCTCATAAAGGCCAAGAAGGGCGGA<br>AAGTCCAAATTGTAAGGATCCTAGGGCCGCAGCTCGCTTTCTTGCTGTCCAAT<br>TTCTATTAAAGGTTCCTTTGTTCCCTAAGTCCAACTACTAAACTGGGGGATATT<br>ATGAAGGGCCTTGAGCATTTGGATTCTGCCTAATAAAAAACATTTATTTTCATT<br>GCGTTTAGCTCGCTTTCTTGCTGTCCAATTTCTATTAAAGGTTCCTTTGTTCCC<br>TAAGTCCAACTACTAAACTGGGGGATATTATGAAGGGCCTTGAGCATTTGGAT<br>TCTGCCTAATAAAAAACATTTATTTTCATTGCATTTAAATGTTTAAACAAAAAAAA<br>AAAAAAAAAAAAAAAAAAAAAAAAAAAAAAAAAAAAAAAAAAAAAAAAAAAAAAA<br>AAAAAAAAAAAAAAAAAAAAAAAAAAA

Table S3. Summary of the synthesis scales, yields, HPLC, and HRMS data for synthesized dinucleotides.

No	Abbreviation	Synthesis scale [μmol] [a]	Yield [μmol]	RP-HPLC R_t [min] [b]	m/z calcd.	m/z found
**20**	pApG	25	19.6	8.680*	691.10324	691.10392
**21**	pA$_m$pG	4×50	141.7	12.070*	705.11889	705.11981
**22**	p^{m6}A$_m$pG	2×50	60.7	14.434*	719.13454	719.13537
**26**	p$^{5'S}$ApG	50	15.9	6.497**	707.08040	707.08101
**27**	p$^{5'S}$A$_m$pG	50	10.5	11.398*	721.09605	721.09686
**28**	pCH$_2$pA$_m$pG	50	12.7	9.728*	783.10596	783.10695

[a] calculated as a product of solid support weight and loading; [b] conditions A (*) or conditions B (**) – see *General information*;

Table S4. Summary of the synthesis scales, yields, HPLC and HRMS data for trinucleotide cap analogs 1–19.

No	Abbreviation	Synthesis scale [μmol] [a]	Yield [μmol] [b]	RP-HPLC R_t [min] [c]	m/z calcd.	m/z found
**1**<br>**2**	m^{7}Gpp$_S$pApG	4.65	*R*$_P$: 0.81<br>*S*$_P$: 1.17	8.705*<br>9.045*	1146.10981	1146.11096<br>1146.11147
**3**<br>**4**	m^{7}Gpp$_S$pA$_m$pG	33.0	*R*$_P$: 6.50<br>*S*$_P$: 6.16	10.493*<br>10.713*	1160.12546	1160.12689<br>1160.12696
**5**<br>**6**	m^{7}Gpp$_S$p^{m6}A$_m$pG	30.4	*R*$_P$: 7.06<br>*S*$_P$: 5.94	12.225*<br>12.372*	1174.14111	1174.14244<br>1174.14250
**7**	m^{7}Gppp$^{5'S}$ApG	15.9	1.22	6.393**	1146.10981	1146.11080
**8**	m^{7}Gppp$^{5'S}$A$_m$pG	10.5	1.17	7.353**	1160.12546	1160.12658
**9**	m^{7}GppCH$_2$pA$_m$pG	12.7	10.8	9.973*	1142.16904	1142.17023
**10**	m^{7}GppppApG	19.0	2.65	5.089*	1210.09899	1210.10009
**11**	m^{7}GppppA$_m$pG	58.1	17.0	6.356*	1224.11464	1224.11521
**12**	m^{7}Gpppp^{m6}A$_m$pG	50.0	25.8	7.392*	1238.13029	1238.13119
**13**	m^{7}GppCCl$_2$ppApG	20.4	4.75	5.438*	1276.04178	1276.04367
**14**	m^{7}GppCCl$_2$ppA$_m$pG	34.8	9.19	6.553*	1290.05743	1290.05776
**15**	m^{7}GppCCl$_2$pp^{m6}A$_m$pG	58.9	18.2	7.547*	1304.07308	1304.07353
**16**	m^{7}GppCH$_2$ppA$_m$pG	50.9	27.9	6.481*	1222.13537	1222.13583
**17**	m^{7}GpppA$_m$pG-L13$_N$	13.5	4.75	*I1:* 10.842*<br>*I2:* 11.845*	1390.30626	1390.30808<br>1390.30788
**18**	m^{7}GppppA$_m$pG-L13$_N$	17.8	10.0	*I1:* 6.528**<br>*I2:* 7.000**	1470.27259	1470.27372<br>1470.27377
**19**	m^{7}GppCCl$_2$pA$_m$pG-L13$_N$	17.8	2.81	*I1:* 6.375**<br>*I2:* 6.778**	1536.21539	1536.21595<br>1536.21573

[a] based on the amount of pNpG used for the synthesis; [b] after RP-HPLC; [c] conditions A (*) or conditions B (**) – see *General information*;

Table S5. Physicochemical properties of LNPs used in the in vivo experiments.

Formulation	RNA ID	N/P	EE (%)	Size (nm)	PDI	Conditions
**GenVoy**	FLUC1	6	93	89	0.09 ± 0.01	PBS, 25°C
**GenVoy**	FLUC2	6	94	112 ± 1	0.11	PBS, 25°C
**Moderna (SM-102)**	FLUC1	6	89	84	0.03 ± 0.03	PBS, 25°C
**Moderna (SM-102)**	FLUC2	6	89	86	0.06 ± 0.01	PBS, 25°C
**Onpattro (MC3)**	FLUC1	6	95	82	0.07	PBS, 25°C
**Onpattro (MC3)**	FLUC2	6	97	74 ± 1	0.05 ± 0.03	PBS, 25°C
**GenVoy**	EPO1	6	92	89 ± 1	0.08 ± 0.01	PBS, 25°C
**GenVoy**	EPO2	6	92	104 ± 1	0.10 ± 0.01	PBS, 25°C
**Moderna (SM-102)**	EPO1	6	90	76	0.06 ± 0.04	PBS, 25°C
**Moderna (SM-102)**	EPO2	6	92	83 ± 1	0.06 ± 0.02	PBS, 25°C
**Onpattro (MC3)**	EPO1	6	94	72	0.08 ± 0.01	PBS, 25°C
**Onpattro (MC3)**	EPO2	6	96	62	0.09	PBS, 25°C

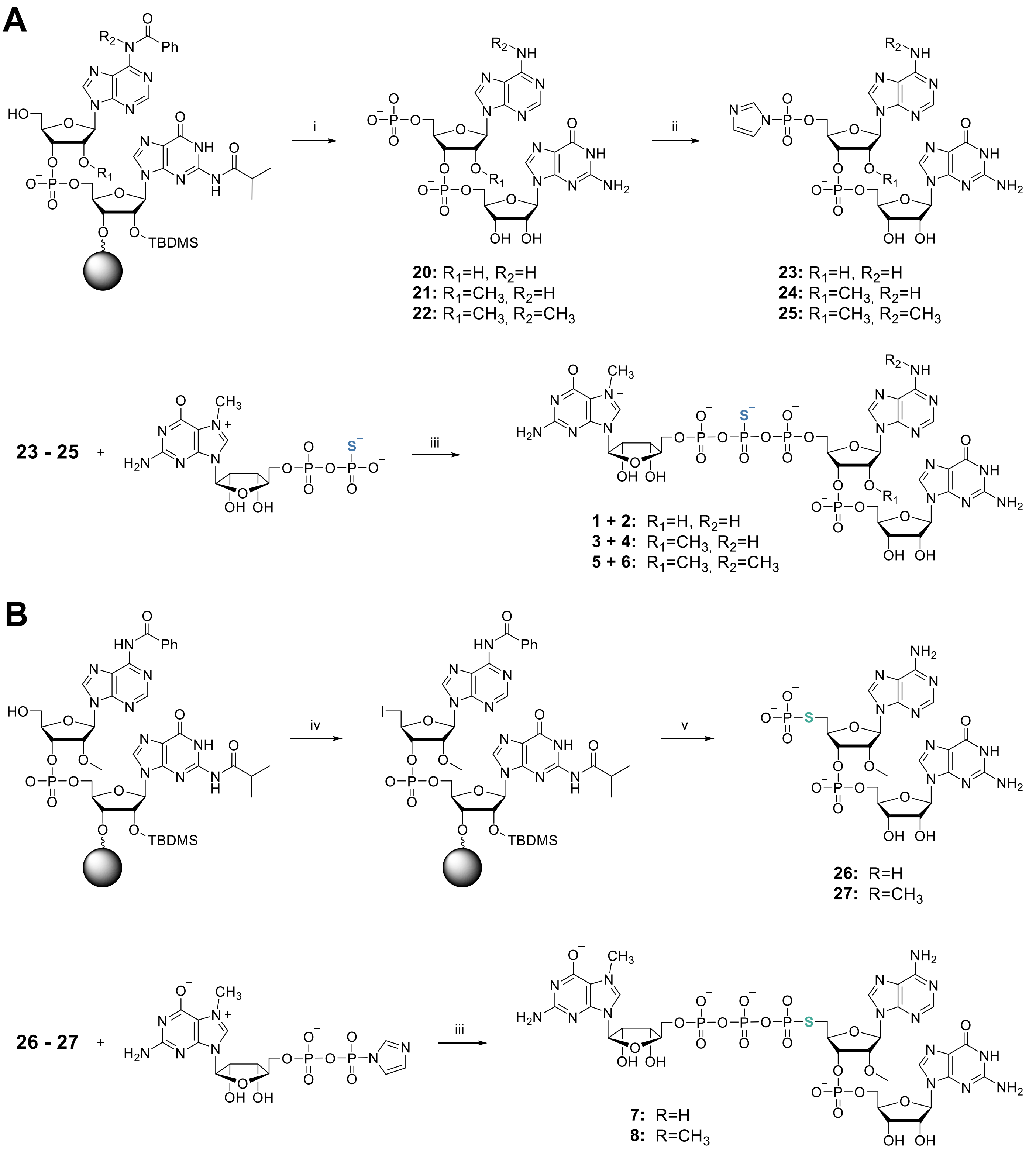

Figure S1. Synthesis of cap analogs 1–16. Reaction conditions: i. 1) bis-cyanoethylphosphoramidite, benzylthiotetrazole, acetonitrile; 2) I_2, pyridine/water; 3) diethylamine, acetonitrile; 4) AMA, 37 °C, 3 h; 5) TEA·3HF, DMSO, 65 °C, 2 h; ii. imidazole, 2,2′-ditiodipiridine, triphenylphosphine, triethylamine, DMF; iii. $ZnCl_2$, DMF; iv. triphenoxymethylphoshonium iodide, DMF; v. 1) triethylammonium thiophosphate, DMF; 2) AMA, 37 °C, 3 h; 3) TEA·3HF, DMSO, 65 °C, 2 h;

C

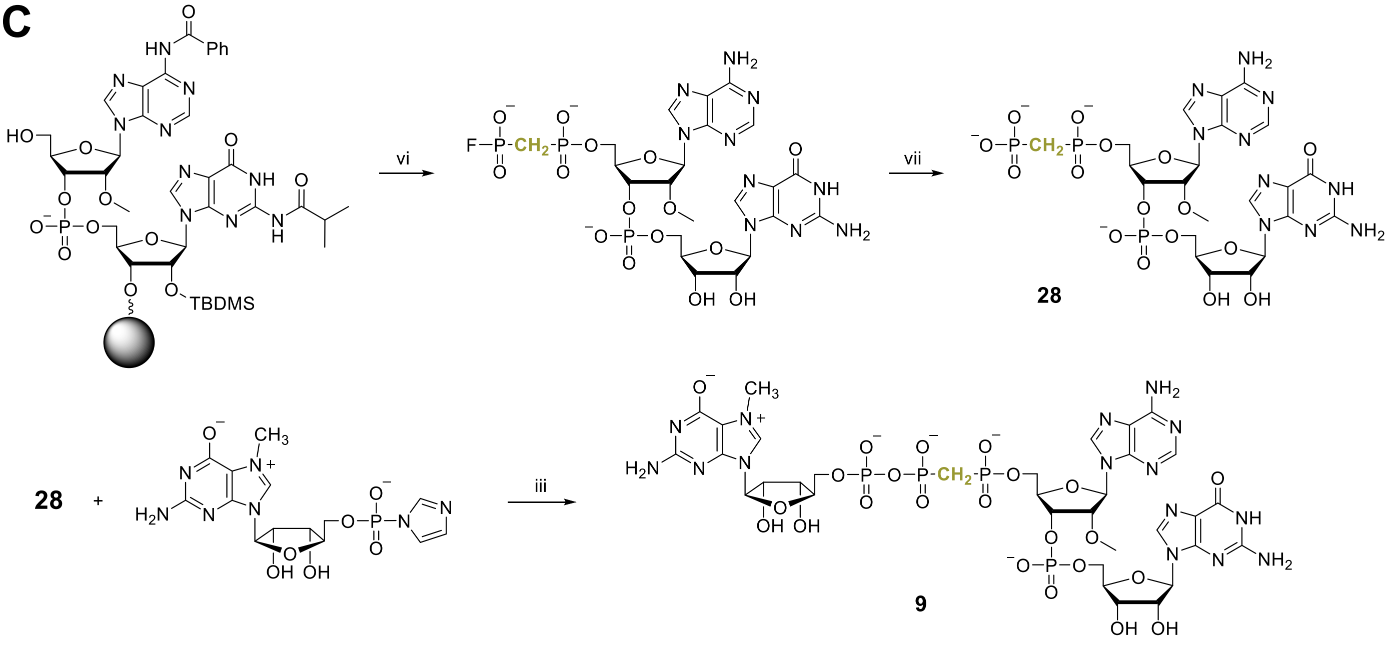

D

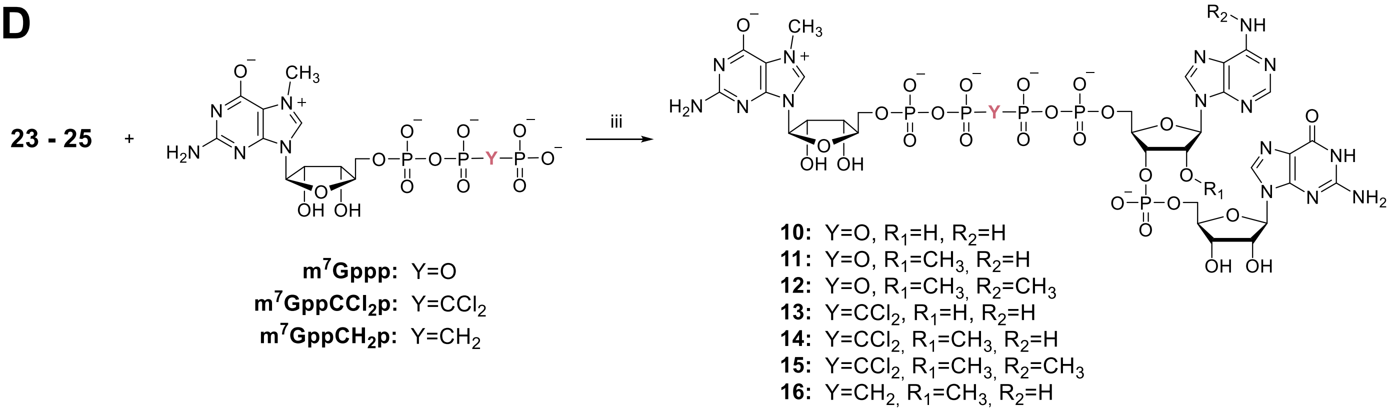

Figure S1 cont. Synthesis of cap analogs 1–16. Reaction conditions: vi. 1) methylenebis(phosphonic dichloride), trimethyl phosphate, 2°C, 7 h, 2) AMA, 37 °C, 3 h; 3) TEA·3HF, DMSO, 65 °C, 2 h; vii. HCl/H_2O, pH=1, 7 days;

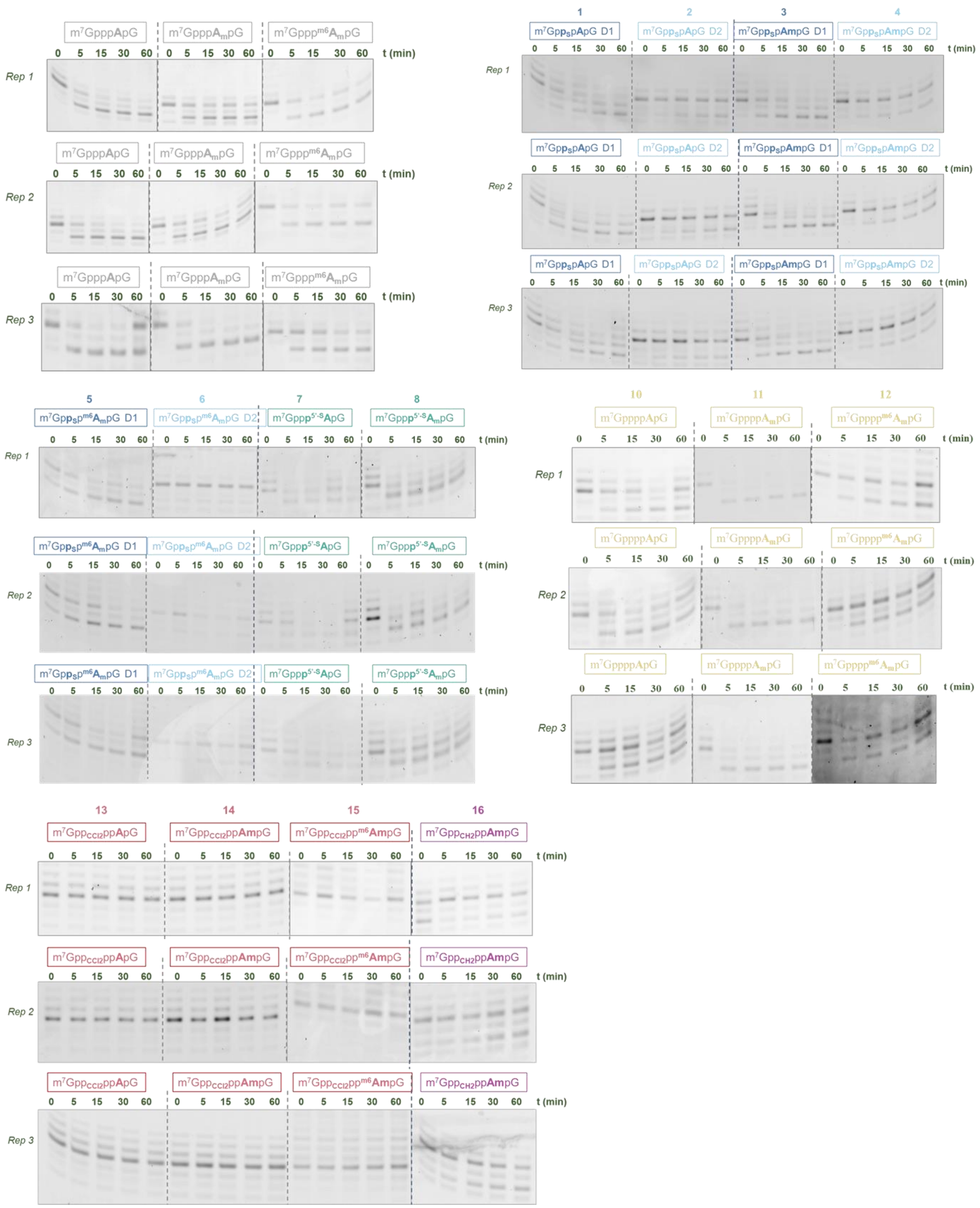

Figure S2. PAGE analysis of RNA decapping.

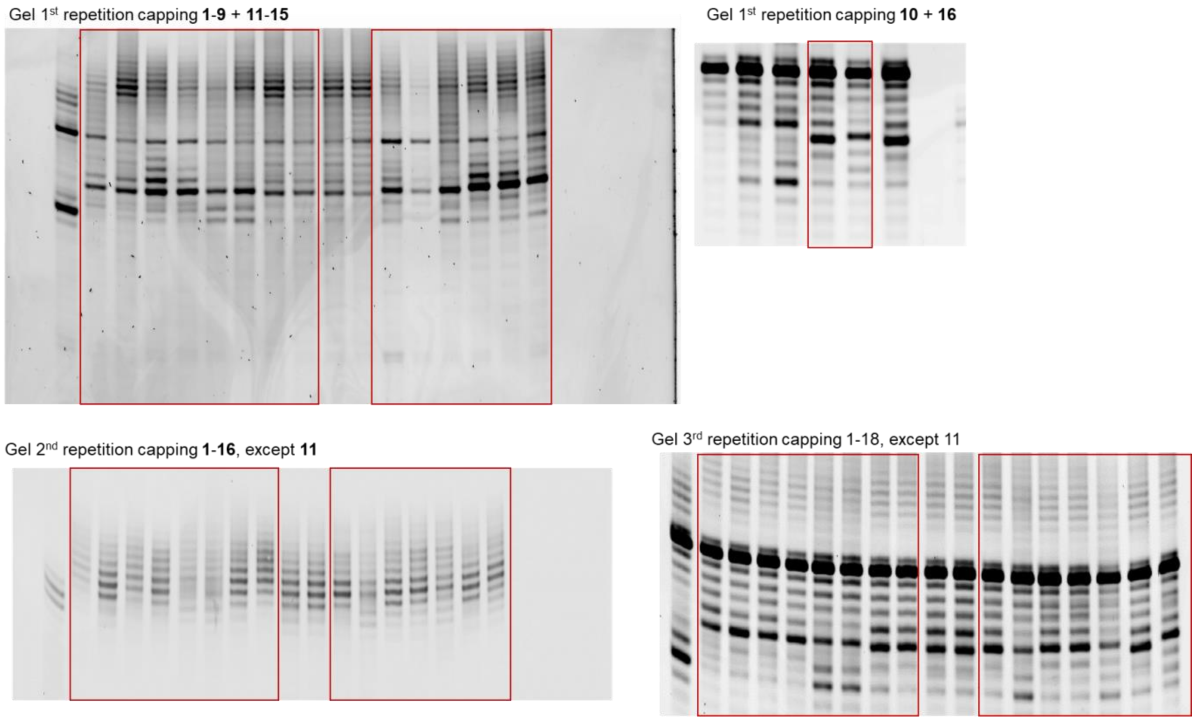

Figure S3. Raw data (3 replicates) for PAGE analysis of capping efficiency.

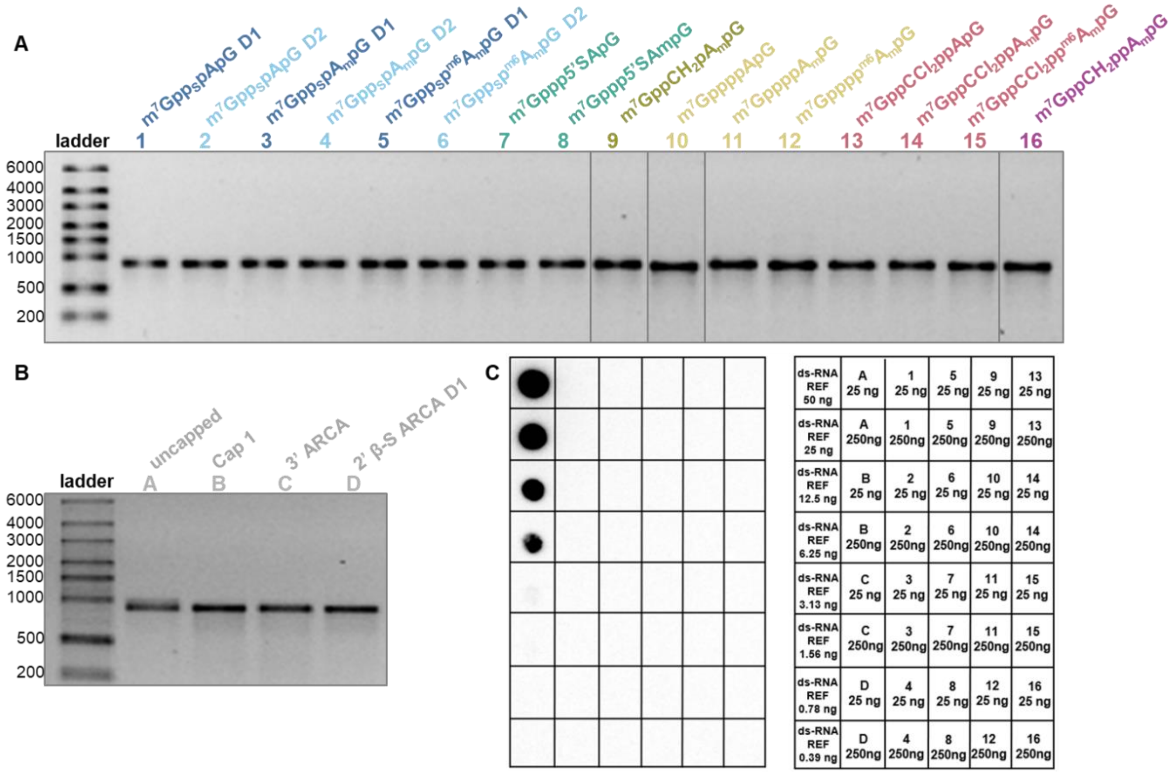

ds-RNA REF 50 ng	A 25 ng	1 25 ng	5 25 ng	9 25 ng	13 25 ng
ds-RNA REF 25 ng	A 250ng	1 250ng	5 250ng	9 250ng	13 250ng
ds-RNA REF 12.5 ng	B 25 ng	2 25 ng	6 25 ng	10 25 ng	14 25 ng
ds-RNA REF 6.25 ng	B 250ng	2 250ng	6 250ng	10 250ng	14 250ng
ds-RNA REF 3.13 ng	C 25 ng	3 25 ng	7 25 ng	11 25 ng	15 25 ng
ds-RNA REF 1.56 ng	C 250ng	3 250ng	7 250ng	11 250ng	15 250ng
ds-RNA REF 0.78 ng	D 25 ng	4 25 ng	8 25 ng	12 25 ng	16 25 ng
ds-RNA REF 0.39 ng	D 250ng	4 250ng	8 250ng	12 250ng	16 250ng

Figure S4. mRNA quality control. Agarose gel (1.2% in 1×TBE) of capped mRNAs (**A**) and reference mRNAs (**B**) used for translation experiments. The lanes in gel **A** have been re-ordered according to the analogs numbering. **C)** Dot-blot analysis of final mRNAs using dsRNA specific antibodies.

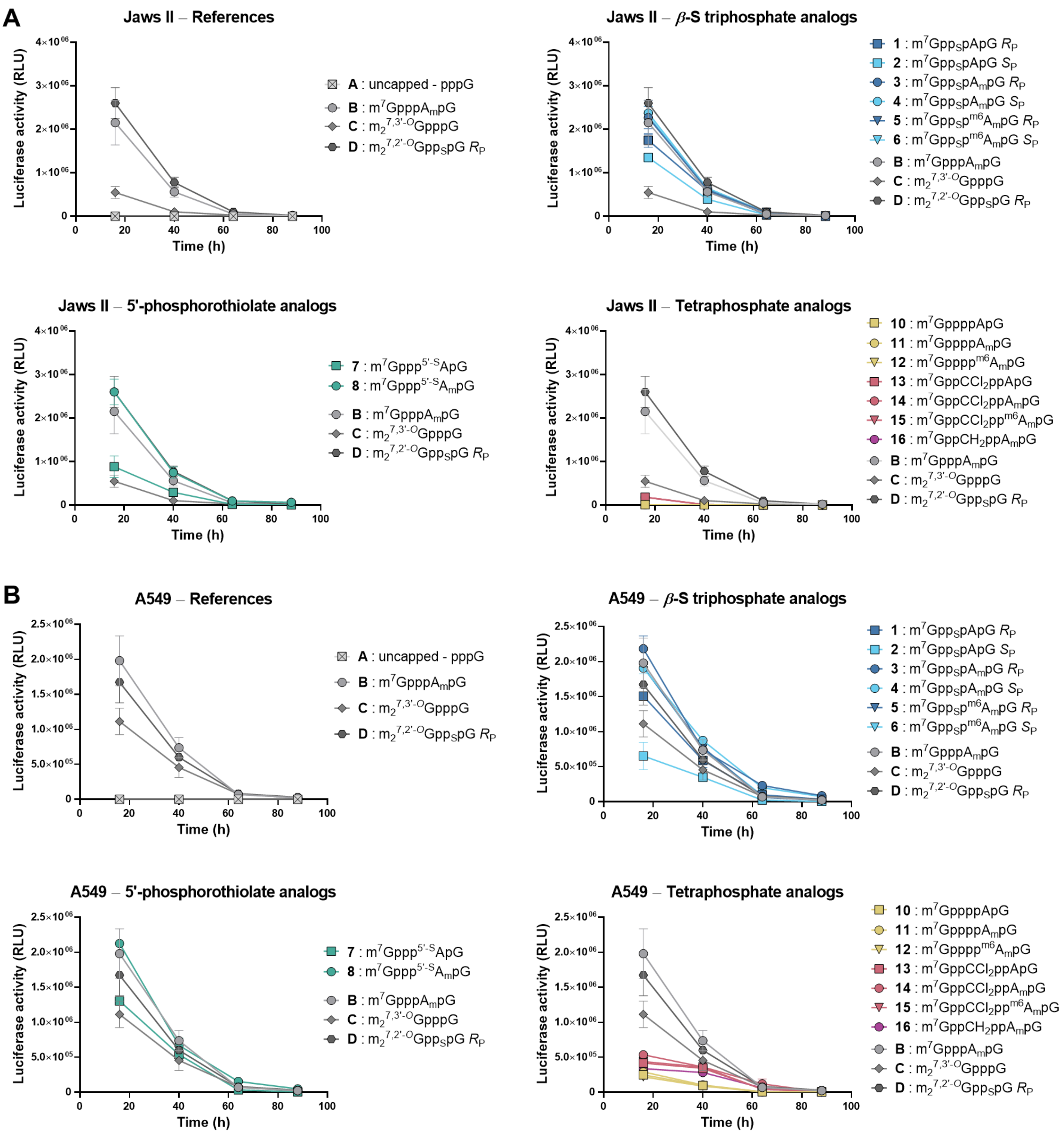

Figure S5. Time-dependent protein expression of capped mRNAs. A) in Jaws II cell line. **B**) in A549 cell line.

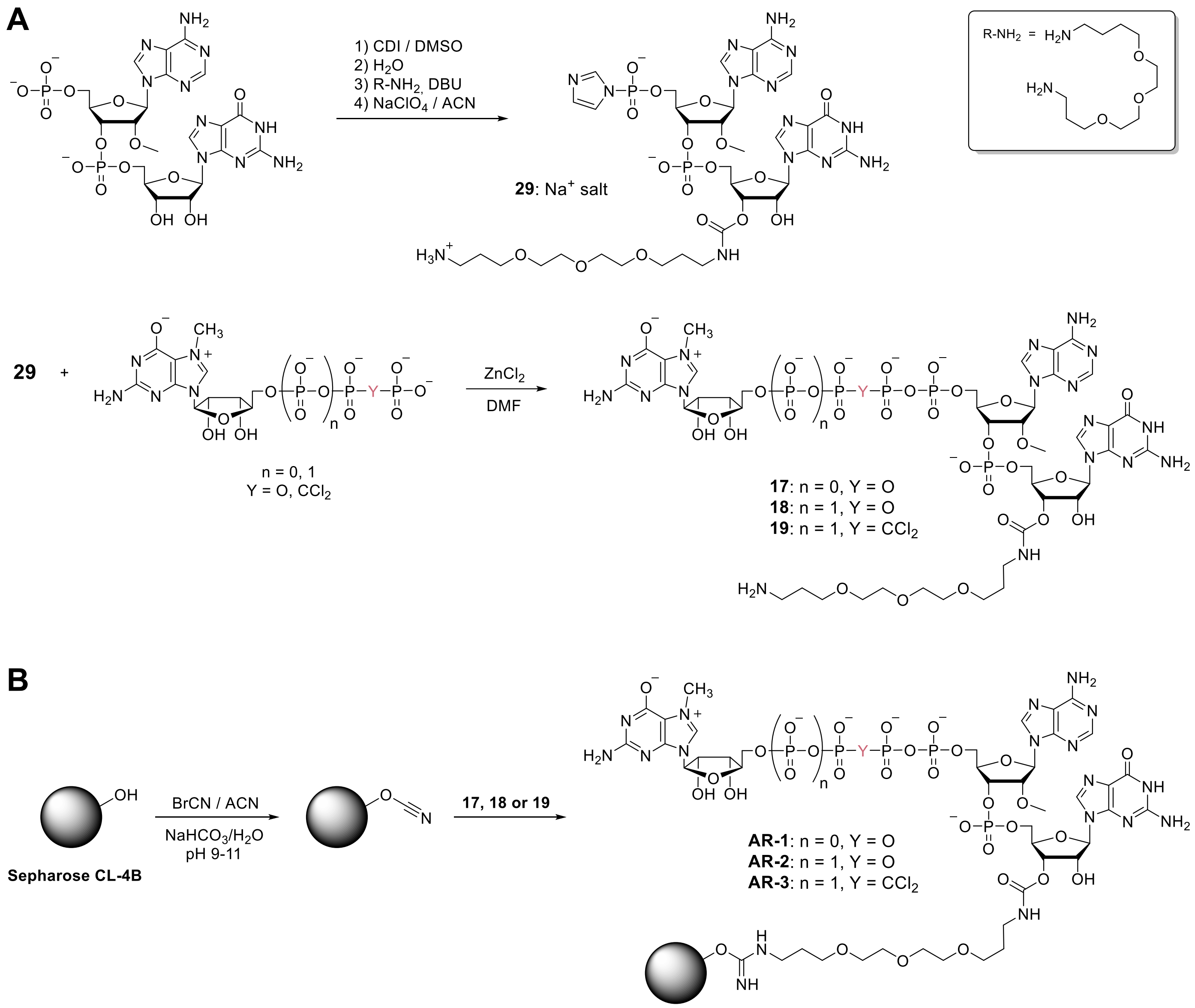

Figure S6. Synthesis of affinity resins. A) Synthesis of functionalized trinucleotide cap analogs 17–19; **B**) immobilization of analogs 17–19 on BrCN-activated Sepharose.

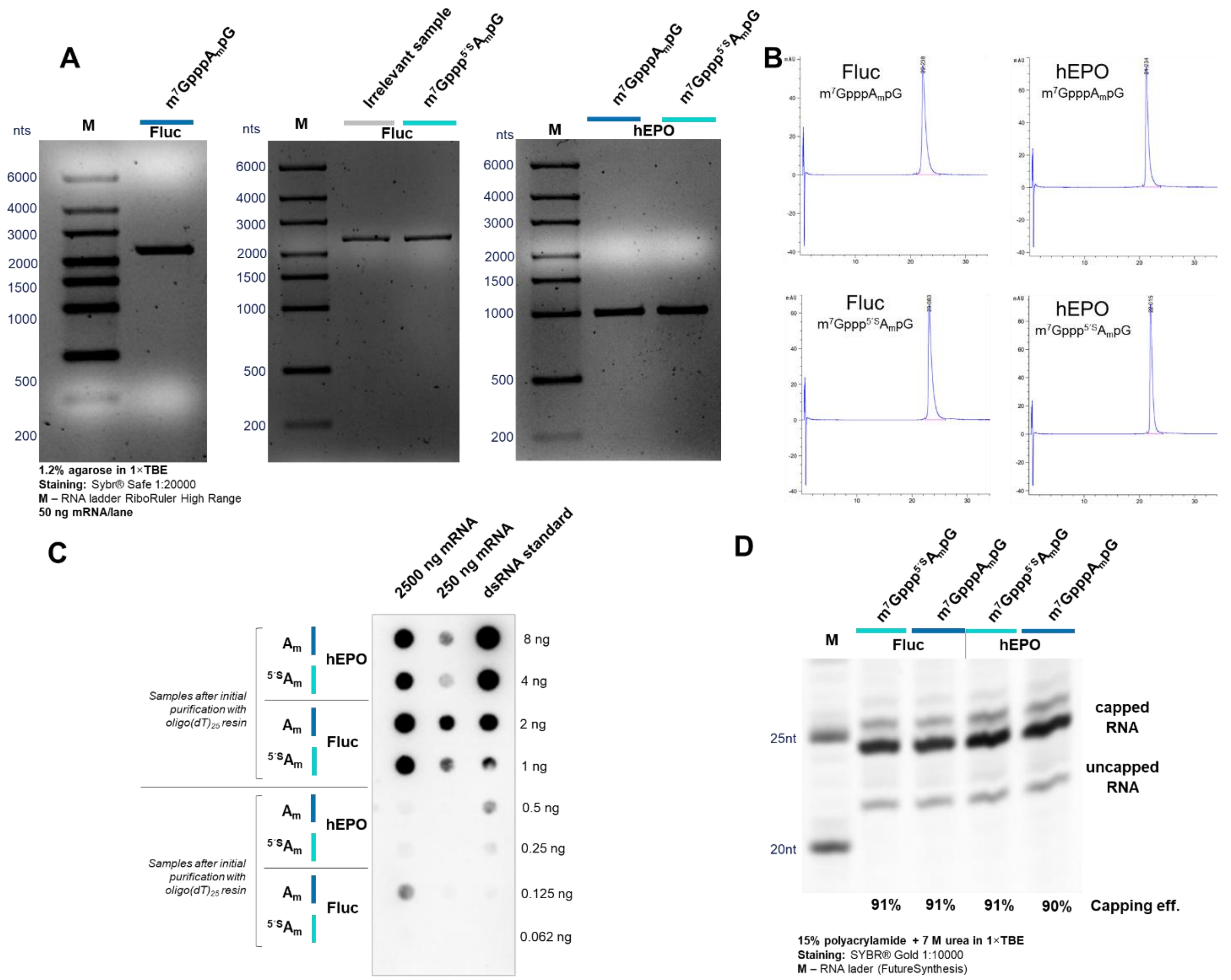

Figure S7. Quality control of mRNA encoding firefly luciferase (Fluc) and human erythropoietin (hEPO) for *in vivo* studies. (**A**) Integrity analysis: 50 ng of each mRNA was analysed using 1.2% agarose gel in 1×TBE. (**B**) Purity analysis by RP-HPLC: 1 µg of mRNA was applied on bioZen^{TM} 2.6 µm Oligo LC column (Phenomenex) and separated using linear gradient of acetonitrile (10-20%) in 0.1 M TEAA pH 7.0 at 55°C. (**C**) Detection of dsRNA: mRNA samples purified with oligo(dT)$_{25}$ resin and HPLC were immobilized on the nylon membrane (Hybond^{TM}-N^{+}, Amersham^{TM}), incubated with dsRNA-specific J2 antibody (SCICONS) and secondary anti-mouse HRP-conjugated antibody (Cell Signaling Technology), followed by chemiluminescence signal detection (ImageQuant 800, Amersham^{TM}). (**D**) Capping efficiency: densitometric quantification of bands intensities corresponding to 5´ mRNA fragments cleaved by 5´UTR complementary ribozyme. 15% polyacrylamide gel with 7 M urea and 1× TBE was used for separation of digested RNA fragments.

Compounds characterization

m^{7}Gpp$_S$pApG *R*$_P$ (**1**): **HRMS ESI(-):** *m/z* 1146.11096 (Calc. [M-H]$^-$ $C_{31}H_{40}N_{15}O_{23}P_4S^-$ 1146.10981)

m^{7}Gpp$_S$pApG *S*$_P$ (**2**): **HRMS ESI(-):** *m/z* 1146.11147 (Calc. [M-H]$^-$ $C_{31}H_{40}N_{15}O_{23}P_4S^-$ 1146.10981)

m^{7}Gpp$_S$pA$_m$pG *R*$_P$ (**3**): **^{1}H NMR (500 MHz, D_2O, 25°C):** *δ* = 9.17 (s, 1H, H8$_{m7G}$), 8.59 (s, 1H, H8$_A$), 8.30 (s, 1H, H2$_A$), 8.01 (s, 1H, H8$_G$), 6.07 (d, $^3J_{H-H}$ = 5.5 Hz, 1H, H1'$_A$), 5.94 (d, $^3J_{H-H}$ = 3.6 Hz, 1H, H1'$_{m7G}$), 5.82 (d, $^3J_{H-H}$ = 5.7 Hz, 1H, H1'$_G$), 5.01 – 4.96 (m, 1H, H3'$_A$), 4.83 (m, overlapped with HDO, 1H, H2'$_G$), 4.65 (dd, $^3J_{H-H}$ = 4.9 Hz, $^3J_{H-H}$ = 3.6 Hz, 1H, H2'$_{m7G}$), 4.57 (m, 1H, H3'$_G$), 4.54 – 4.49 (m, 3H, H2'$_A$, H3'$_{m7G}$, H4'$_A$), 4.44 – 4.39 (m, 1H, H5'm7G), 4.36 (m, 2H, H4'$_G$, H4'$_{m7G}$), 4.33 – 4.22 (m, 3H, H5"$_{m7G}$, H5'$_A$, H5"$_A$), 4.21 – 4.18 (m, 2H, H5'G, H5'$_G$), 4.06 (s, 3H, *N*^{7}-CH_3), 3.44 (s, 3H, 2'-*O*-CH_3); **^{31}P NMR (202.5 MHz, D_2O, H_3PO_4, 25°C):** *δ* = 30.99 (m, 1P, P$_β$), 0.02 (s, 1P, P$_{A-G}$), -11.47 (m, 2P, P$_α$, P$_γ$) ppm; **HRMS ESI(-):** *m/z* 1160.12689 (Calc. [M-H]$^-$ $C_{32}H_{42}N_{15}O_{23}P_4S^-$ 1160.12546)

m^{7}Gpp$_S$pA$_m$pG *S*$_P$ (**4**): **^{1}H NMR (500 MHz, D_2O, 25°C):** *δ* = 9.17 (s, 1H, H8$_{m7G}$), 8.58 (s, 1H, H8$_A$), 8.30 (s, 1H, H2$_A$), 8.01 (s, 1H, H8$_G$), 6.08 (d, $^3J_{H-H}$ = 5.6 Hz, 1H, H1'$_A$), 5.93 (d, $^3J_{H-H}$ = 3.8 Hz, 1H, H1'$_{m7G}$), 5.82 (d, $^3J_{H-H}$ = 5.8 Hz, 1H, H1'$_G$), 5.00 (m, 1H, H3'$_A$), 4.80 (m, overlapped with HDO, 1H, H2'$_G$), 4.67 – 4.63 (m, 1H, H2'$_{m7G}$), 4.52 (m, 4H, H2'$_A$, H3'$_G$, H3'$_{m7G}$, H4'$_A$), 4.43 – 4.29 (m, 4H, H4'$_G$, H4'$_{m7G}$, H5'$_{m7G}$, H5"$_{m7G}$), 4.26 (m, 2H, H5'$_A$, H5"$_A$), 4.22 – 4.15 (m, 2H, H5'$_G$, H5"$_G$), 4.07 (s, 3H, *N*^{7}-CH_3), 3.45 (s, 3H, 2'-*O*-CH_3); **^{31}P NMR (202.5 MHz, D_2O, H_3PO_4, 25°C):** *δ* =30.90 (m, 1P, P$_β$), -0.02 (s, 1P, P$_{A-G}$), -11.47 (m, 2P, P$_α$, P$_γ$) ppm; **HRMS ESI(-):** *m/z* 1160.12696 (Calc. [M-H]$^-$ $C_{32}H_{42}N_{15}O_{23}P_4S^-$ 1160.12546)

m^{7}Gpp$_S$p^{m6}A$_m$pG *R*$_P$ (**5**): **^{1}H NMR (500 MHz, D_2O, 25°C):** *δ* = 9.16 (s, 1H, H8$_{m7G}$), 8.54 (s, 1H, H8$_A$), 8.27 (s, 1H, H2$_A$), 8.01 (s, 1H, H8$_G$), 6.06 (d, $^3J_{H-H}$ = 5.3 Hz, 1H, H1'$_A$), 5.96 (d, $^3J_{H-H}$ = 3.5 Hz, 1H, H1'$_{m7G}$), 5.83 (d, $^3J_{H-H}$ = 5.8 Hz, 1H, H1'$_G$), 4.98 (m, 1H, H3'$_A$), 4.81 (m, overlapped with HDO, 1H, H2'$_G$), 4.68 – 4.64 (m, 1H, H2'$_{m7G}$), 4.57 (m, 1H, H3'$_G$), 4.52 (m, 3H, H2'$_A$, H4'$_A$), 4.51 – 4.39 (m, 3H, H3'$_{m7G}$, H4'$_{m7G}$, H5'$_{m7G}$), 4.35 (m, 2H, H4'$_G$, H5"$_{m7G}$), 4.28 (m, 3H, H5'$_A$, H5"$_A$), 4.19 (m, 2H, H5'$_G$, H5"$_G$), 4.07 (s, 3H, *N*^{7}-CH_3), 3.45 (s, 3H, 2'-*O*-CH_3), 3.20 (s, 3H, *N*^{6}-CH_3); **^{31}P NMR (202.5 MHz, D_2O, H_3PO_4, 25°C):** 30.89 (m, 1P, P$_β$), 0.02 (s, 1P, P$_{A-G}$), -11.49 (m, 2P, P$_α$, P$_γ$) ppm; **HRMS ESI(-):** *m/z* 1174.14244 (Calc. [M-H]$^-$ $C_{33}H_{44}N_{15}O_{23}P_4S^-$ 1174.14111).

m^{7}Gpp$_S$p^{m6}A$_m$pG *S*$_P$ (**6**): **^{1}H NMR (500 MHz, D_2O, 25°C):** *δ* = 9.14 (s, 1H, H8$_{m7G}$), 8.51 (s, 1H, H8$_A$), 8.25 (s, 1H, H2$_A$), 8.00 (s, 1H, H8$_G$), 6.05 (d, $^3J_{H-H}$ = 5.4 Hz, 1H, H1'$_A$), 5.93 (d, $^3J_{H-H}$ = 3.8 Hz, 1H, H1'$_{m7G}$), 5.82 (d, $^3J_{H-H}$ = 5.9 Hz, 1H, H1'$_G$), 5.02 – 4.96 (m, 1H, H3'$_A$), 4.79 (m, overlapped with HDO, 1H, H2'$_G$), 4.65 (m, 1H, H2'$_{m7G}$), 4.56 – 4.48 (m, 4H, H2'$_A$, H3'$_G$, H3'$_{m7G}$, H4'$_A$), 4.44 – 4.30 (m, 4H, H4'$_G$, H4'$_{m7G}$, H5'$_{m7G}$, H5"$_{m7G}$), 4.27 (s, 2H, H5"$_A$, H5"$_A$), 4.22 – 4.15 (m, 2H, H5'$_G$, H5"$_G$), 4.07 (s, 3H, *N*^{7}-CH_3), 3.46 (s, 3H, 2'-*O*-CH_3), 3.18 (s, 3H, *N*^{6}-CH_3); **^{31}P NMR (202.5 MHz, D_2O, H_3PO_4, 25°C):** 30.86 (m, 1P, P$_β$), -0.02 (s, 1P, P$_{A-G}$),, -11.47 (m, 2P, P$_α$, P$_γ$) ppm; **HRMS ESI(-):** *m/z* 1174.14250 (Calc. [M-H]$^-$ $C_{33}H_{44}N_{15}O_{23}P_4S^-$ 1174.14111

m^{7}Gppp$^{5'-S}$ApG (**7**): **HRMS ESI(-):** *m/z* 1146.11080 (Calc. [M-H]$^-$ $C_{31}H_{40}N_{15}O_{23}P_4S^-$ 1146.10981)

m^{7}Gppp$^{5'-S}$A$_m$pG (**8**): **HRMS ESI(-):** *m/z* 1160.12658 (Calc. [M-H]$^-$ $C_{31}H_{40}N_{15}O_{23}P_4S^-$ 1146.10981)

m^{7}GppCH$_2$pA$_m$pG (**9**): **HRMS ESI(-):** *m/z* 1142.17023 (Calc. [M-H]$^-$ $C_{33}H_{44}N_{15}O_{23}P_4^-$ 1142.16904)

m^{7}GppppApG (**10**): **^{1}H NMR (500 MHz, D_2O, 25°C):** *δ* = 9.15 (s, 1H, H8$_{m7G}$), 8.54 (s, 1H, H2$_A$), 8.27 (s, 1H, H8$_A$), 7.95 (s, 1H, H8$_G$), 5.98 (d, $^3J_{H,H}$ = 5.3 Hz, 1H, H1'$_A$), 5.91 (d, $^3J_{H,H}$ = 3.7 Hz, 1H, H1'm7$_G$), 5.79 (d, $^3J_{H,H}$ = 5.5 Hz, 1H, H1'$_G$), 4.79 (m, overlapped with HDO, 2H, H3'$_A$, H2'$_G$), 4.71 (m, 1H, H2'$_A$), 4.62 (dd, $^3J_{H,H}$ = 4.8 Hz, $^3J_{H,H}$ = 3.7 Hz, 1H, H2'$_{m7G}$), 4.50 (m, 3H, H3'$_G$, H3'$_{m7G}$, H4'$_A$), 4.39 (m, 2H, H4'$_{m7G}$', H5'$_{m7G}$), 4.33 (m, 2H, H4'$_G$, H5"$_{m7G}$), 4.29 – 4.21 (m, 3H, H5'$_A$, H5"$_A$, H5'$_G$), 4.15 (dt, *J* = 11.5, 4.2 Hz, 1H, H5"$_G$), 4.05 (s, 3H, *N*^{7}-CH_3) ppm; **^{31}P NMR (202.5 MHz, D_2O, H_3PO_4, 25°C):** *δ* = 0.18 (s, 1P, P$_{A-G}$), -10.49 (P$_α$, P$_δ$), -21.97 (m, 2P, P$_β$, P$_γ$) ppm; **HRMS ESI(-):** *m/z* 1210.10009 (*calcd for* $C_{31}H_{41}N_{15}O_{27}P_5^-$ [M-H]$^-$ 1210.09899);

m^{7}GppppA$_m$pG (**11**): **^{1}H NMR (500 MHz, D_2O, 25°C):** *δ* = 9.17 (s, 1H, H8$_{m7G}$), 8.69 (s, 1H, H2$_A$), 8.35 (s, 1H, H8$_A$), 8.04 (s, 1H, H8$_G$), 6.12 (d, $^3J_{H,H}$ = 4.3 Hz, 1H, H1'$_A$), 5.80 (d, $^3J_{H,H}$ = 3.5 Hz, 1H, H1'$_{m7G}$), 5.76 (d, $^3J_{H,H}$ = 5.3 Hz, 1H, H1'$_G$), 4.91 (m, 1H, H3'$_A$), 4.77 – 4.73 (m, 1H, H2'$_G$), 4.56 – 4.55 (m, 1H, H2'$_{m7G}$), 4.52 (m, 1H, H4'$_A$), 4.49 – 4.46 (m, 2H, H3'$_G$, H3'$_{m7G}$), 4.43 – 4.37 (m, 3H, H5'$_{m7G}$, H5"$_{m7G}$, H2'$_A$), 4.35 – 4.33 (m, 2H, H4'$_G$, H4"$_{m7G}$), 4.29 – 4.19 (m, 3H, H5'$_A$, H5"$_A$, H5'$_G$), 4.18 – 4.14 (m, 1H, H5"$_G$), 4.02 (s, 3H, *N*^{7}-CH_3), 3.51 (s, 3H, 2'-*O*-CH_3) ppm; **^{31}P NMR (202.5 MHz, D_2O, H_3PO_4, 25°C):** *δ* = -0.03 (s, 1P, P$_{A-G}$), -10.60 (m, 2P, P$_α$, P$_δ$), -21.92 (m, 2P, P), -22.18 (m, 2P, P) ppm; **HRMS ESI(-):** *m/z* 1224.11521 (*calcd for* $C_{32}H_{43}N_{15}O_{27}P_5^-$ [M-H]$^-$ 1224.11464);

m^{7}Gpppp^{m6}A$_m$pG (**12**): **^{1}H NMR (500 MHz, D_2O, 25°C):** *δ* = 9.17 (s, 1H, H8$_{m7G}$), 8.50 (s, 1H, H2$_A$), 8.24 (s, 1H, H8$_A$), 7.95 (s, 1H, H8$_G$), 6.01 (d, $^3J_{H,H}$ = 5.3 Hz, 1H, H1'$_A$), 5.95 (d, $^3J_{H,H}$ = 3.7 Hz, 1H, H1'$_{m7G}$), 5.80 (d, $^3J_{H,H}$ = 5.6 Hz, 1H, H1'$_G$), 4.92 (m, 1H, H3'$_A$), 4.88 – 4.82 (m, 2H, H2'$_G$), 4.64 (dd, $^3J_{H,H}$ = 4.8 Hz, $^3J_{H,H}$ **=** 3.7 Hz, 1H, H2'$_{m7G}$), 4.53 (m, 1H, H3'$_G$), 4.51 – 4.46 (m, 2H, H3'$_{m7G}$, H4'$_A$), 4.42 (m, 1H, H2'$_A$), 4.39 (m, 2H, H4'$_G$, H5'$_{m7G}$), 4.36 – 4.29 (m, 2H, H4"$_{m7G}$, H5'$_{m7G}$), 4.29 – 4.11 (m, 4H, H5'$_A$, H5"$_A$, H5'$_G$, H5"$_G$), 4.06 (s, 3H, *N*^{7}-CH_3), 3.42 (s, 3H, 2'-*O*-CH_3), 3.17 (s, 3H, *N*^{6}-CH_3) ppm; **^{31}P NMR (202.5 MHz, D_2O, H_3PO_4, 25°C):** *δ* = -0.04 (s, 1P, P$_{A-G}$), -10.50 (m, 2P, P$_α$, P$_δ$), -22.05 (m, 2P, P$_β$, P$_γ$) ppm; **HRMS ESI(-):** *m/z* 1238.13119 (*calcd for* $C_{33}H_{45}N_{15}O_{27}P_5^-$ [M-H]$^-$ 1238.13029);

m^{7}GppCCl$_2$ppApG (**13**): **^{1}H NMR (500 MHz, D_2O, 25°C):** *δ* = 9.19 (s, 1H, H8$_{m7G}$), 8.49 (s, 1H, H2$_A$), 8.22 (s, 1H, H8$_A$), 7.92 (s, 1H, H8$_G$), 5.98 (d, $^3J_{H,H}$ = 5.4 Hz, 1H, H1'$_A$), 5.91 (d, $^3J_{H,H}$ = 3.8 Hz, 1H, H1'$_{m7G}$), 5.80 (d, $^3J_{H,H}$ = 5.6 Hz, 1H, H1'$_G$), 4.80 (m, overlapped with HDO, 2H, H2'$_G$, H3'$_A$), 4.72 (m, H2'$_A$), 4.62 (m, 1H, H2'$_{m7G}$), 4.55 – 4.48 (m, 3H, H3'$_{m7G}$, H3'$_G$, H4'$_A$), 4.43 (m, 1H, H5'$_{m7G}$), 4.35 (m, 3H, H4'$_G$, H4'$_{m7G}$, H5"$_{m7G}$), 4.25 (m, 3H, H5'$_A$, H5"$_A$, H5'$_G$), 4.17 (m, 1H, H5"$_G$), 4.04 (s, 3H, *N*^{7}-CH_3) ppm; **^{31}P NMR (202.5 MHz,**

D_2O, H_3PO_4, 25°C): *δ* = 0.27 (s, 1P, P_{A-G}), -0.53 – -1.36 (m, 2P, $P_β$, $P_γ$), -10.25 (d, $^2J_{P-P}$ = 28.6 Hz, 1P, $P_δ$), -10.57 (d, $^2J_{P-P}$ = 28.8 Hz, 1P, $P_α$) ppm; **HRMS ESI(-):** *m/z* 1276.04367 (*calcd for* $C_{32}H_{41}Cl_2N_{15}O_{26}P_5^-$ [M-H]⁻ 1276.04178);

m^{7}GppCCl$_2$ppA$_m$pG (**14**): **^{1}H NMR (500 MHz, D_2O, 25°C)**: *δ* = 9.19 (s, 1H, H8$_{m7G}$), 8.56 (s, 1H, H8$_{m7G}$), 8.25 (s, 1H, H2$_A$), 7.95 (s, 1H, H8$_G$), 6.08 (d, $^3J_{H,H}$ = 5.1 Hz, 1H, H1'$_A$), 5.85 (d, $^3J_{H,H}$ = 3.6 Hz, 1H, H1'$_{m7G}$), 5.80 (d, $^3J_{H,H}$ = 5.5 Hz, 1H, H1'$_G$), 4.91 (m, 1H, H3'$_A$), 4.79 (m, 1H, H2'$_G$), 4.58 – 4.55 (m, 1H, H2'$_{m7G}$), 4.50 (m, 3H, H3'$_G$, H3'$_{m7G}$, H4'$_A$), 4.47 – 4.40 (m, 3H, H2'$_A$, H5'$_{m7G}$, H5"$_{m7G}$), 4.39 – 4.32 (m, 3H, H4'$_G$, H4'$_{m7G}$), 4.27 (m, 2H, H5'$_A$, H5"$_A$), 4.19 (m, 2H, H5'$_G$, H5"$_G$), 4.01 (s, 3H, *N*^{7}-CH_3), 3.46 (s, 3H, 2'-*O*-CH_3) ppm; **^{31}P NMR (202.5 MHz, D_2O, H_3PO_4, 25°C):** *δ* = 0.02 (s, 1P, P_{A-G}), -0.53 – -1.22 (m, 2P, $P_β$, $P_γ$), -10.22 (d, $^2J_{P-P}$ = 27.5 Hz, 1P, $P_δ$), -10.59 (d, $^2J_{P-P}$ = 28.2 Hz, 1P, $P_α$) ppm; **HRMS ESI(-):** *m/z* 1290.05776 (*calcd for* $C_{33}H_{43}Cl_2N_{15}O_{26}P_5^-$ [M-H]⁻ 1290.05743);

m^{7}GppCCl$_2$pp^{m6}A$_m$pG (**15**): **^{1}H NMR (500 MHz, D_2O, 25°C):** *δ* = 9.17 (s, 1H, H8$_{m7G}$), 8.42 (s, 1H, H2$_A$), 8.16 (s, 1H, H8$_A$), 7.90 (s, 1H, H8$_G$), 6.02 (d, $^3J_{H,H}$ = 5.3 Hz, 1H, H1'$_A$), 5.91 (d, $^3J_{H,H}$ = 3.8 Hz, 1H, H1'$_{m7G}$), 5.80 (d, $^3J_{H,H}$ = 5.7 Hz, 1H, H1'$_G$), 4.92 (m, 1H, H3'$_A$), 4.84 (m, H2'$_G$), 4.61 (m, 1H, H2'$_{m7G}$), 4.53 – 4.48 (m, 3H, H3'$_{m7G}$, H3'$_G$, H4'$_G$), 4.44 (m, 2H, H2'$_A$, H5'$_{m7G}$), 4.36 (m, 3H, H4'$_{m7G}$, H4'$_A$, H5"$_{m7G}$), 4.25 (m, 2H, H5'$_A$, H5"$_A$), 4.21 – 4.17 (m, 2H, H5'$_G$, H5"$_G$), 4.04 (s, 3H, *N*^{7}-CH_3), 3.42 (s, 3H, 2'-*O*-CH_3), 3.12 (s, 3H, CH_3 *N*^{6}-CH_3) ppm; **^{31}P NMR (202.5 MHz, D_2O, H_3PO_4, 25°C):** *δ* = 0.08 (s, 1P, P_{A-G}), -0.77 (d, $^2J_{P-P}$ = 28.1 Hz, 1P, P), -1.12 (d, $^2J_{P-P}$ = 28.0 Hz, 1P, P), -10.31 (d, $^2J_{P-P}$ = 28.5 Hz, 1P, $P_δ$), -10.65 (d, $^2J_{P-P}$ = 28.6 Hz, 1P, $P_α$) ppm; **HRMS ESI(-):** *m/z* 1304.07353 (*calcd for* $C_{34}H_{45}Cl_2N_{15}O_{26}P_5^-$ [M-H]⁻ 1304.07308);

m^{7}GppCH$_2$ppA$_m$pG (**16**): **^{1}H NMR (500 MHz, D_2O, 25°C)**: *δ* = 9.24 (s, 1H, H8$_{m7G}$), 8.77 (s, 1H, H2$_A$), 8.37 (s, 1H, H8$_A$), 8.15 (s, 1H, H8$_G$), 6.17 (d, $^3J_{H,H}$ = 3.6 Hz, 1H, H1'$_A$), 5.79 (d, $^3J_{H,H}$ = 4.9 Hz, 1H, H1'$_G$), 5.77 (d, $^3J_{H,H}$ = 2.9 Hz, 1H, H1'$_{m7G}$), 4.89 – 4.84 (m, 1H, H3'$_A$), 4.66 (m, 1H, H2'$_G$), 4.54 (m, 1H, H4'$_A$), 4.52 – 4.48 (m, 2H, H2'$_{m7G}$, H3'$_{m7G}$), 4.46 (m, 1H, H3'$_G$), 4.43 – 4.37 (m, 3H, H2'$_A$, H5'$_{m7G}$, H5" $_{m7G}$), 4.35 (m, 2H, H4'$_G$, H4'$_{m7G}$), 4.27 (m, 3H, H5'$_A$, H5"$_A$, H5'$_G$), 4.22 – 4.15 (m, 1H, H5"$_G$), 3.97 (s, 3H, *N*^{7}-CH_3), 3.59 (s, 3H, 2'-*O*-CH_3), 2.66 (td, $^2J_{H-P}$ = 21.0 Hz, $^2J_{H-H}$ = 4.2 Hz, $^2J_{H-H}$ = 4.2 Hz, 2H, CH_2); **^{31}P NMR (202.5 MHz, D_2O, H_3PO_4, 25°C):** *δ* = 9.11– 8.89 (m, 2P, $P_β$, $P_γ$), -0.06 (s, 1P, P_{A-G}), -10.19 (m, 2P, $P_α$, $P_δ$) ppm; **HRMS ESI(-):** *m/z* 1222.13583 (*calcd for* $C_{33}H_{45}N_{15}O_{26}P_5^-$ [M-H]⁻ 1222.13537).

Compounds Characterization

(1) m^7Gpp_SpApG R_P 17

(2) m^7Gpp_SpApG S_P 19

(3) $m^7Gpp_SpA_mpG$ R_P 21

(4) $m^7Gpp_SpA_mpG$ S_P 27

(5) $m^7Gpp_Sp^{m6}A_mpG$ R_P 33

(6) $m^7Gpp_Sp^{m6}A_mpG$ S_P 39

(7) $m^7Gppp^{5'S}ApG$ 45

(8) $m^7Gppp^{5'S}A_mpG$ 47

(9) $m^7GppCH_2pA_mpG$ 49

(10) $m^7GppppApG$ 51

(11) $m^7GppppA_mpG$ 57

(12) $m^7Gpppp^{m6}A_mpG$ 65

(13) $m^7GppCCl_2ppApG$ 72

(14) $m^7GppCCl_2ppA_mpG$ 78

(15) $m^7GppCCl_2pp^{m6}A_mpG$ 84

(16) $m^7GppCH_2ppA_mpG$ 90

(17) m^7GpppA_mpG-$L13_N$ 97

(18) $m^7GppppA_mpG$-$L13_N$ 100

(19) $m^7GppCCl_2ppA_mpG$-$L13_N$ 103

(20) pApG 106

(21) pA_mpG 108

(22) $p^{m6}A_mpG$ 110

(23) $p^{5'S}ApG$ 112

(24) $p^{5'S}A_mpG$ 114

(25) pCH_2pA_mpG 116

(1) m^{7}Gpp$_{S}$pApG R_{P}	
**Chemical structure**	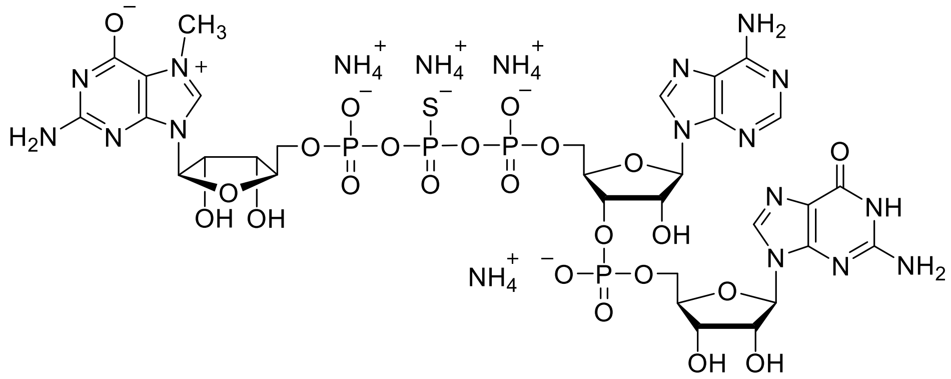
**RP HPLC** Abs. @ 254 nm	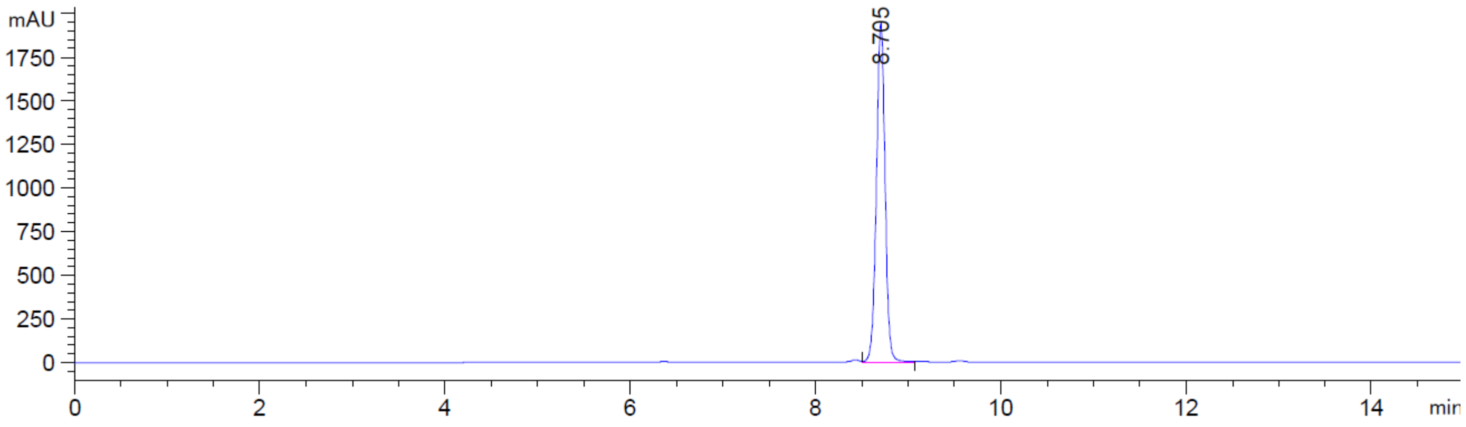

MS (-) ESI

(Calc. $[M-H]^-$ $C_{31}H_{40}N_{15}O_{23}P_4S^-$ 1146.10981)

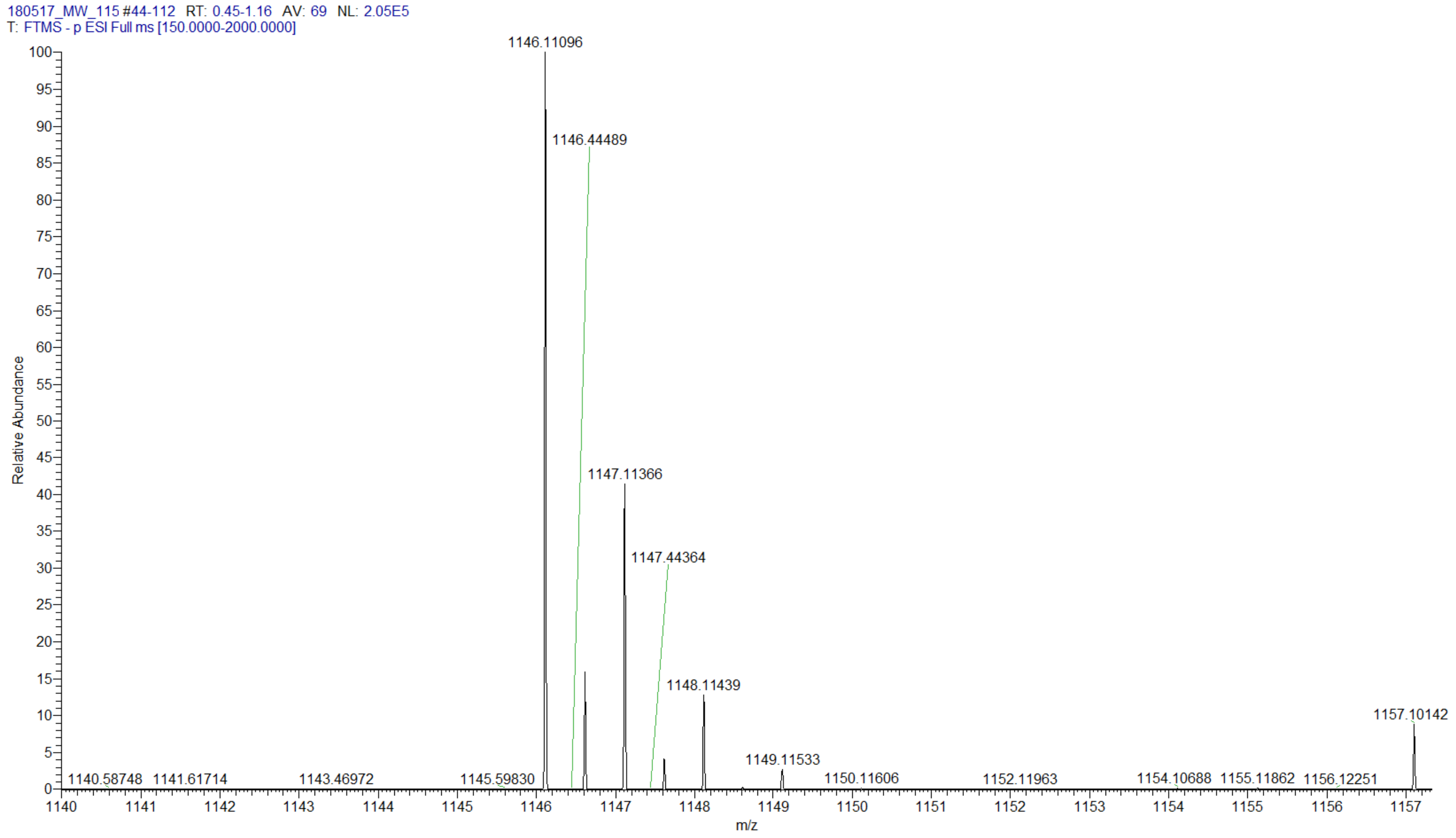

(2) m[7]GppₛpApG S_P	
**Chemical structure**	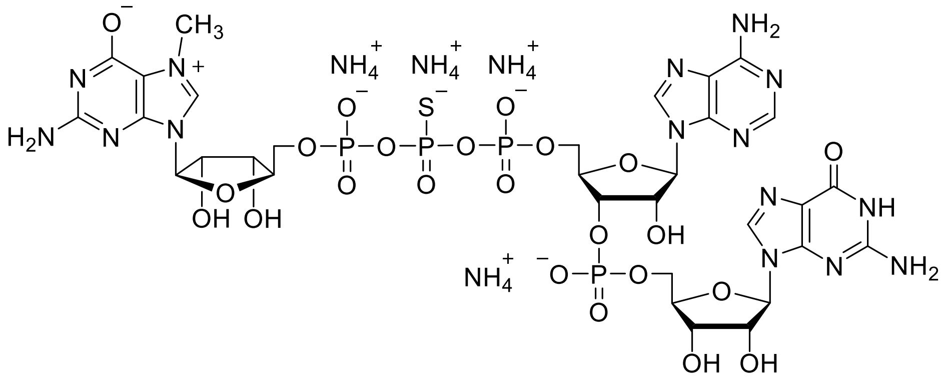
**RP HPLC** Abs. @ 254 nm	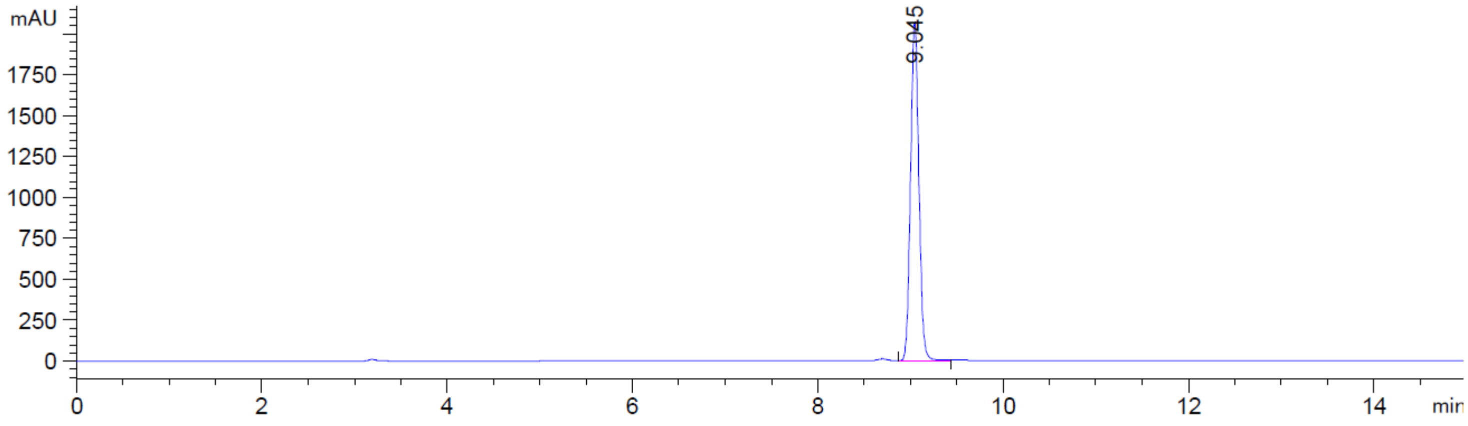

MS (-) ESI
(Calc. $[M-H]^-$ $C_{31}H_{40}N_{15}O_{23}P_4S^-$ 1146.10981)

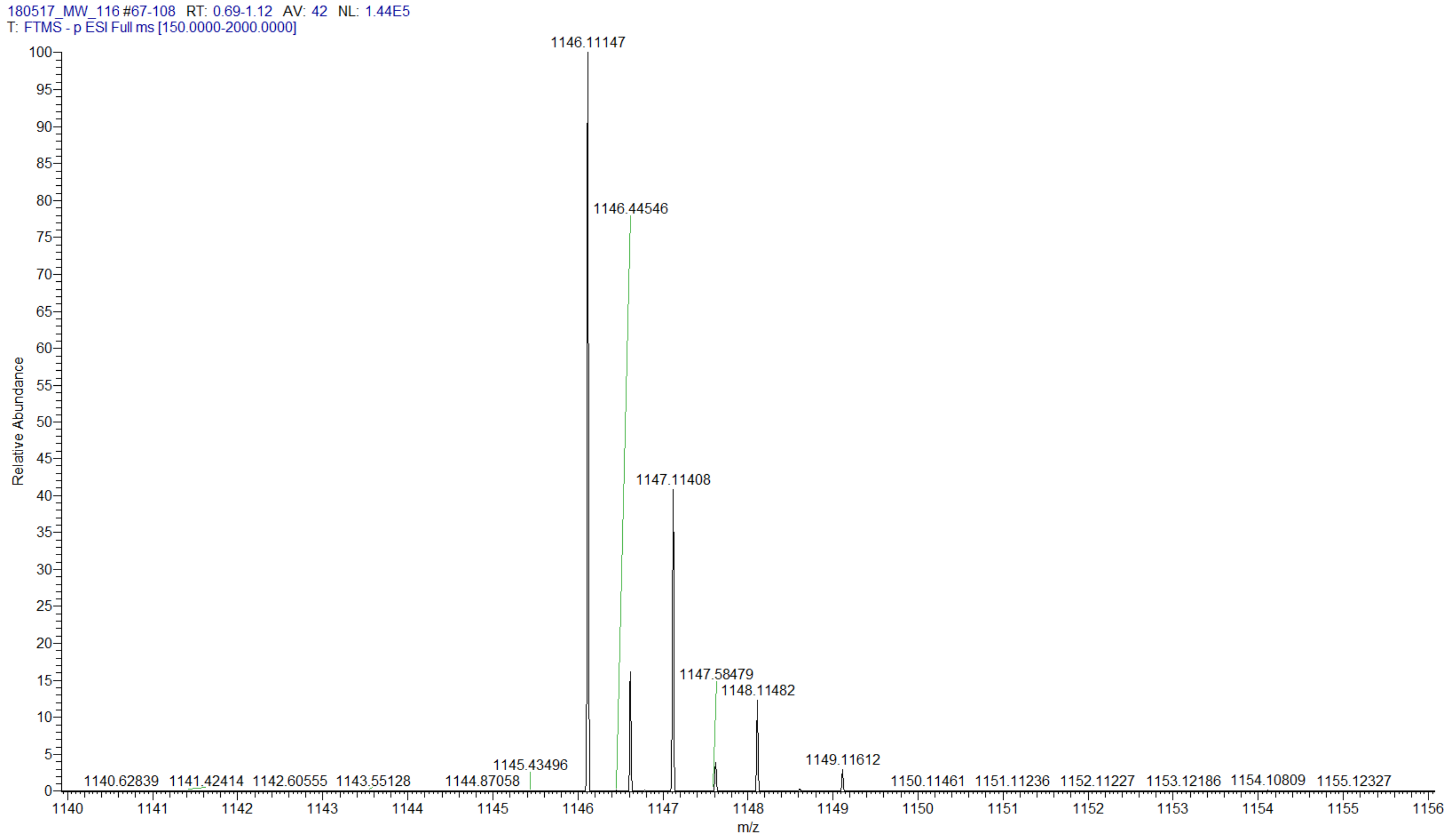

(3) $m^7Gpp_SpA_mpG$ *R*$_P$	
**Chemical structure**	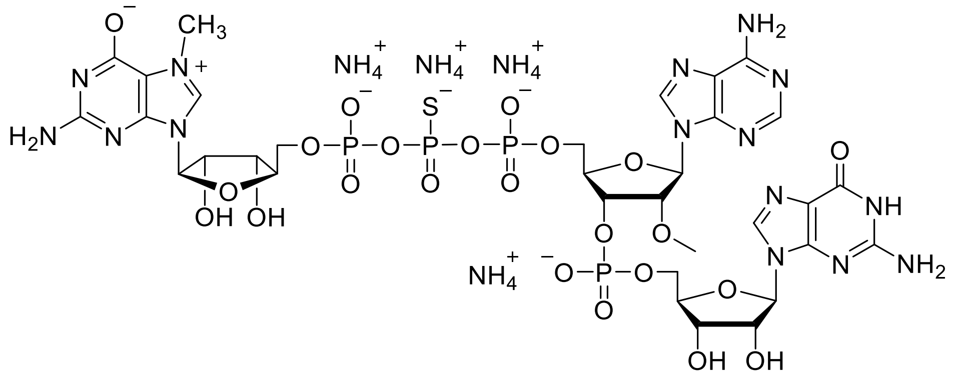
**RP HPLC** Abs. @ 254 nm	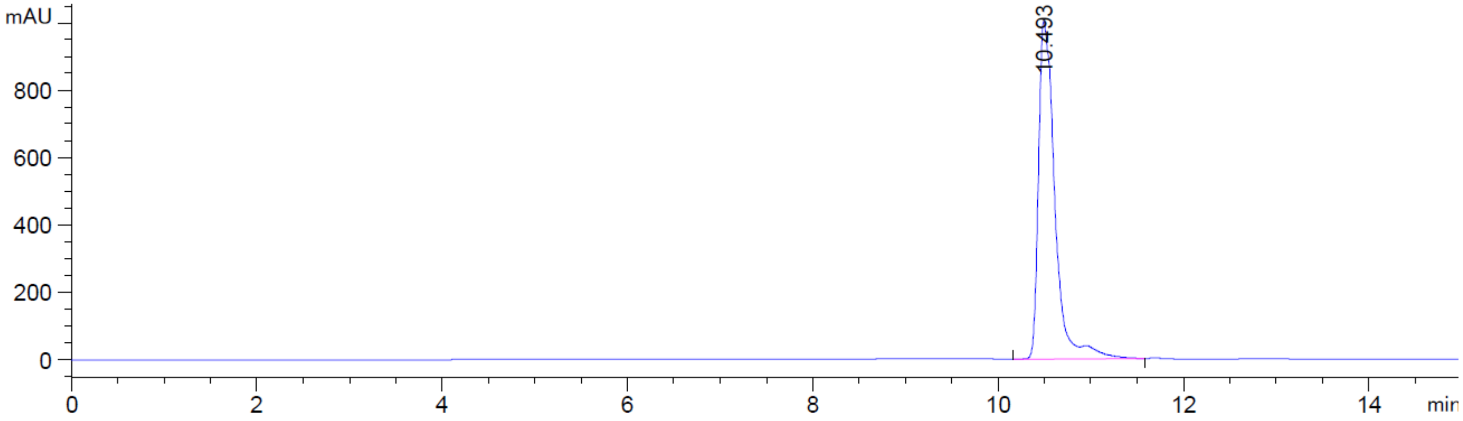

MS (-) ESI

(Calc. $[M-H]^-$ $C_{32}H_{42}N_{15}O_{23}P_4S^-$ 1160.12546)

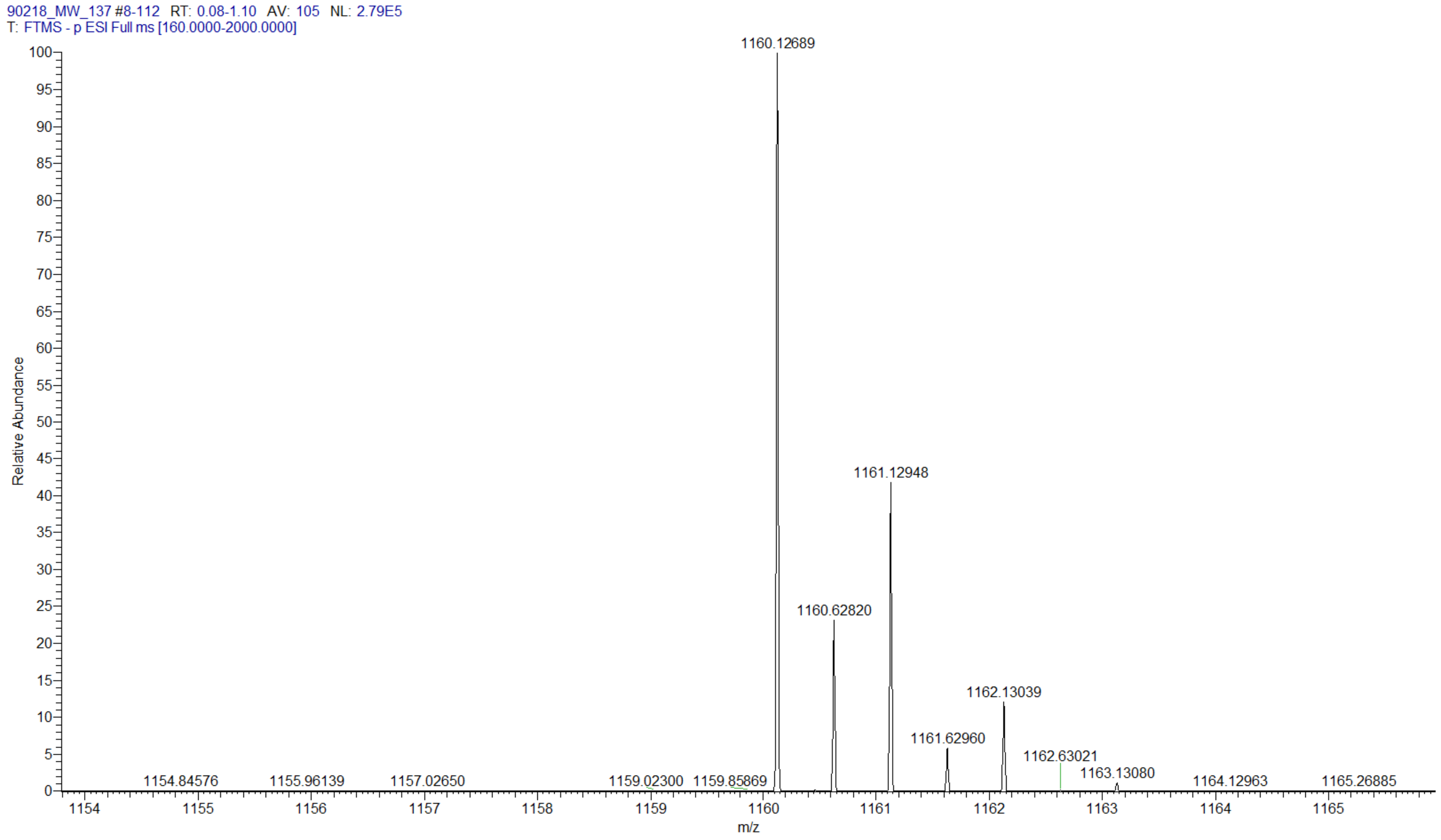

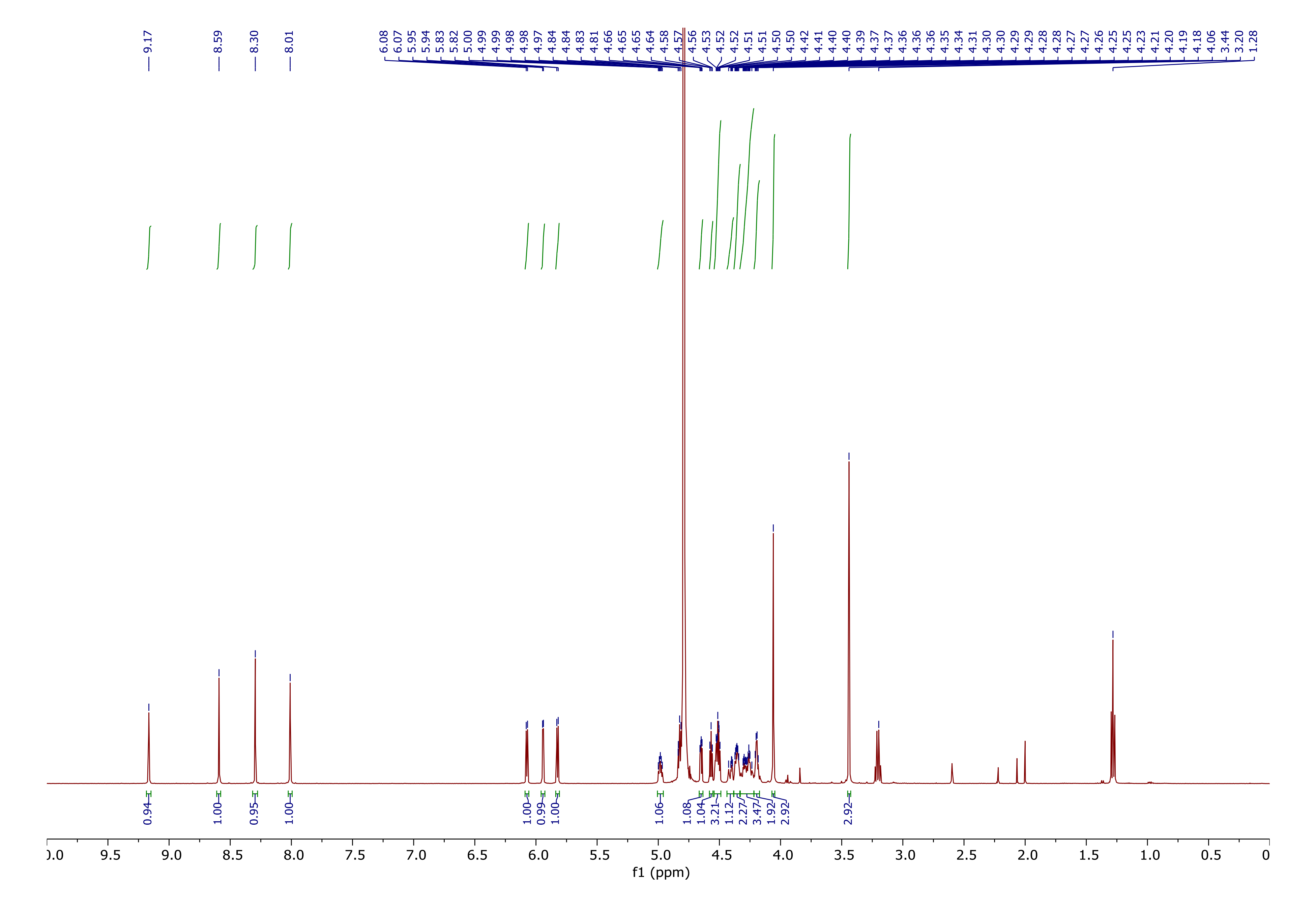

^{1}H NMR (500 MHz, D_2O, 25°C)

COSY NMR (D_2O, 25°)

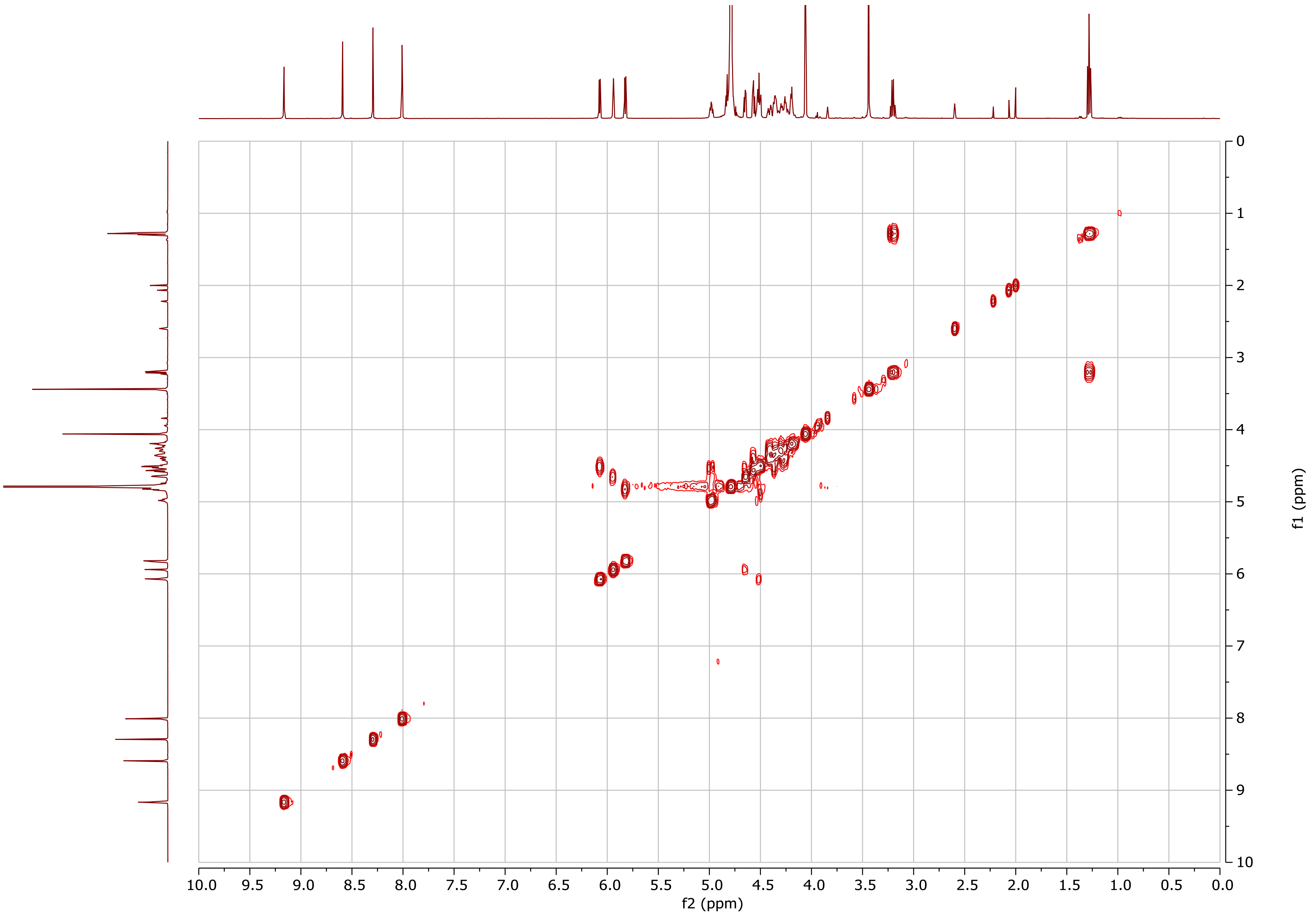

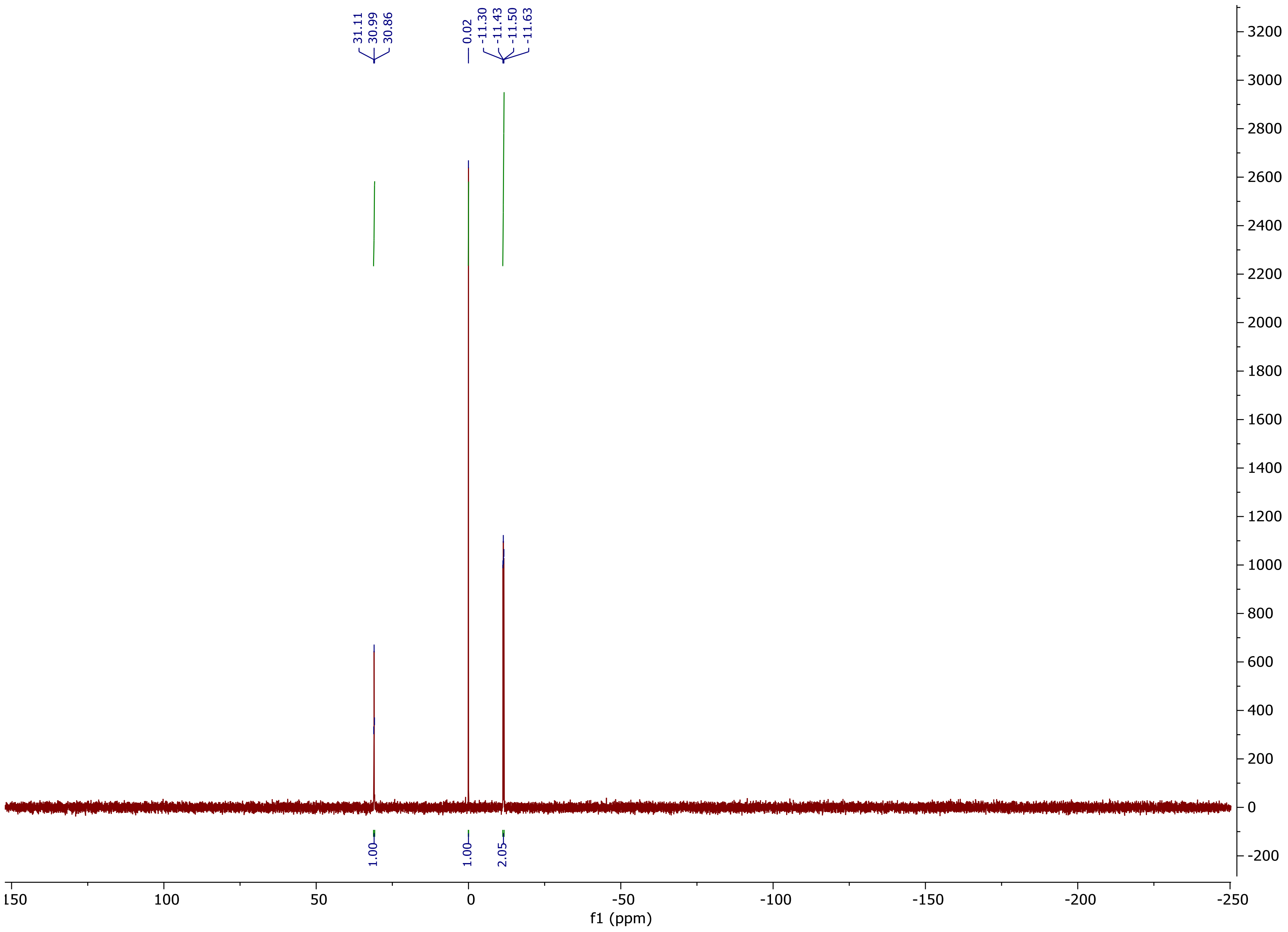

^{31}P NMR (202.5 MHz, D_2O, 25°C)

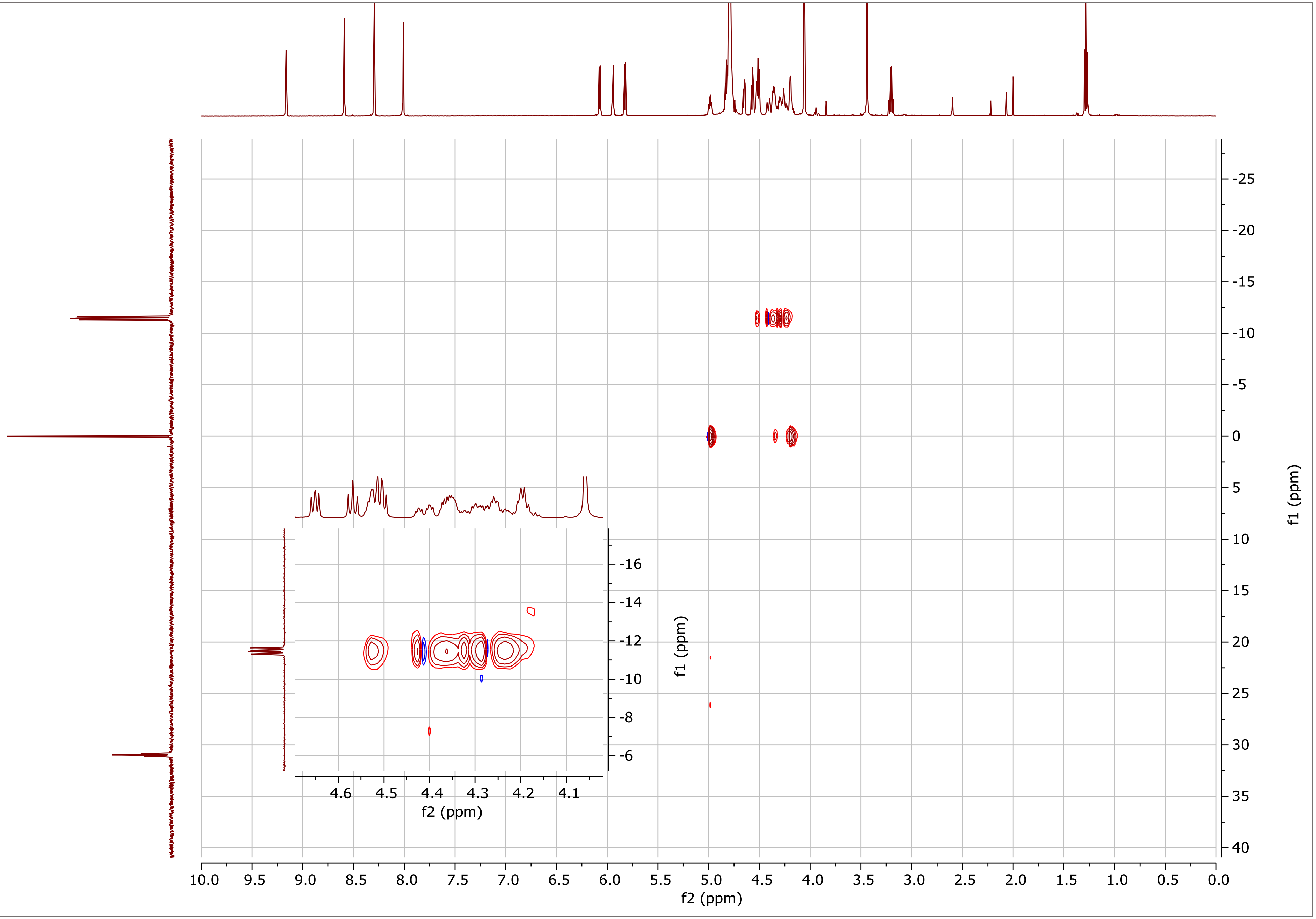

^{1}H-^{31}P HSQC (D_2O, 25°C)

(4) $m^7Gpp_SpA_mpG$ S_P

Chemical structure

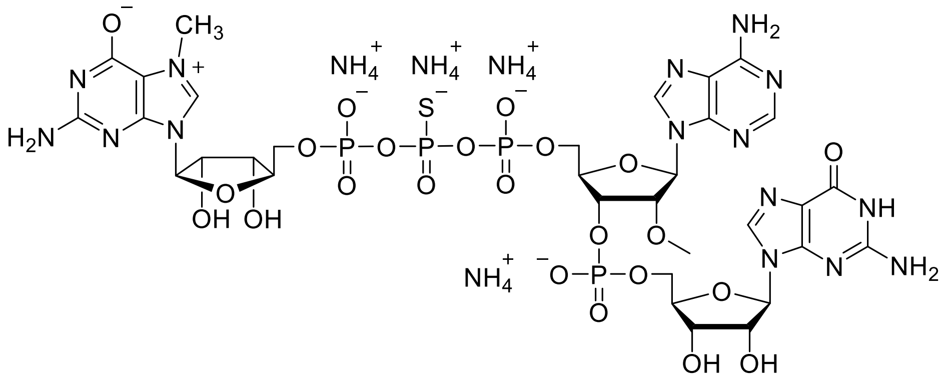

RP HPLC
Abs. @ 254 nm

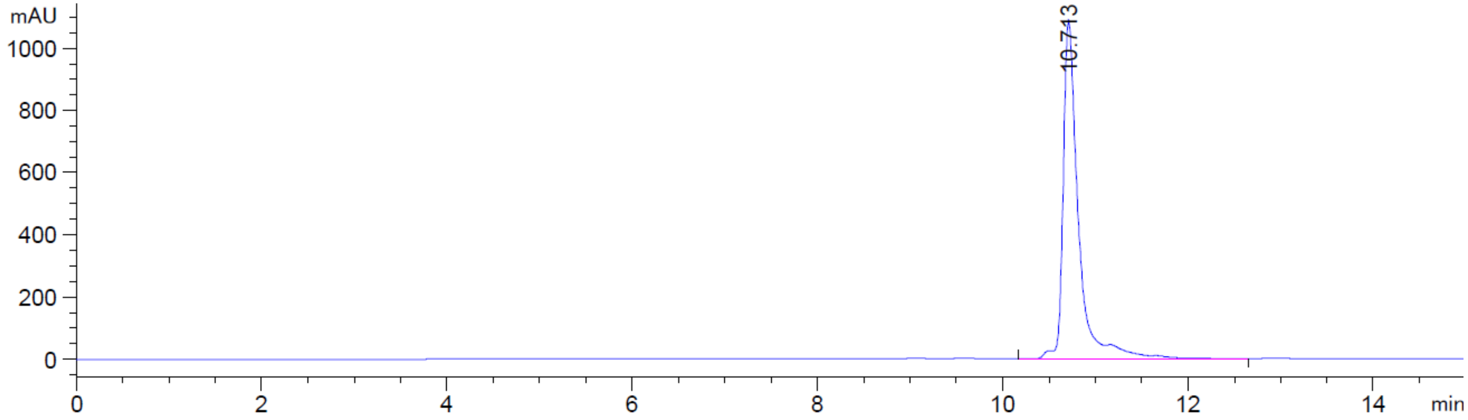

MS (-) ESI

(Calc. $[M-H]^-$ $C_{32}H_{42}N_{15}O_{23}P_4S^-$ 1160.12546)

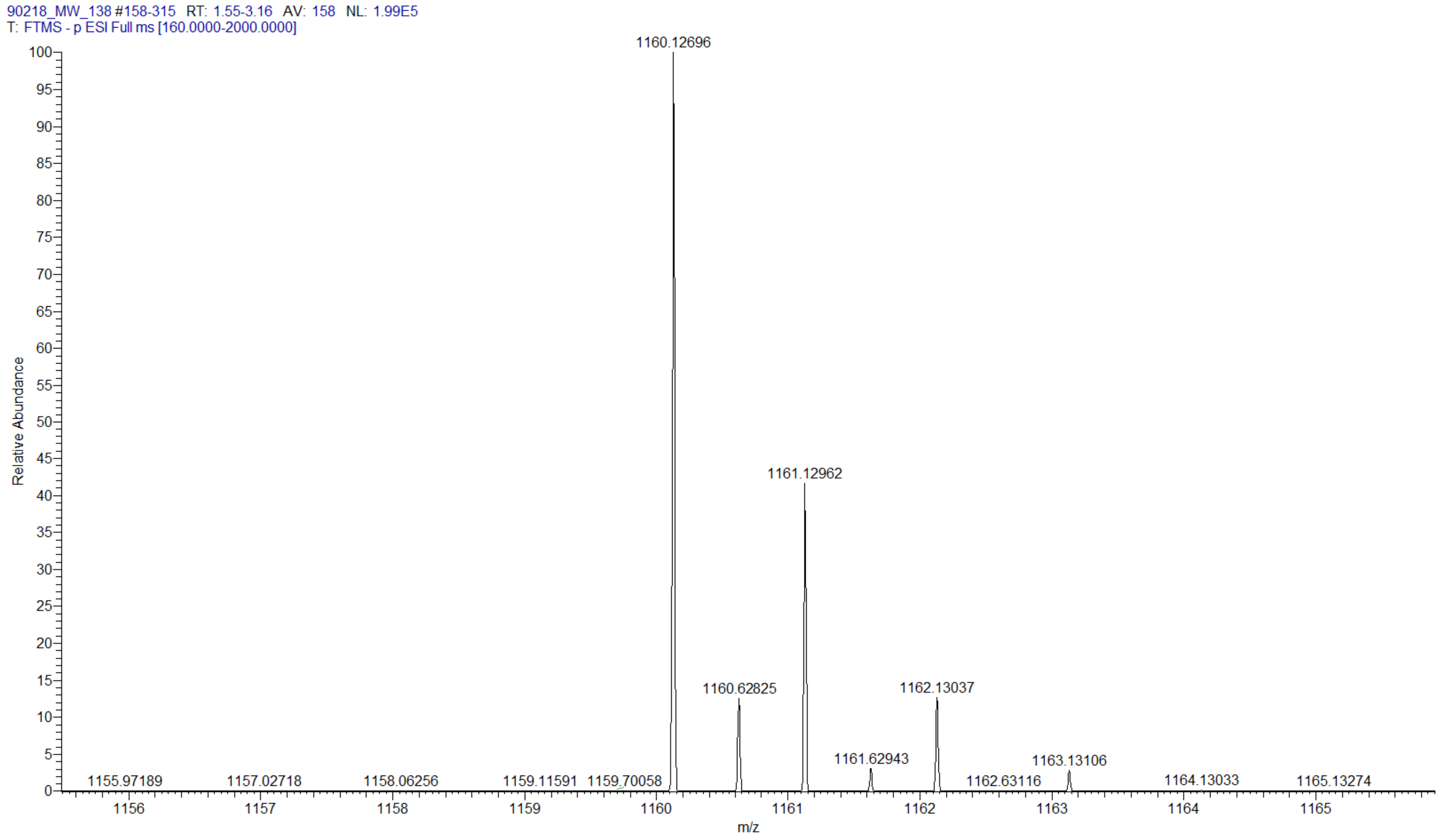

^1H NMR (500 MHz, D_2O, 25°C)

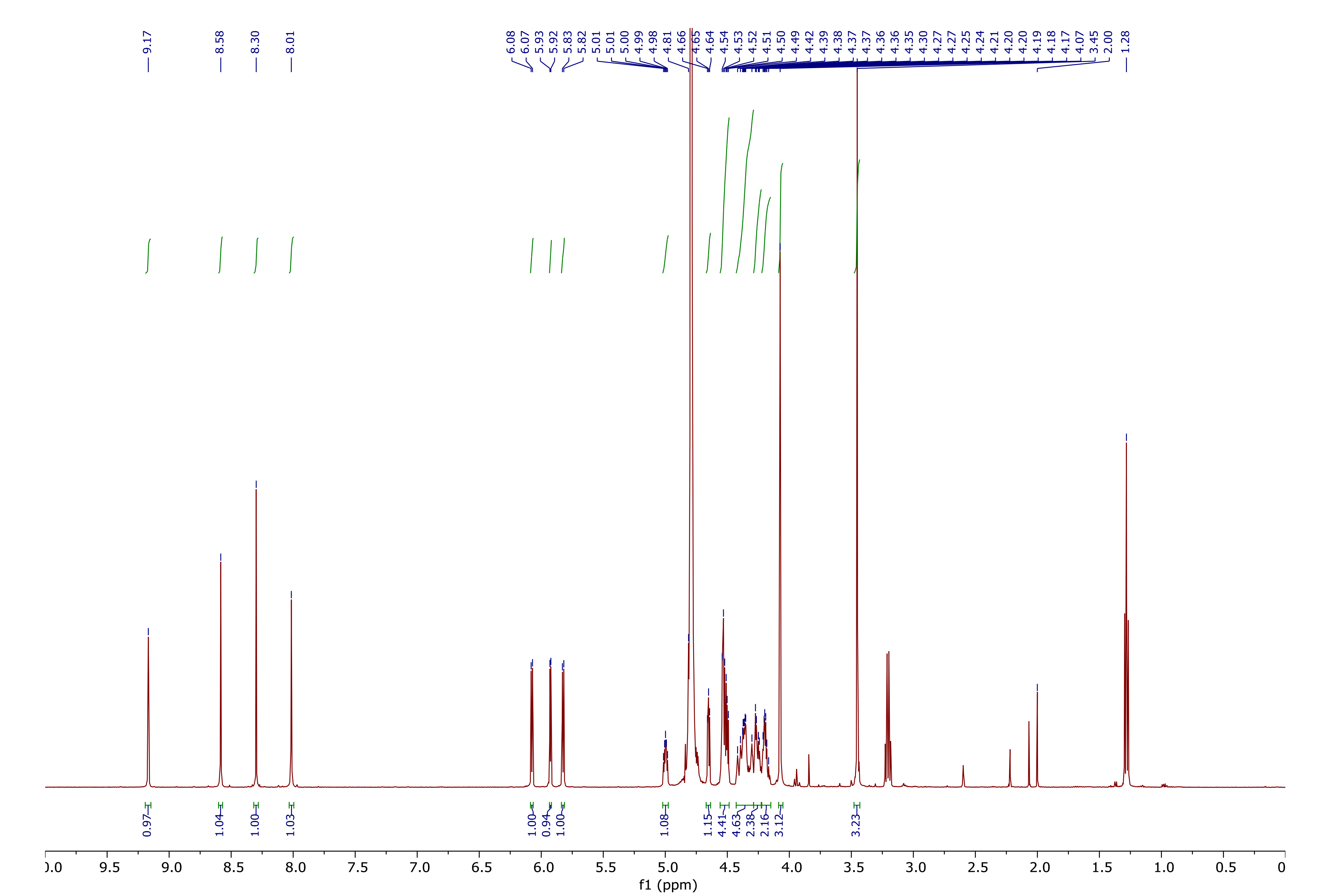

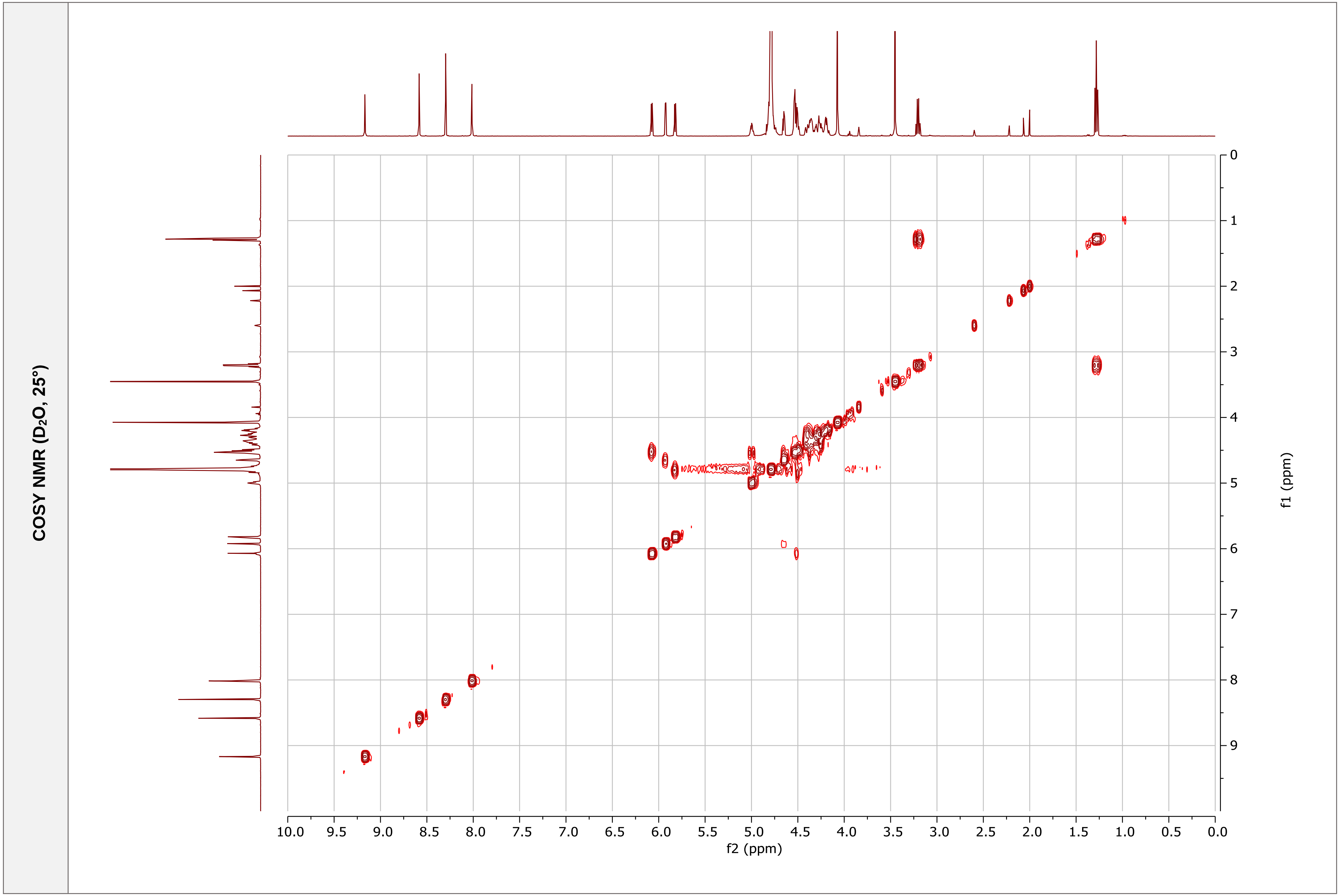

COSY NMR (D_2O, 25°)
f2 (ppm)
f1 (ppm)
10.0 9.5 9.0 8.5 8.0 7.5 7.0 6.5 6.0 5.5 5.0 4.5 4.0 3.5 3.0 2.5 2.0 1.5 1.0 0.5 0.0
0 1 2 3 4 5 6 7 8 9

^{31}P NMR (202.5 MHz, D_2O, 25°C)

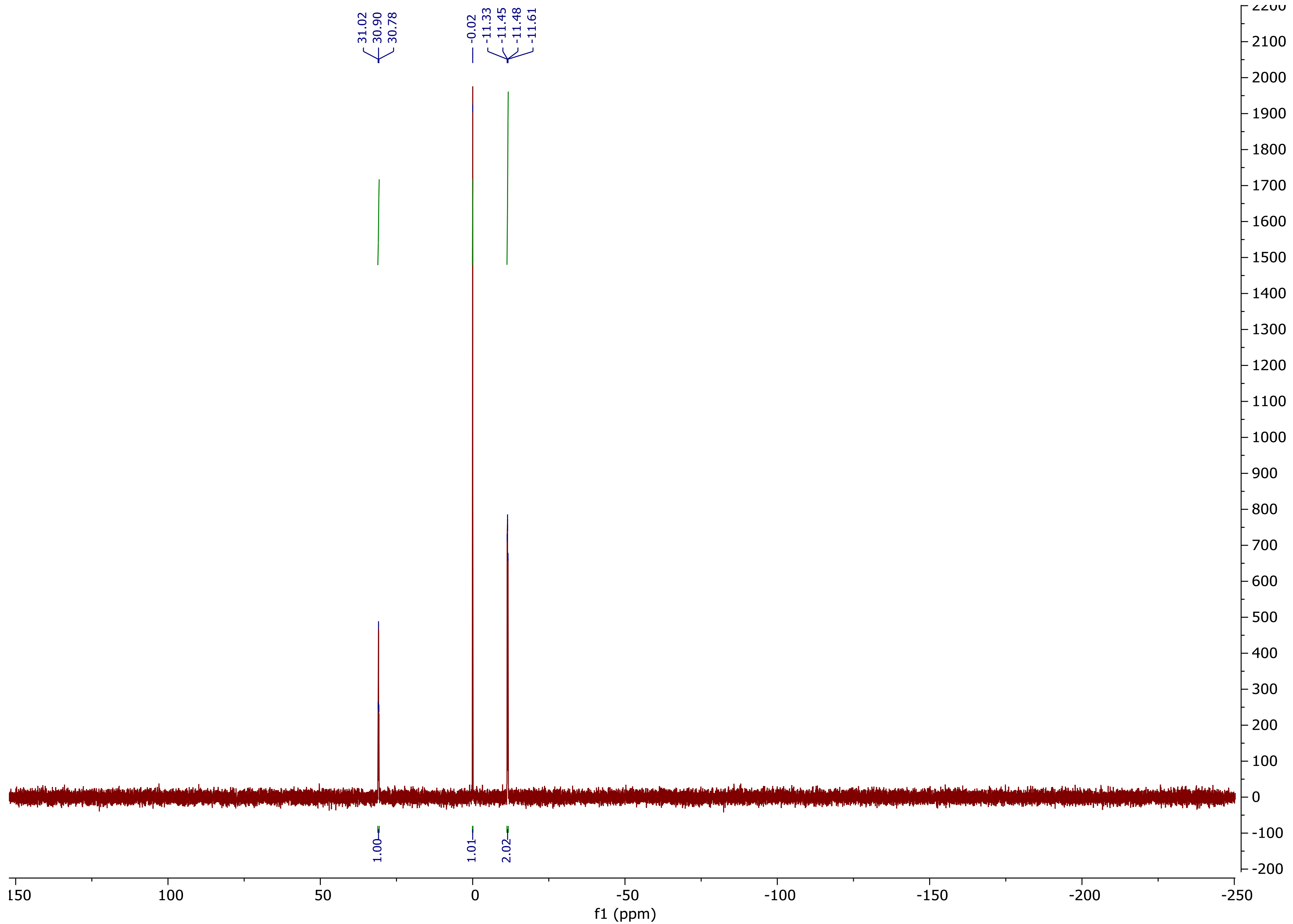

^1H-^{31}P HSQC (D_2O, 25°C)

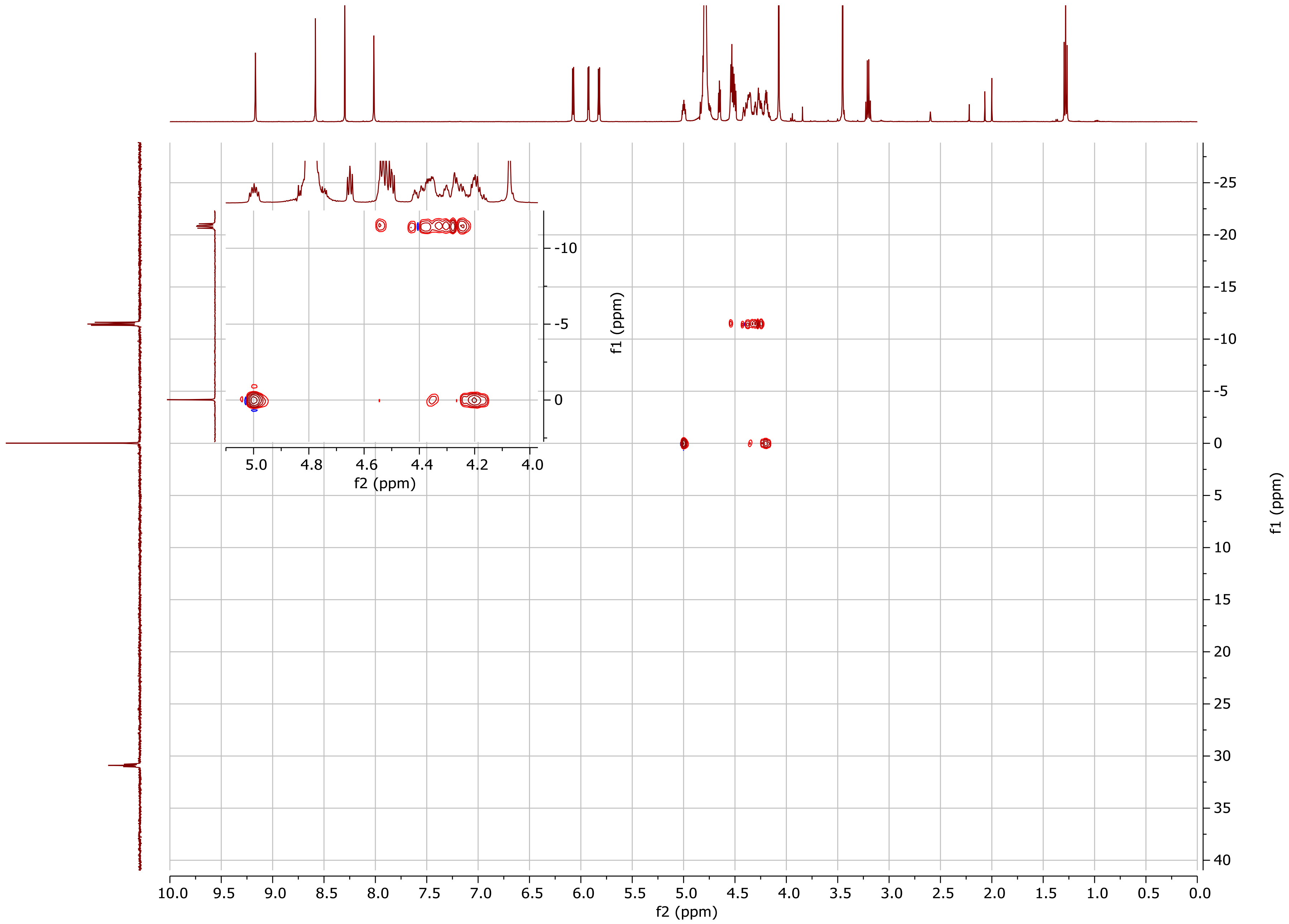

(5) m^{7}Gpp$_S$p^{m6}A$_m$pG R_P

Chemical structure

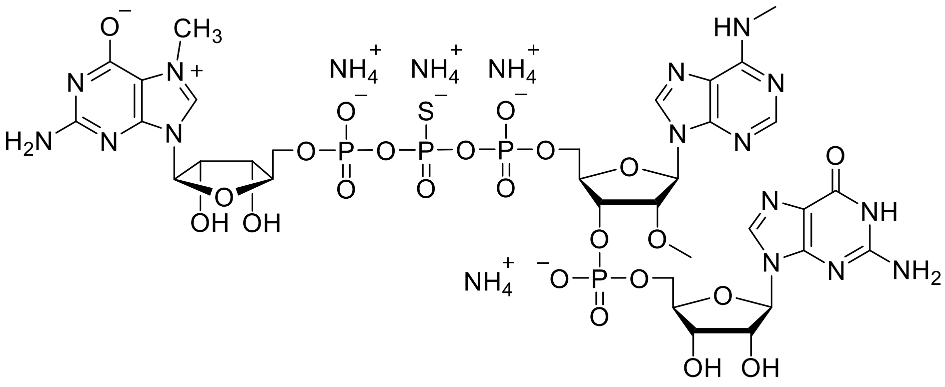

RP HPLC
Abs. @ 254 nm

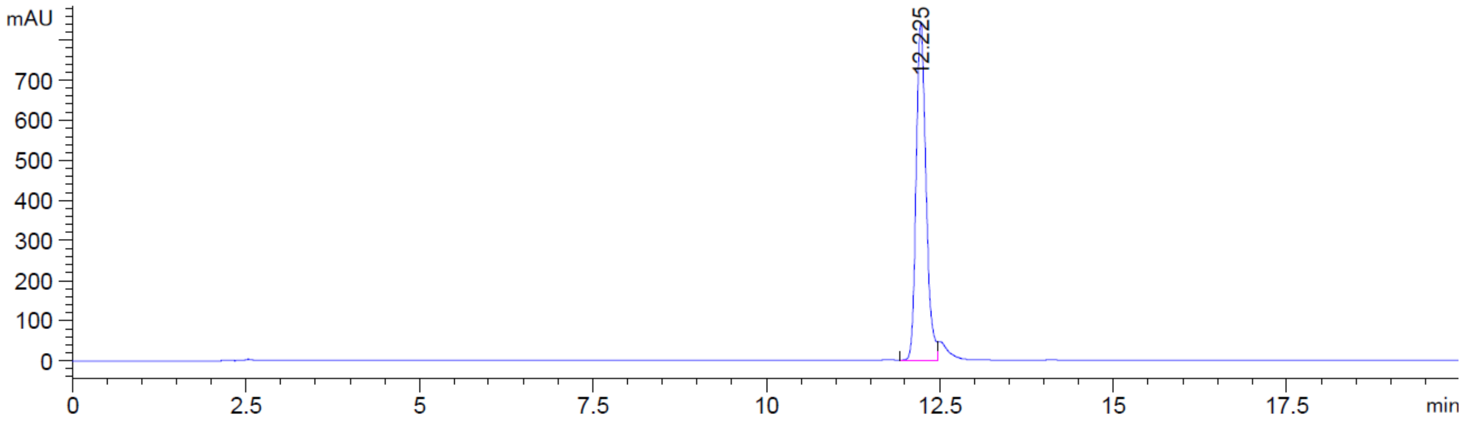

MS (-) ESI

(Calc. $[M-H]^-$ $C_{33}H_{44}N_{15}O_{23}P_4S^-$ 1174.14111)

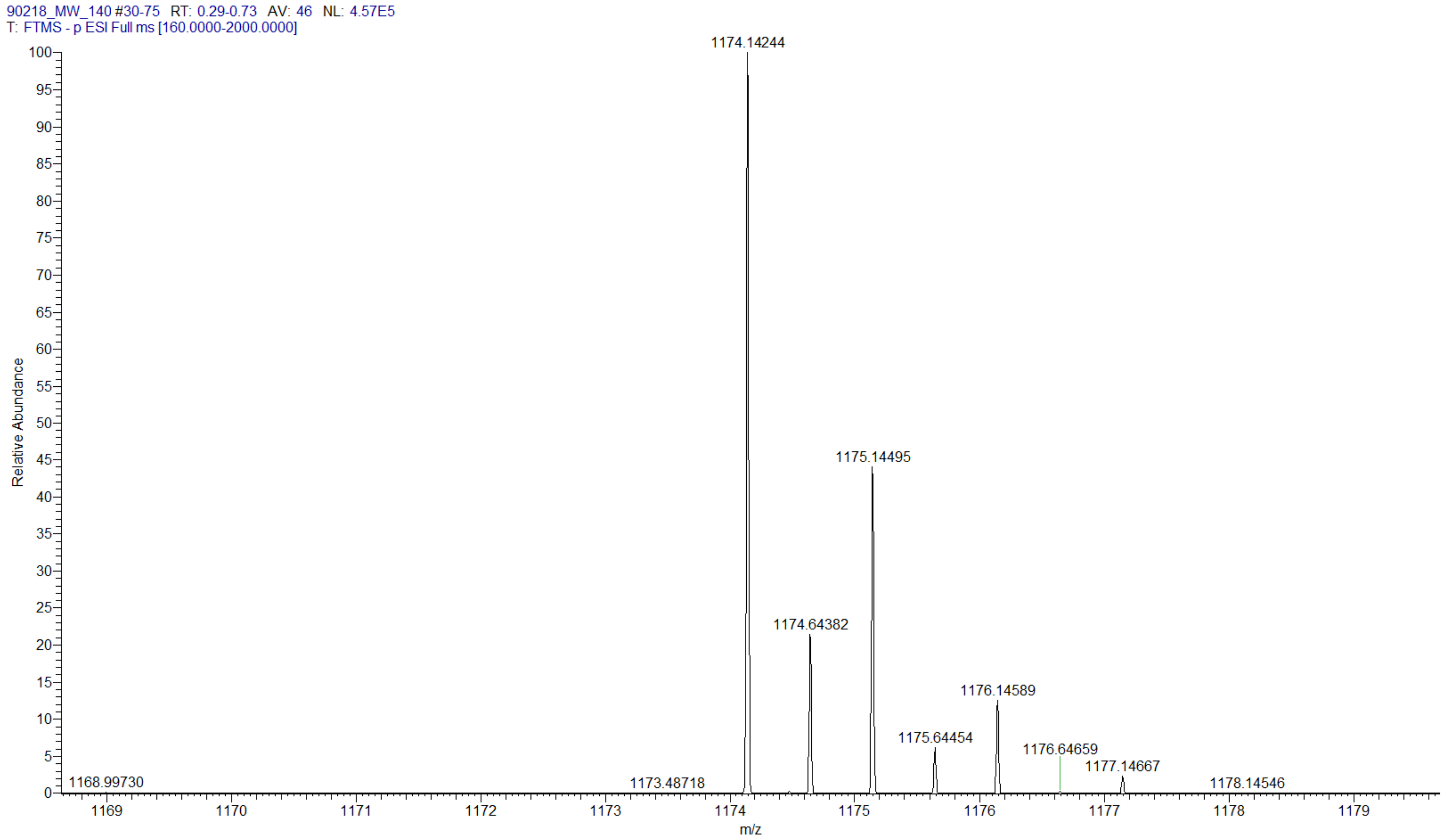

^1H NMR (500 MHz, D_2O, 25°C)

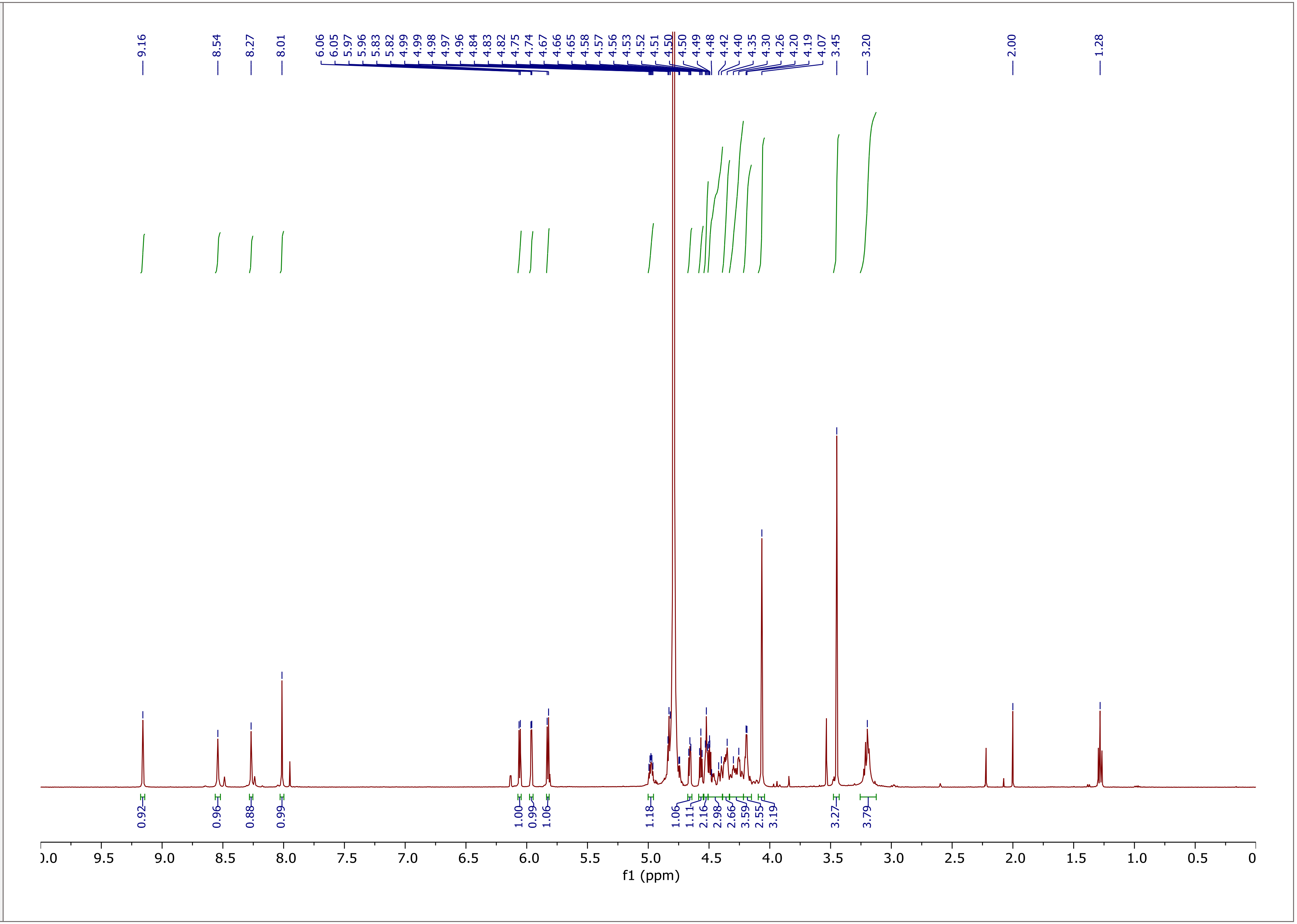

COSY NMR (D_2O, 25°)

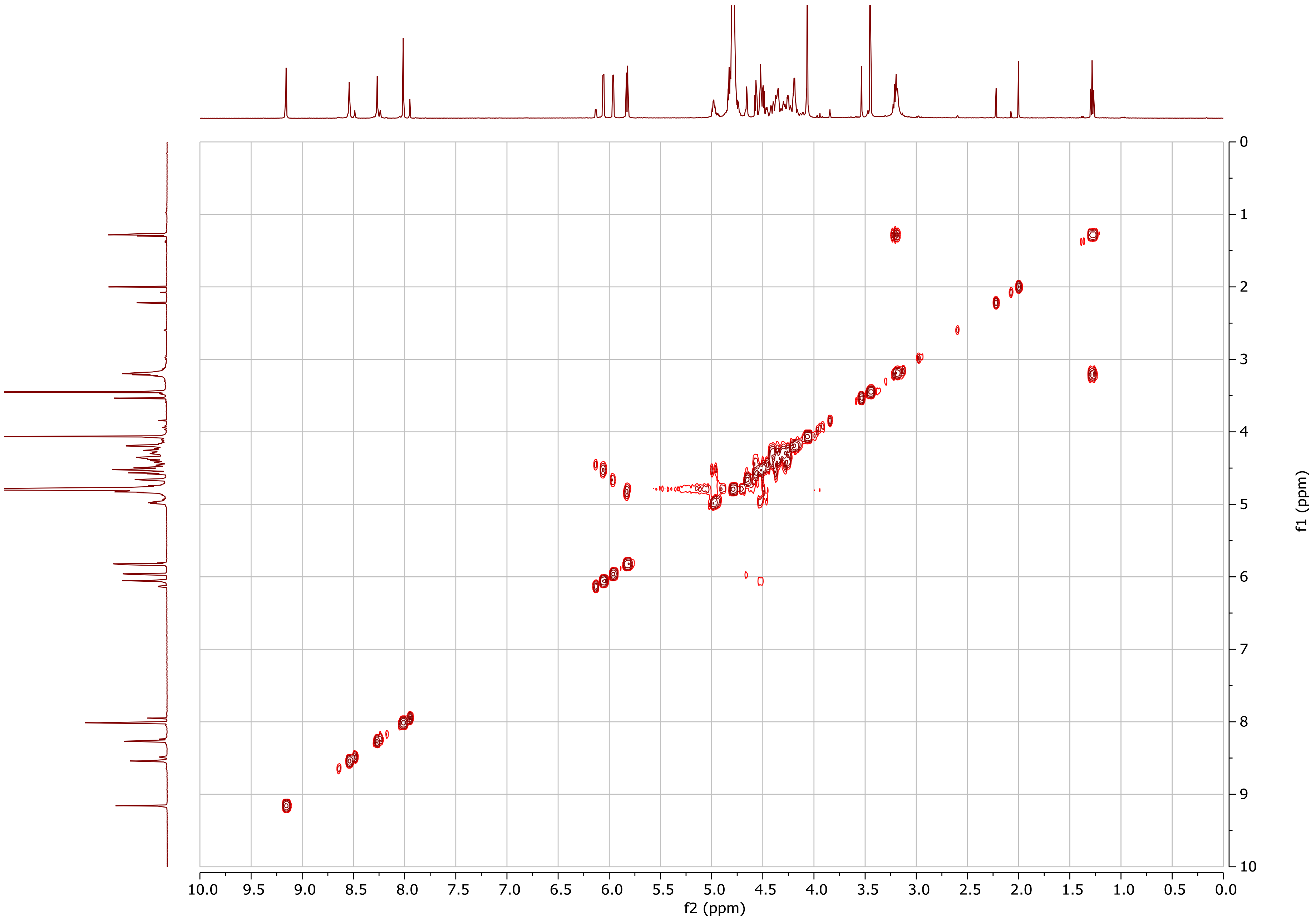

^{31}P NMR (202.5 MHz, D_2O, 25°C)

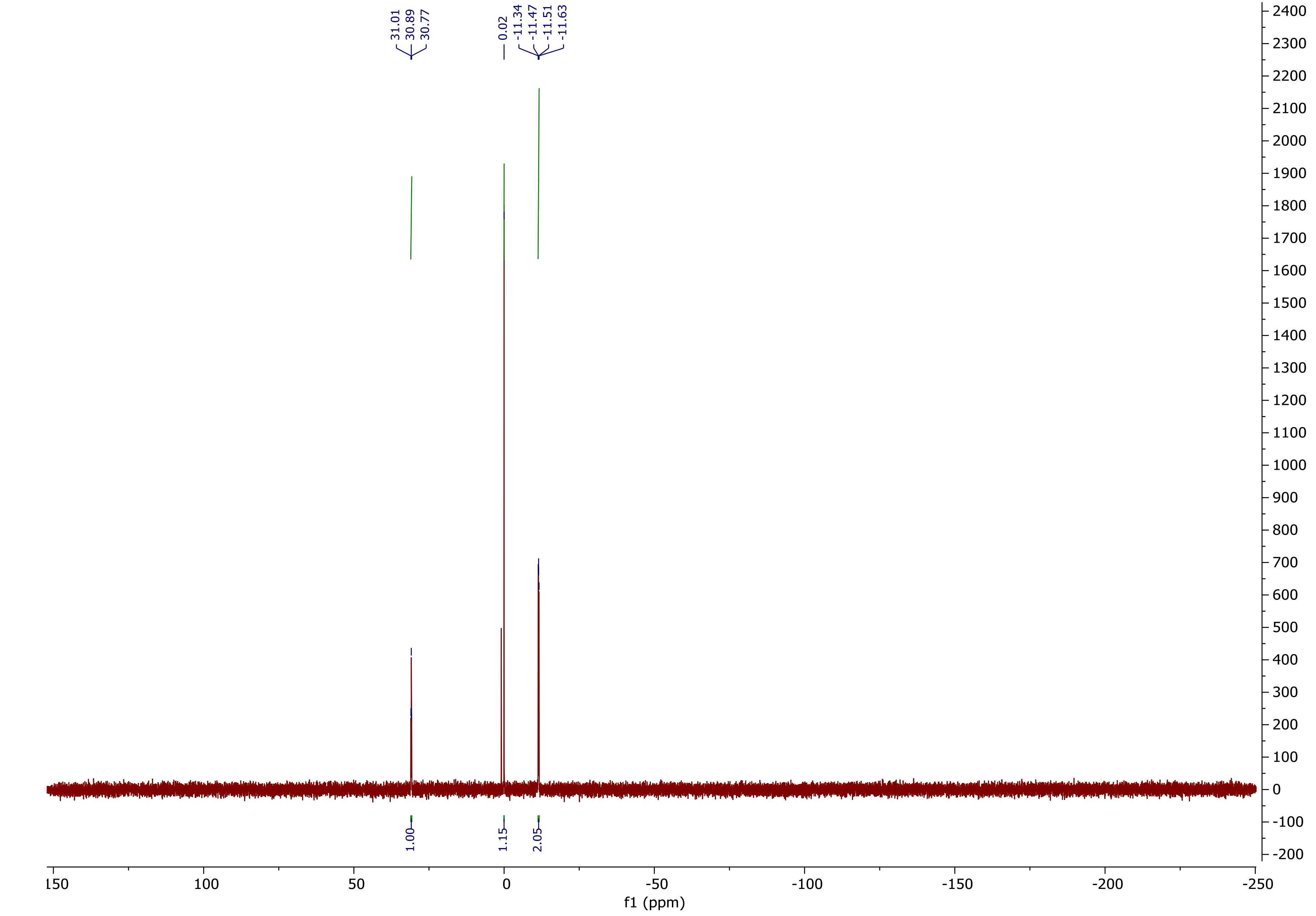

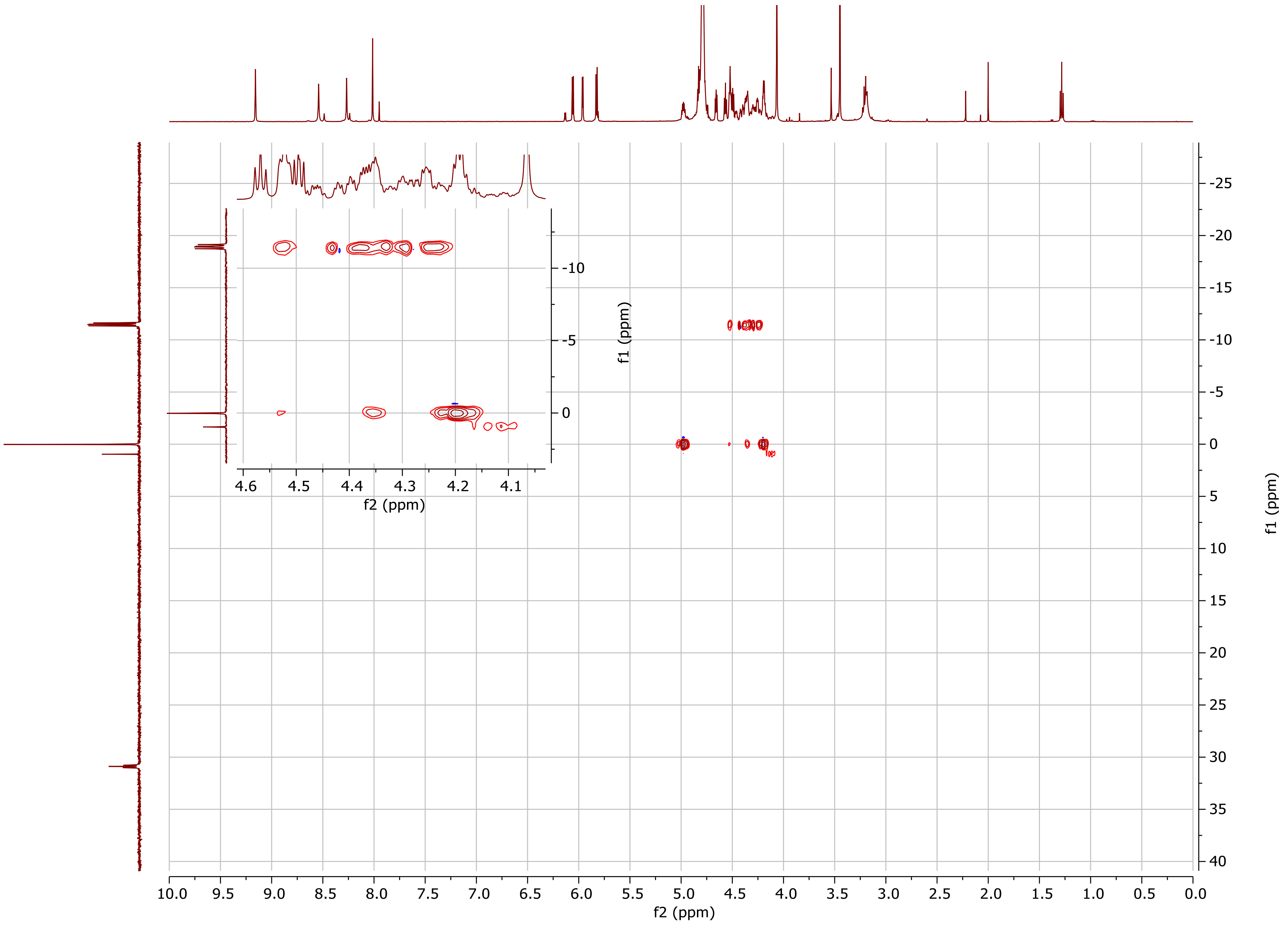

^{1}H-^{31}P HSQC (D_2O, 25°C)

(6) $m^7Gpp_Sp^{m6}A_mpG$ S_P	
**Chemical structure**	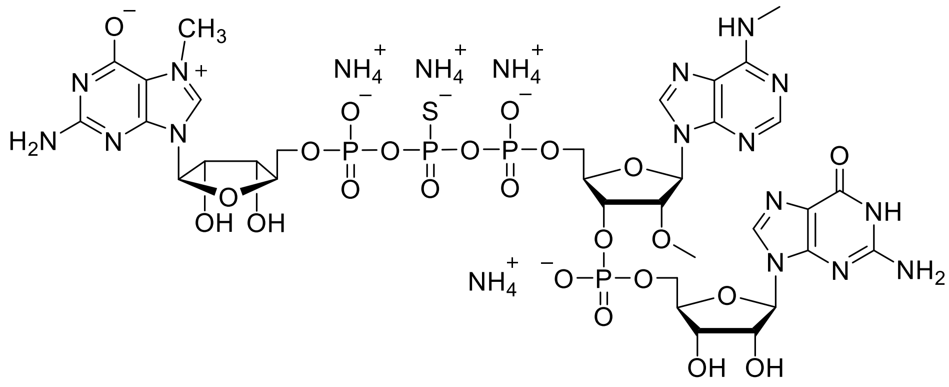
**RP HPLC** Abs. @ 254 nm	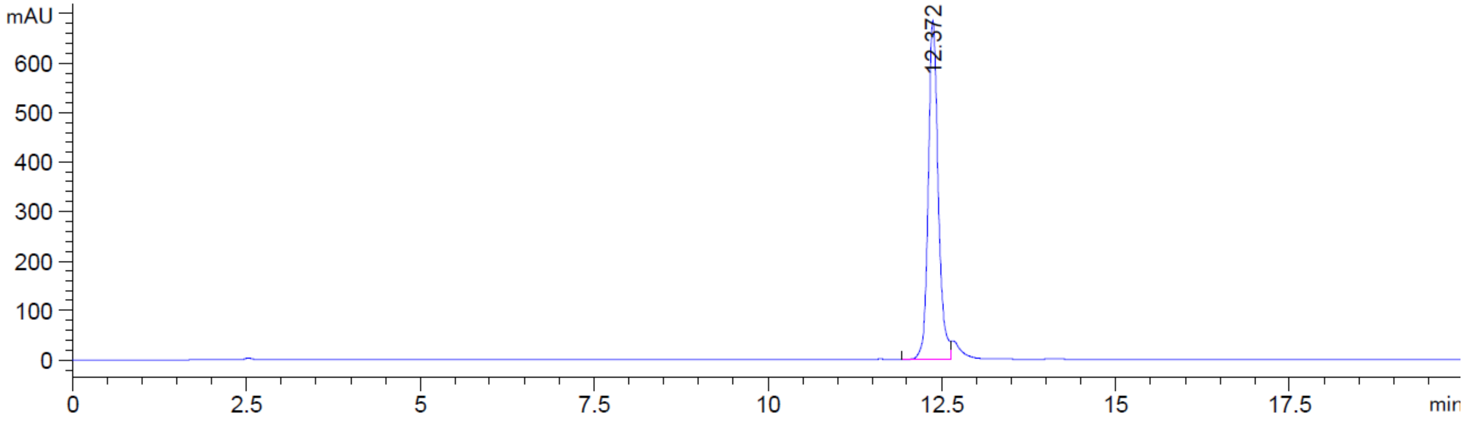

MS (-) ESI

(Calc. $[M-H]^- C_{33}H_{44}N_{15}O_{23}P_4S^-$ 1174.14111)

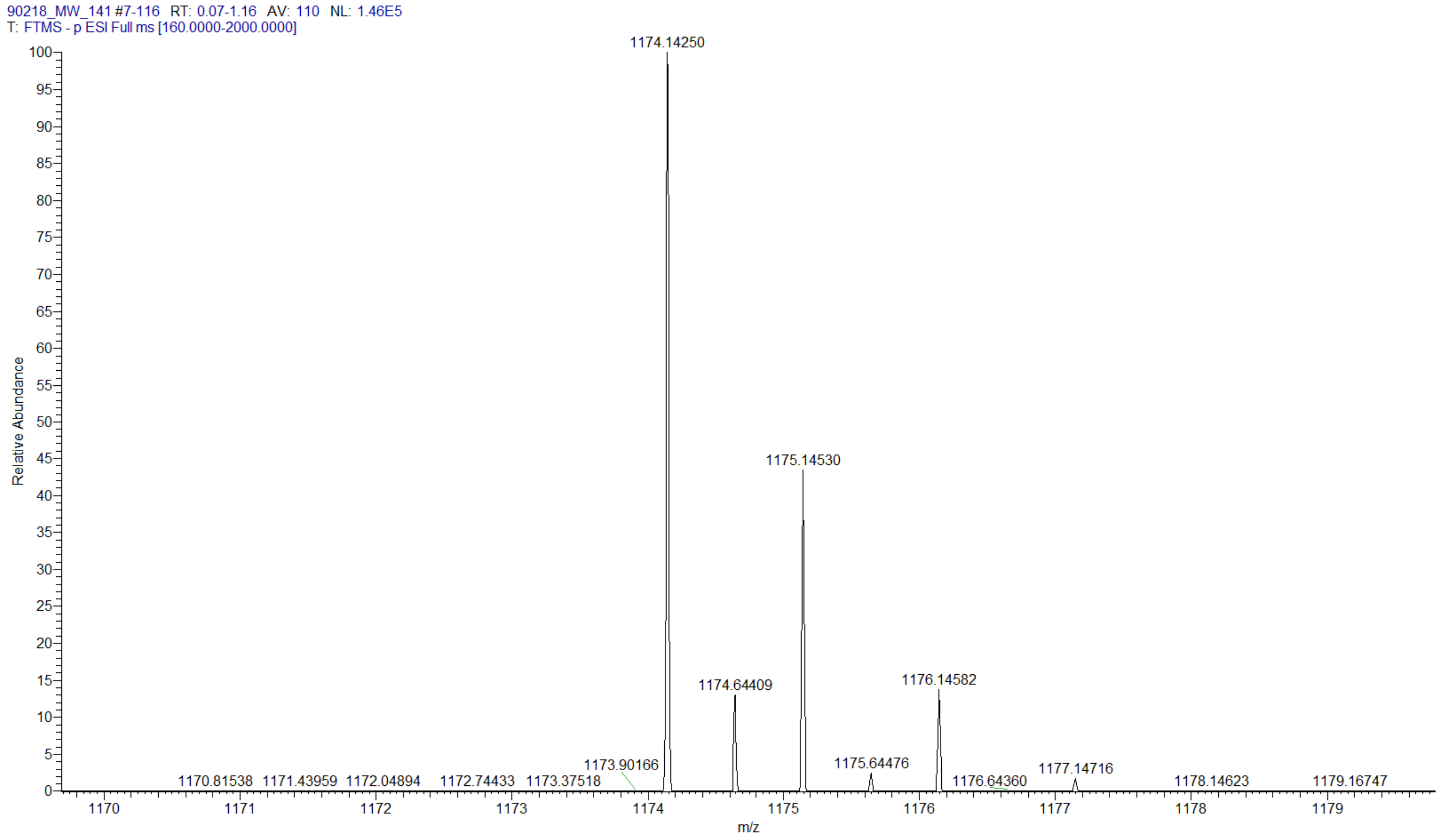

^1H NMR (500 MHz, D_2O, 25°C)

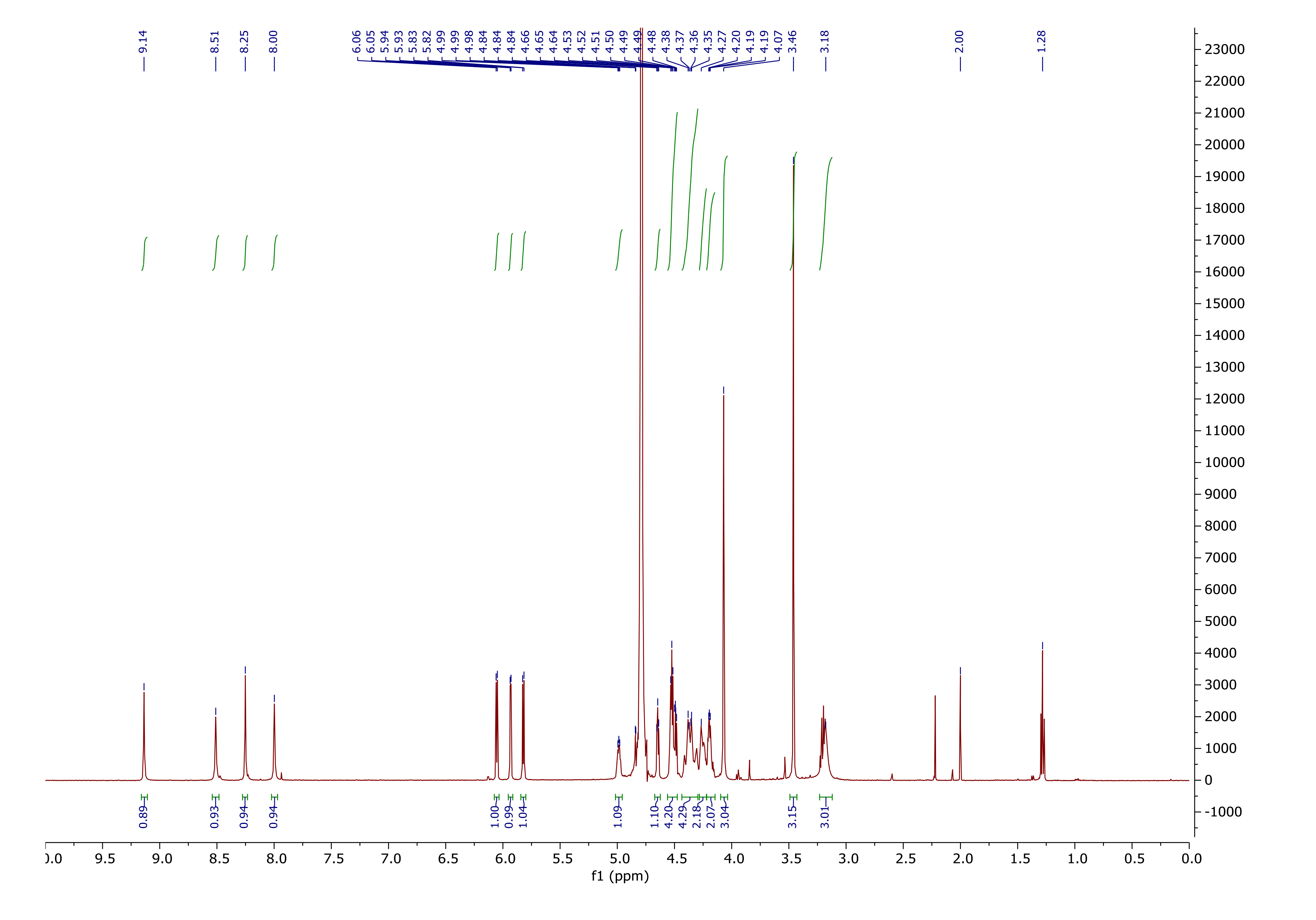

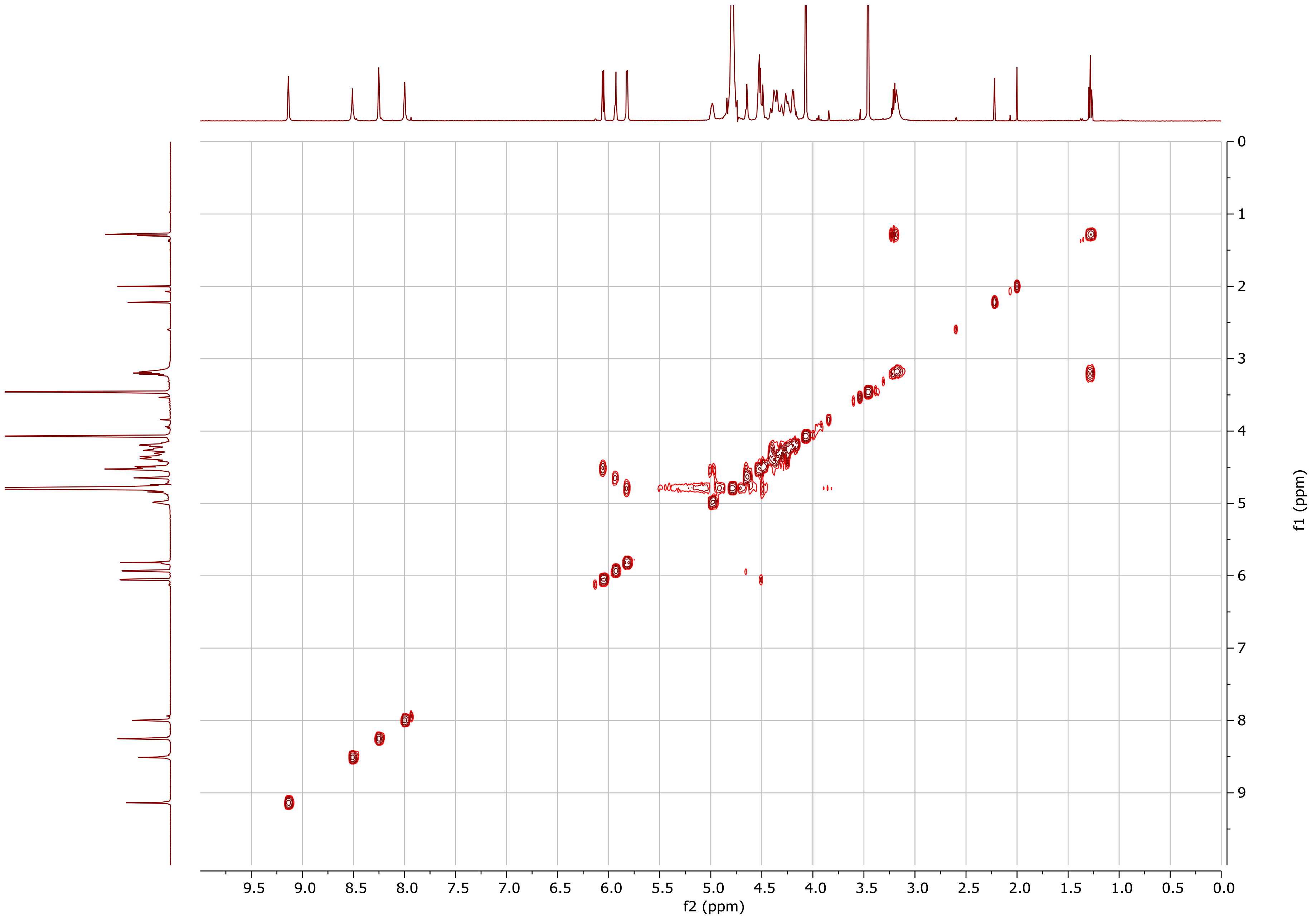

COSY NMR (D_2O, 25°)

^{31}P NMR (202.5 MHz, D_2O, 25°C)

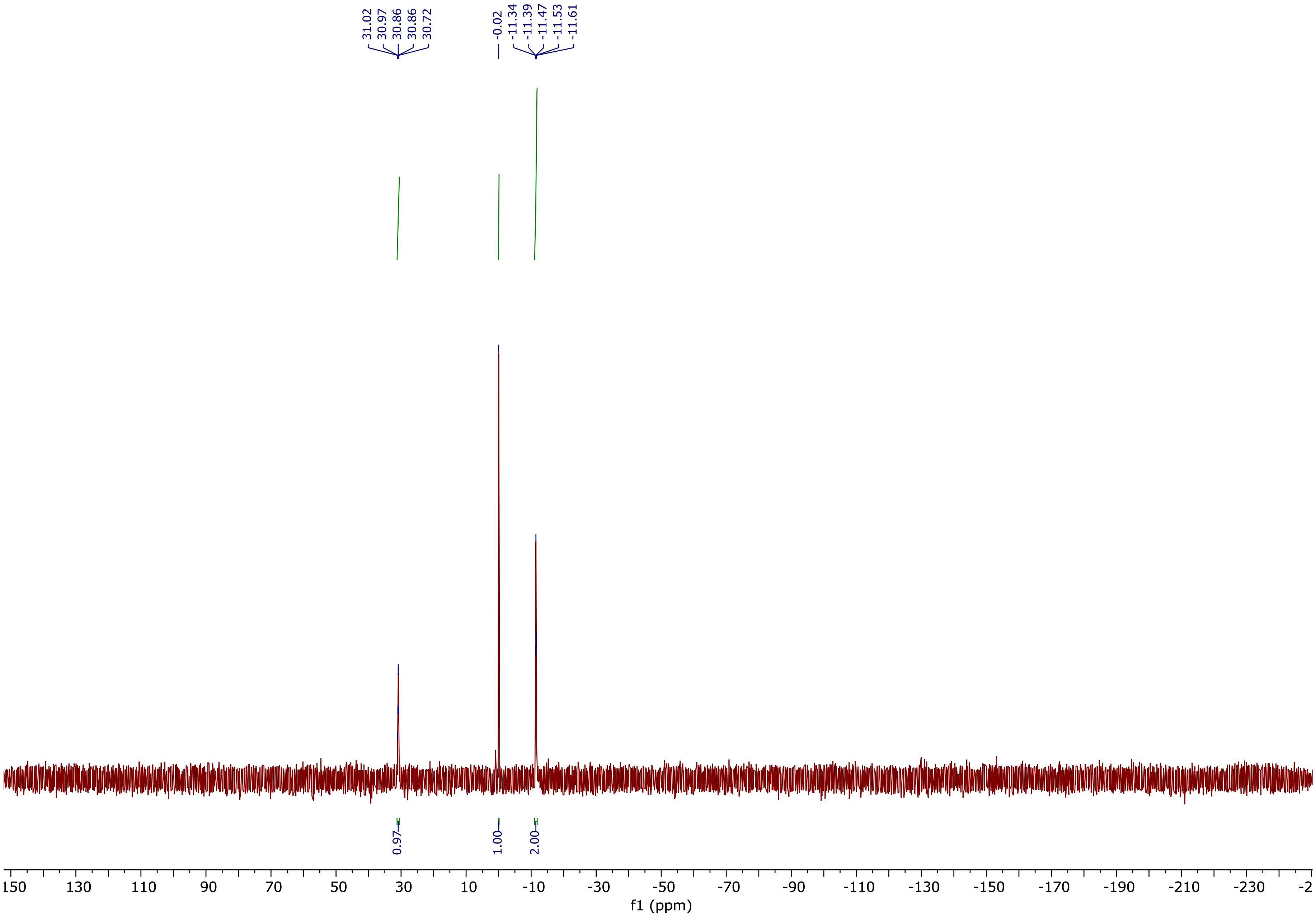

^{1}H-^{31}P HSQC (D_2O, 25°C)

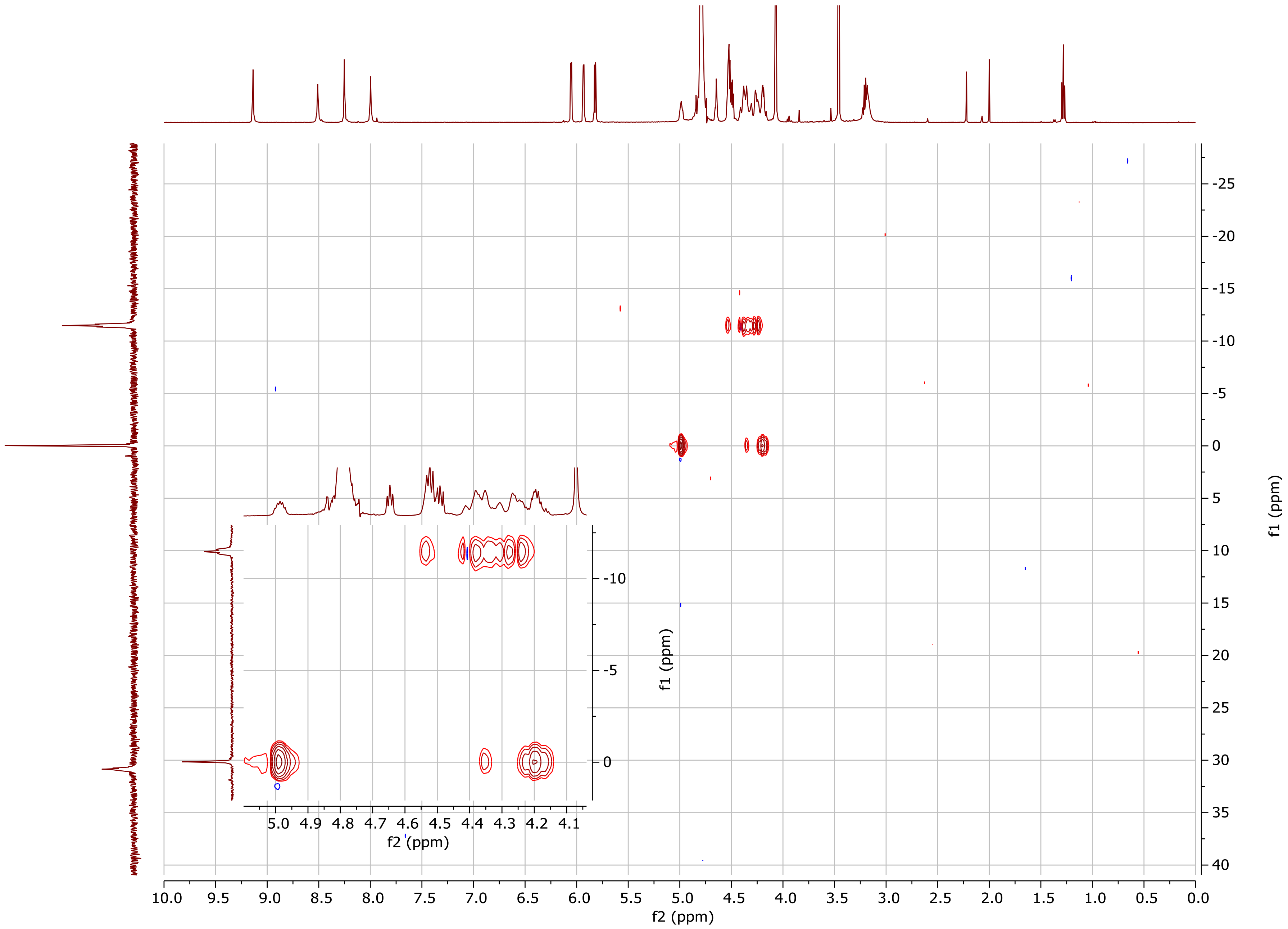

(7) m^{7}Gppp$^{5'S}$ApG

Chemical structure

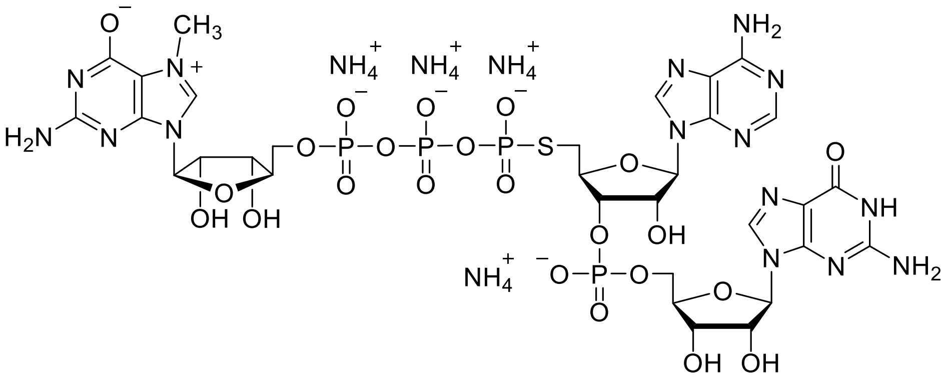

RP HPLC
Abs. @ 254 nm

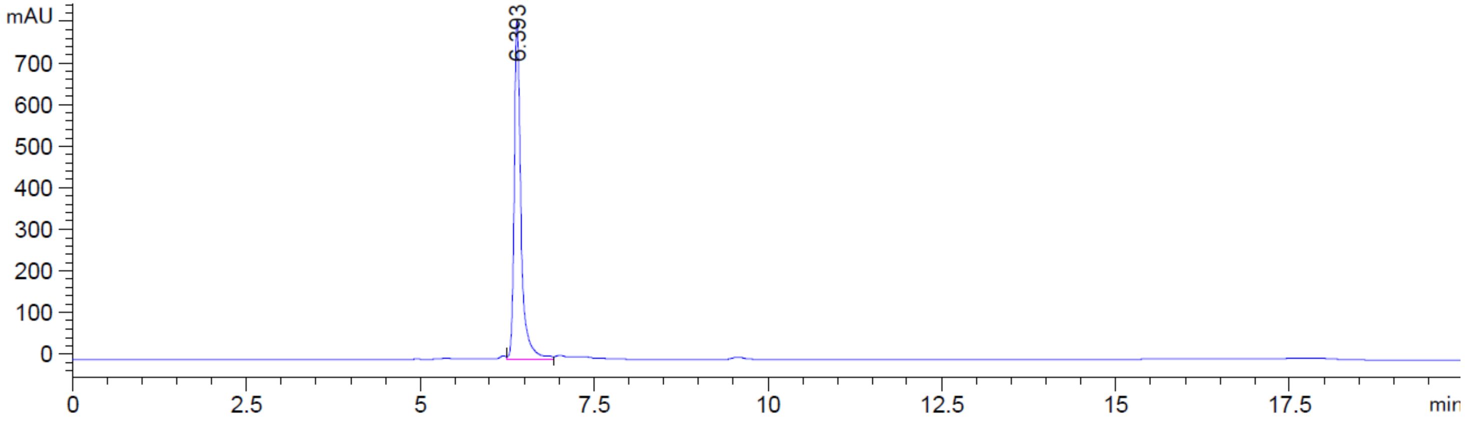

MS (-) ESI

(Calc. $[M-H]^-$ $C_{31}H_{40}N_{15}O_{23}P_4S^-$ 1146.10981)

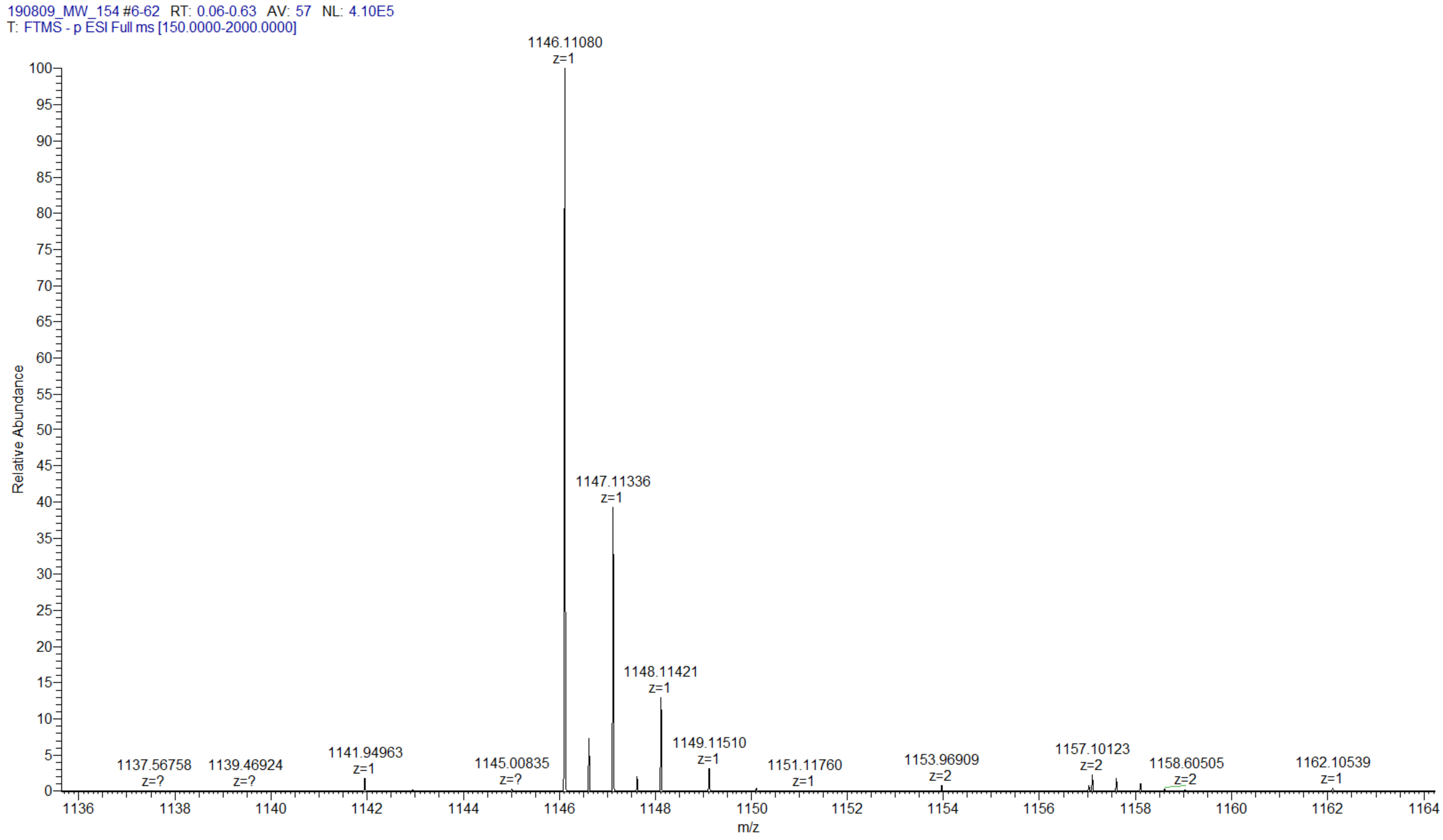

(8) m^{7}Gppp$^{5'S}$A$_m$pG	
**Chemical structure**	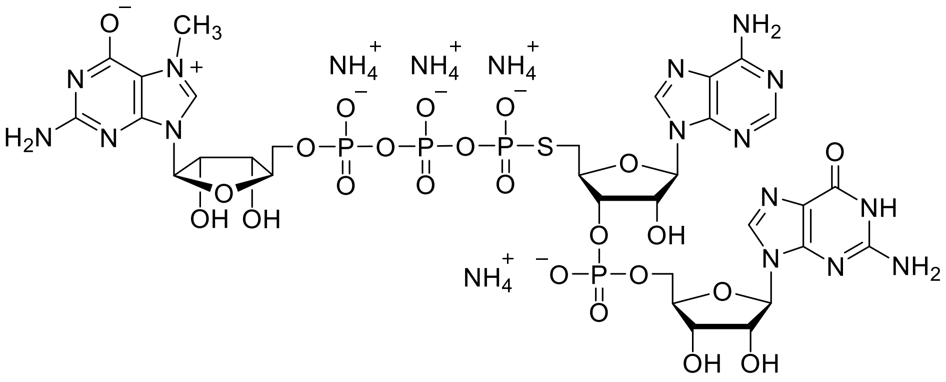
**RP HPLC** Abs. @ 254 nm	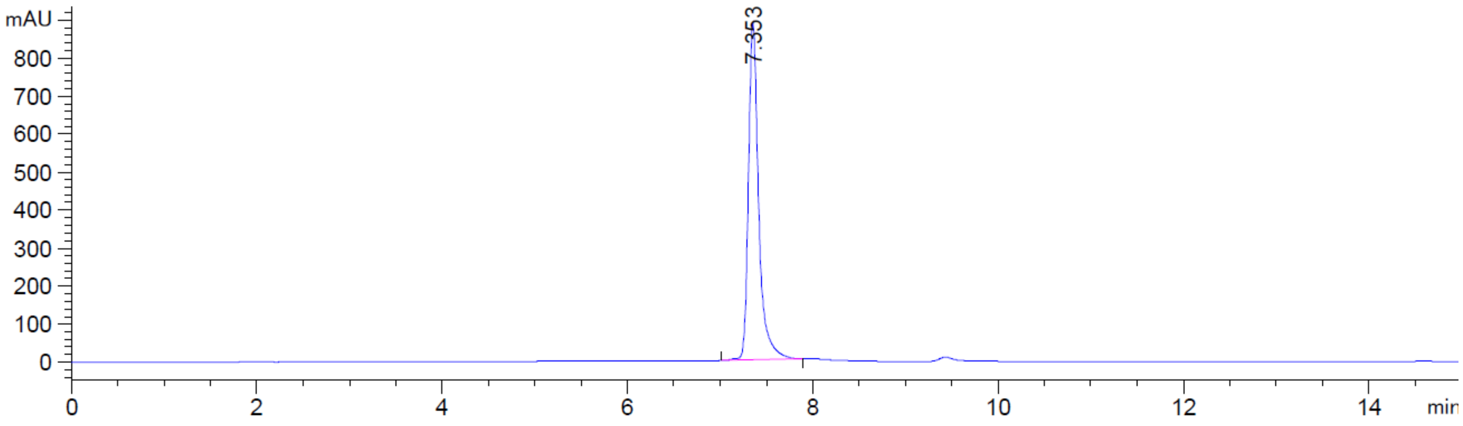

MS (-) ESI

(Calc. $[M-H]^-$ $C_{32}H_{42}N_{15}O_{23}P_4S^-$ 1160.12546)

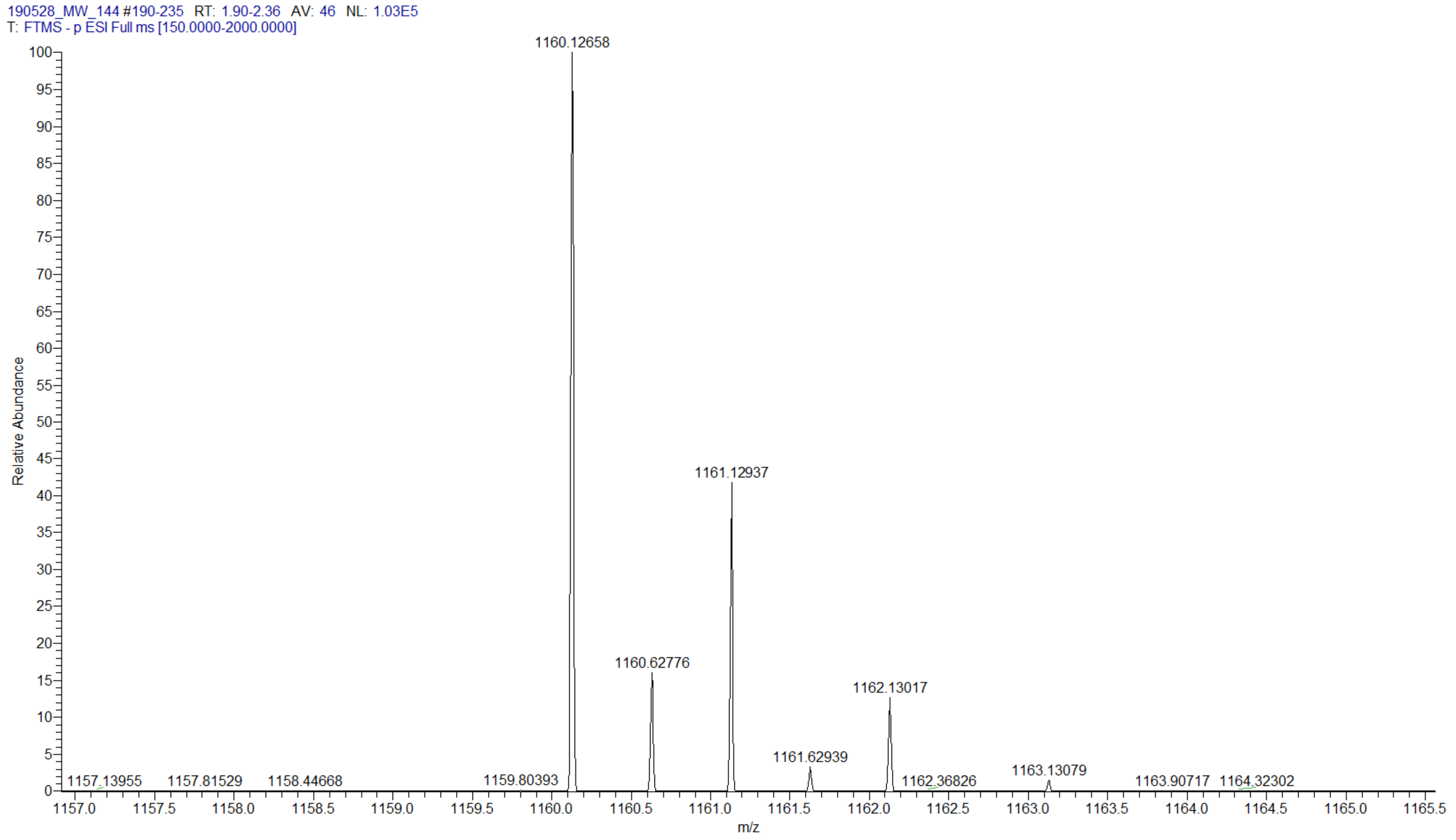

(9) $m^7GppCH_2pA_mpG$

Chemical structure

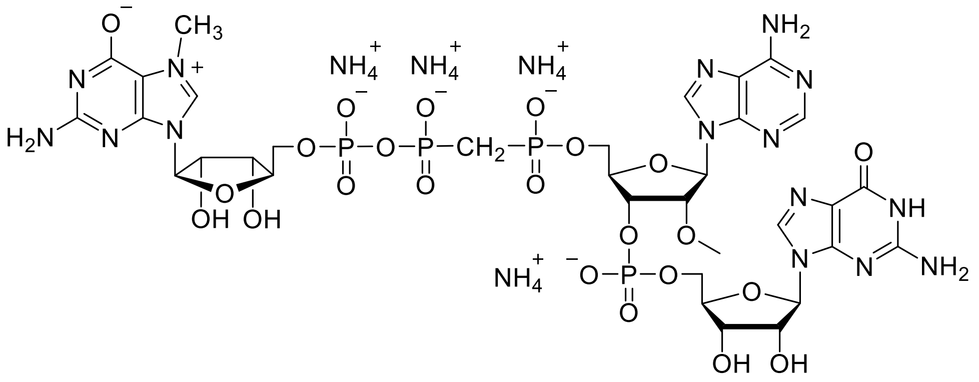

RP HPLC
Abs. @ 254 nm

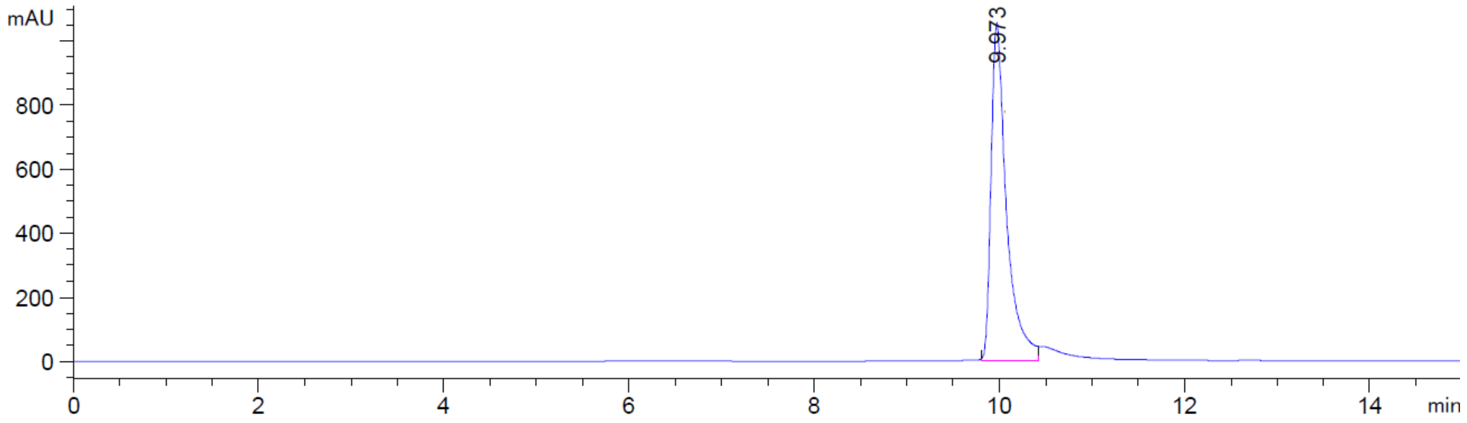

MS (-) ESI

(Calc. $[M-H]^-$ $C_{33}H_{44}N_{15}O_{23}P_4^-$ 1142.16904)

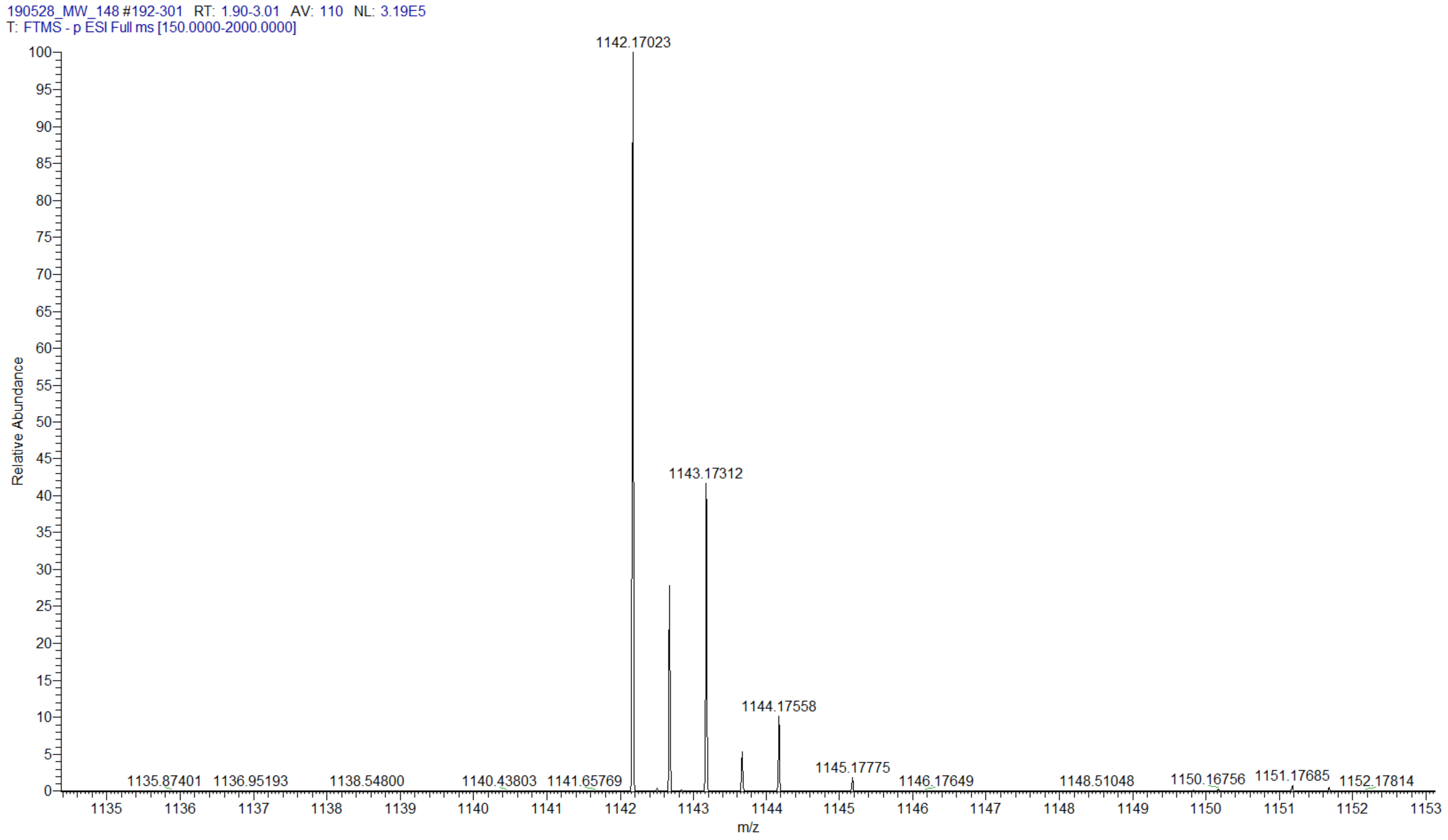

(10) m^{7}GppppApG

Chemical structure

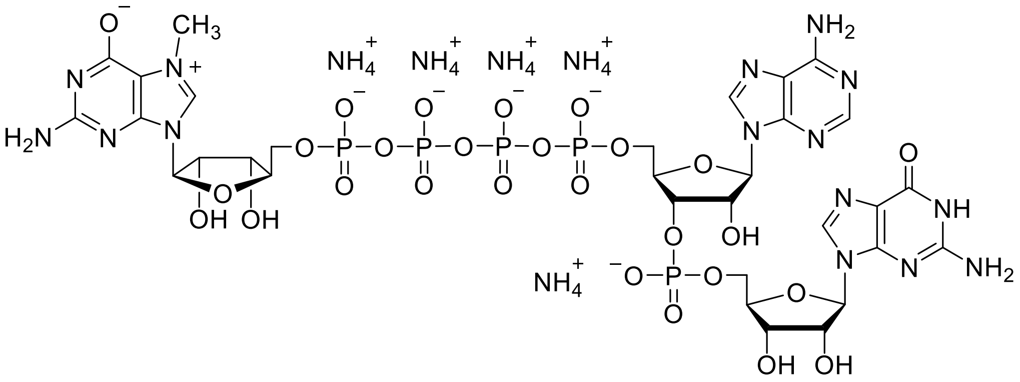

RP HPLC
Abs. @ 254 nm

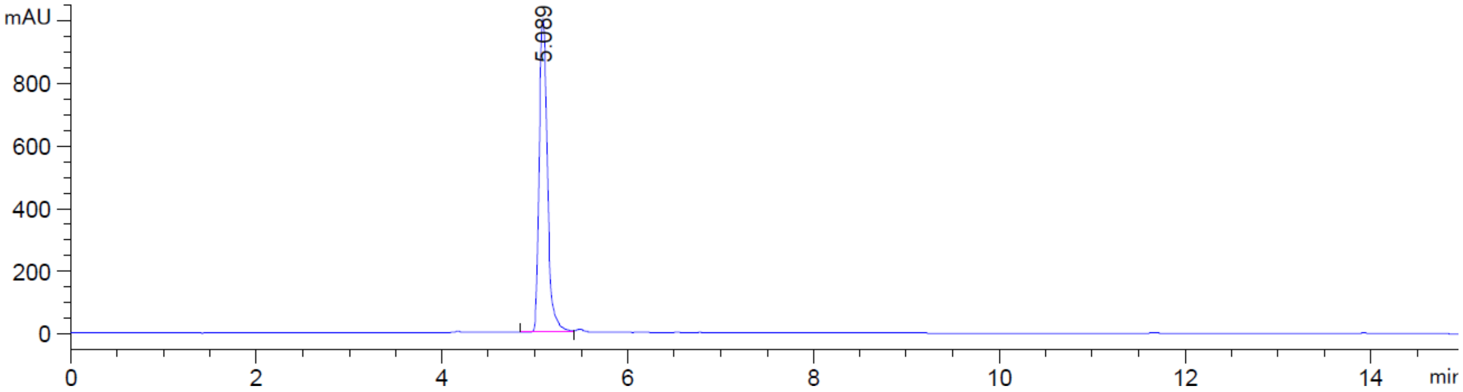

MS (-) ESI

(Calc. $[M-H]^-$ $C_{31}H_{41}N_{15}O_{27}P_5^-$ 1210.09899)

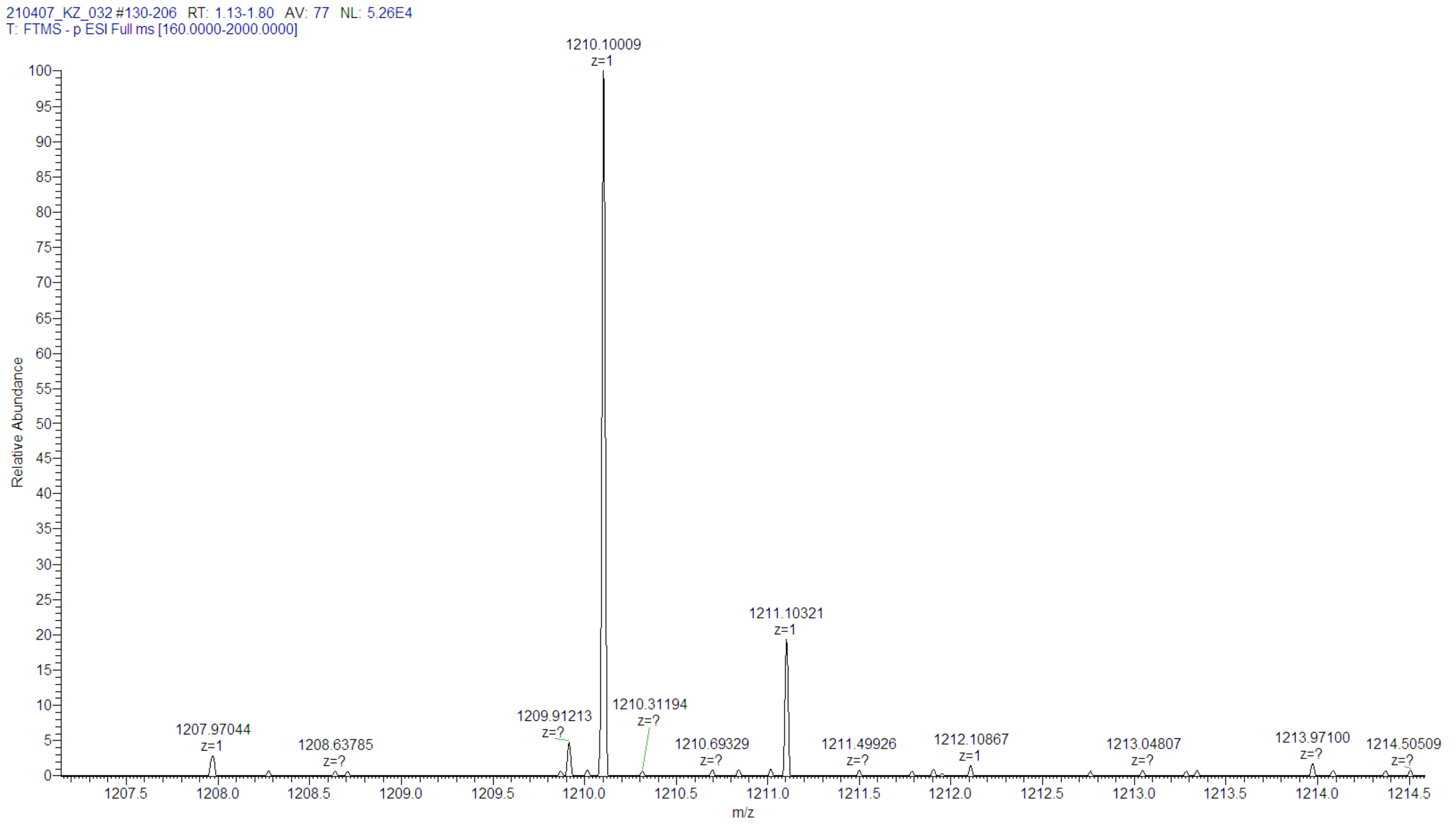

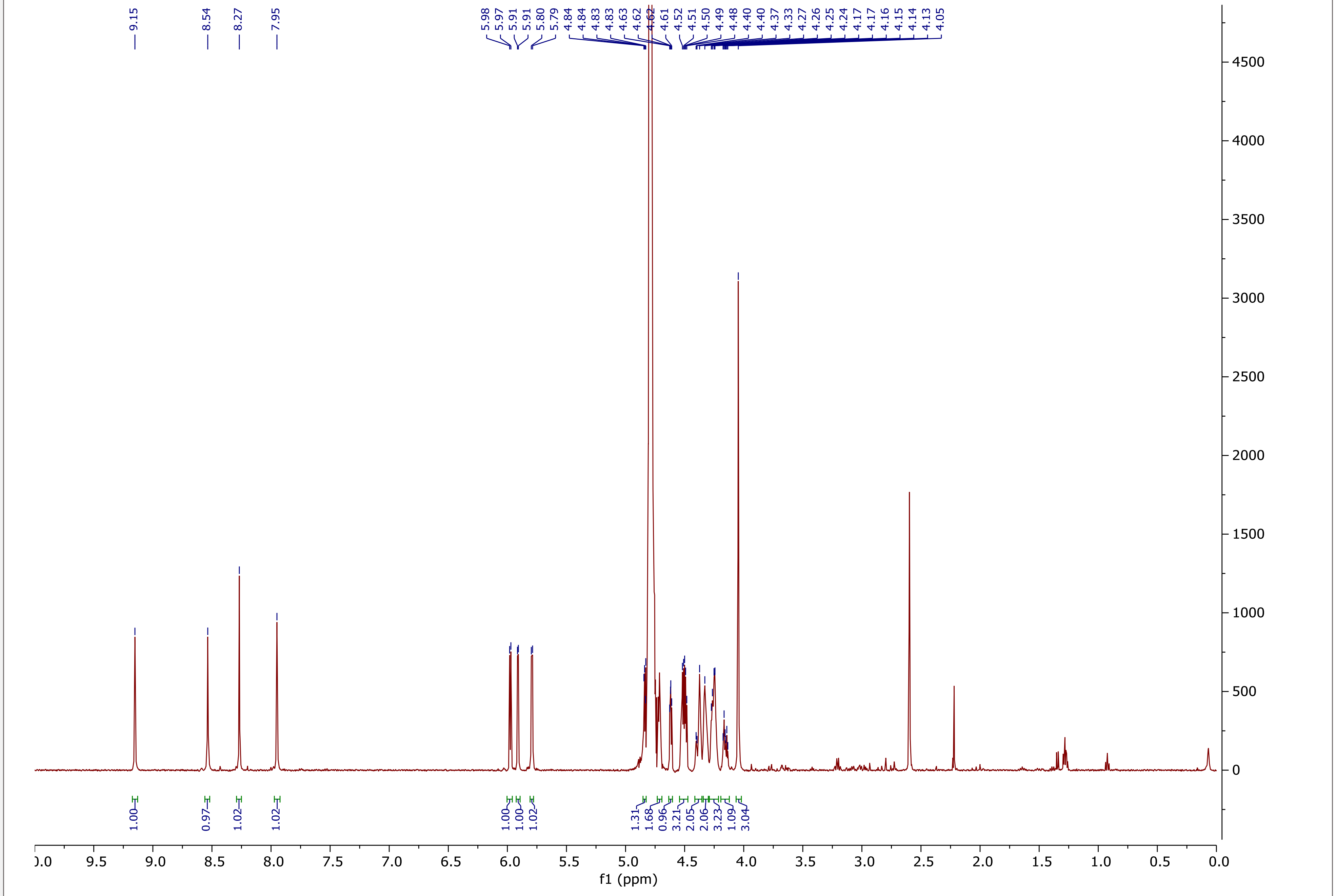

^{1}H NMR (500 MHz, D_2O, 25°C)

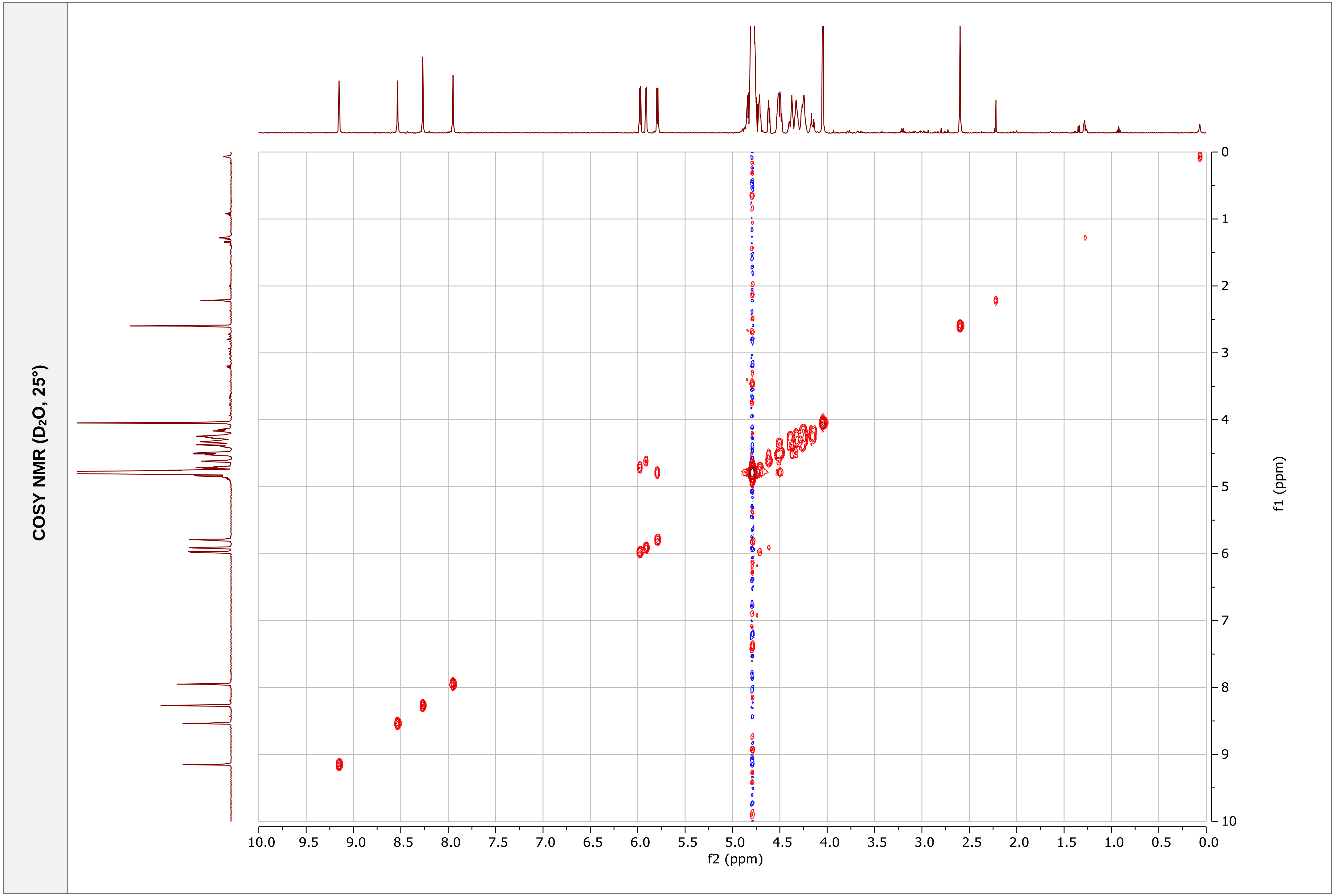
COSY NMR (D_2O, 25°)
f2 (ppm)
f1 (ppm)

^{31}P NMR (202.5 MHz, D_2O, 25°C)

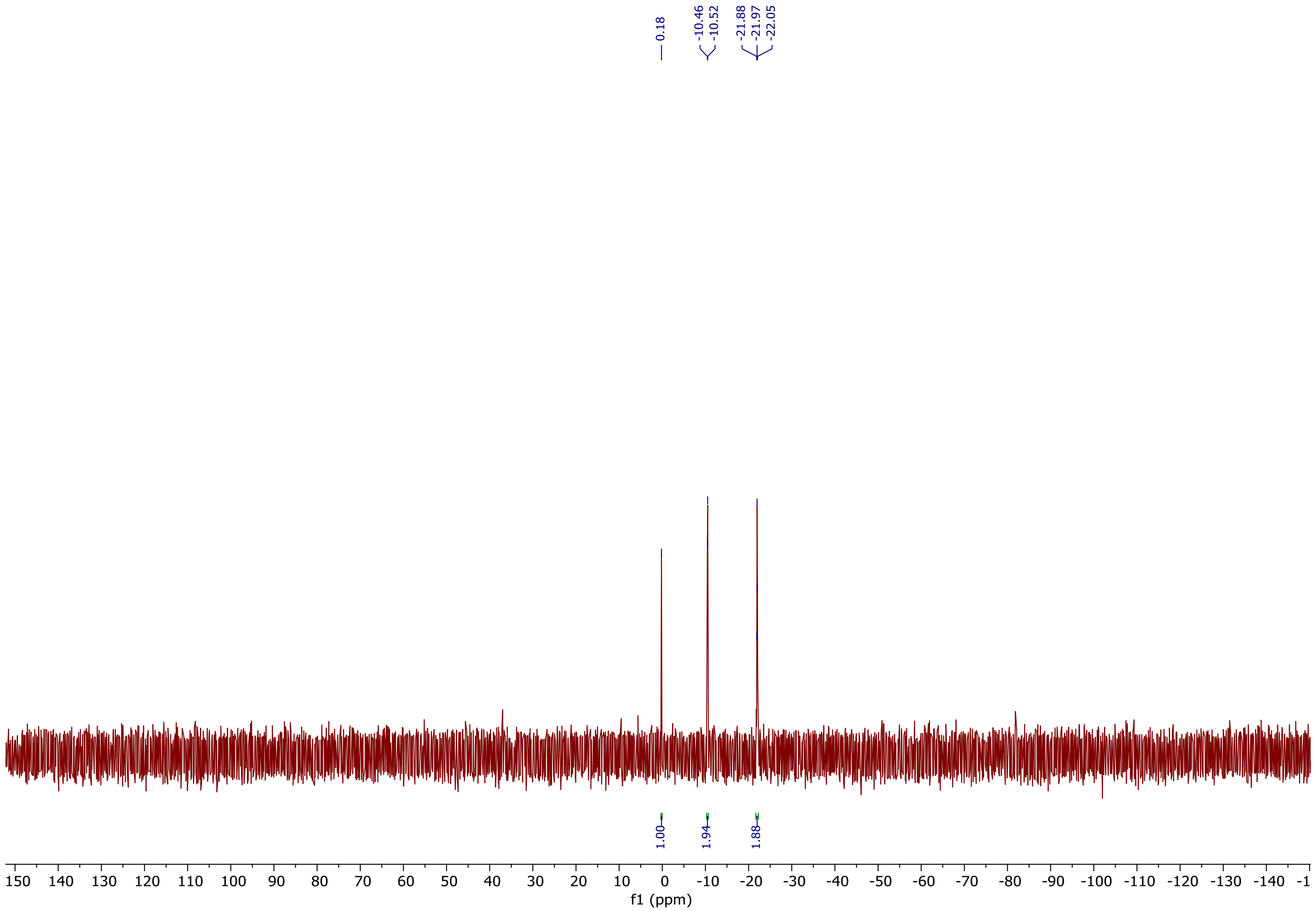

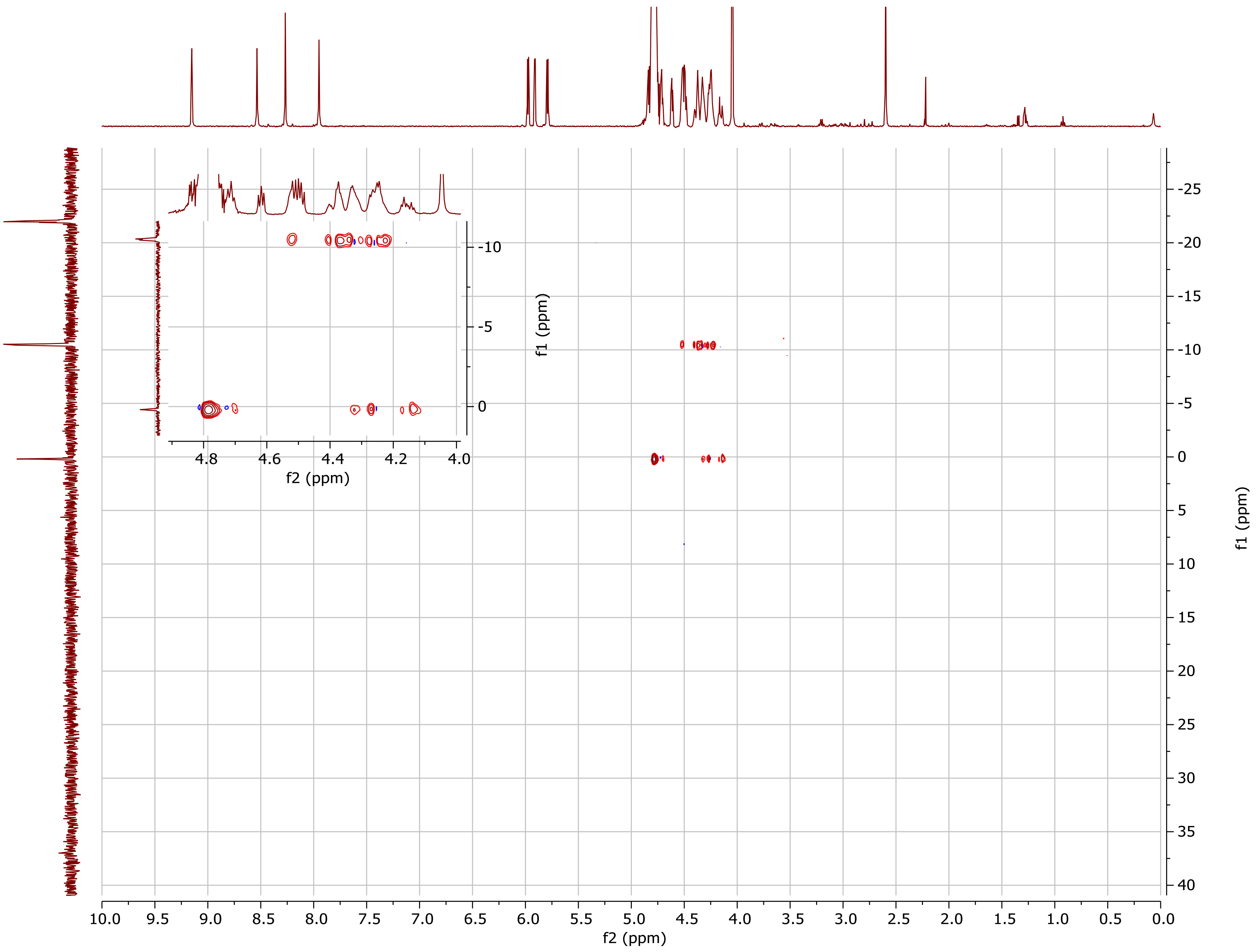

^{1}H-^{31}P HSQC (D_2O, 25°C)

(11) $m^7GppppA_mpG$

Chemical structure

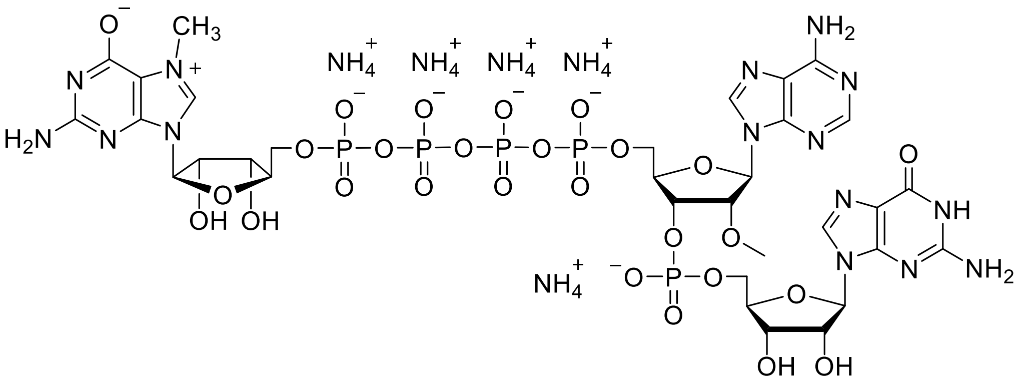

RP HPLC
Abs. @ 254 nm

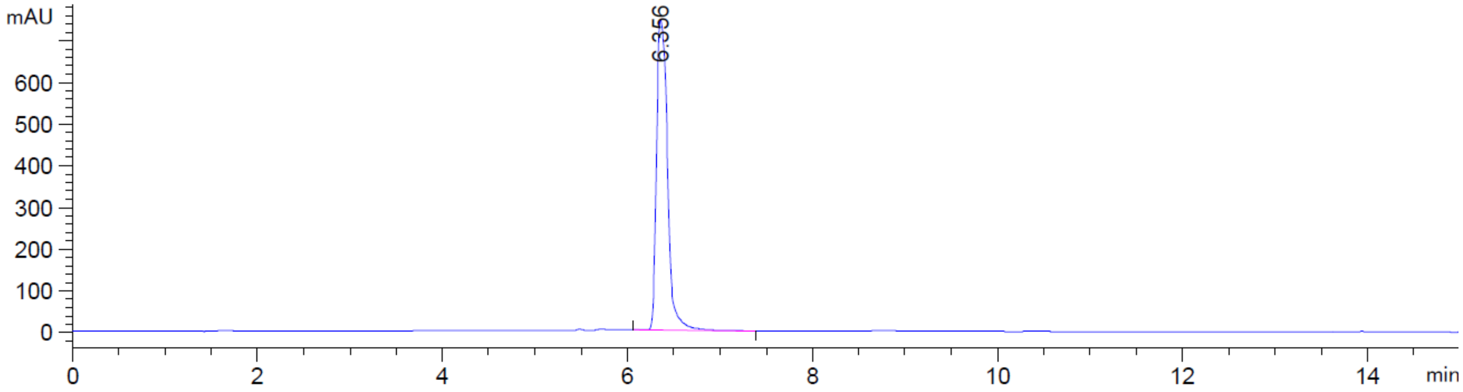

MS (-) ESI

(Calc. [M-H]$^-$ $C_{32}H_{43}N_{15}O_{27}P_5^-$ 1224.11464)

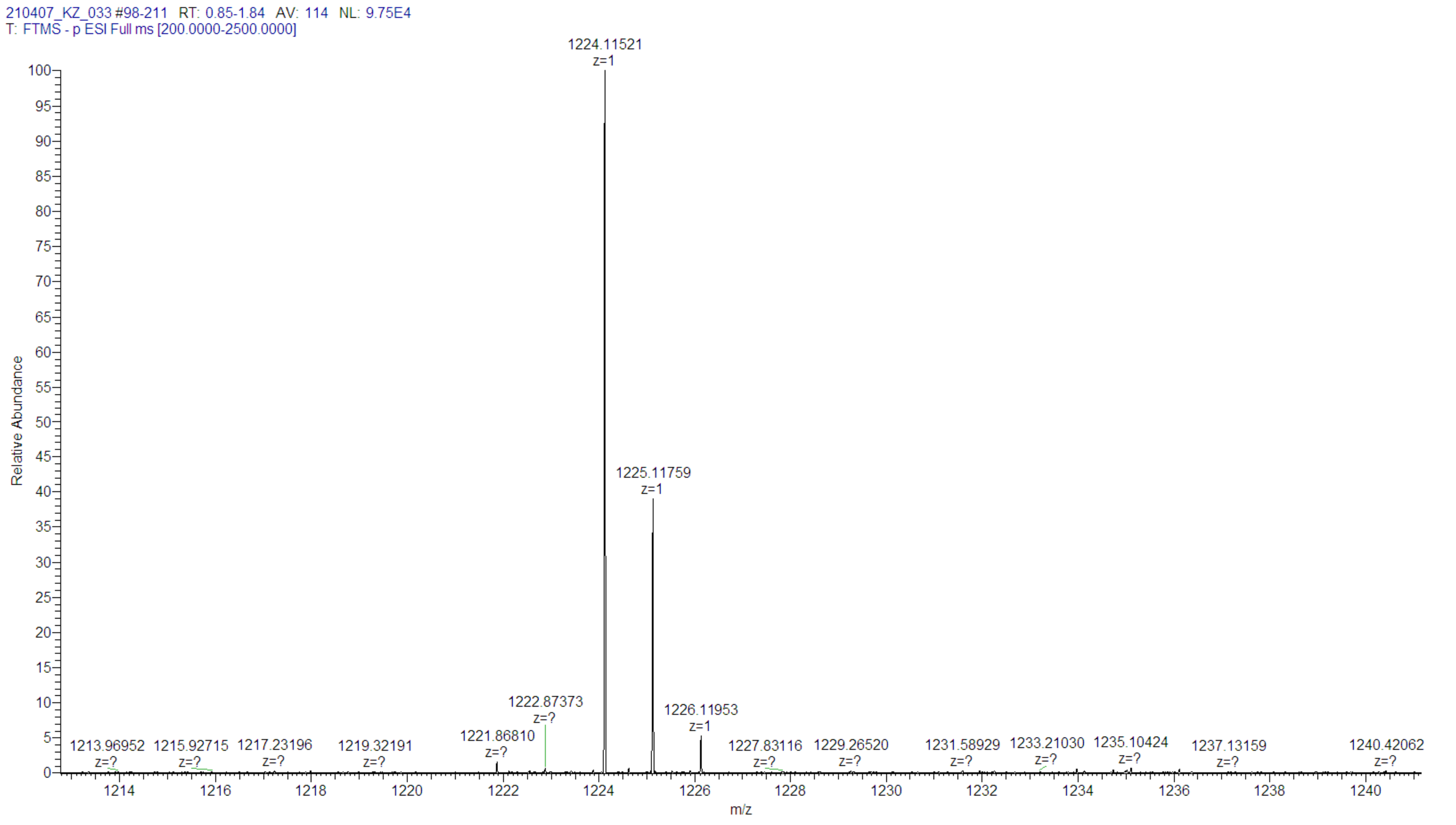

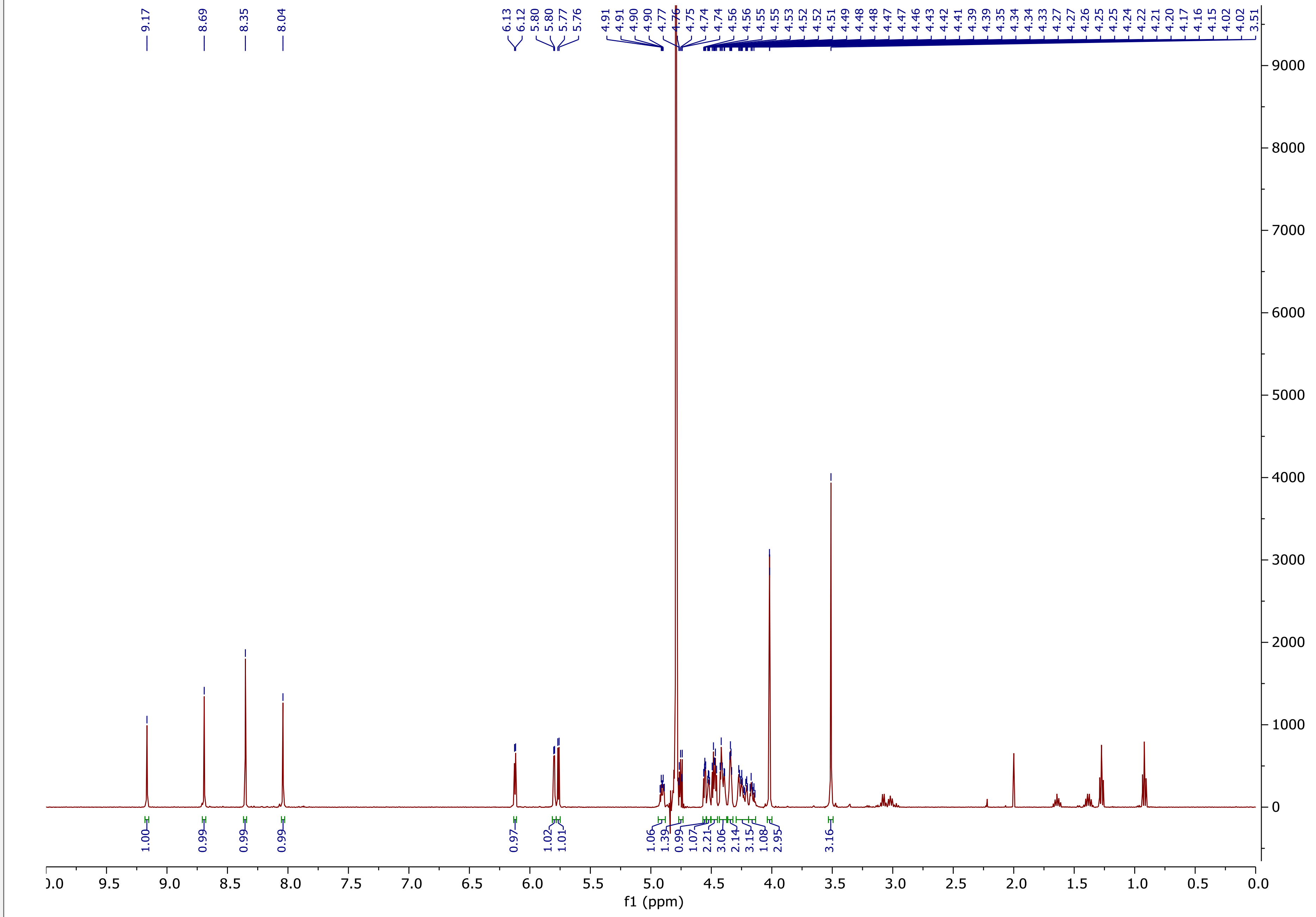

^{1}H NMR (500 MHz, D_2O, 25°C)

COSY NMR (D_2O, 25°)

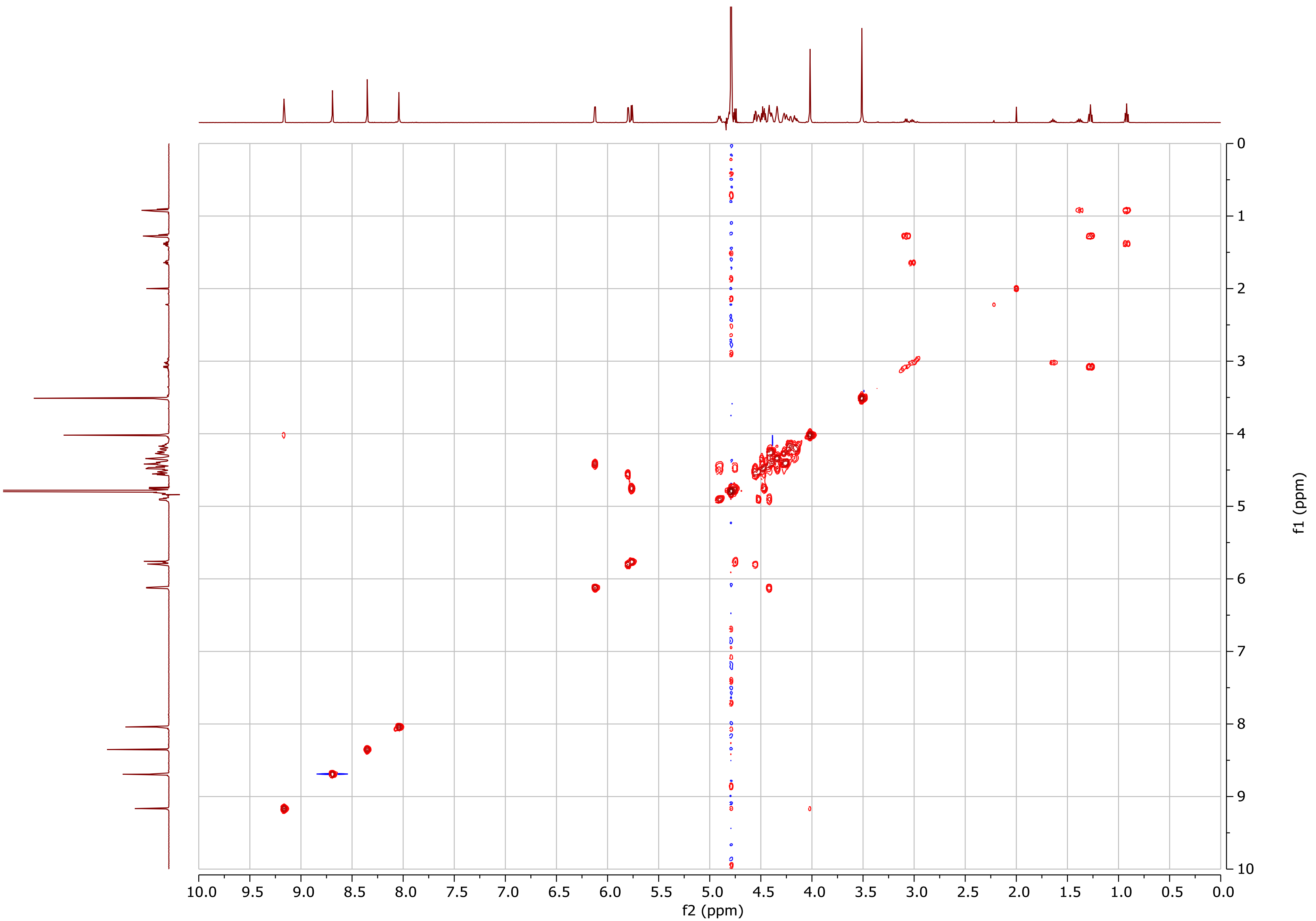

^{31}P NMR (202.5 MHz, D_2O, 25°C)

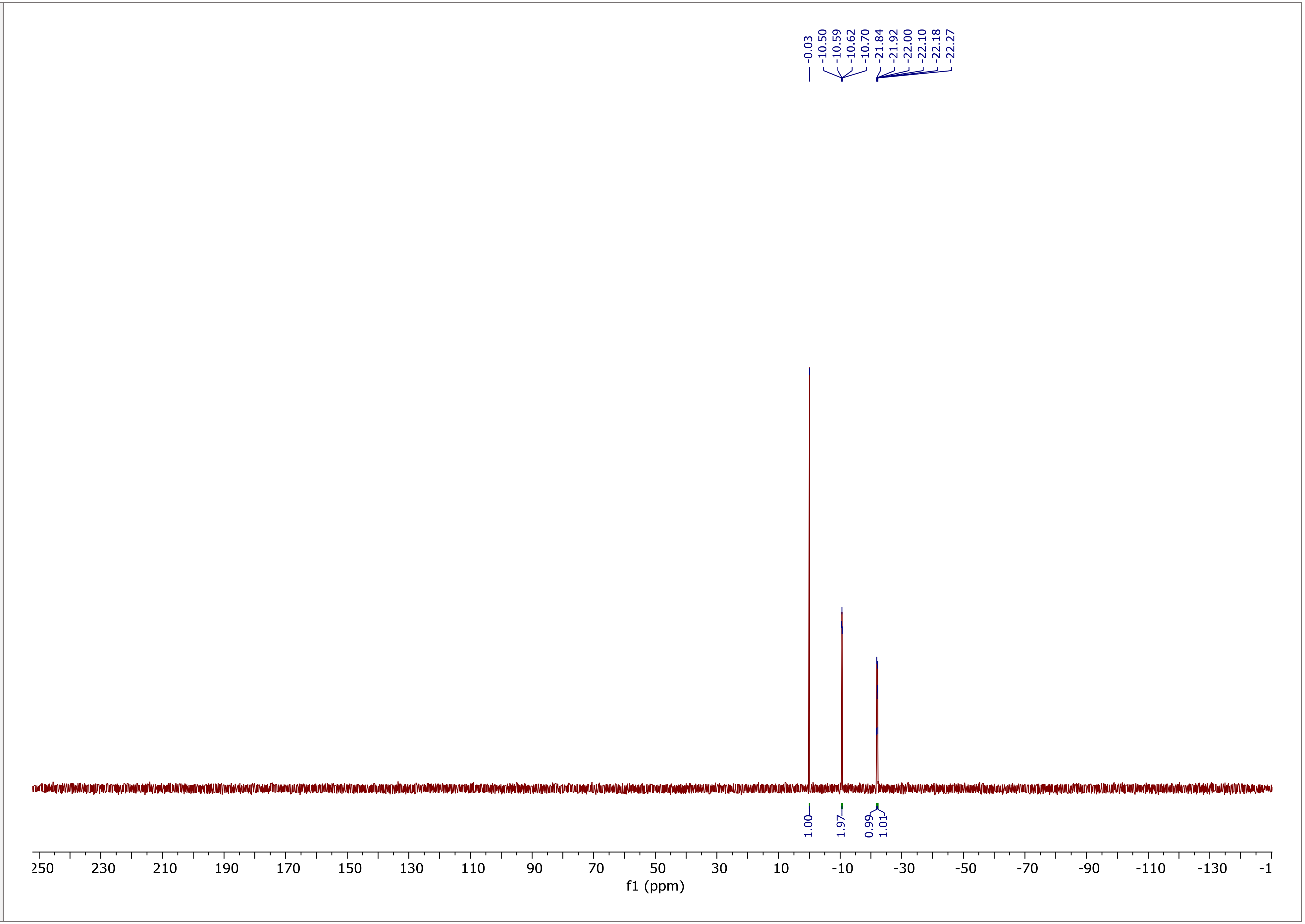

^1H-^{13}C HSQC (D_2O, 25°C)

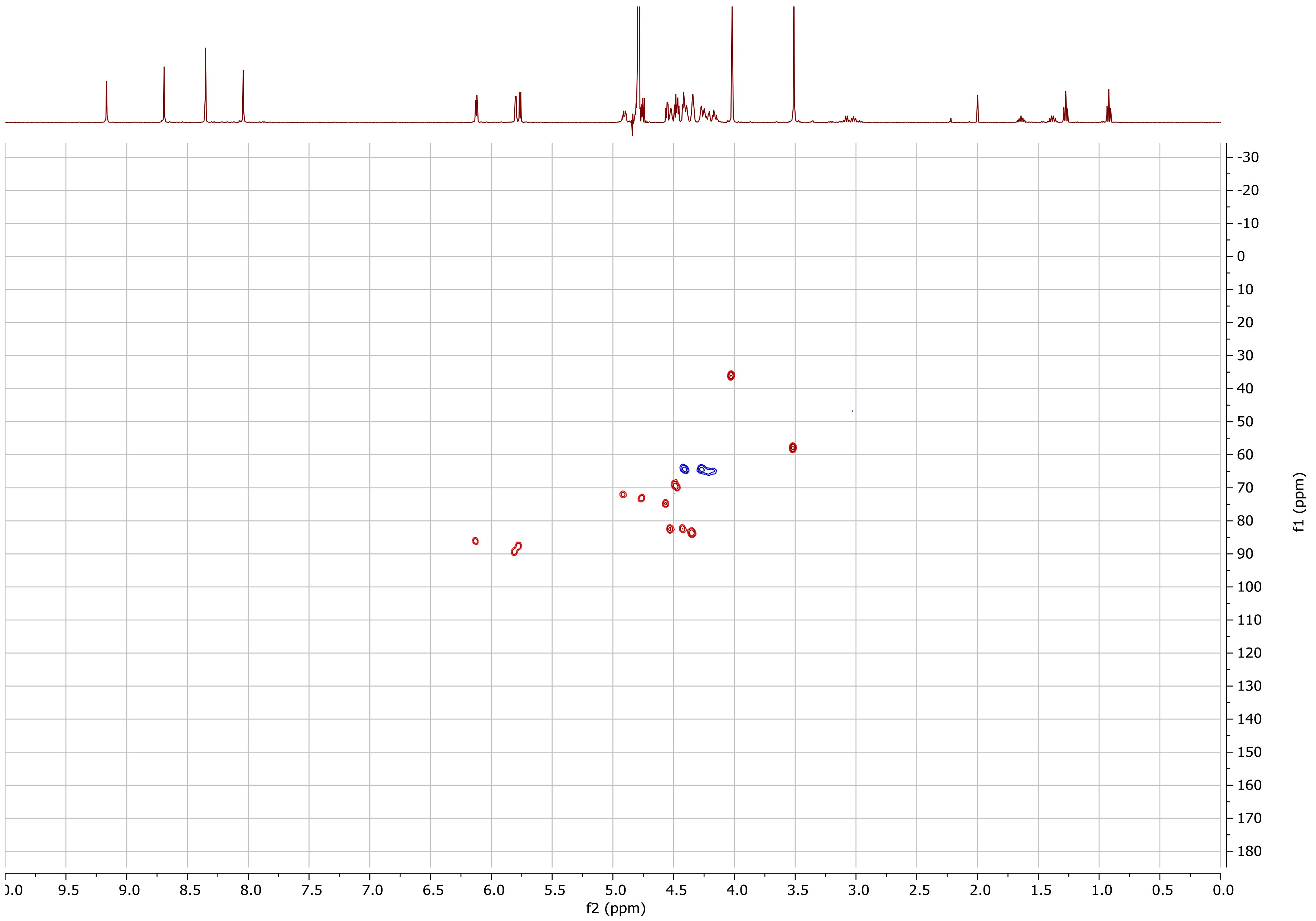

^{1}H-^{31}P HSQC (D_2O, 25°C)

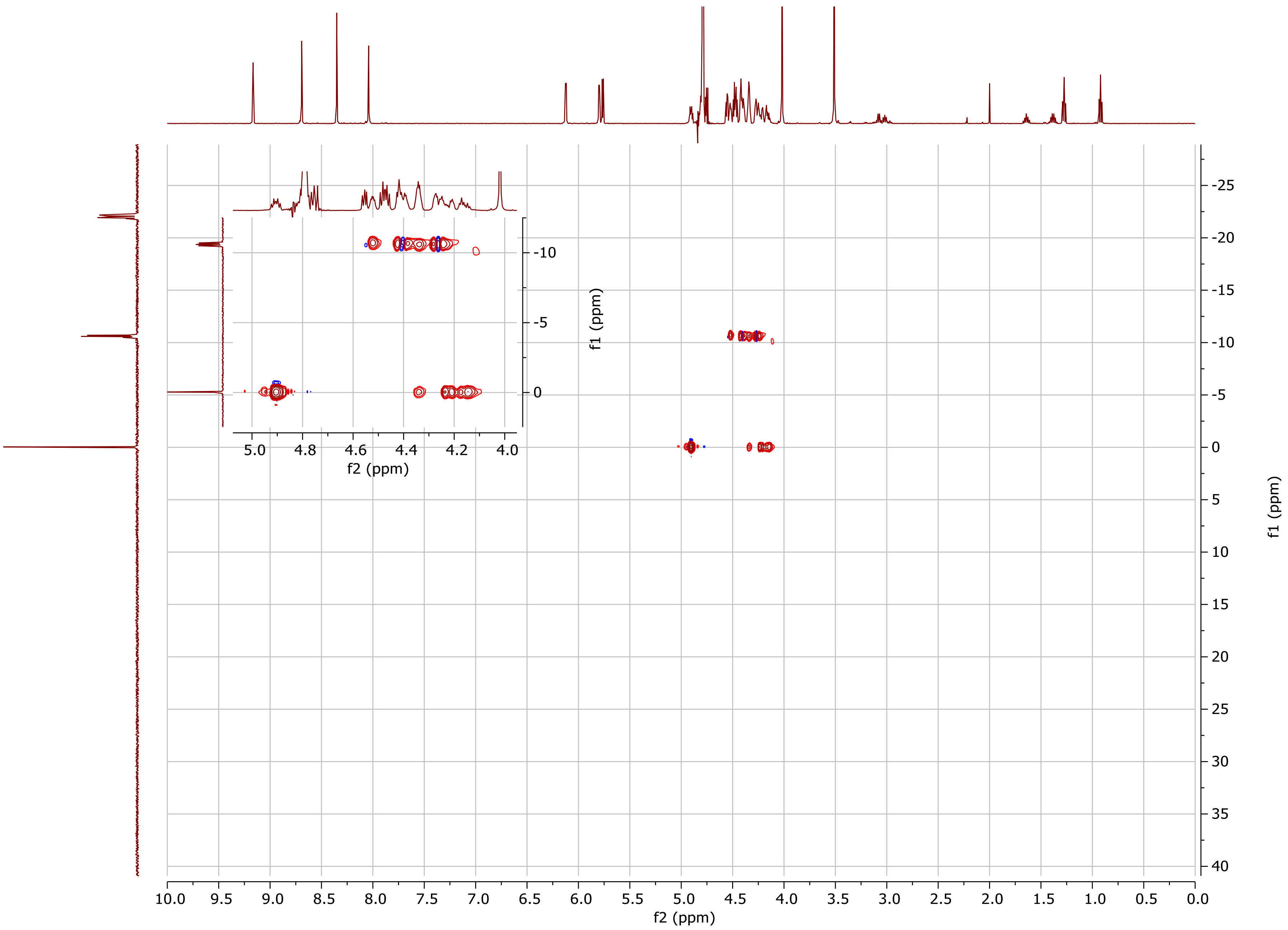

TOCSY (D_2O, 25°C)

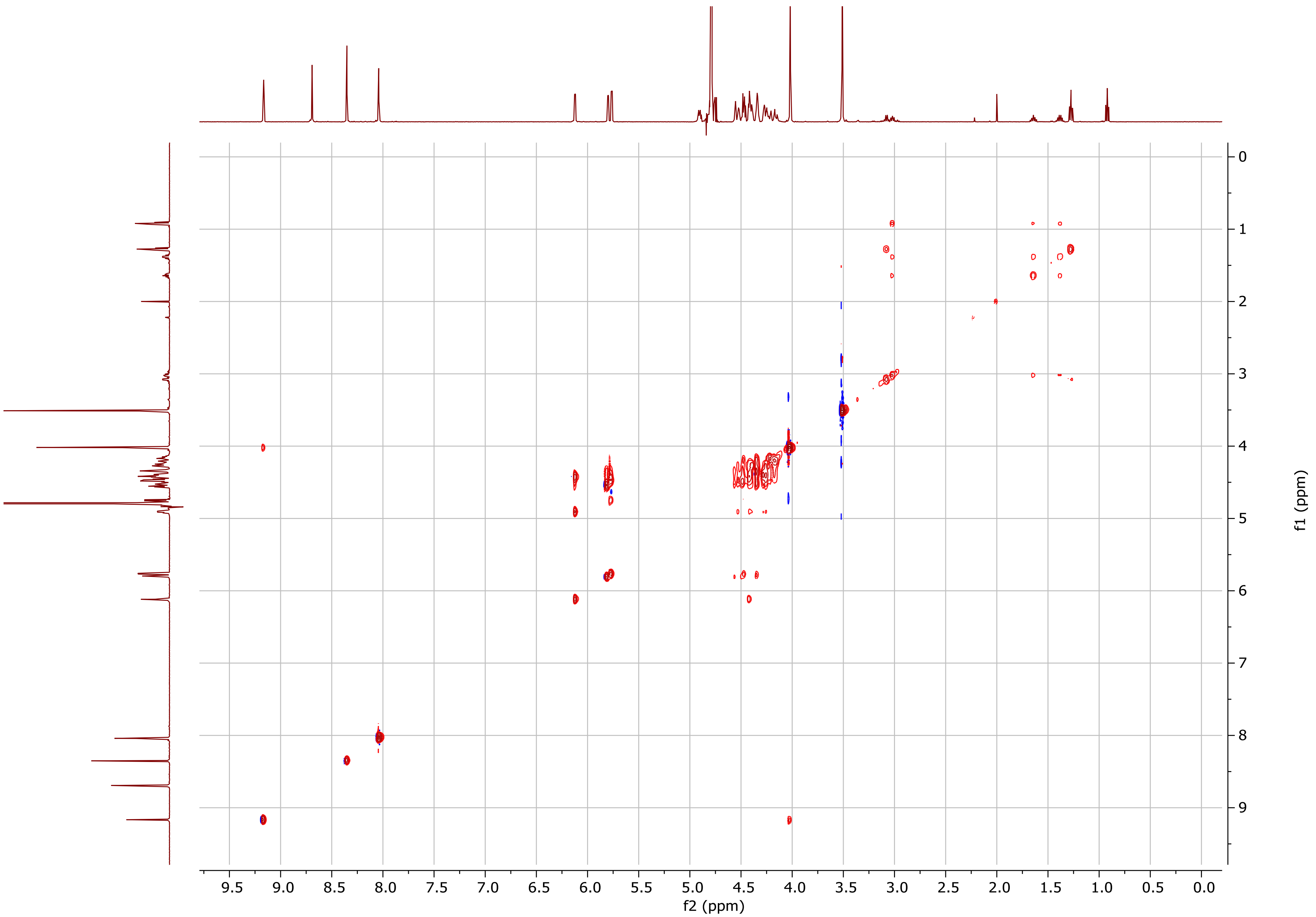

(12) m^{7}Gpppp^{m6}A$_{m}$pG	
**Chemical structure**	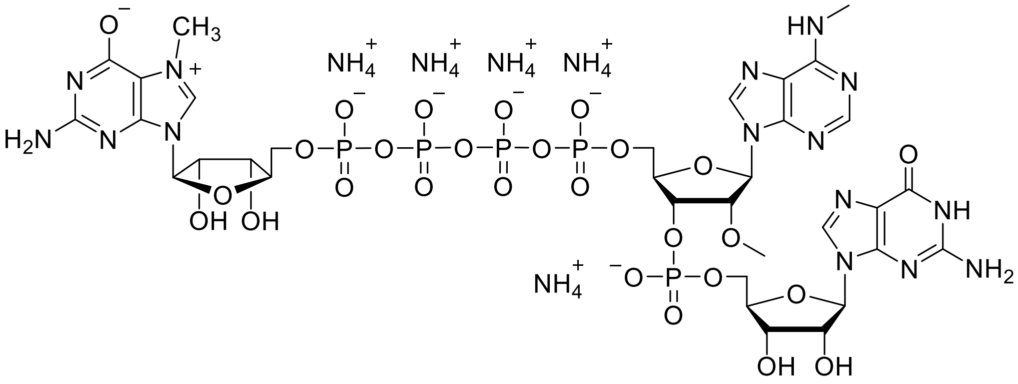
**RP HPLC** Abs. @ 254 nm	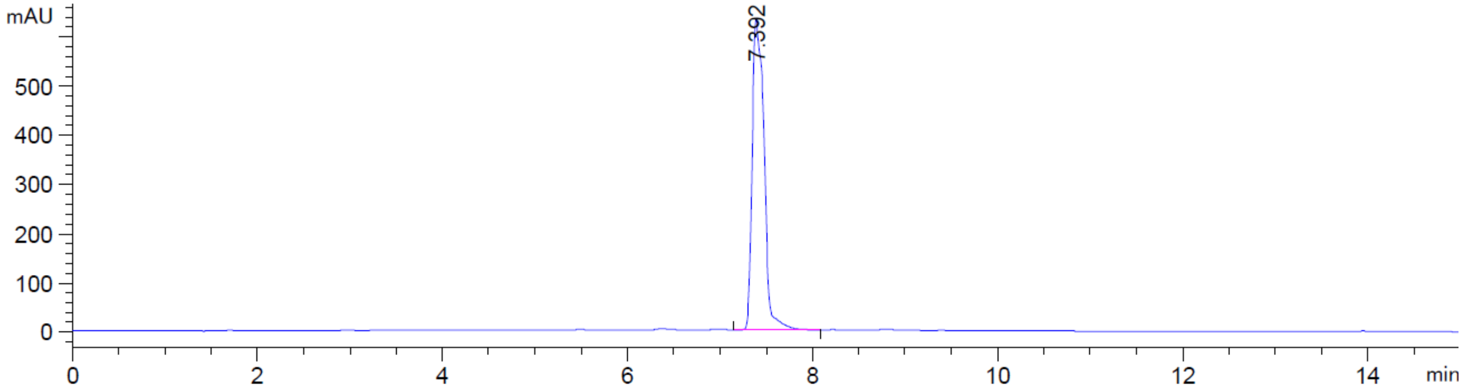

MS (-) ESI

(Calc. [M-H]$^-$ $C_{33}H_{45}N_{15}O_{27}P_5^-$ 1238.13029)

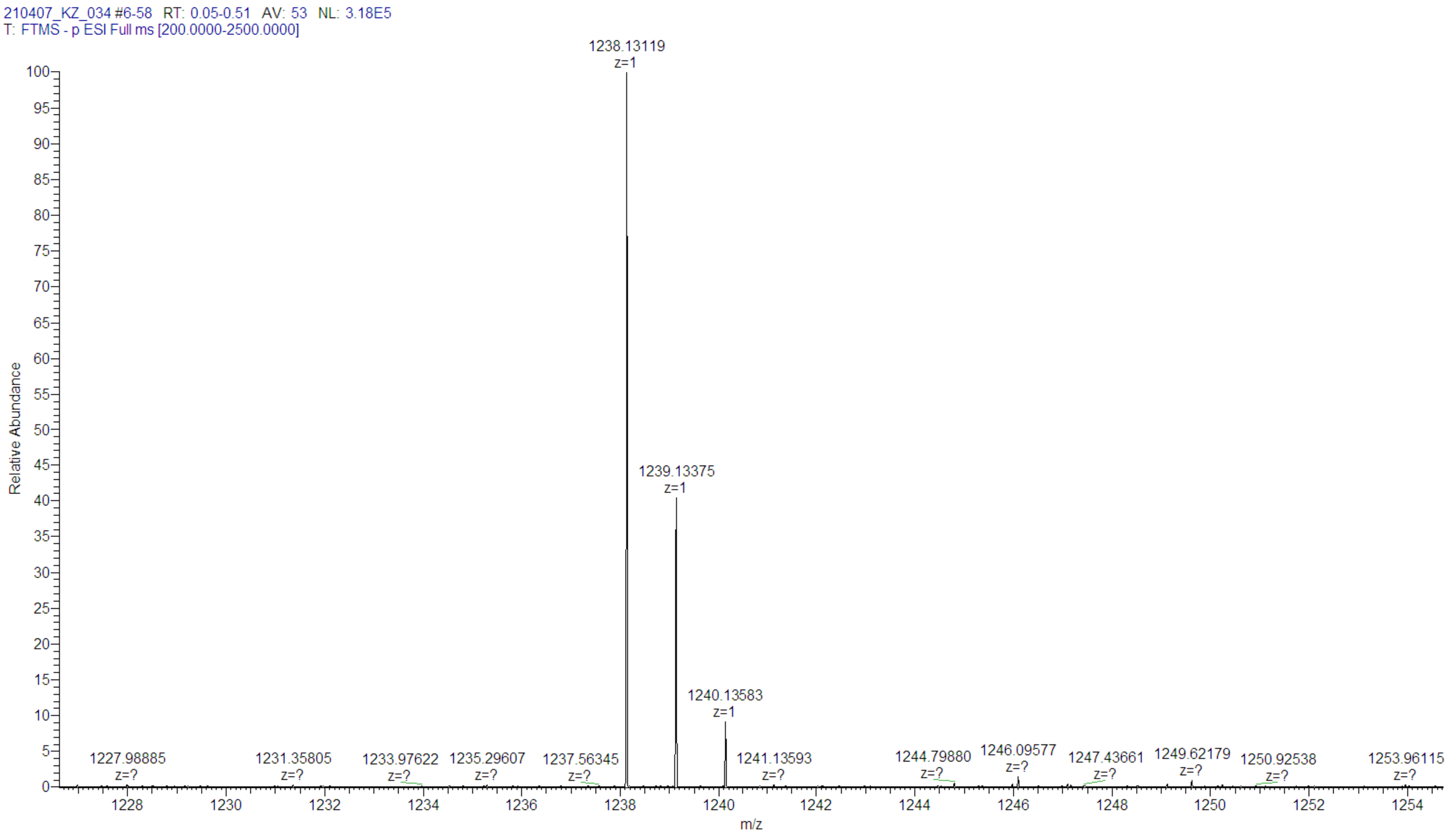

^{1}H NMR (500 MHz, D_2O, 25°C)

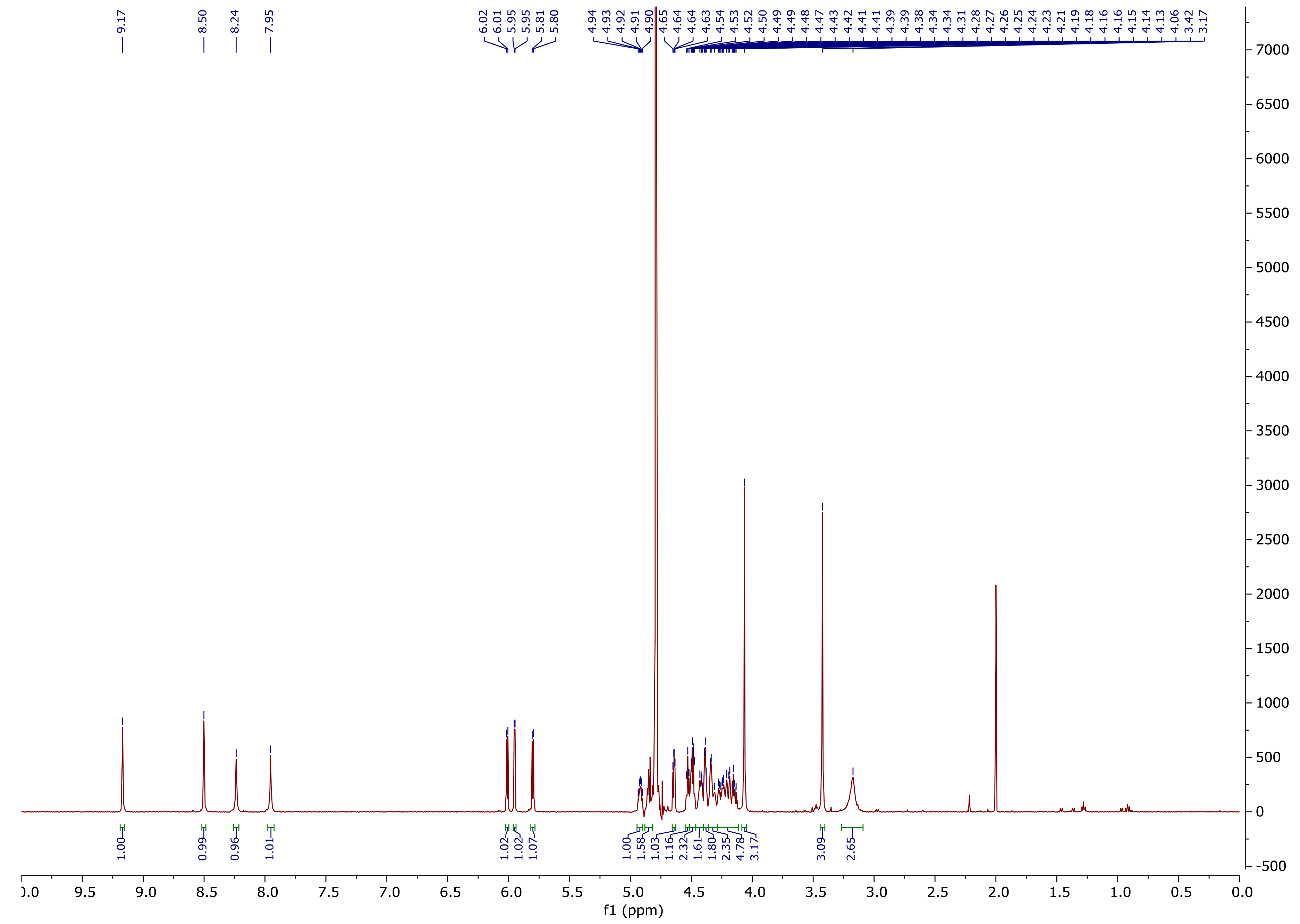

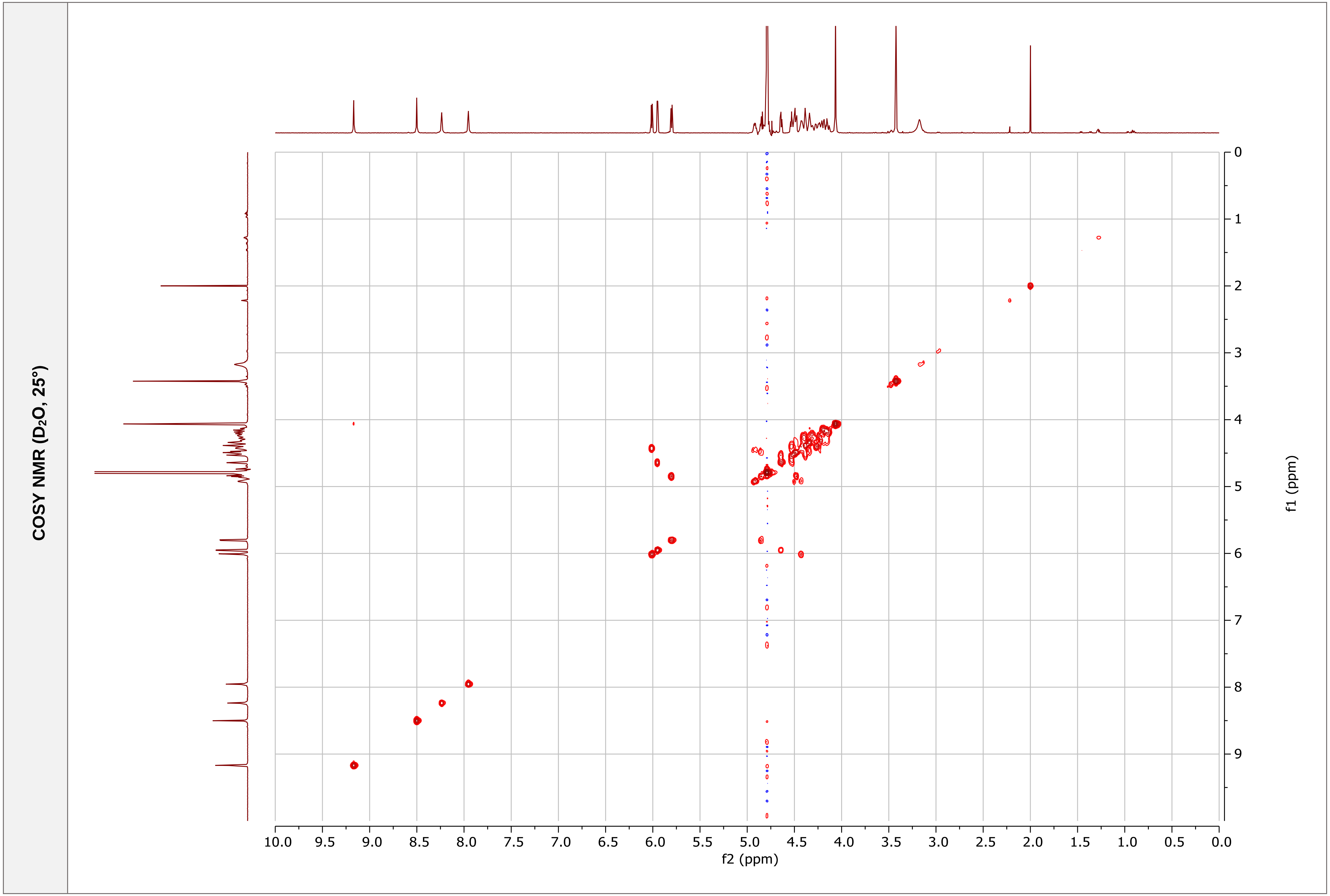
COSY NMR (D2O, 25°)
f2 (ppm)
f1 (ppm)
10.0
9.5
9.0
8.5
8.0
7.5
7.0
6.5
6.0
5.5
5.0
4.5
4.0
3.5
3.0
2.5
2.0
1.5
1.0
0.5
0.0
0
1
2
3
4
5
6
7
8
9

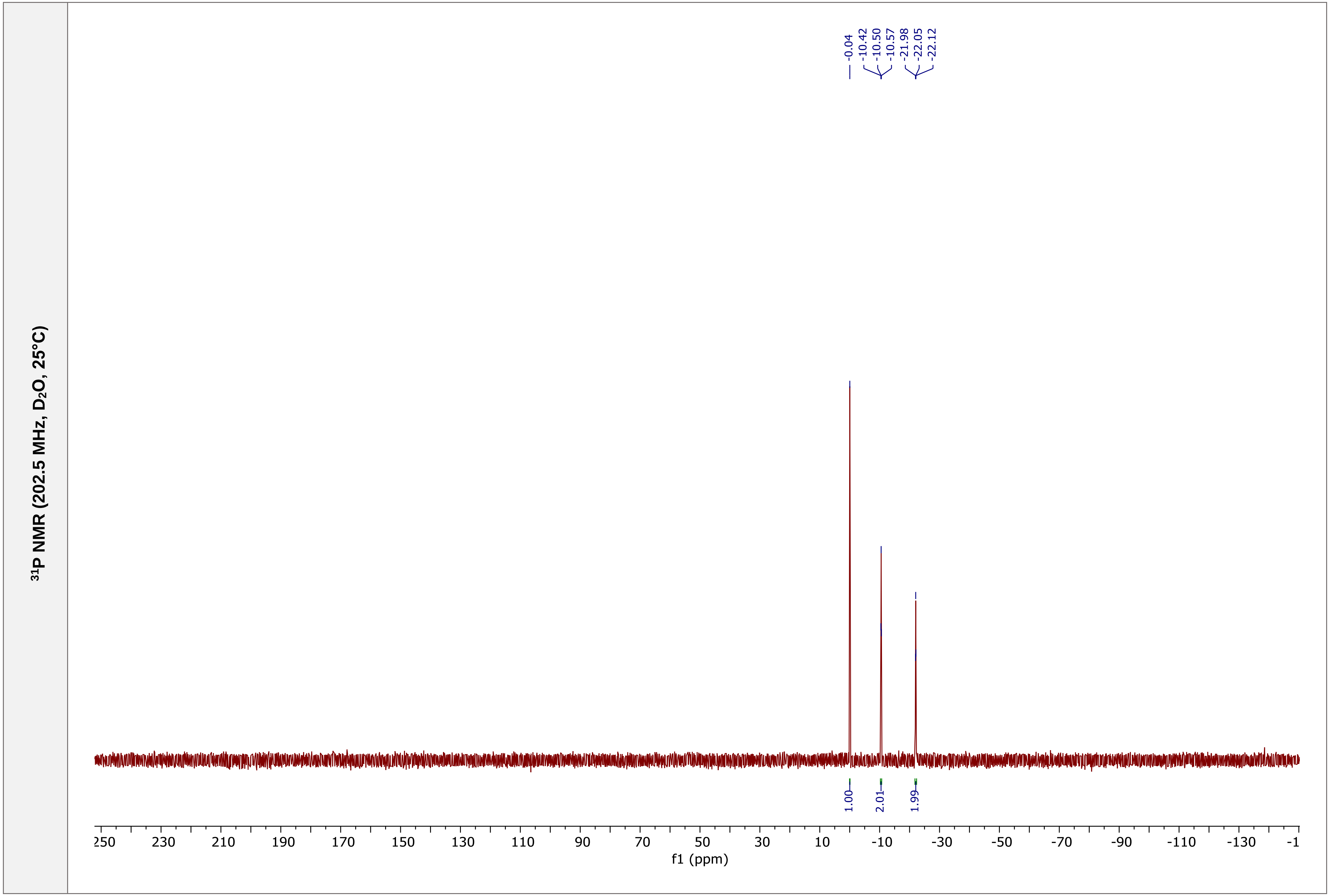

^{31}P NMR (202.5 MHz, D$_2$O, 25°C)
-0.04
-10.42
-10.50
-10.57
-21.98
-22.05
-22.12
1.00
2.01
1.99
250
230
210
190
170
150
130
110
90
70
50
30
10
-10
-30
-50
-70
-90
-110
-130
-1
f1 (ppm)

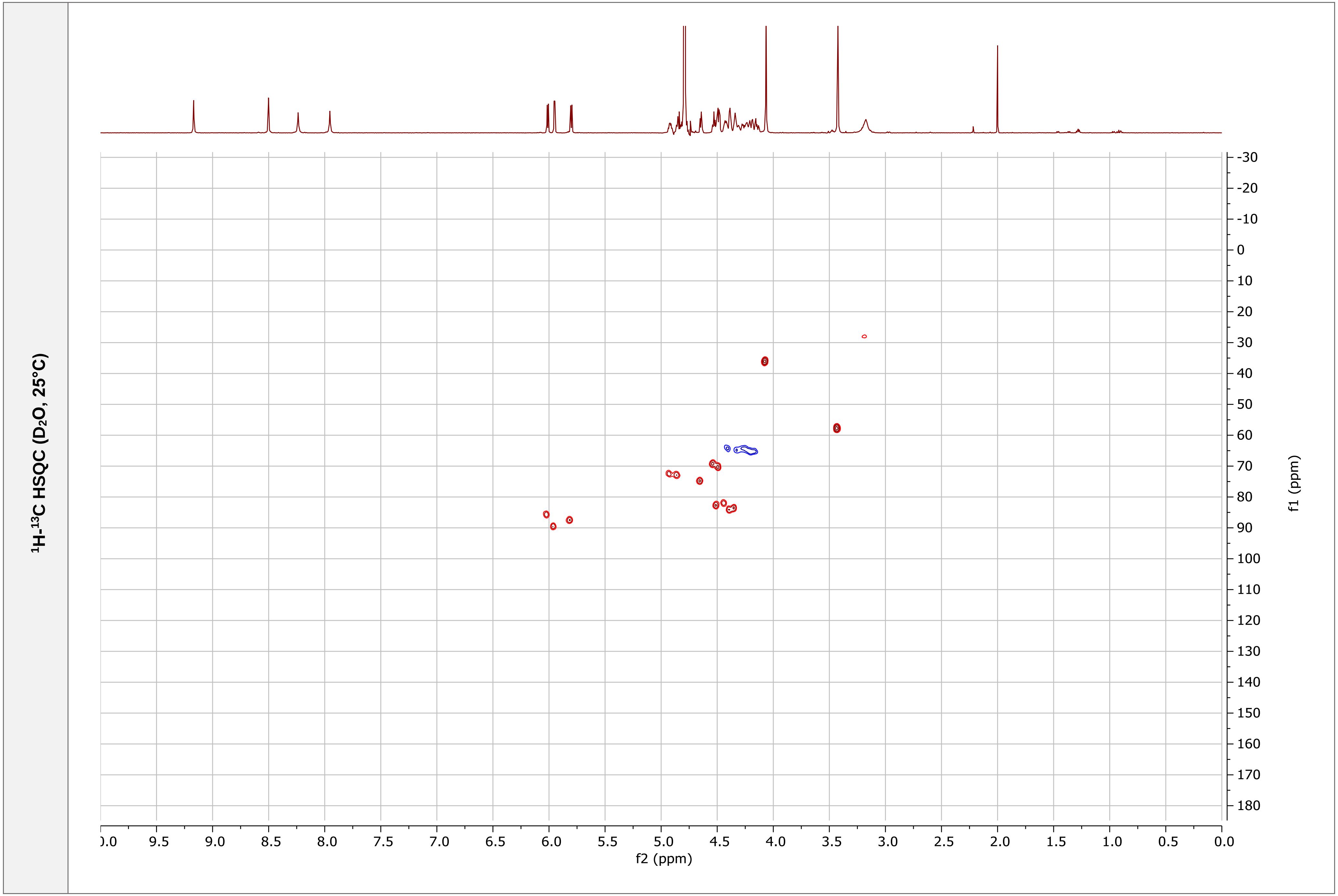

^{1}H-^{13}C HSQC (D_2O, 25°C)
f2 (ppm)
f1 (ppm)

^{1}H-^{31}P HSQC (D_2O, 25°C)

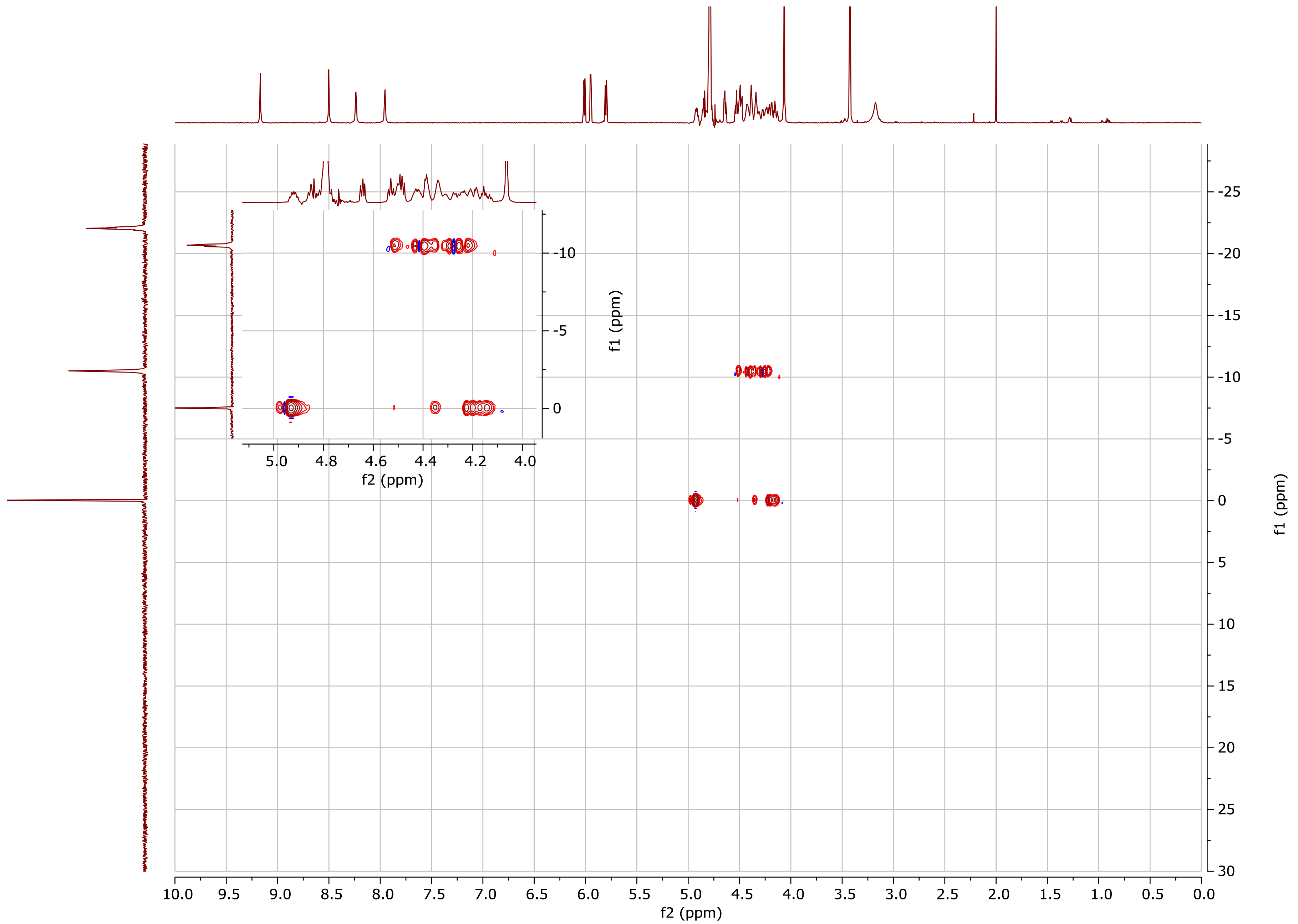

(13) m^{7}GppCCl$_2$ppApG	
**Chemical structure**	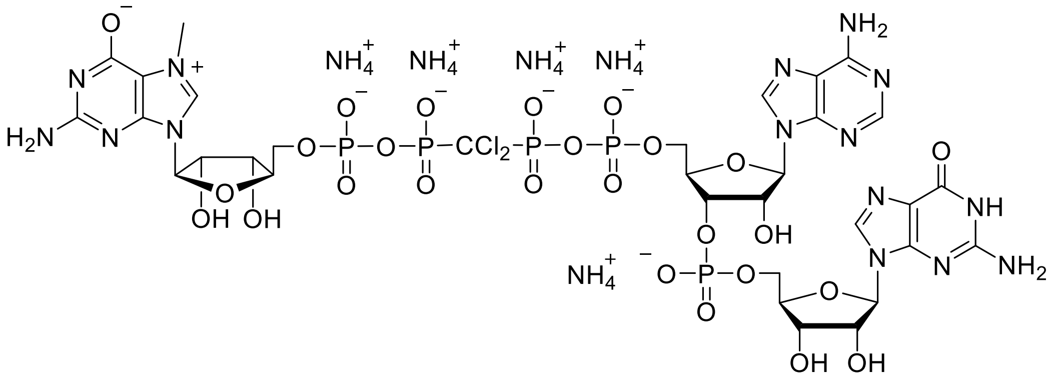
**RP HPLC** Abs. @ 254 nm	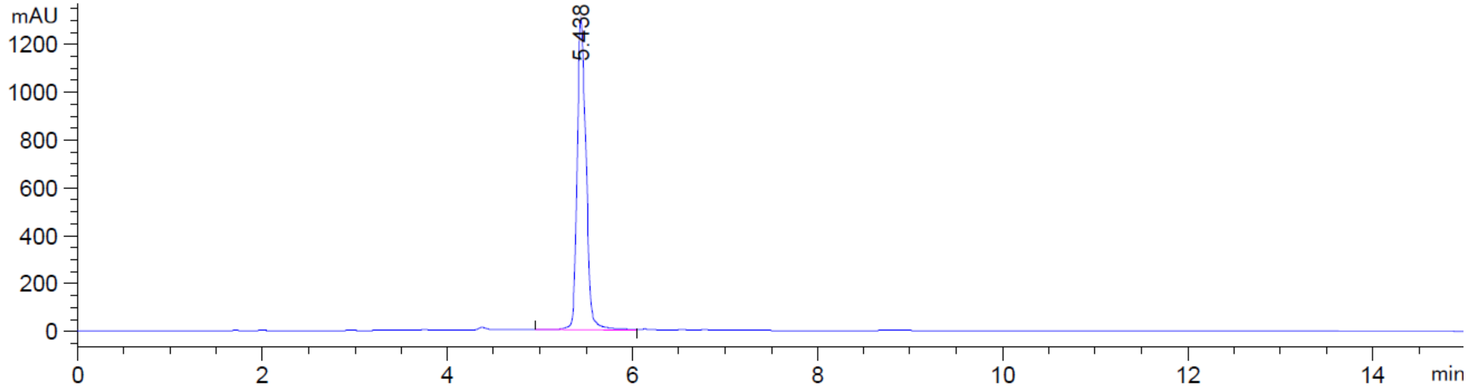

MS (-) ESI

(Calc. $[M-H]^-$ $C_{32}H_{41}Cl_2N_{15}O_{26}P_5^-$ 1276.04178)

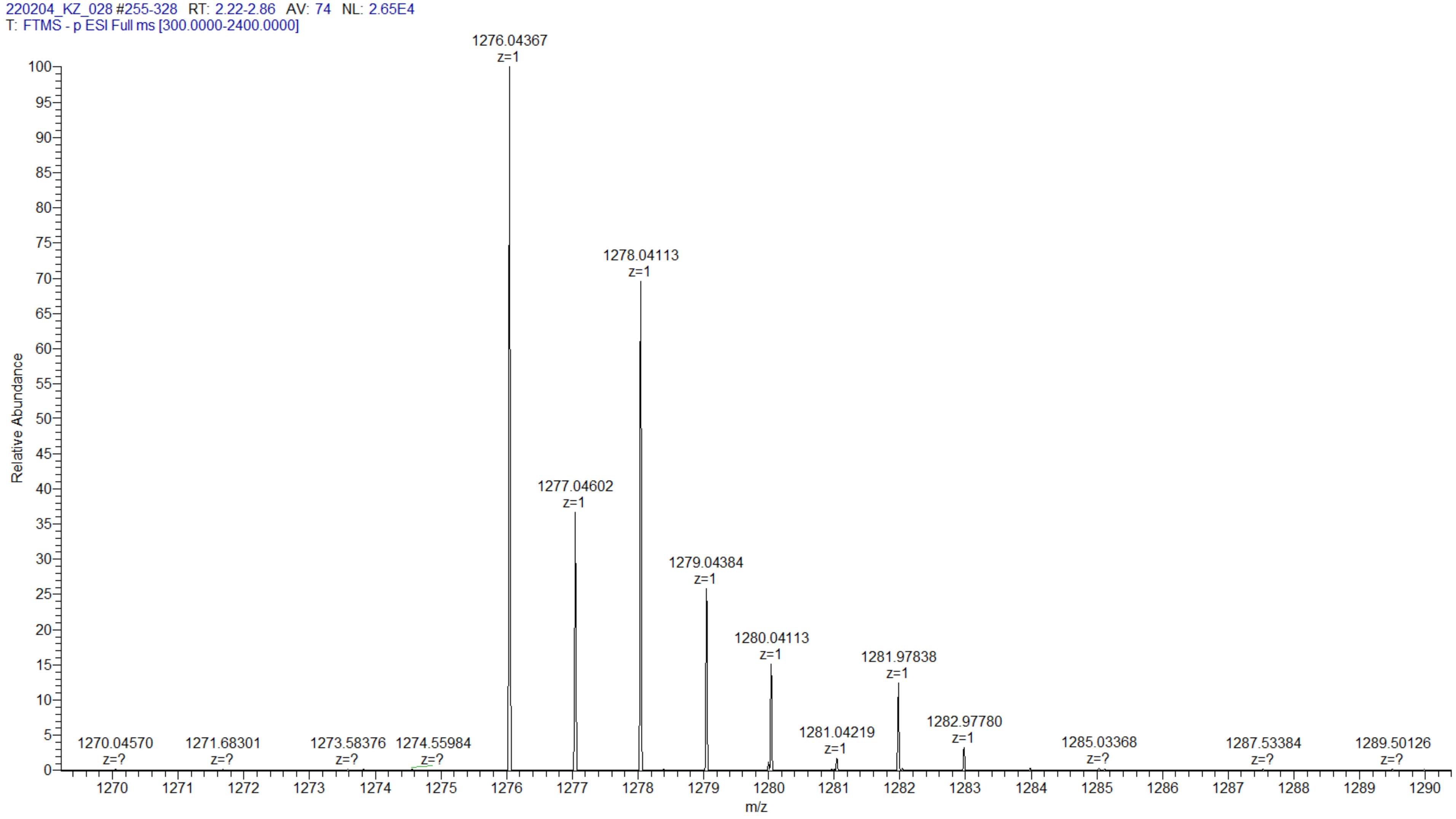

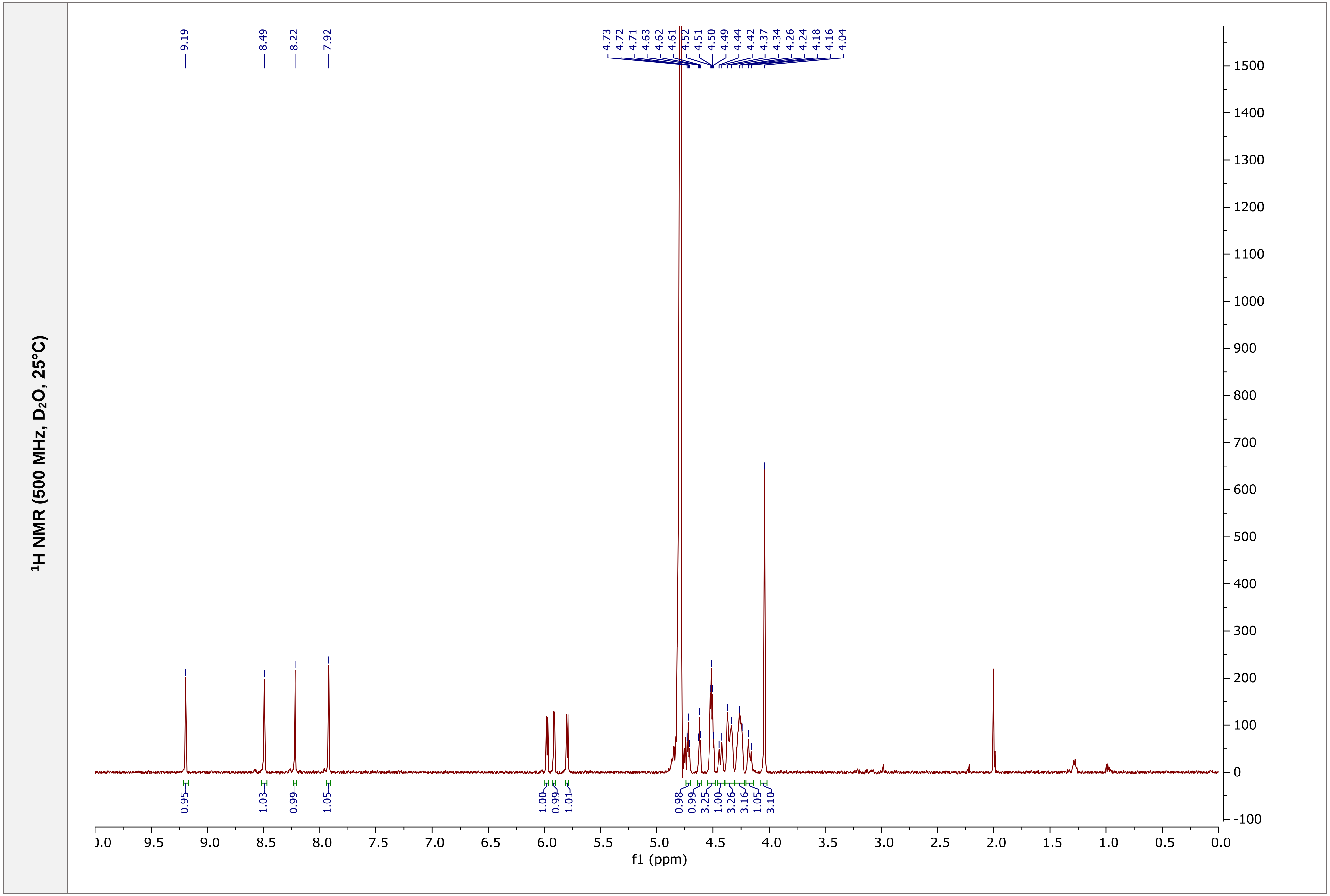
1H NMR (500 MHz, D2O, 25°C)
9.19
8.49
8.22
7.92
4.73
4.72
4.71
4.63
4.62
4.61
4.52
4.51
4.50
4.49
4.44
4.42
4.37
4.34
4.26
4.24
4.18
4.16
4.04
0.95
1.03
0.99
1.05
1.00
0.99
1.01
0.98
0.99
3.25
1.00
3.26
3.16
1.05
3.10
f1 (ppm)

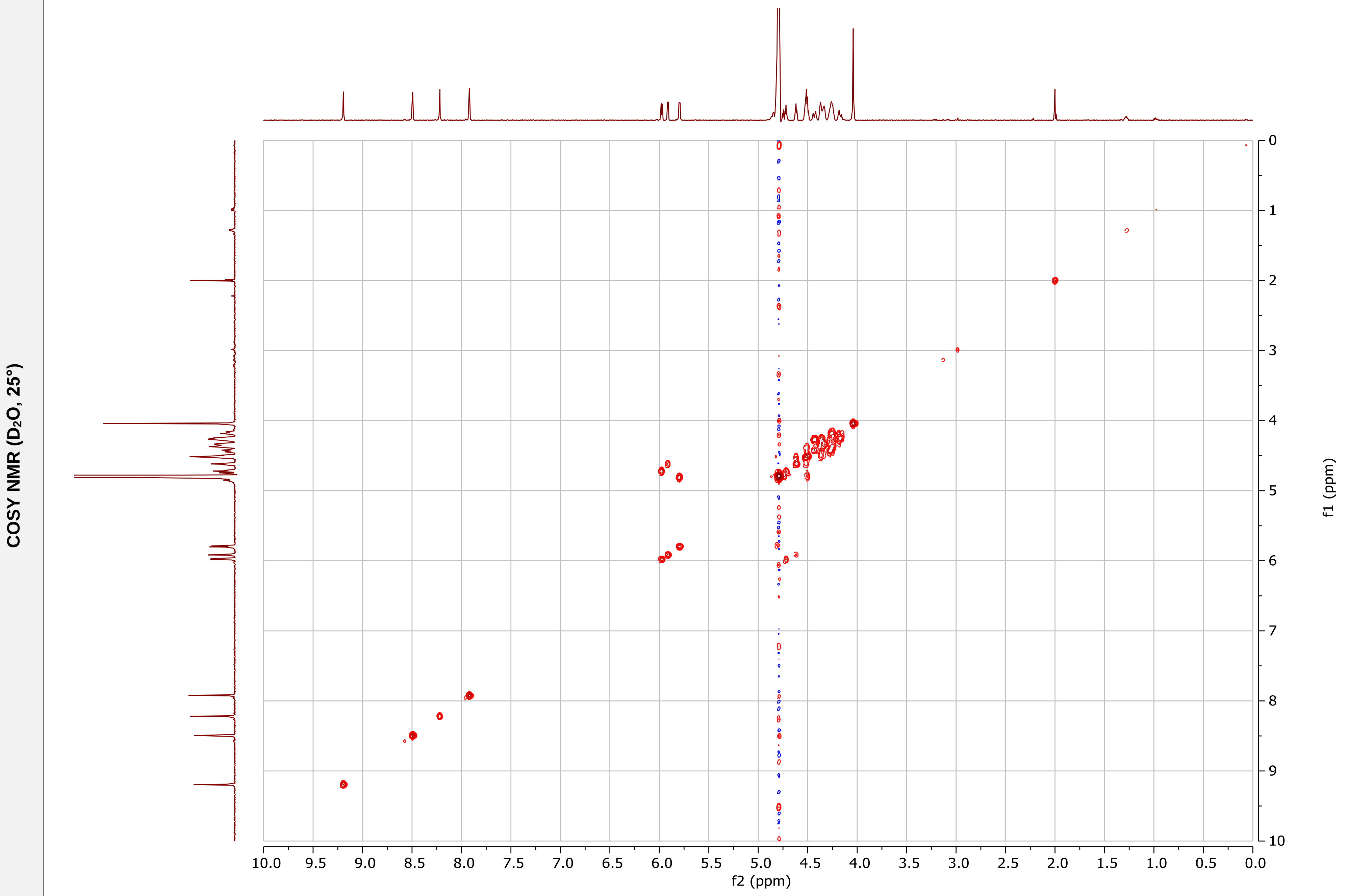
COSY NMR (D_2O, 25°)
f2 (ppm)
f1 (ppm)

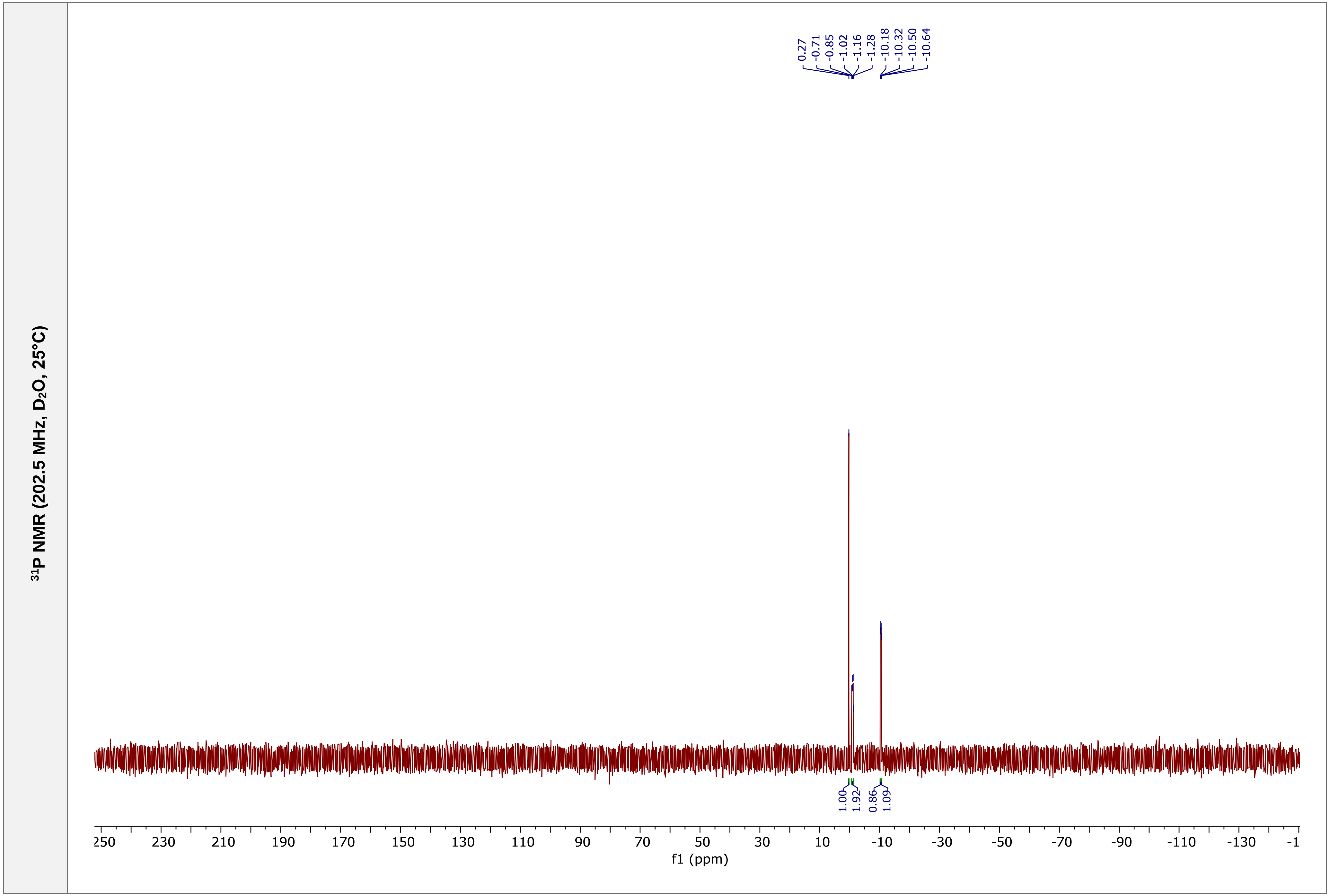

³¹P NMR (202.5 MHz, D₂O, 25°C)
0.27
-0.71
-0.85
-1.02
-1.16
-1.28
-10.18
-10.32
-10.50
-10.64
1.00
1.92
0.86
1.09
250
230
210
190
170
150
130
110
90
70
50
30
10
-10
-30
-50
-70
-90
-110
-130
f1 (ppm)

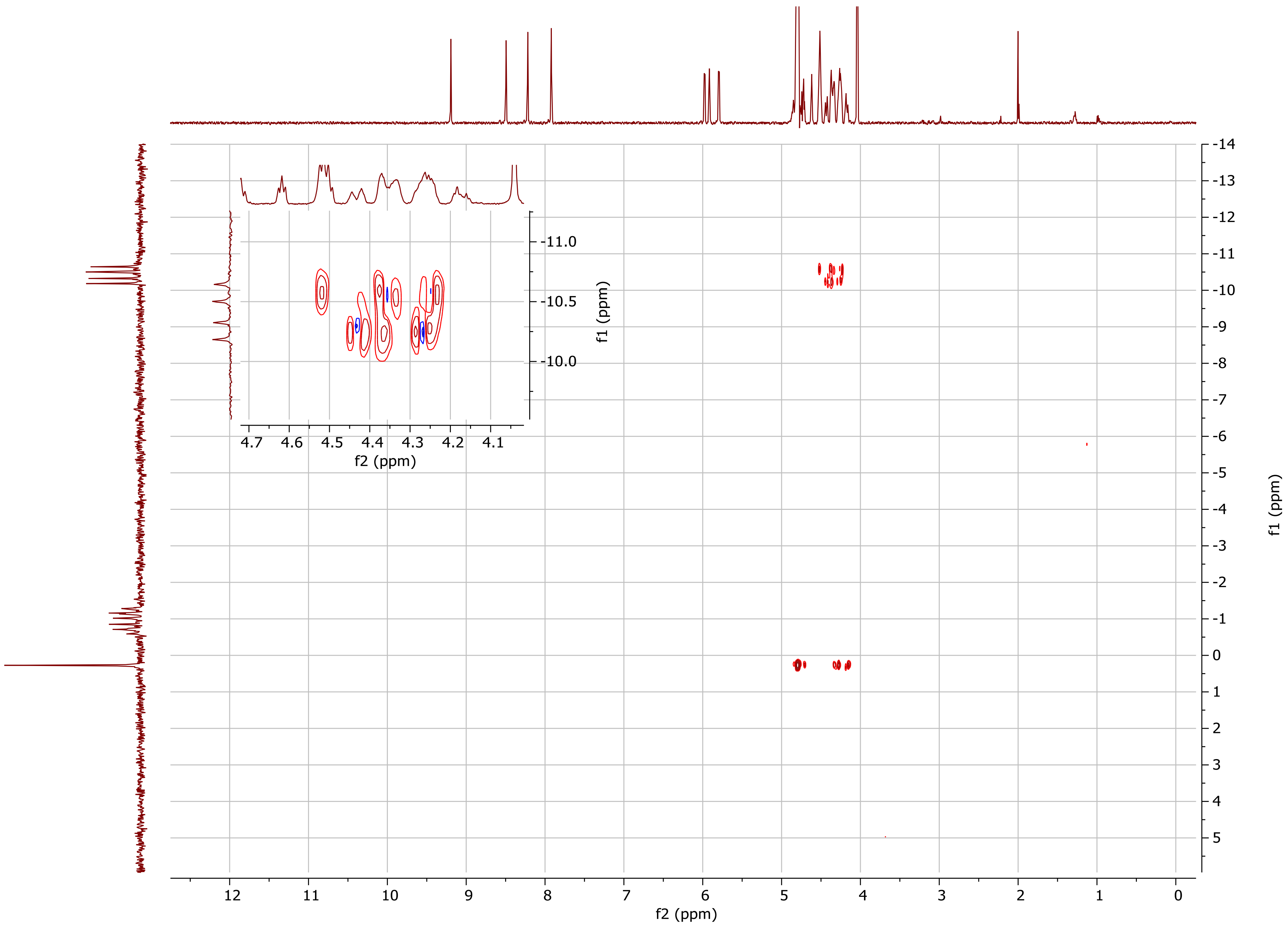

^{1}H-^{31}P HSQC (D_2O, 25°C)

(14) m^{7}GppCCl$_2$ppA$_m$pG

Chemical structure

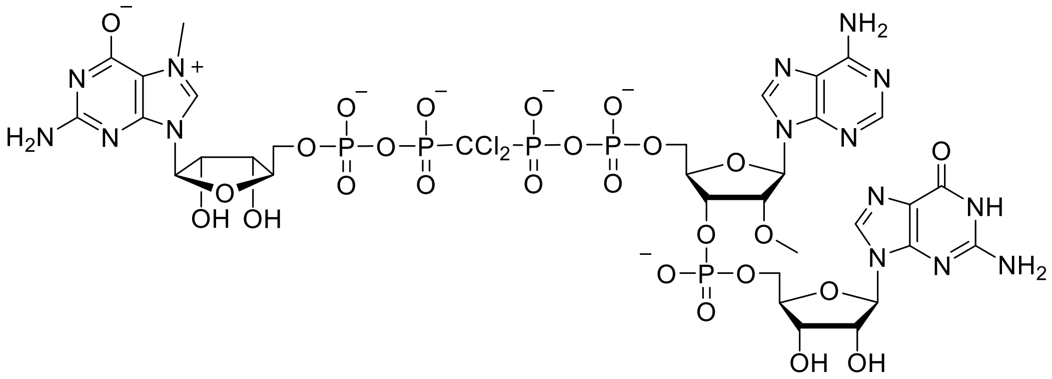

RP HPLC
Abs. @ 254 nm

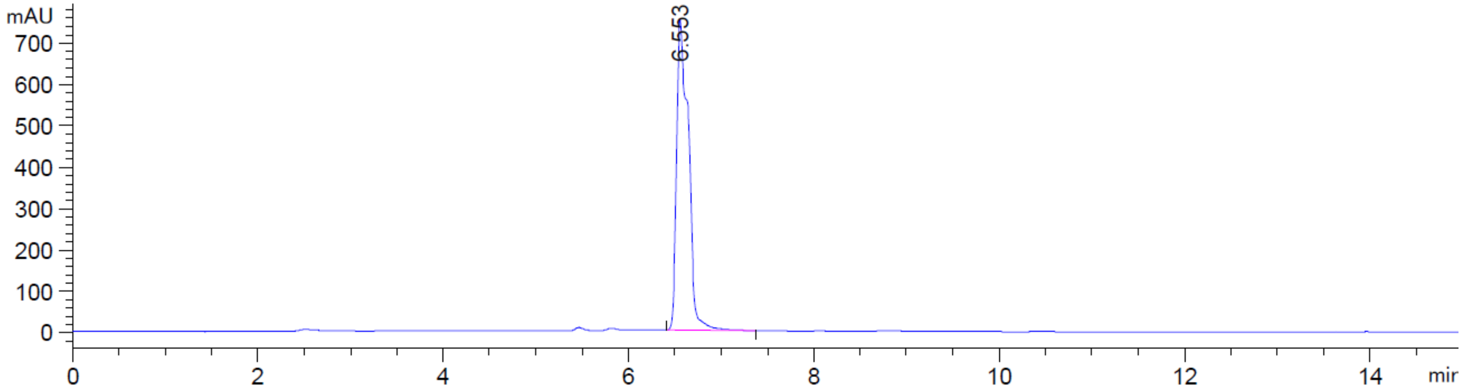

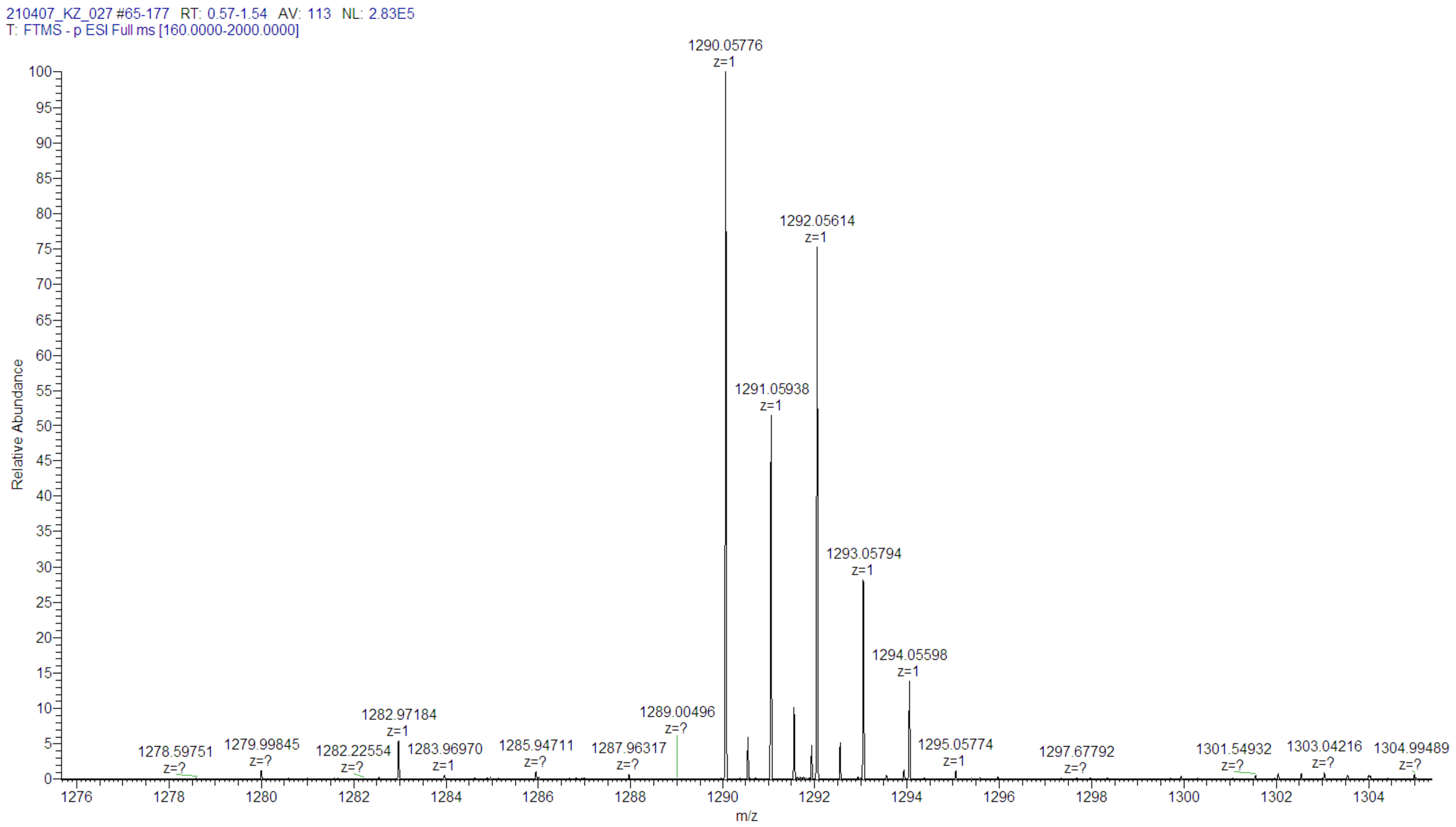

MS (-) ESI

(Calc. [M-H]$^-$ $C_{33}H_{43}Cl_2N_{15}O_{26}P_5^-$ 1290.05743)

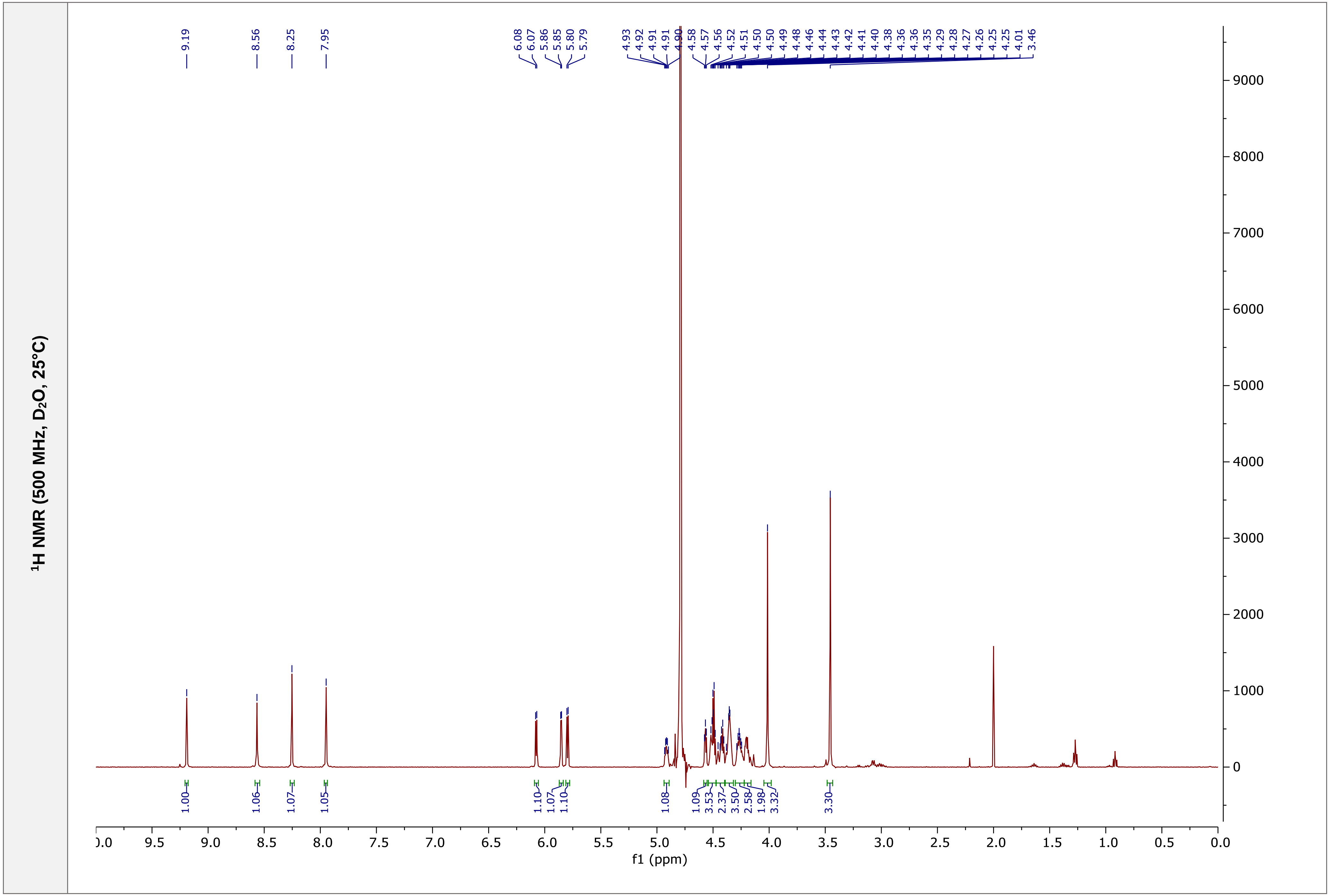

1H NMR (500 MHz, D2O, 25°C)
9.19
8.56
8.25
7.95
6.08
6.07
5.86
5.85
5.80
5.79
4.93
4.92
4.91
4.91
4.90
4.58
4.57
4.56
4.52
4.51
4.50
4.50
4.49
4.48
4.46
4.44
4.43
4.42
4.41
4.40
4.38
4.36
4.36
4.35
4.29
4.28
4.27
4.26
4.25
4.25
4.01
3.46
1.00
1.06
1.07
1.05
1.10
1.07
1.10
1.08
1.09
3.53
2.37
3.50
2.58
1.98
3.32
3.30
f1 (ppm)

COSY NMR (D_2O, 25°)

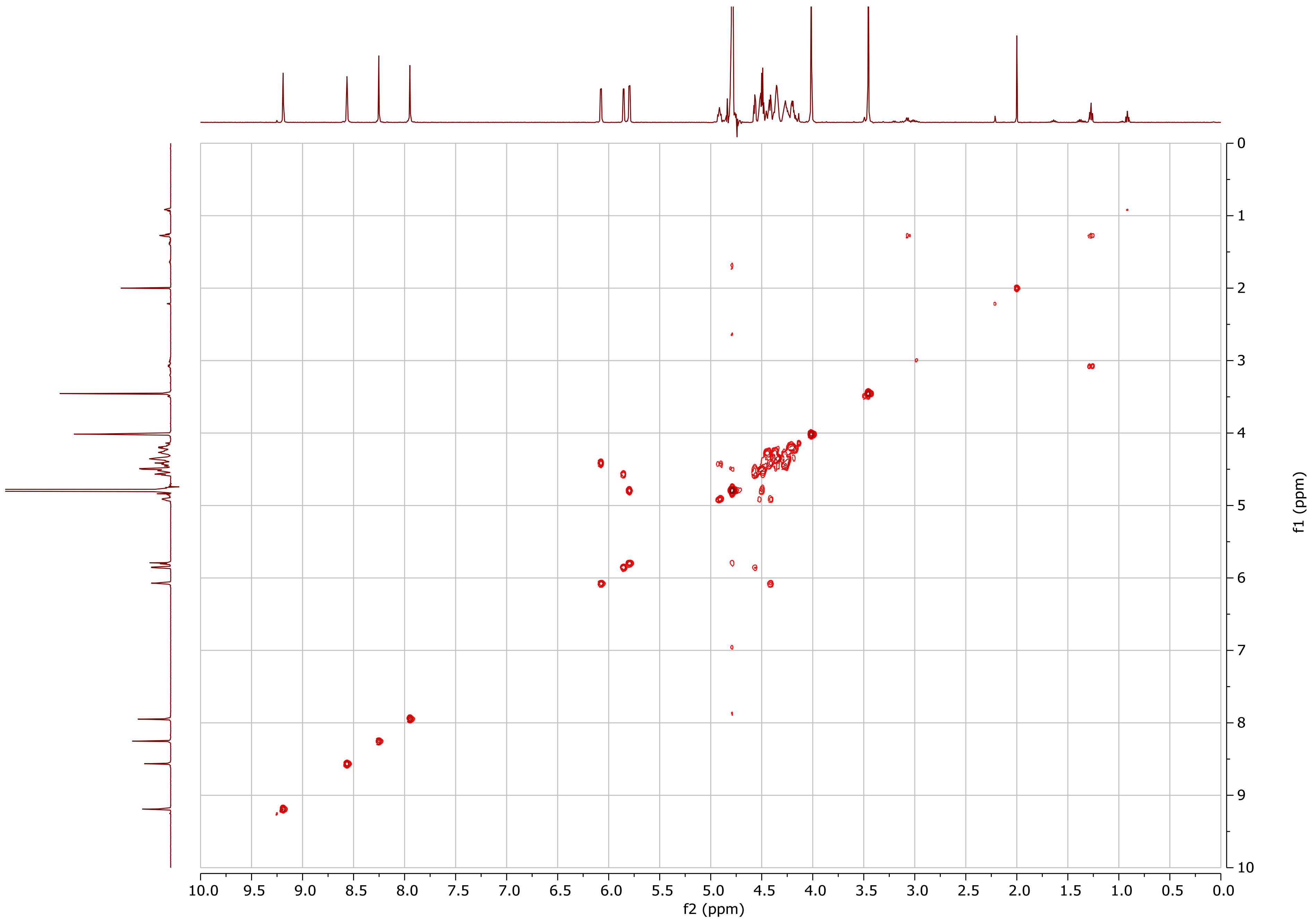

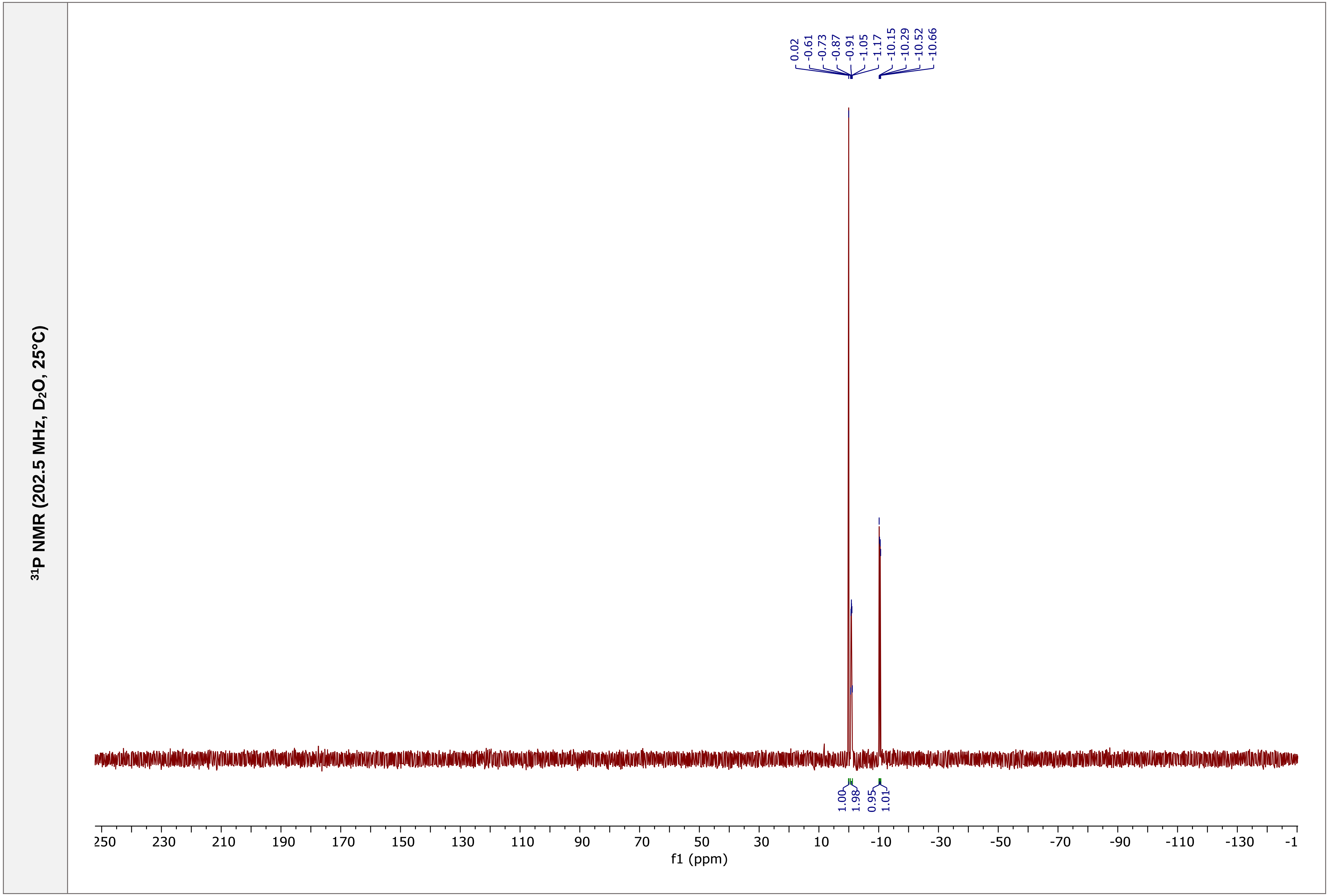

^{31}P NMR (202.5 MHz, D_2O, 25°C)
0.02
-0.61
-0.73
-0.87
-0.91
-1.05
-1.17
-10.15
-10.29
-10.52
-10.66
1.00
1.98
0.95
1.01
250
230
210
190
170
150
130
110
90
70
50
30
10
-10
-30
-50
-70
-90
-110
-130
-1
f1 (ppm)

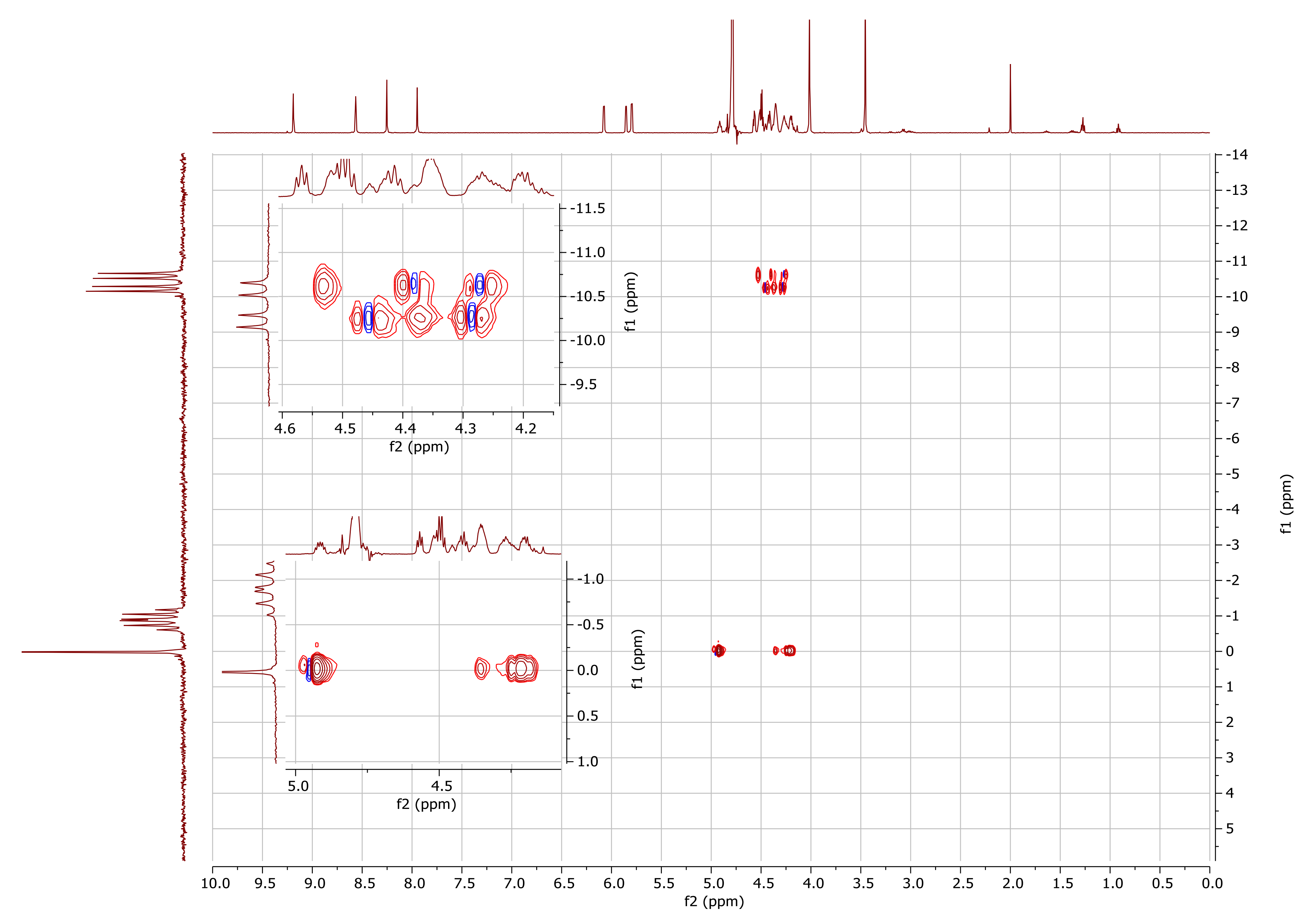

^{1}H-^{31}P HSQC (D_2O, 25°C)

(15) $m^7GppCCl_2pp^{m6}A_mpG$

Chemical structure

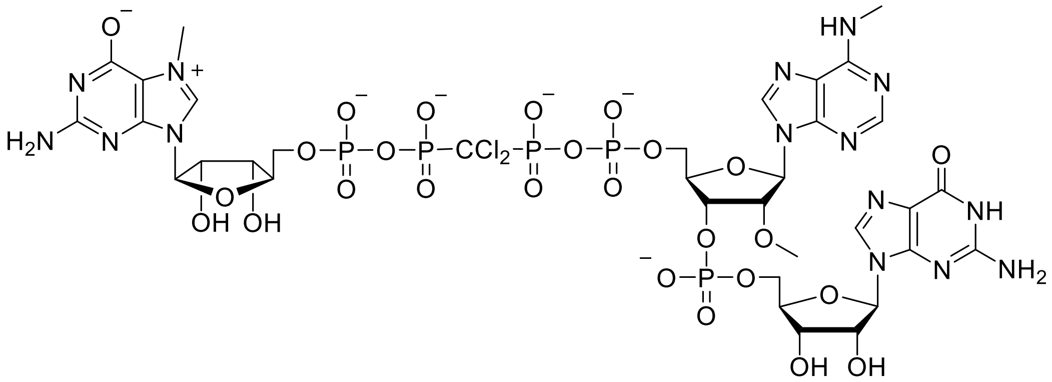

RP HPLC
Abs. @ 254 nm

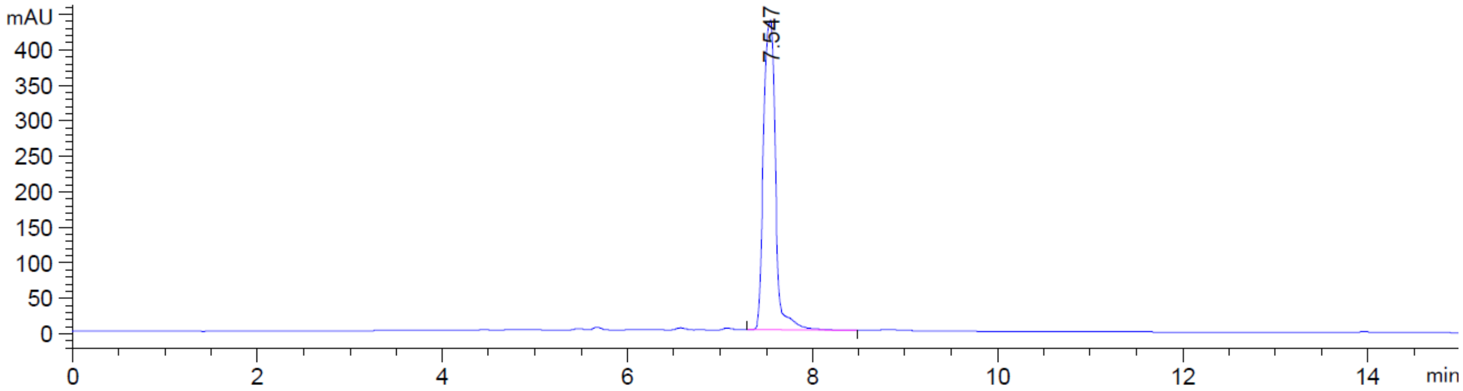

MS (-) ESI

(Calc. [M-H]$^-$ $C_{34}H_{45}Cl_2N_{15}O_{26}P_5^-$ 1304.07308)

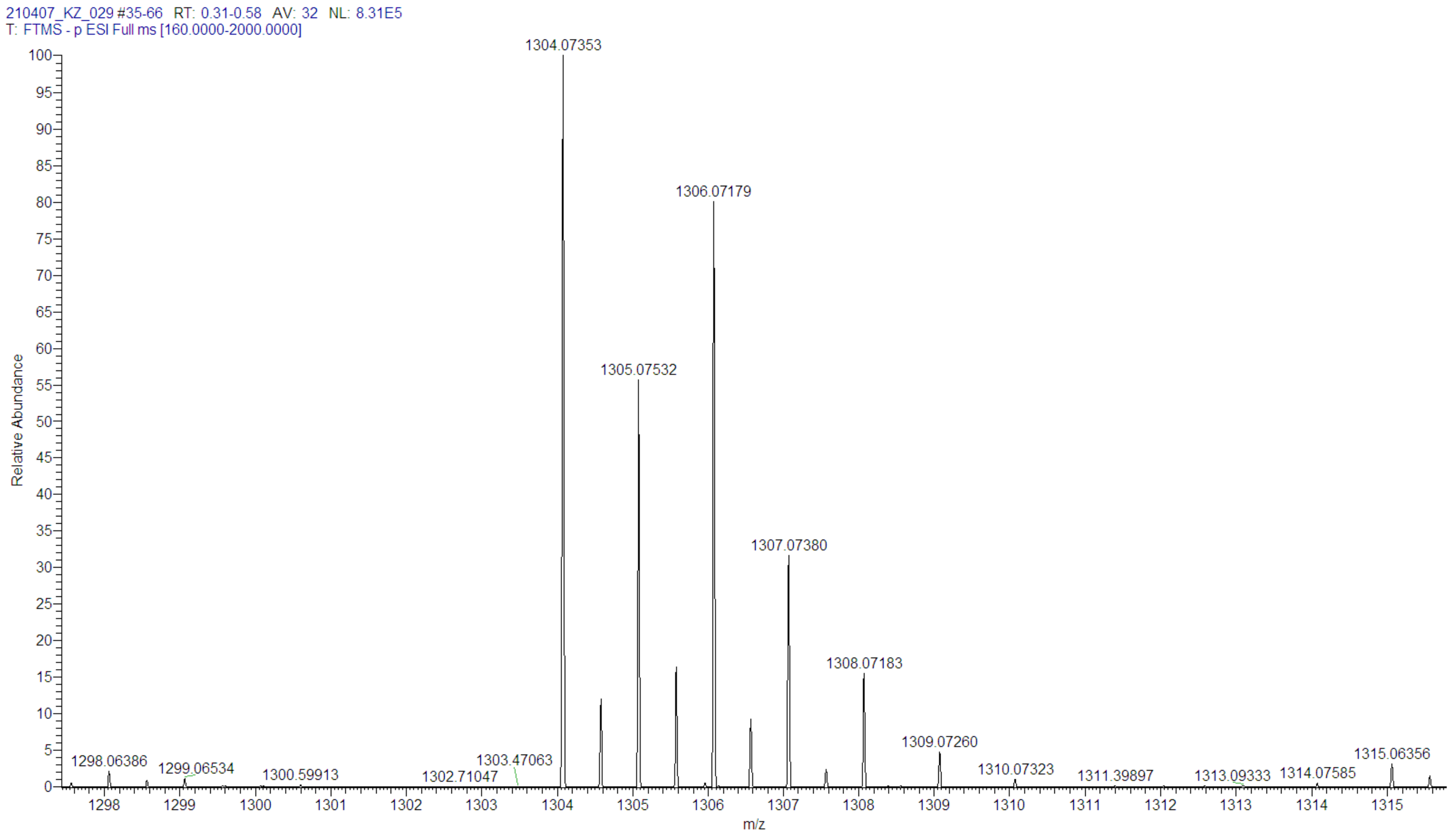

^1H NMR (500 MHz, D_2O, 25°C)

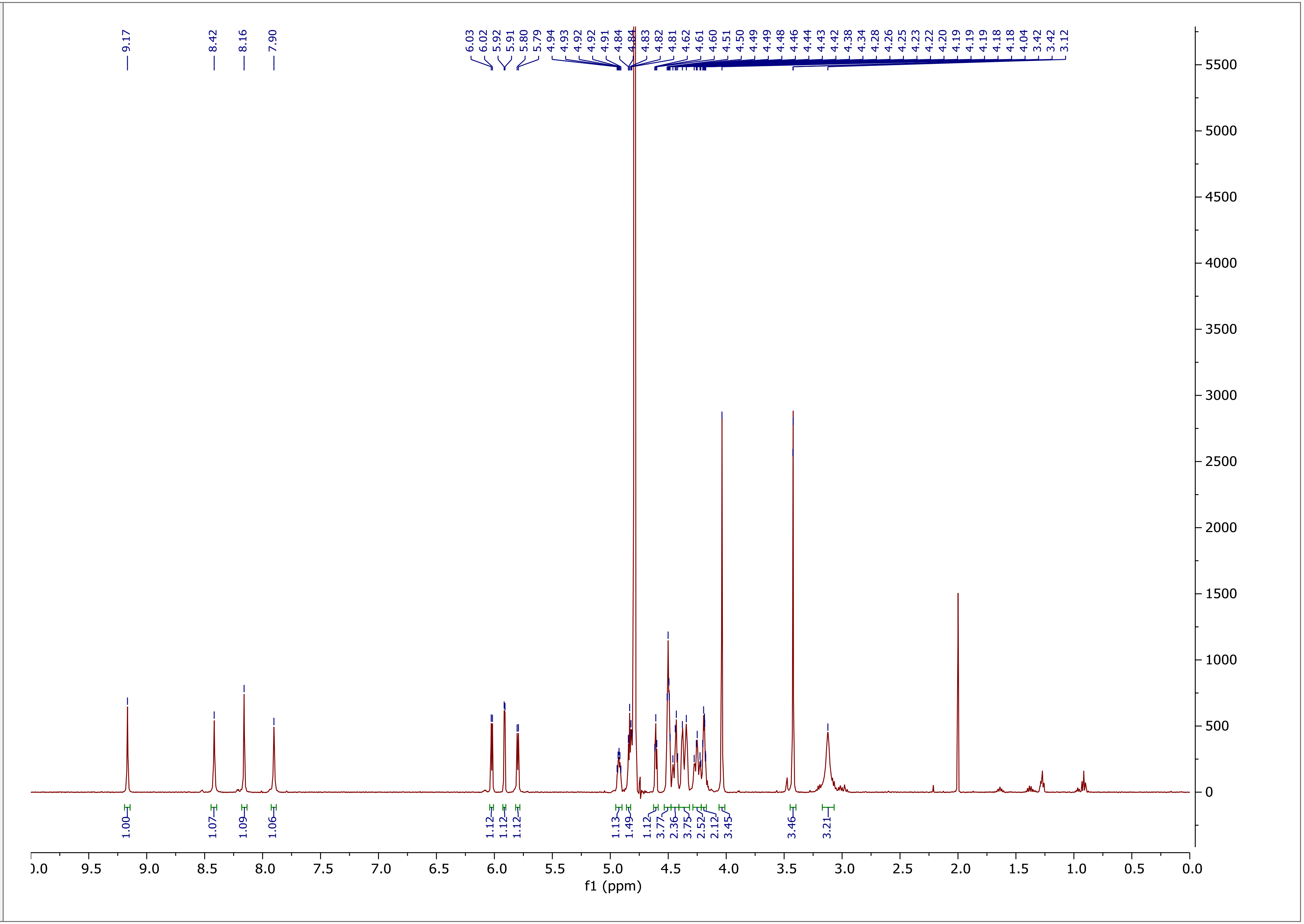

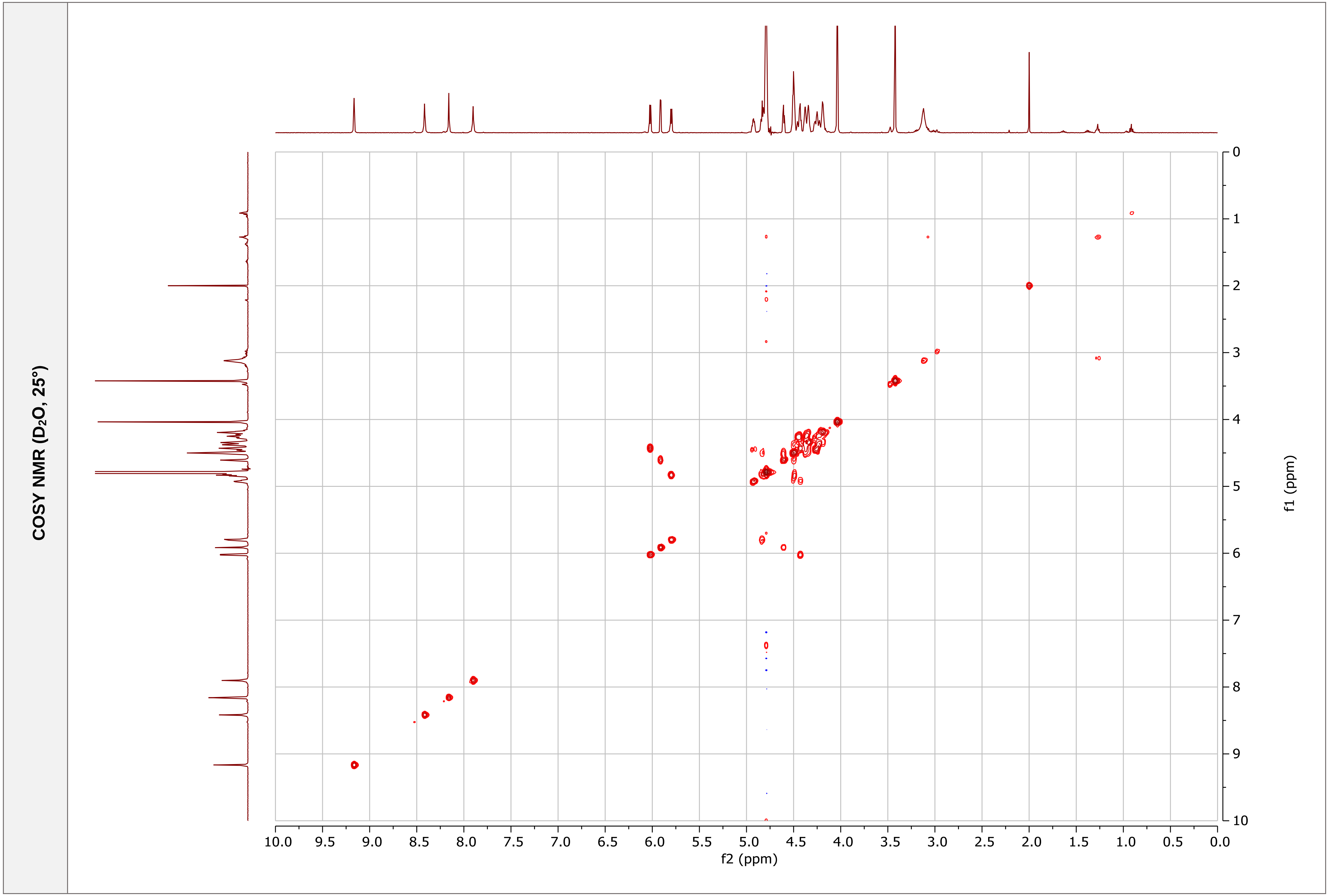
COSY NMR (D_2O, 25°)
f2 (ppm)
f1 (ppm)
10.0 9.5 9.0 8.5 8.0 7.5 7.0 6.5 6.0 5.5 5.0 4.5 4.0 3.5 3.0 2.5 2.0 1.5 1.0 0.5 0.0
0 1 2 3 4 5 6 7 8 9 10

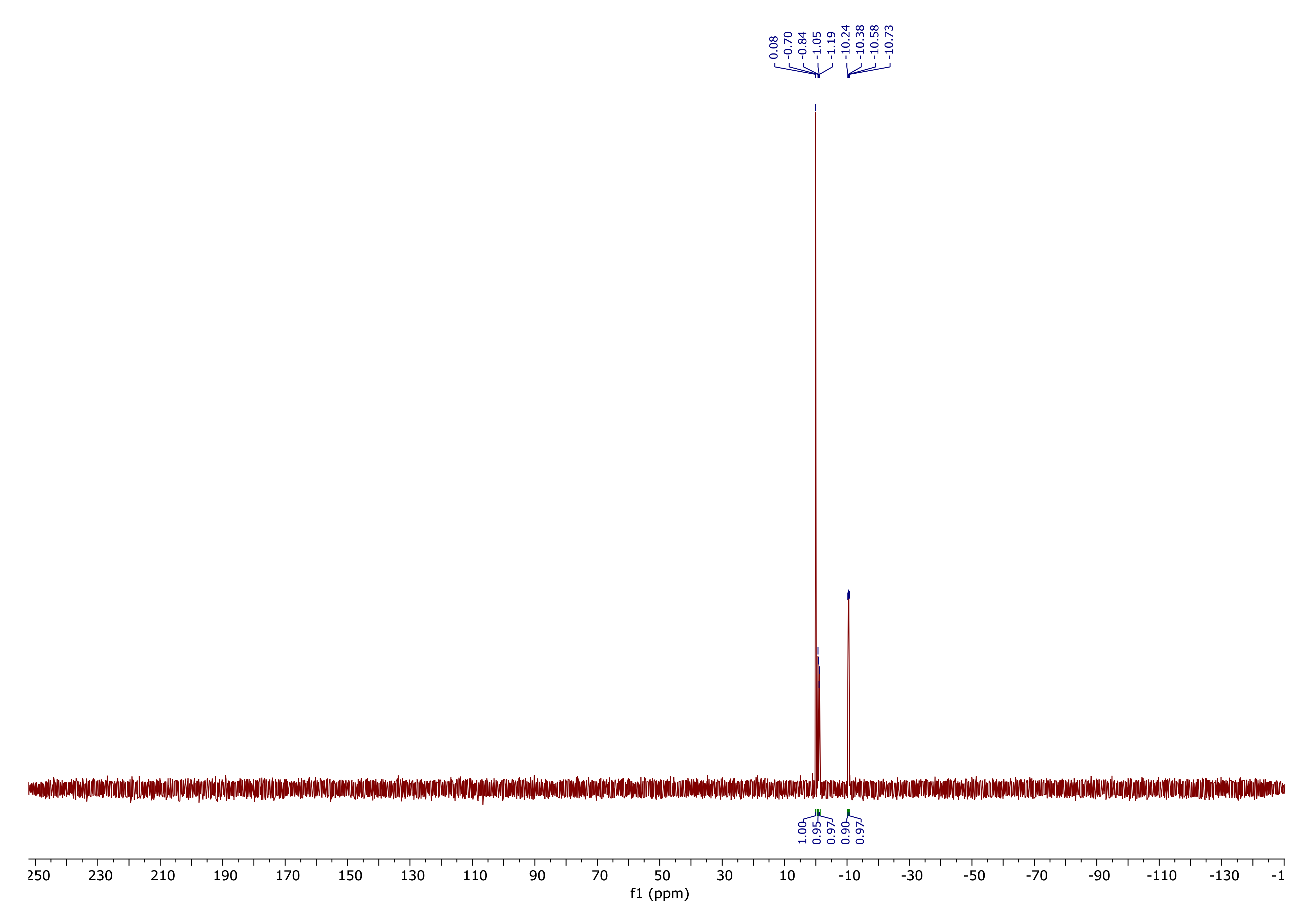

^{31}P NMR (202.5 MHz, D_2O, 25°C)

^{1}H-^{31}P HSQC (D_2O, 25°C)

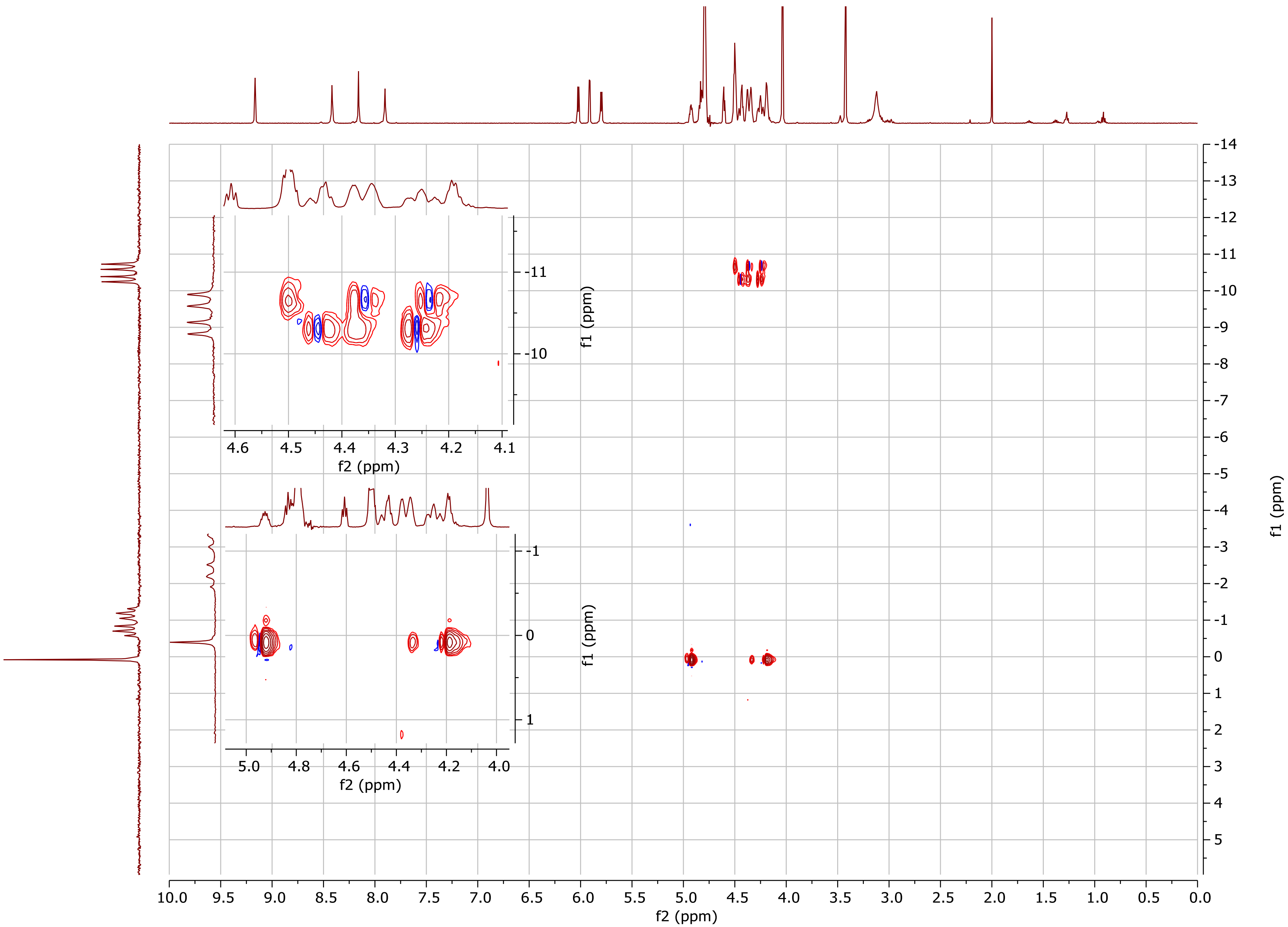

(16) m^{7}GppCH$_{2}$ppA$_{m}$pG

Chemical structure

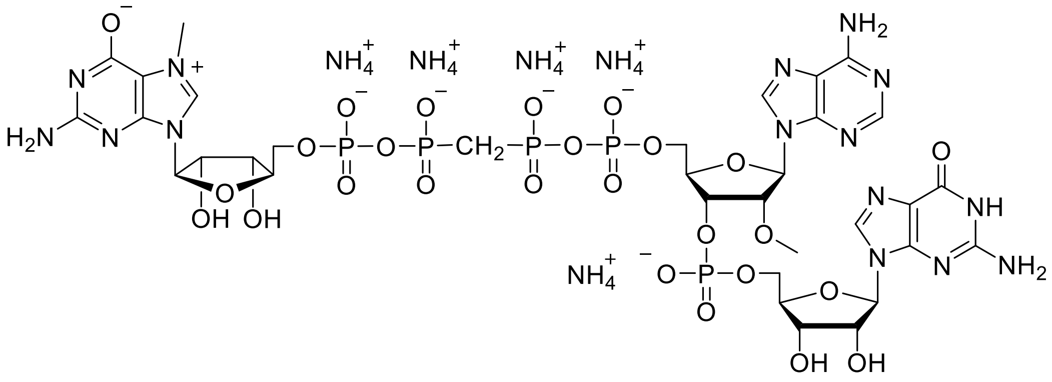

RP HPLC
Abs. @ 254 nm

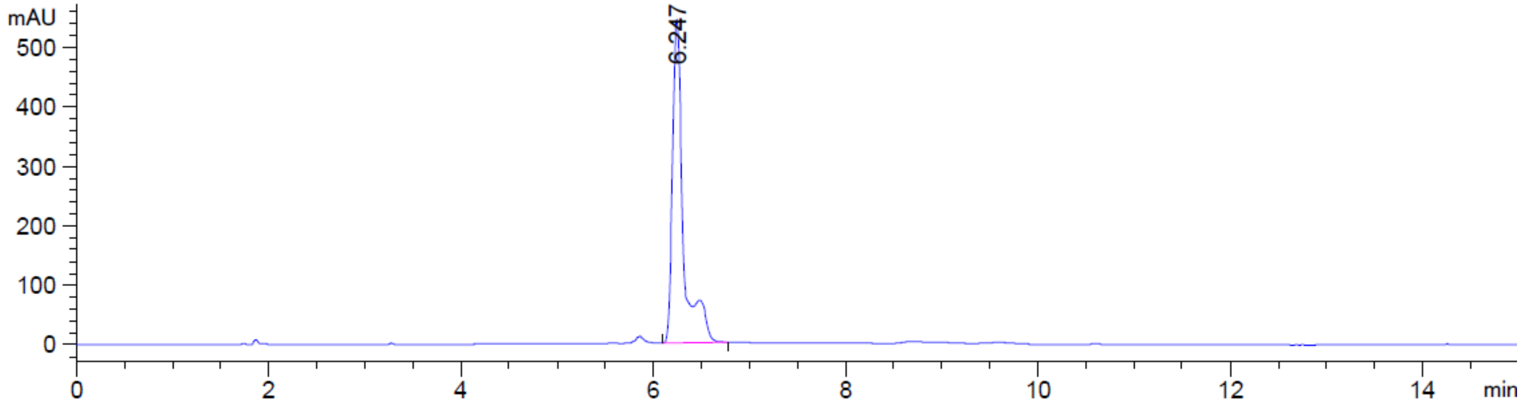

** Re-injection of the first part of the peak gives a peak of the same shape.*

MS (-) ESI

(Calc. $[M-H]^- C_{33}H_{45}N_{15}O_{26}P_5^-$ 1222.13537)

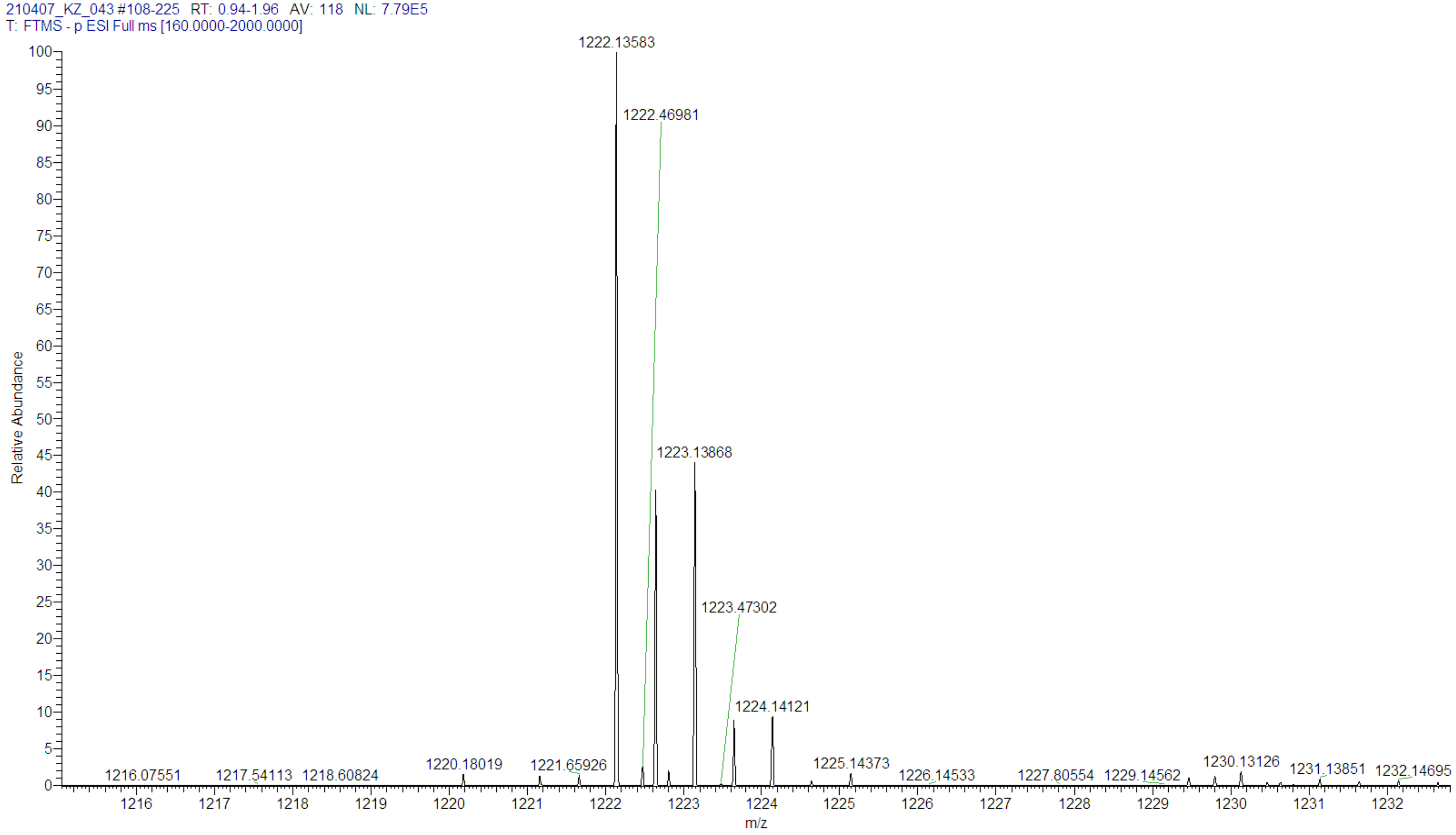

^1H NMR (500 MHz, D_2O, 25°C)

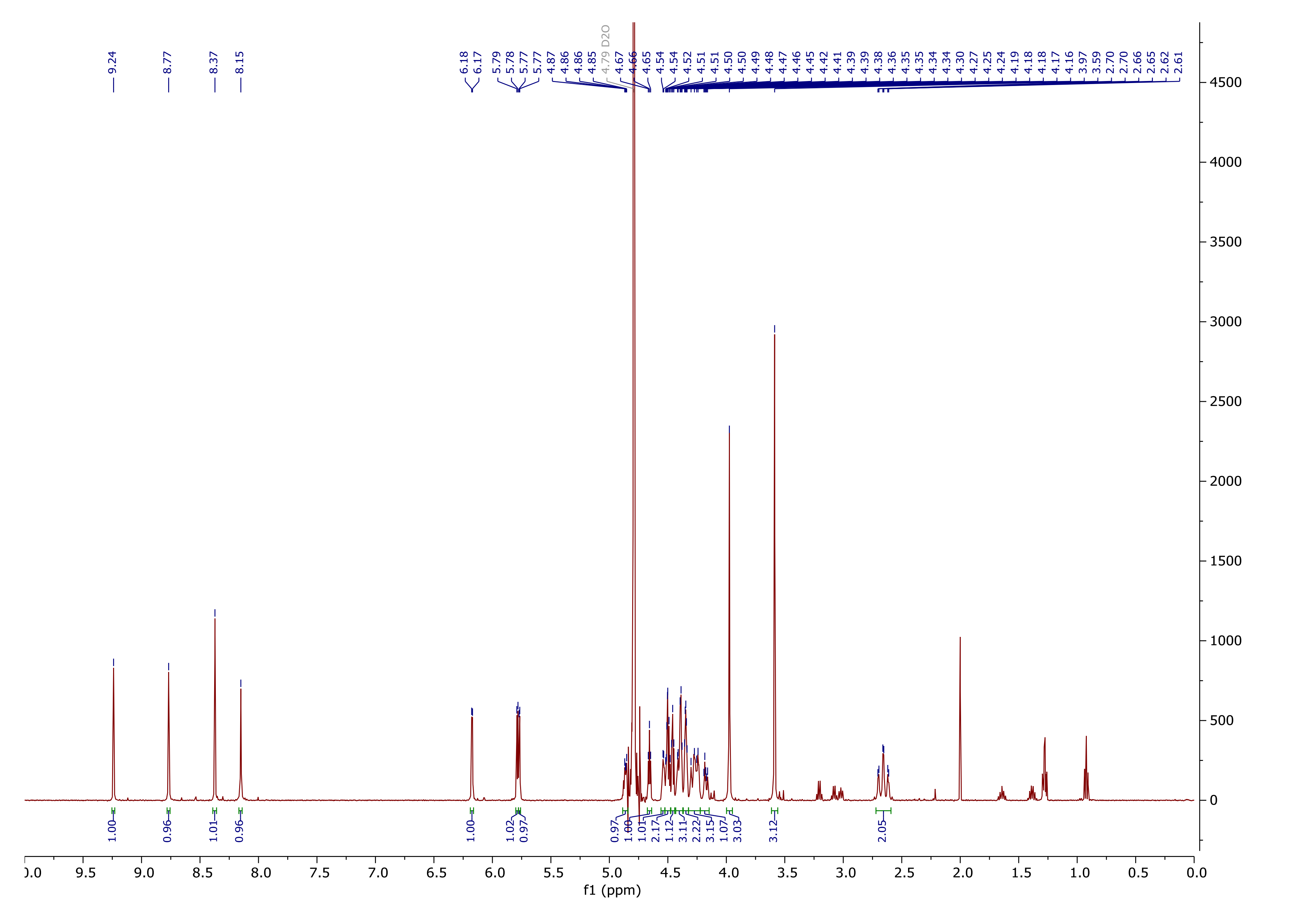

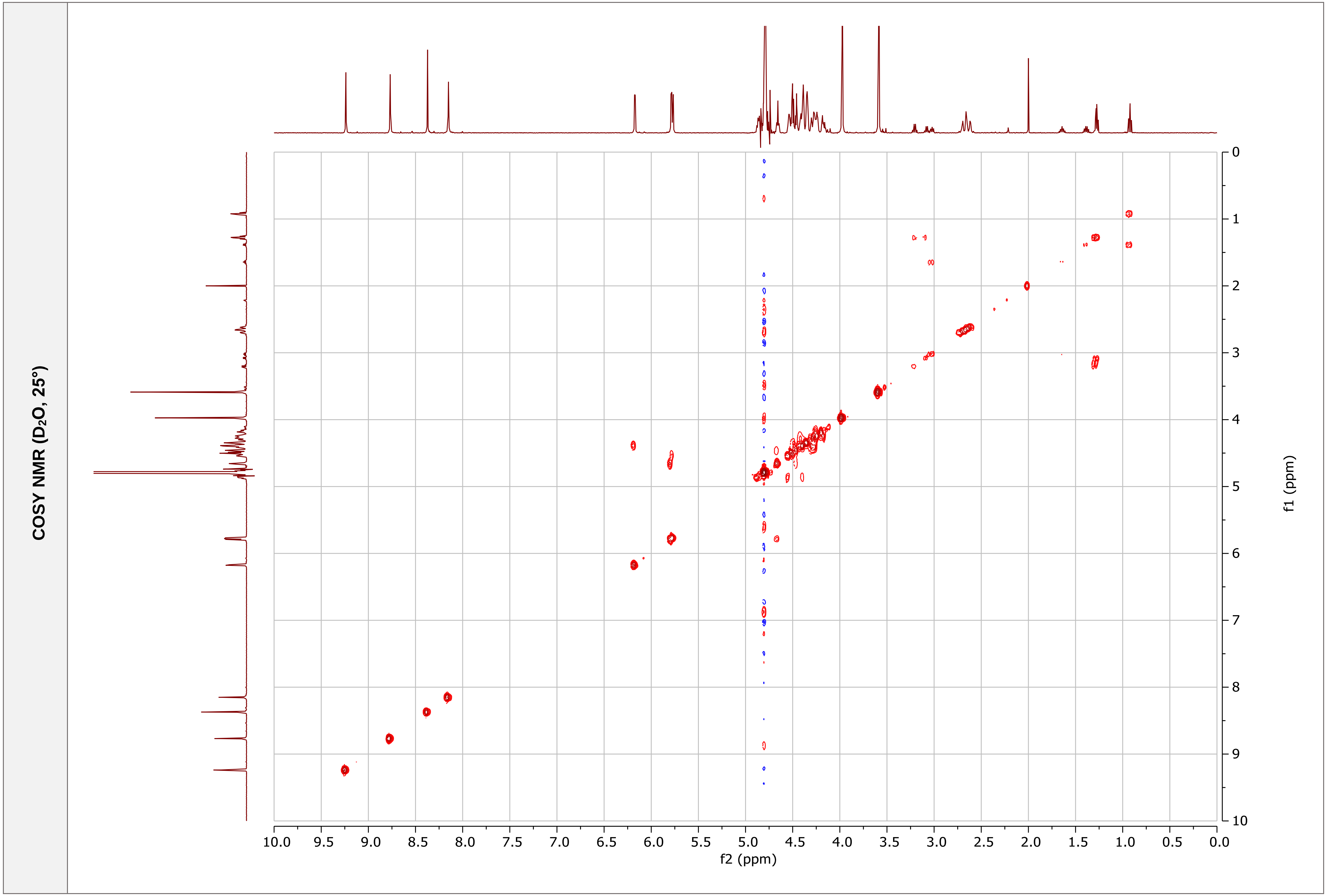

COSY NMR (D_2O, 25°)
f2 (ppm)
f1 (ppm)

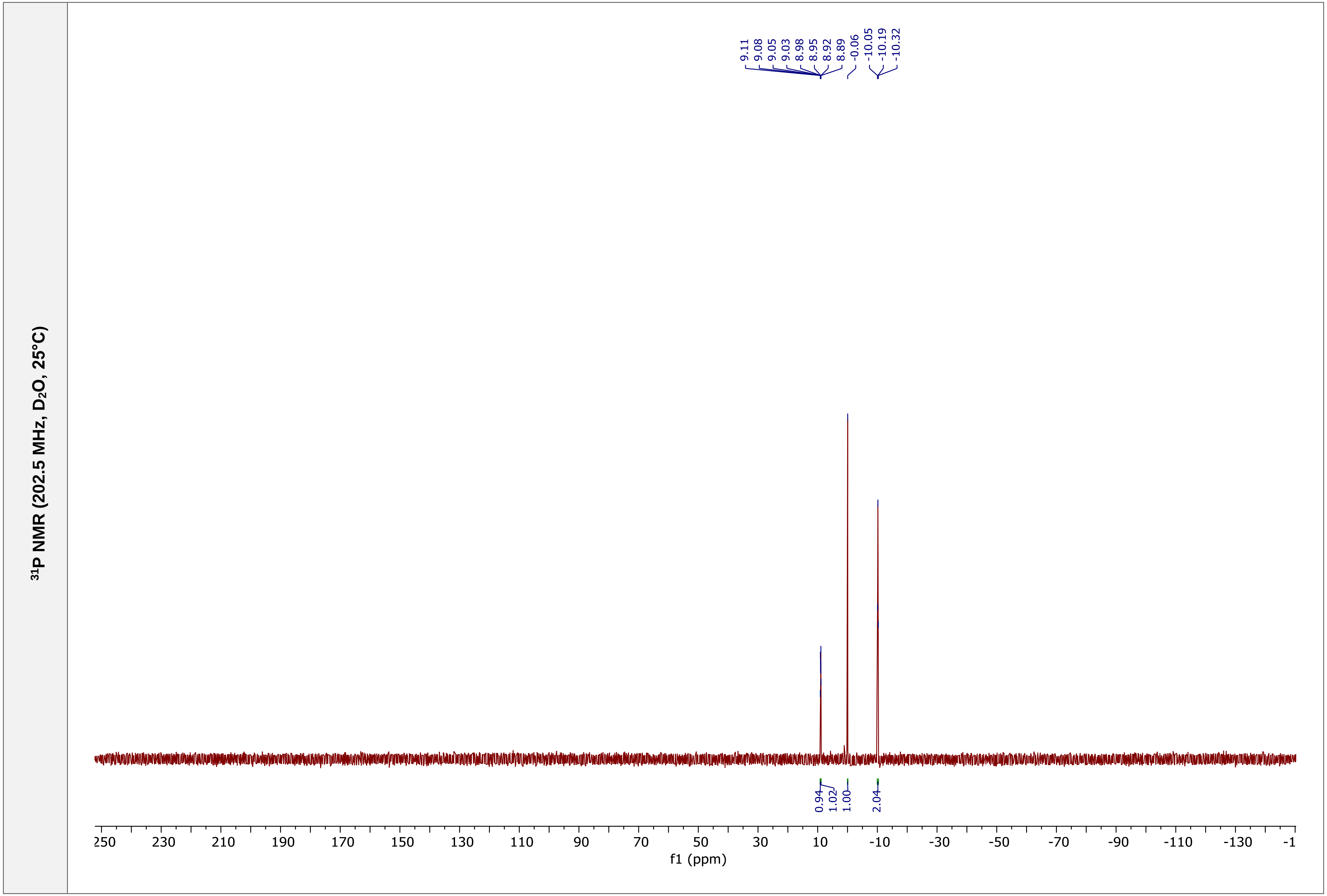

31P NMR (202.5 MHz, D2O, 25°C)
9.11
9.08
9.05
9.03
8.98
8.95
8.92
8.89
-0.06
-10.05
-10.19
-10.32
0.94
1.02
1.00
2.04
250
230
210
190
170
150
130
110
90
70
50
30
10
-10
-30
-50
-70
-90
-110
-130
f1 (ppm)

^{1}H-^{31}P HSQC (D_2O, 25°C)

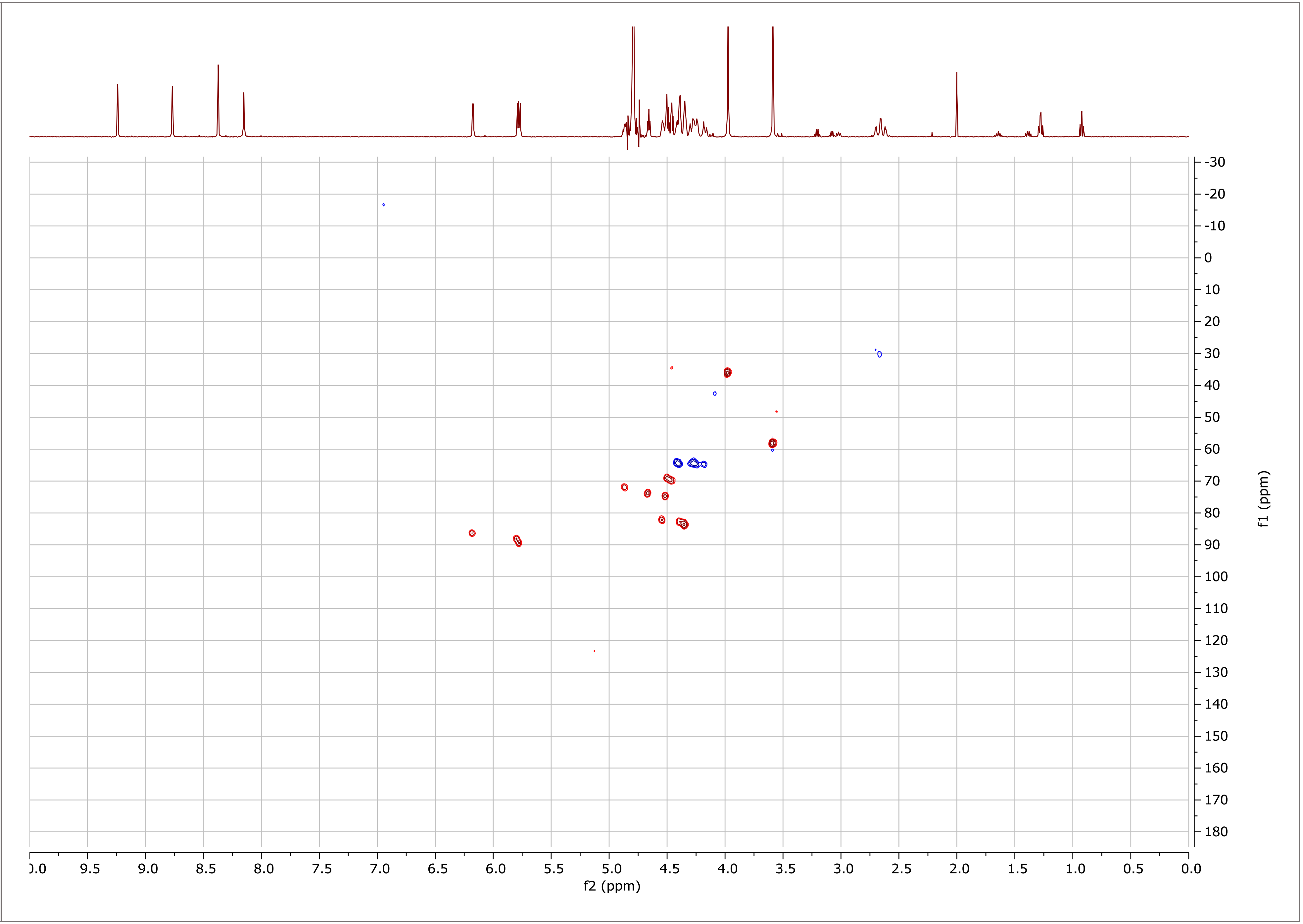

^1H-^{31}P HSQC

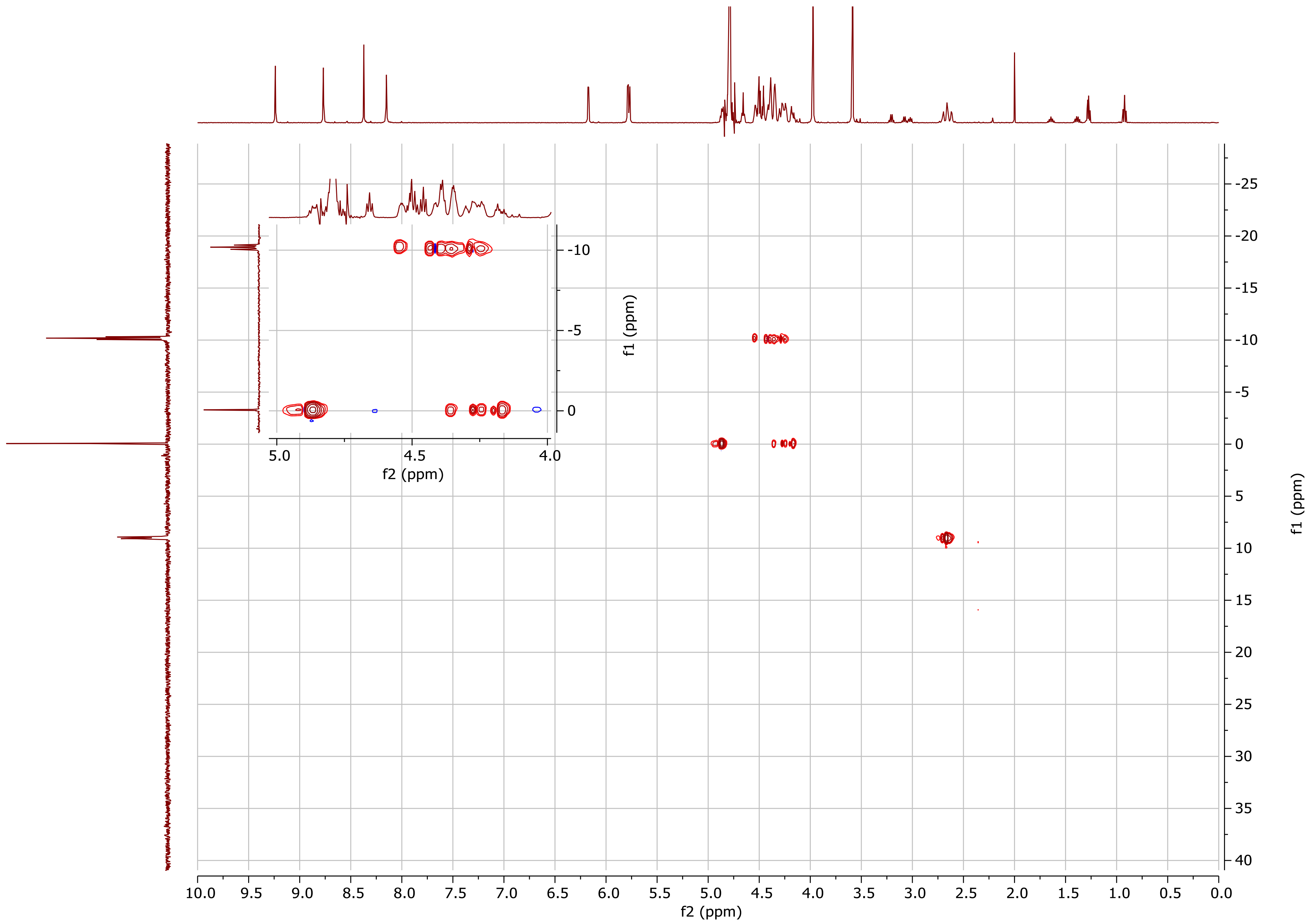

(17) m^{7}GpppA$_m$pG-L13$_N$

Chemical structure

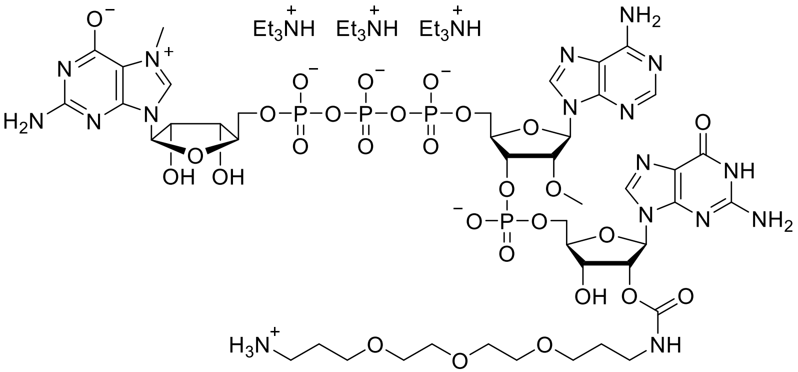

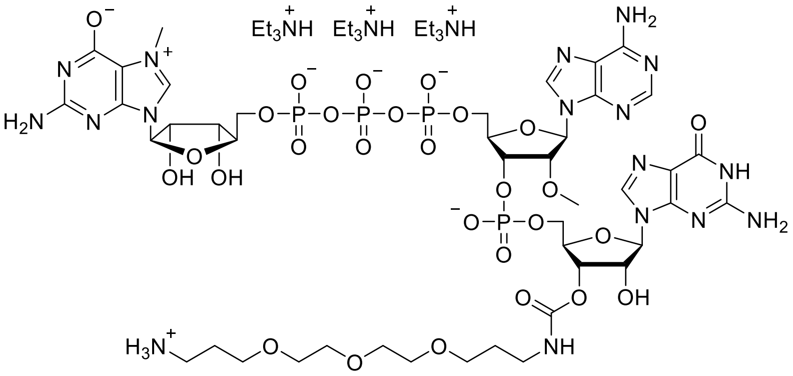

RP HPLC

Abs. @ 254 nm

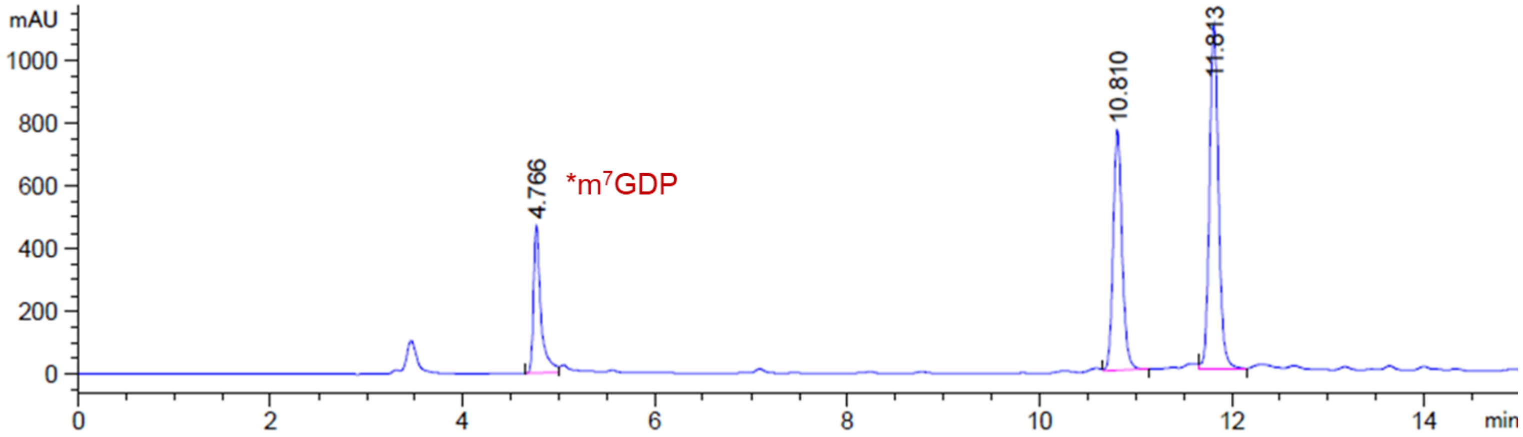

MS (-) ESI: Isomer 1

(Calc. [M-H]$^-$ $C_{43}H_{64}N_{17}O_{28}P_4^-$ 1390.30626)

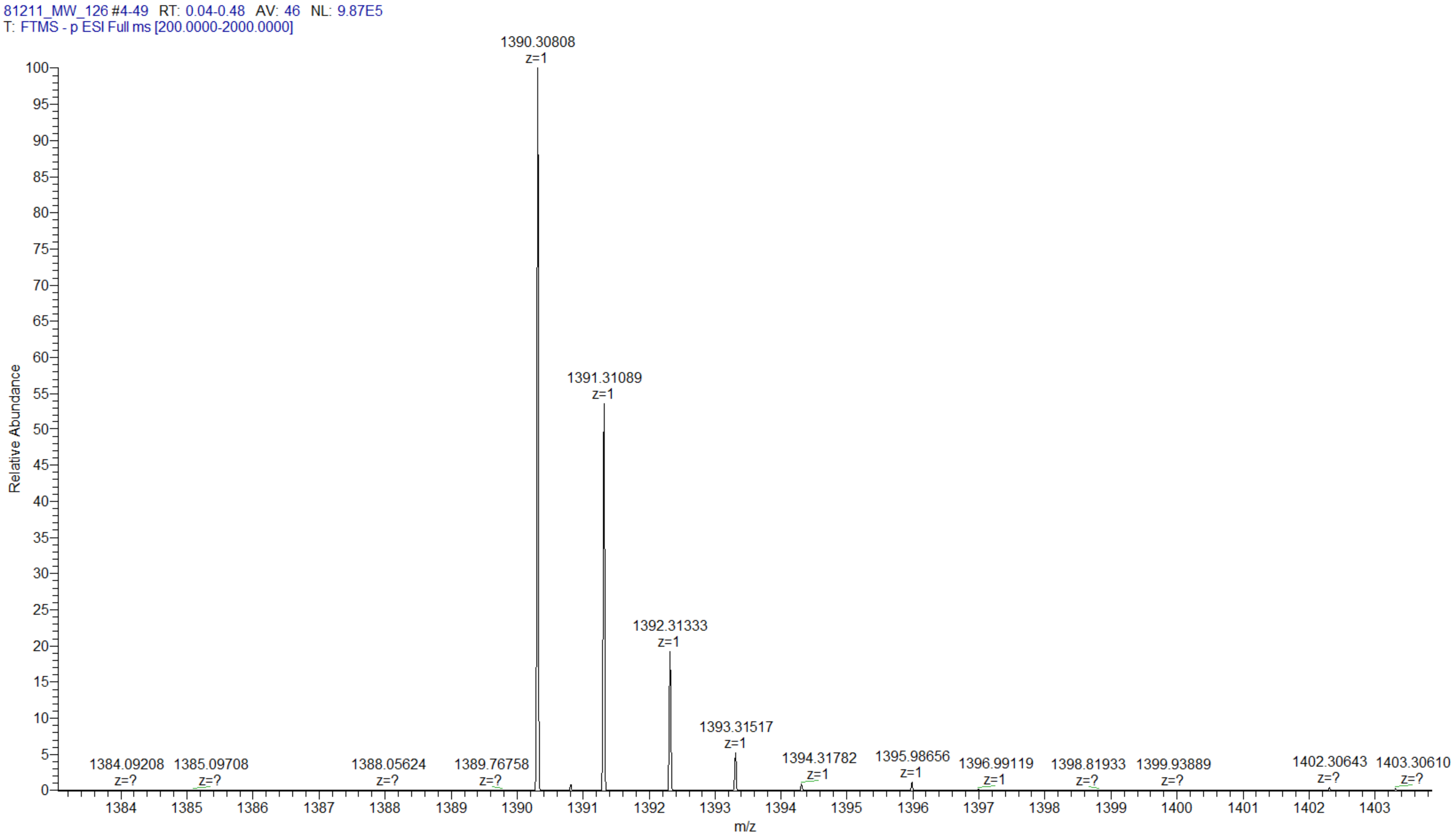

MS (-) ESI: Isomer 2

(Calc. $[M-H]^-$ $C_{43}H_{64}N_{17}O_{28}P_4^-$ 1390.30626)

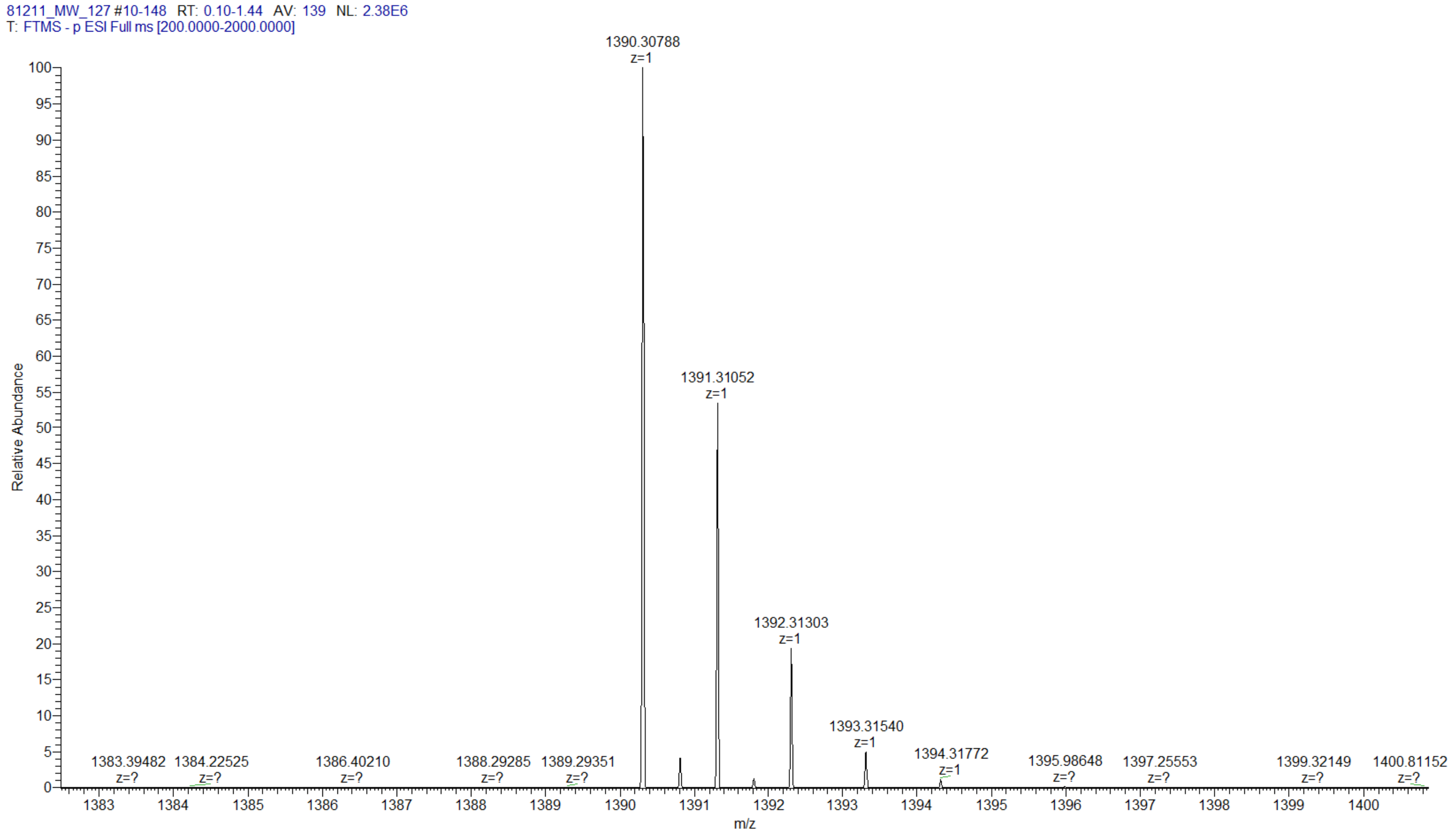

**(18) m^{7}GppppA$_m$pG-L13$_N$**	
**Chemical structure**	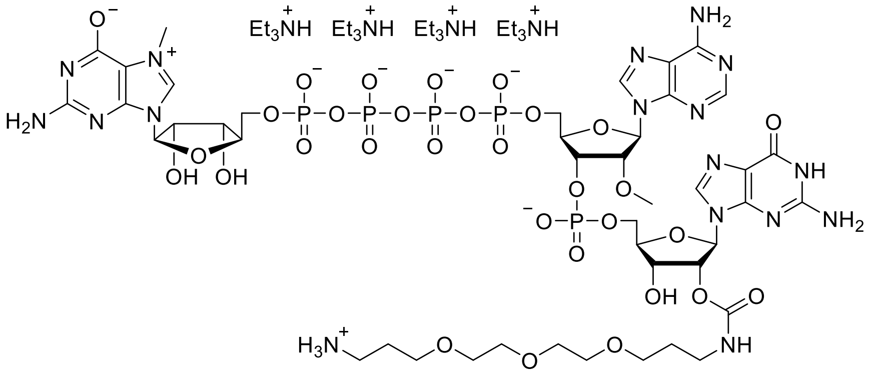 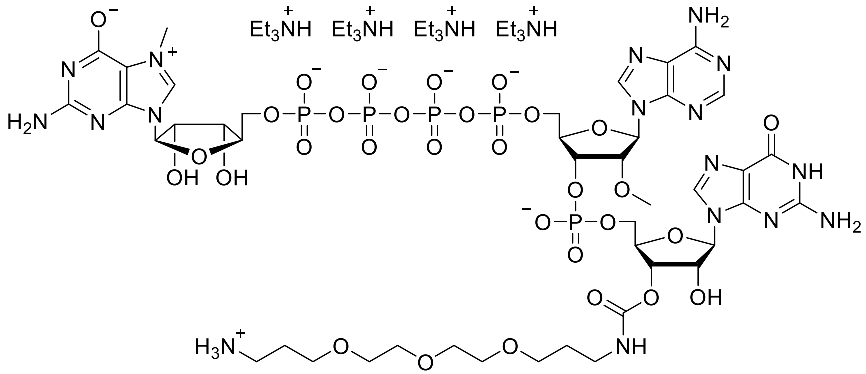
**RP HPLC** Abs. @ 254 nm	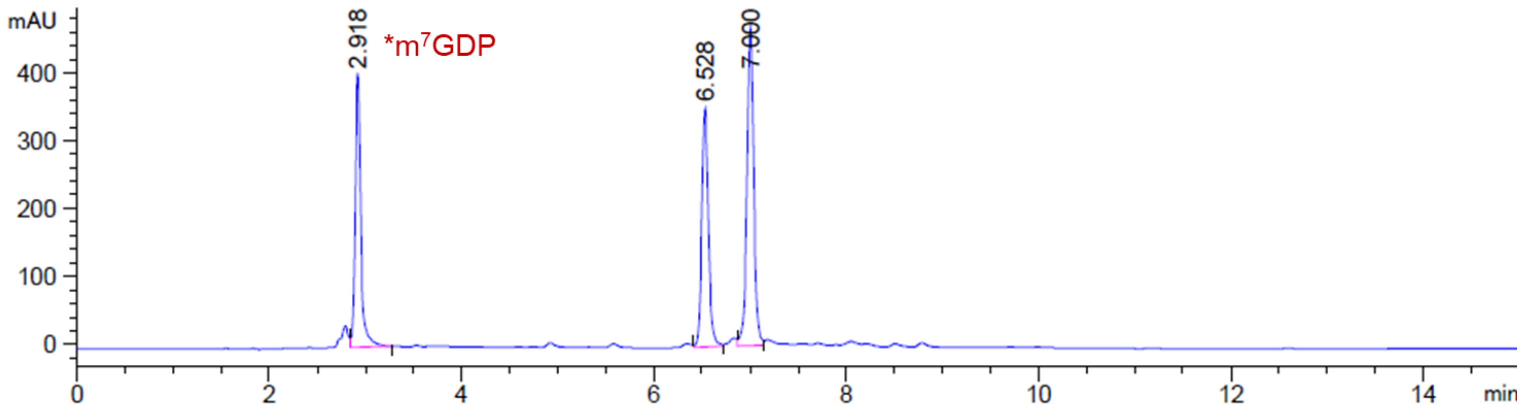

MS (-) ESI: Isomer 1

(Calc. $[M-H]^-$ $C_{43}H_{65}N_{17}O_{31}P_5^-$ 1470.27259)

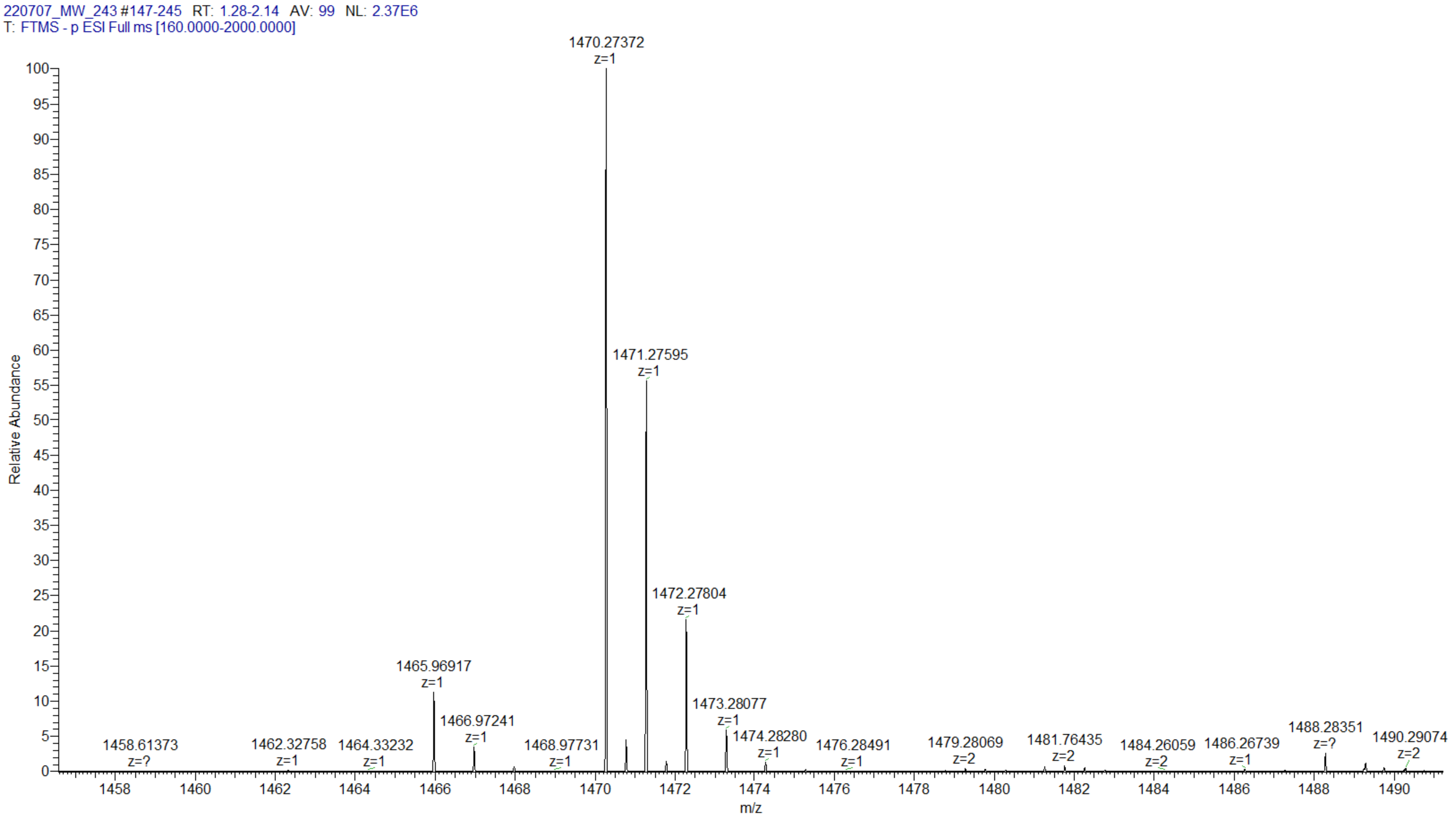

MS (-) ESI: Isomer 2

(Calc. $[M-H]^-$ $C_{43}H_{65}N_{17}O_{31}P_5^-$ 1470.27259)

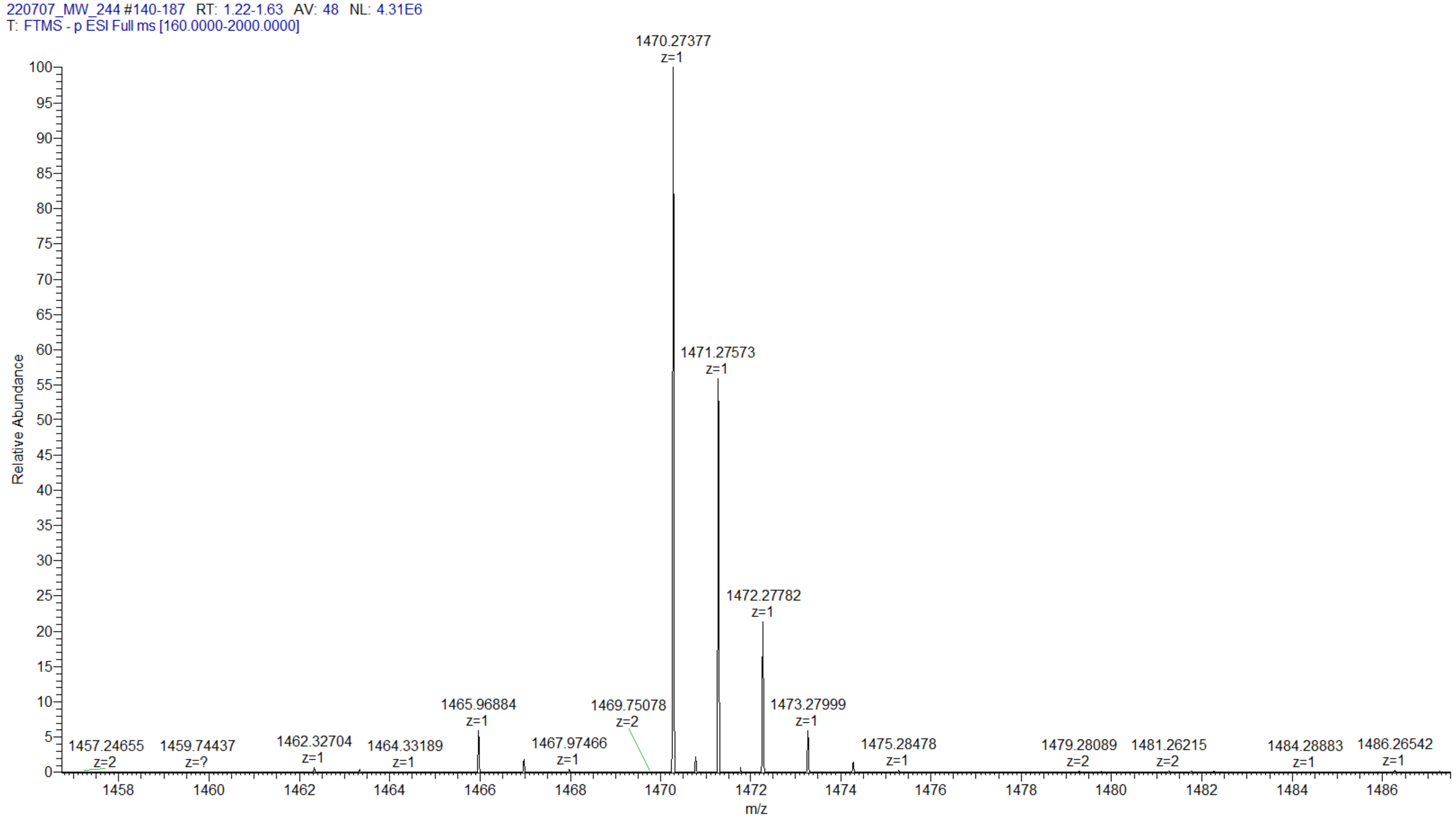

(19) $m^{7}GppCCl_{2}ppA_{m}pG\text{-}L13_{N}$	
**Chemical structure**	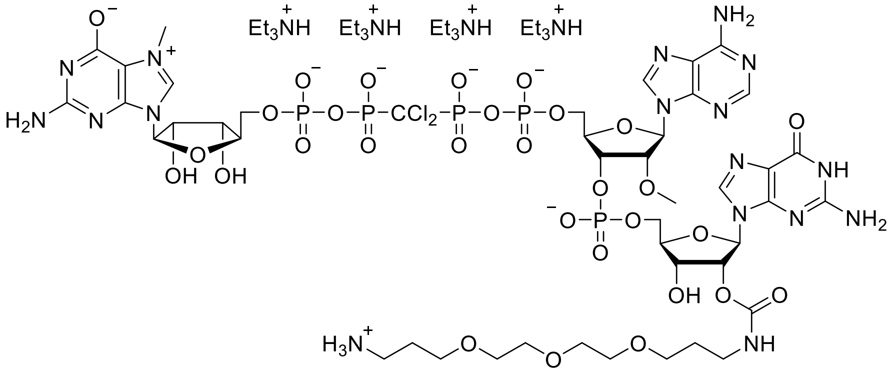 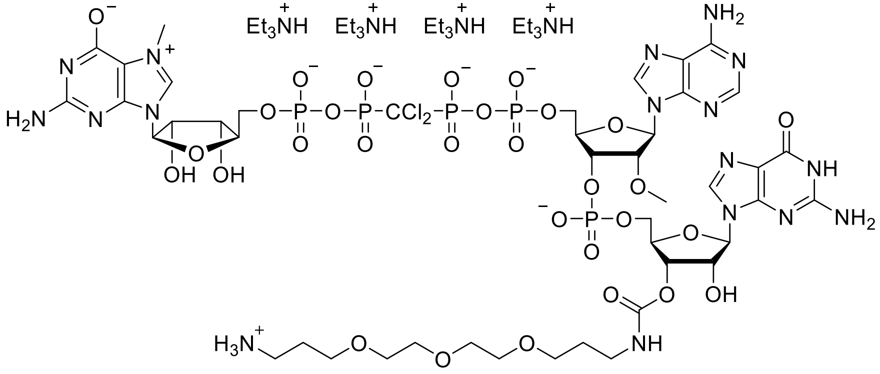
**RP HPLC** Abs. @ 254 nm	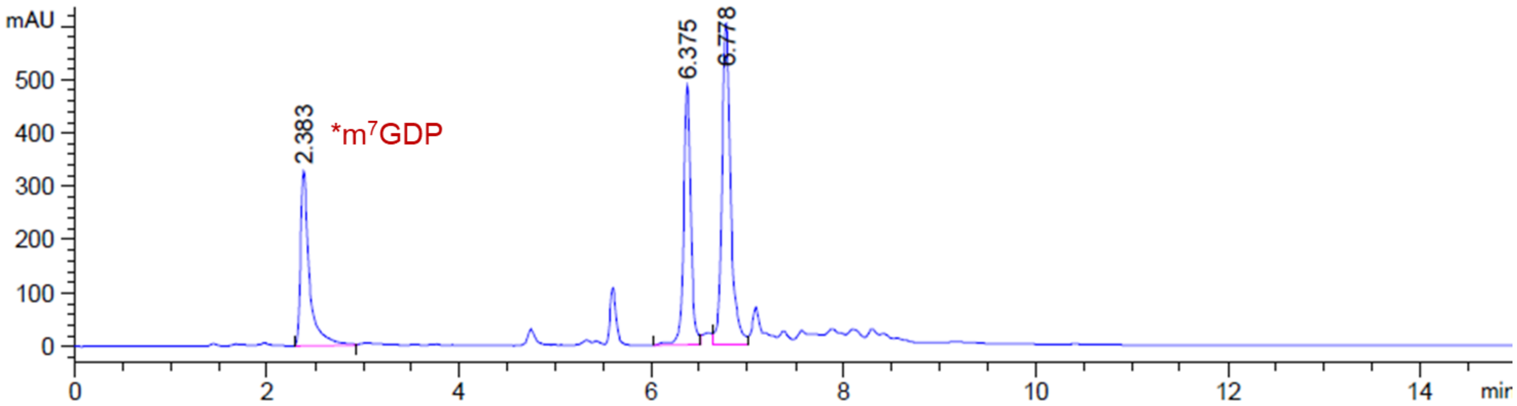

MS (-) ESI: Isomer 1

(Calc. [M-H]$^-$ $C_{44}H_{65}Cl_2N_{17}O_{31}P_5^-$ 1536.21539)

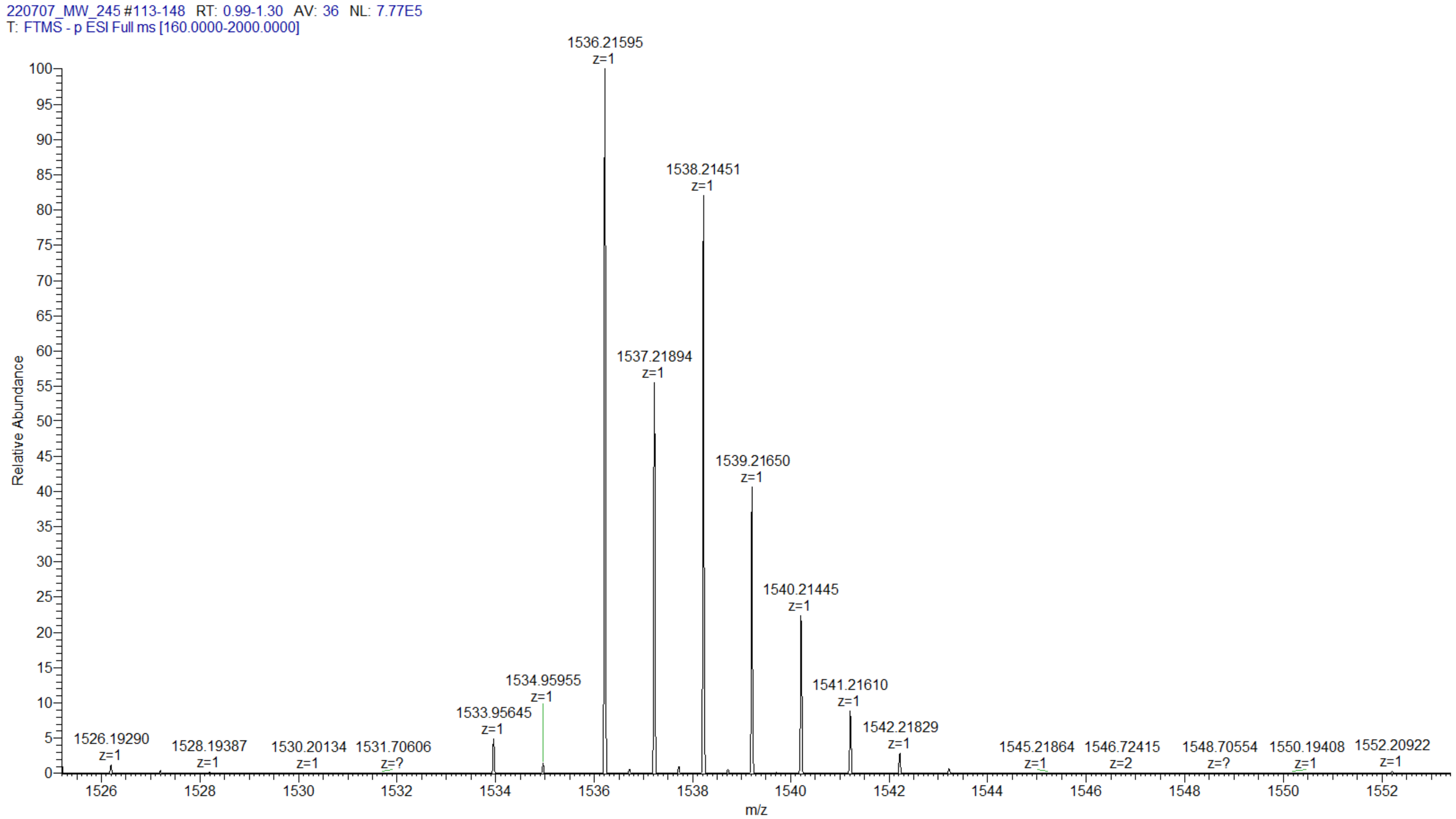

MS (-) ESI: Isomer 2

(Calc. $[M-H]^-$ $C_{44}H_{65}Cl_2N_{17}O_{31}P_5^-$ 1536.21539)

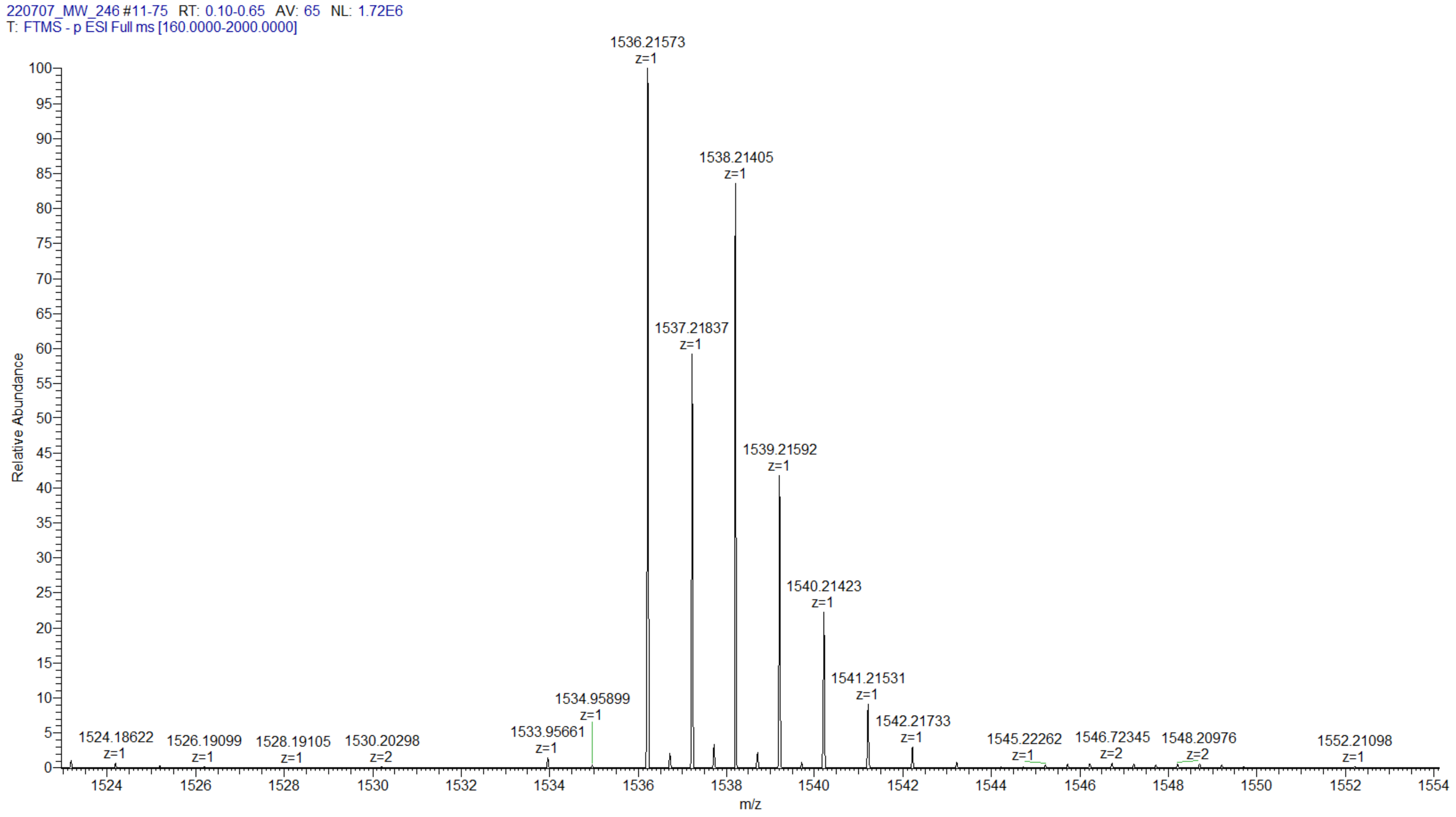

(20) pApG	
**Chemical structure**	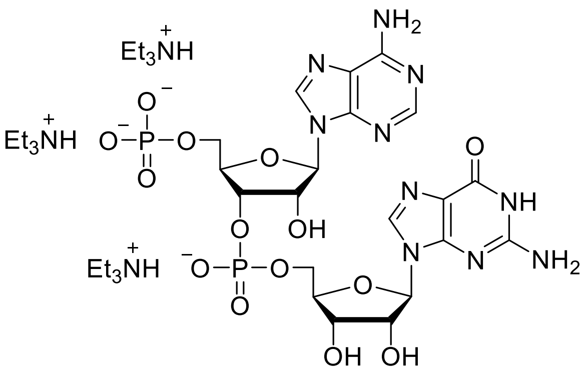
**RP HPLC** Abs. @ 254 nm	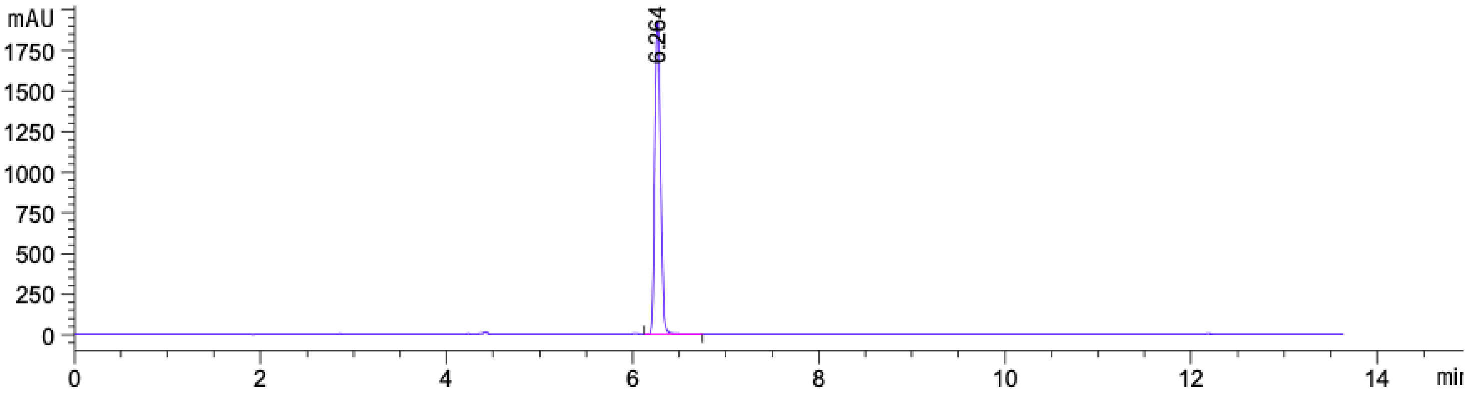

MS (-) ESI

(Calc. $[M\text{-}H]^-$ $C_{20}H_{25}N_{10}O_{14}P_2^-$ 691.10324)

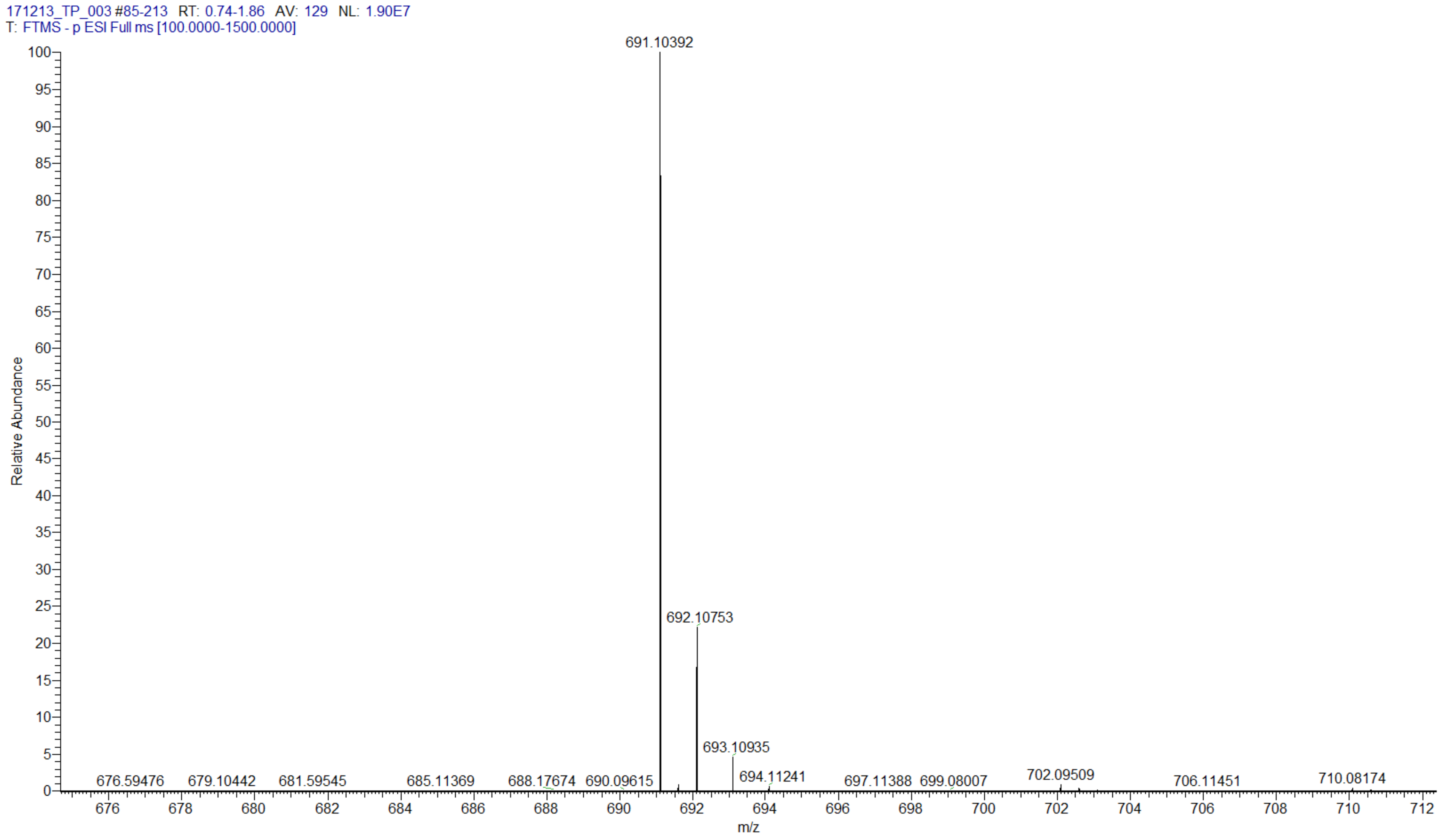

(21) pA$_m$pG	
**Chemical structure**	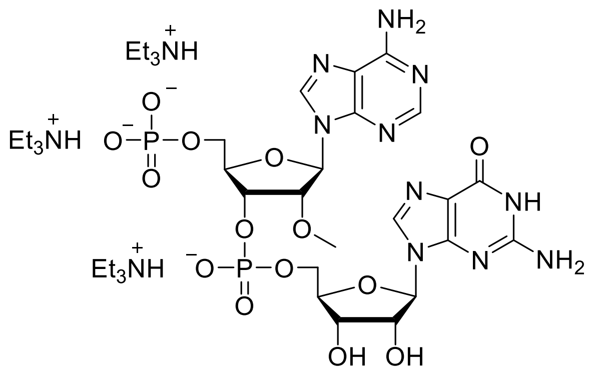
**RP HPLC** Abs. @ 254 nm	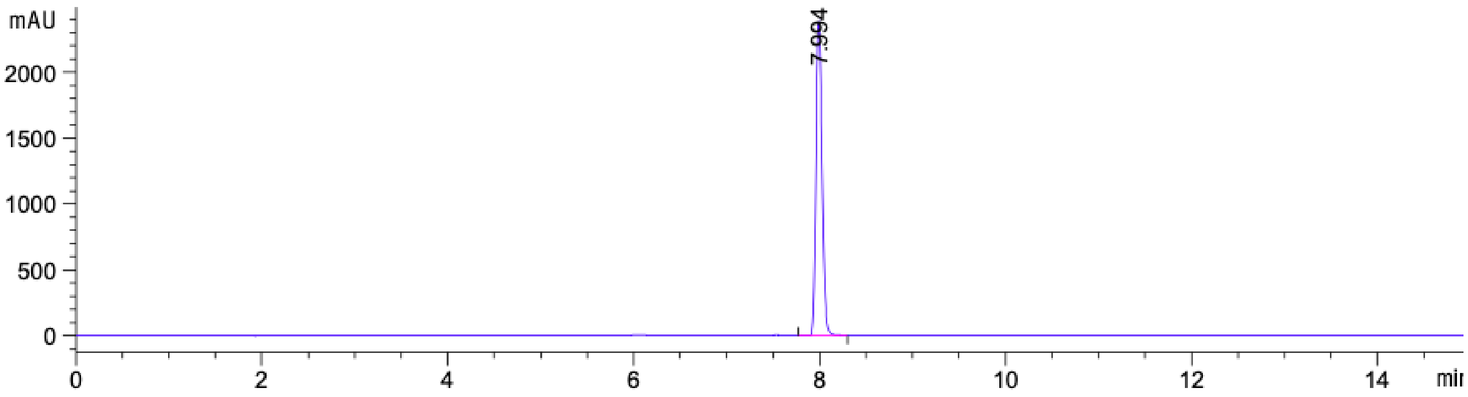

MS (-) ESI

(Calc. [M-H]$^-$ $C_{21}H_{27}N_{10}O_{14}P_2^-$ 705.11889)

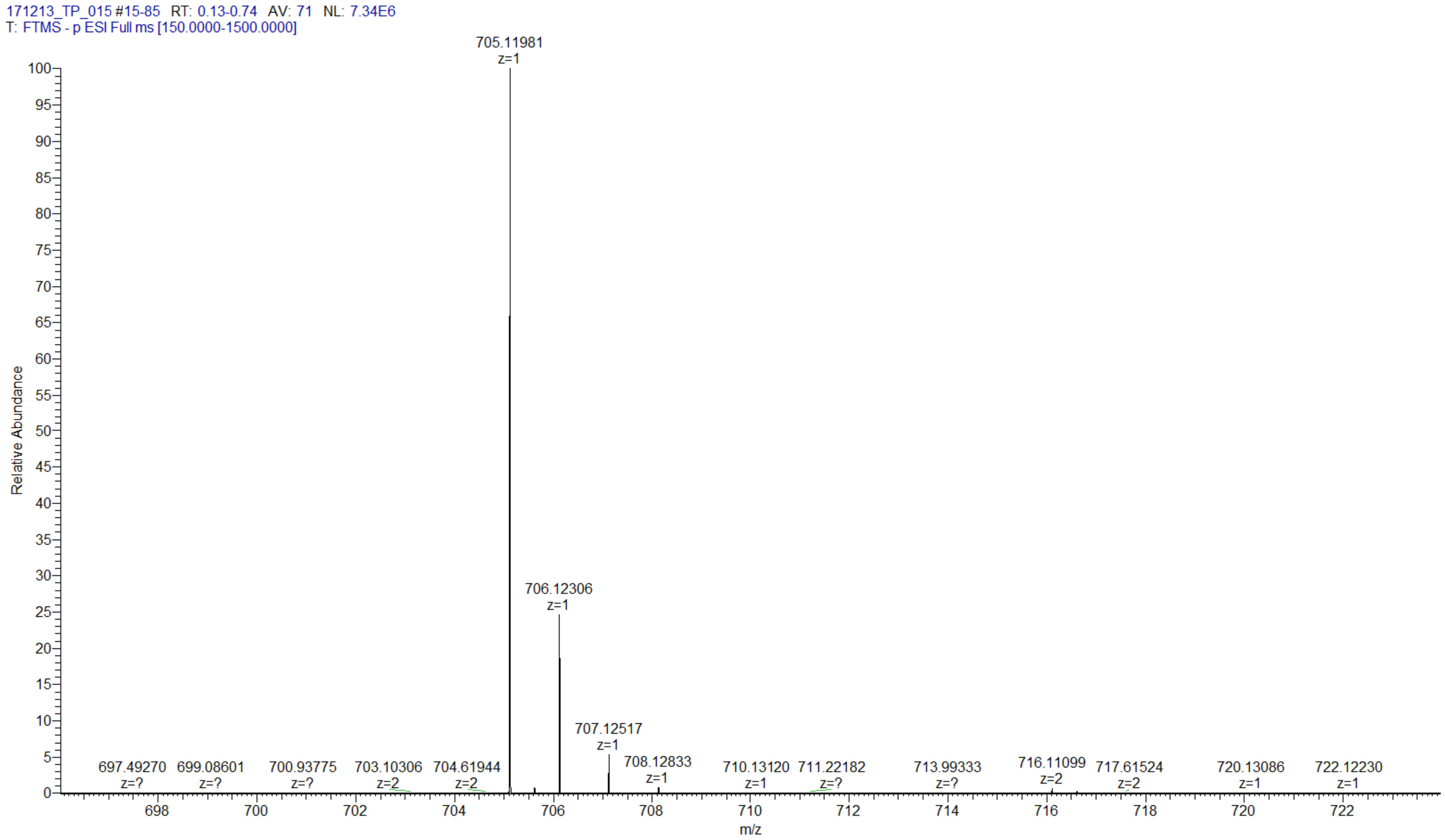

(22) $p^{m6}A_mpG$	
**Chemical structure**	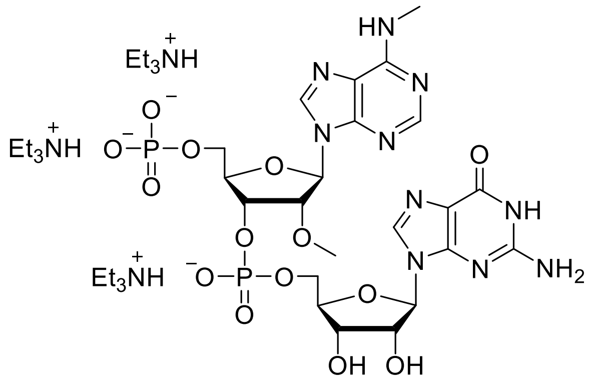
**RP HPLC** Abs. @ 254 nm	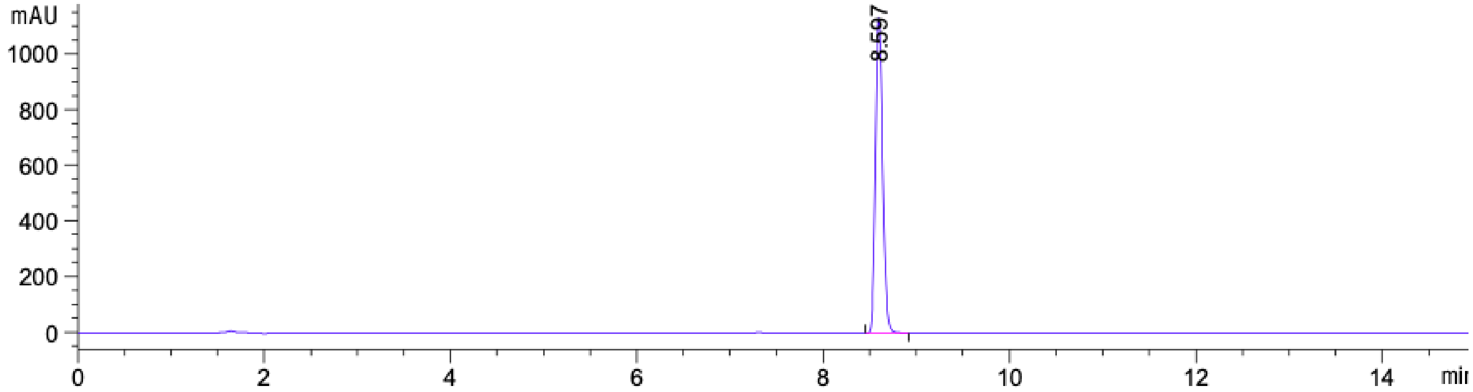

MS (-) ESI

(Calc. $[M-H]^-$ $C_{22}H_{29}N_{10}O_{14}P_2^-$ 719.13454)

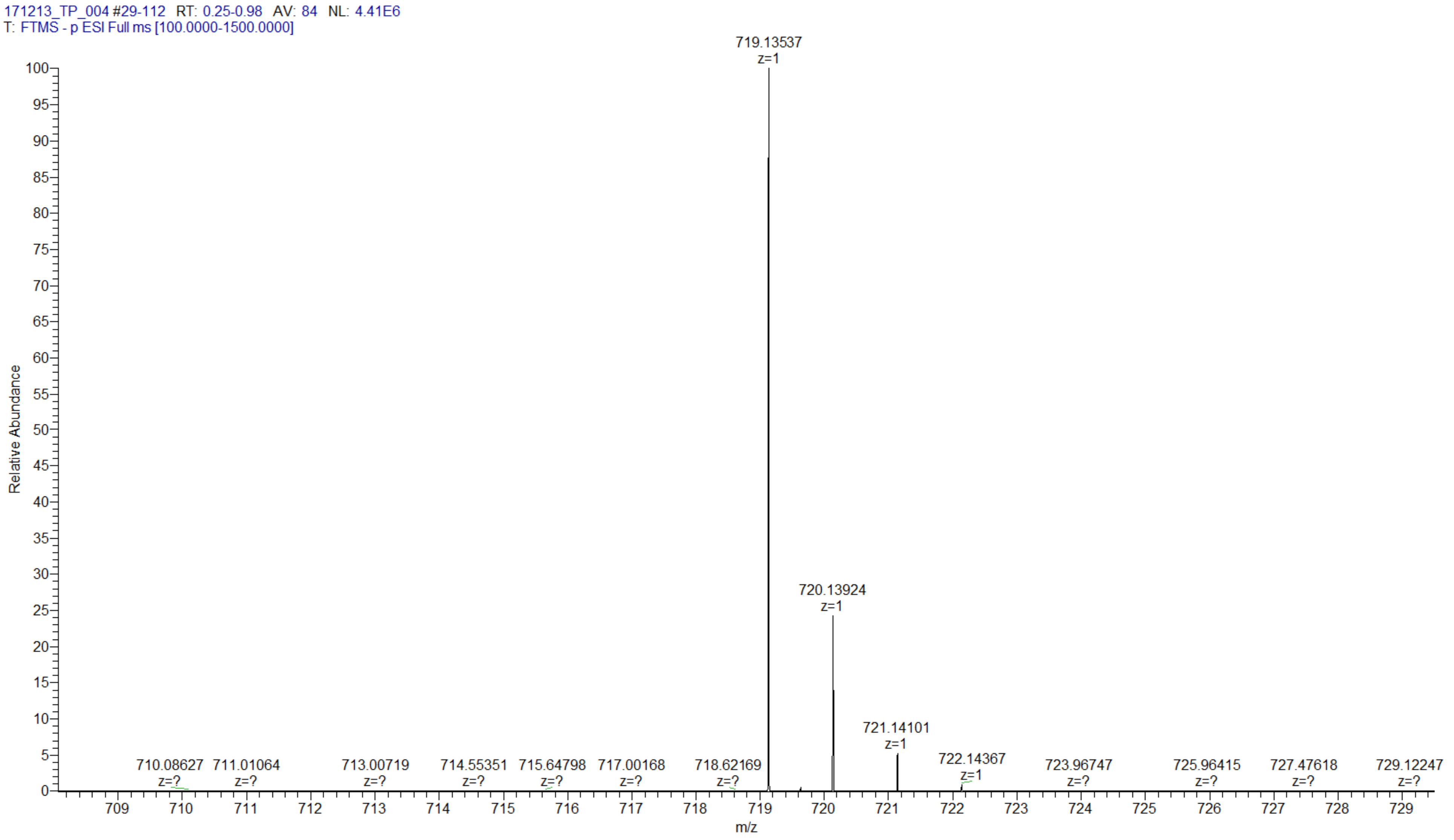

(23) p$^{5'S}$ApG	
**Chemical structure**	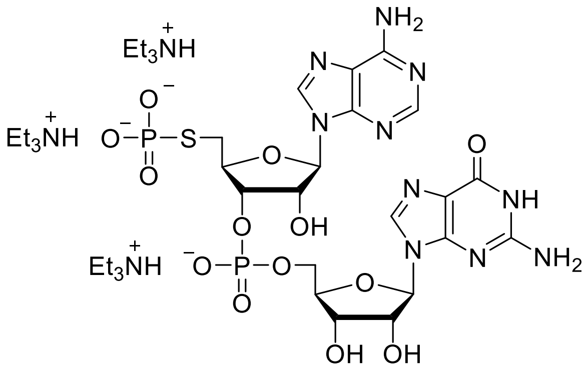
**RP HPLC** Abs. @ 254 nm	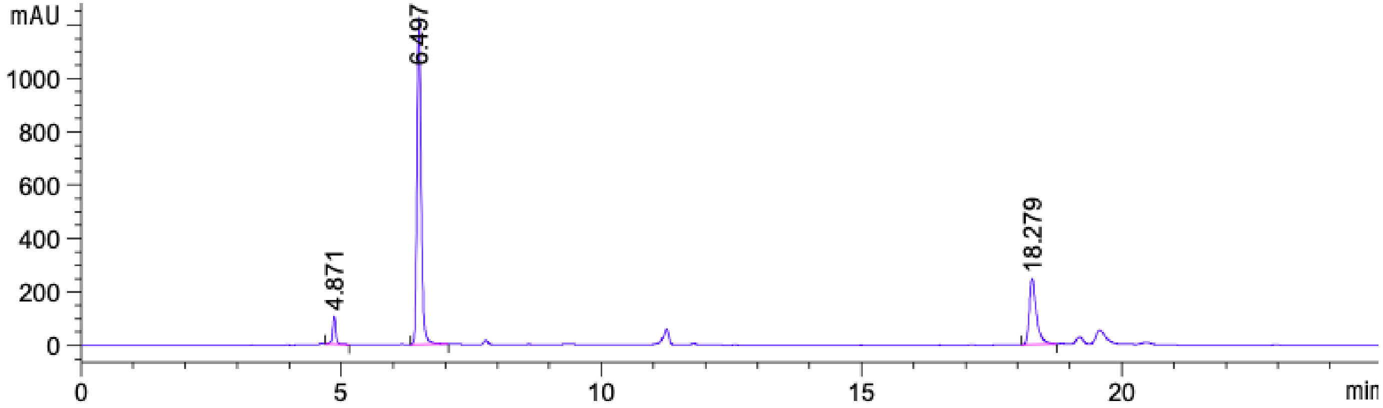

MS (-) ESI
(Calc. [M-H]$^-$ $C_{20}H_{25}N_{10}O_{13}P_2S^-$ 707.08040)

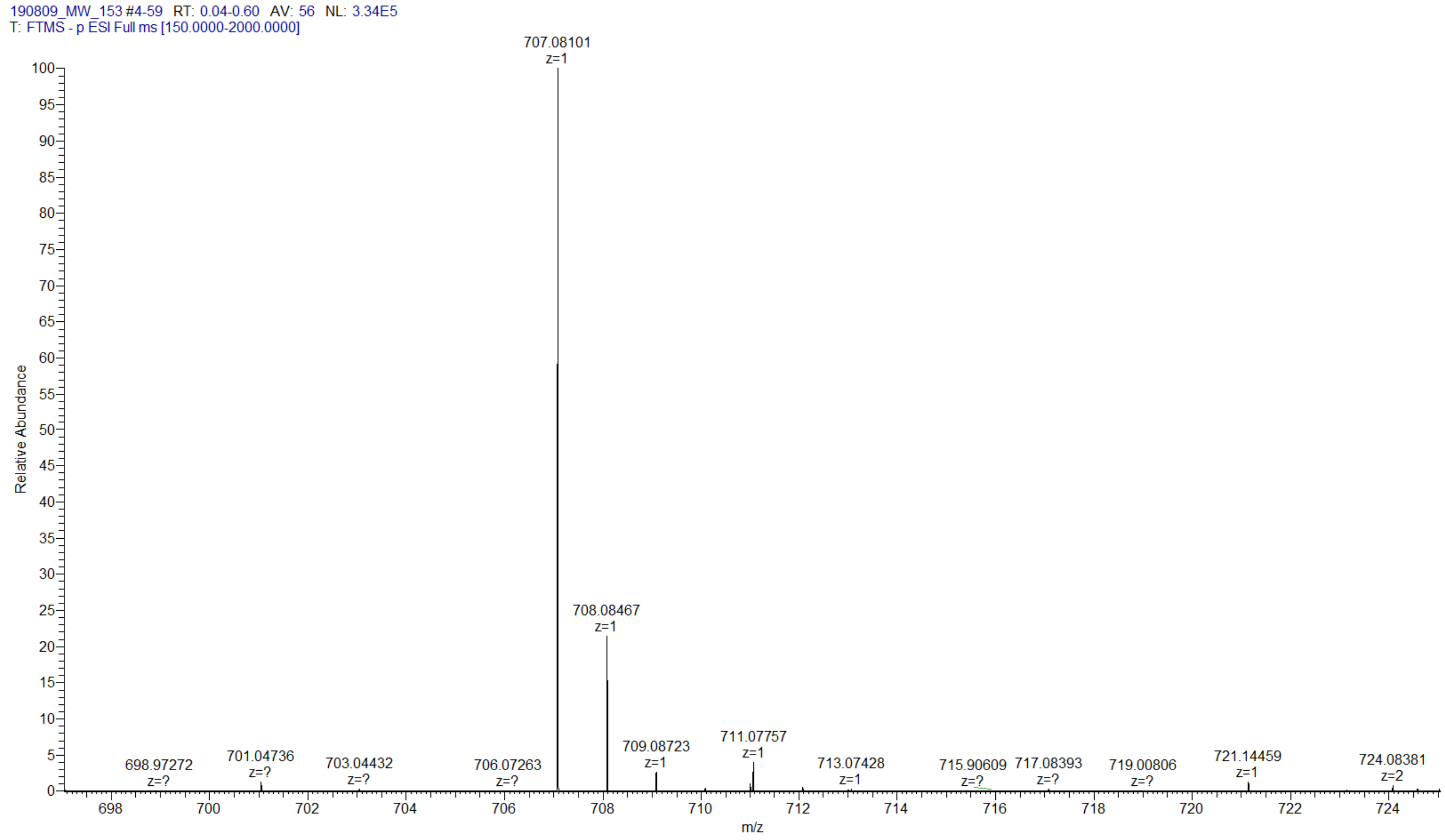

(24) p$^{5'S}$A$_m$pG	
**Chemical structure**	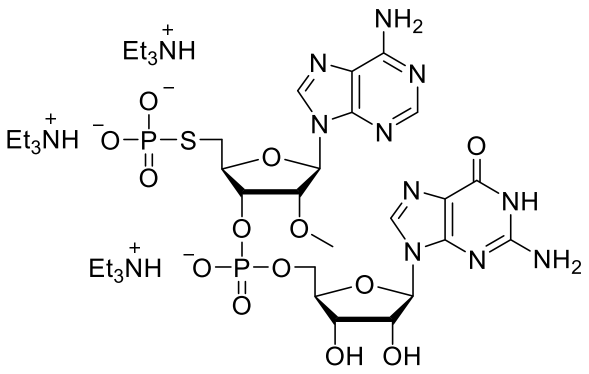
**RP HPLC**<br>Abs. @ 254 nm	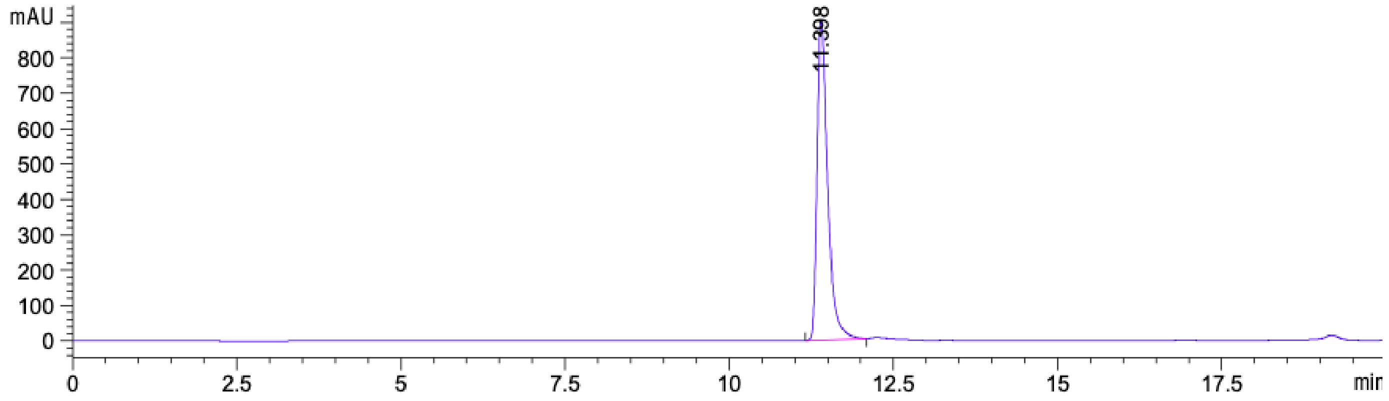

MS (-) ESI

(Calc. [M-H]⁻ $C_{21}H_{27}N_{10}O_{13}P_2S^-$ 721.09605)

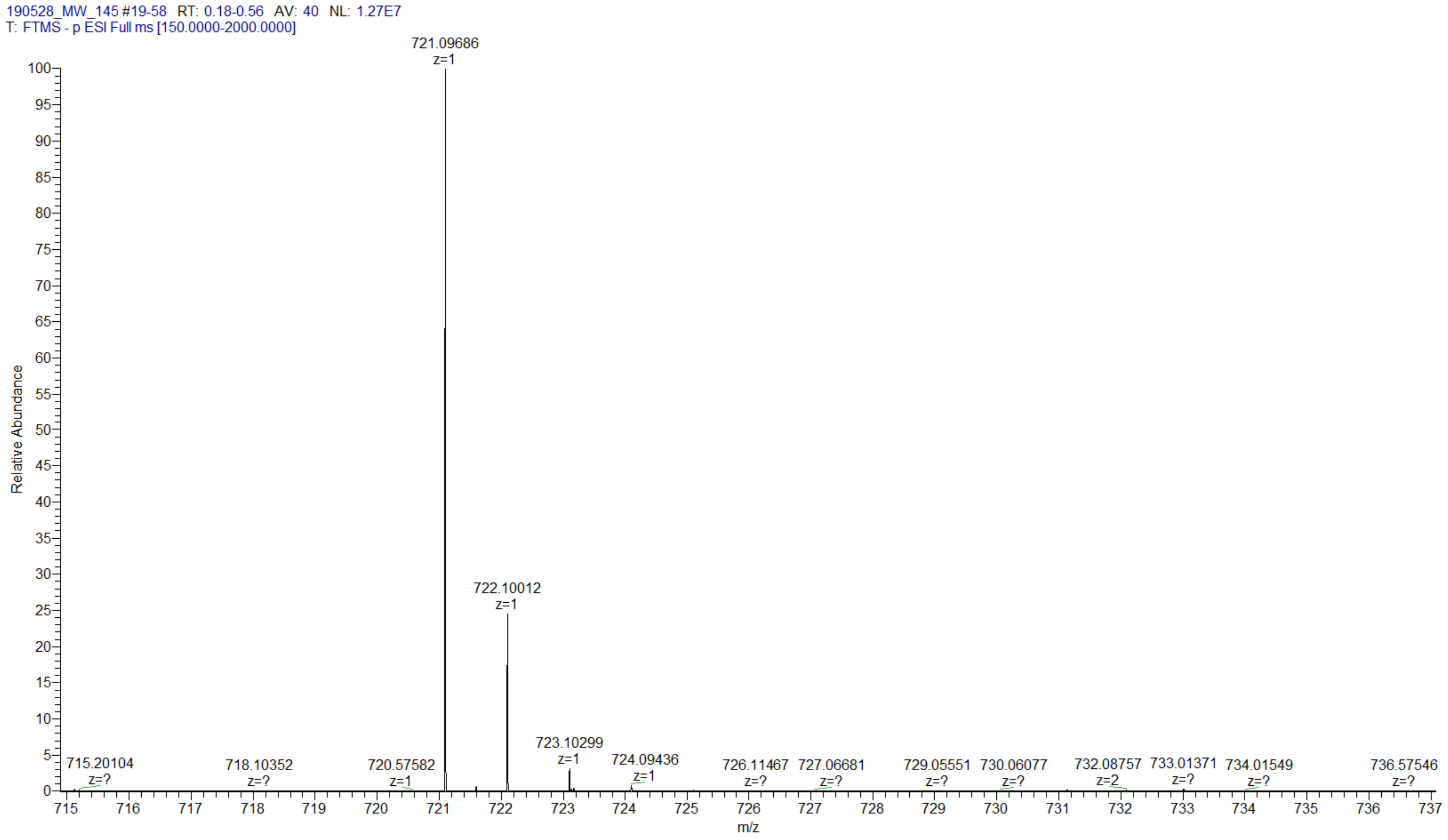

(25) pCH_2pA_mpG

Chemical structure

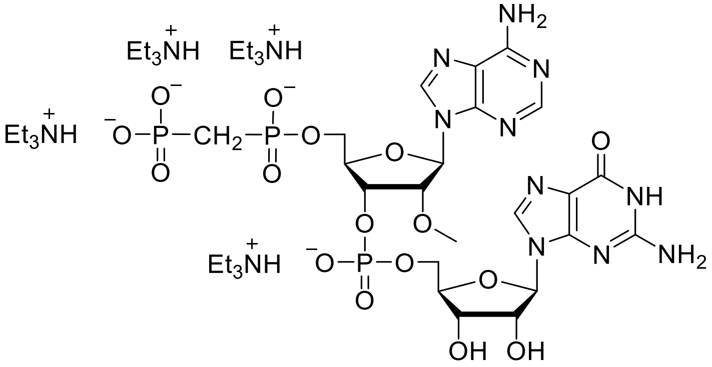

RP HPLC
Abs. @ 254 nm

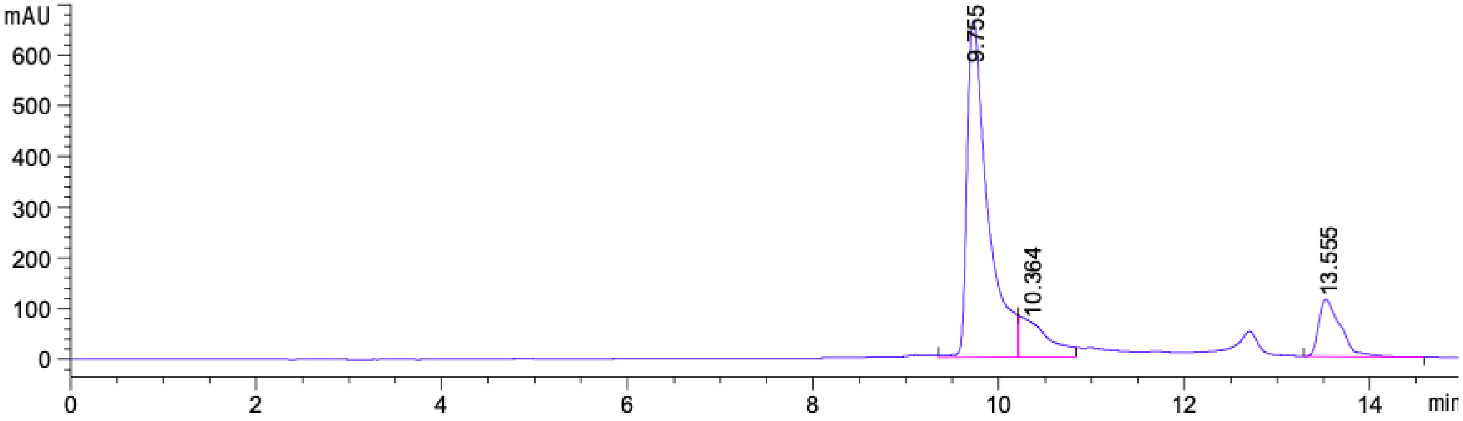

MS (-) ESI

(Calc. $[M-H]^-$ $C_{22}H_{30}N_{10}O_{16}P_3^-$ 783.10596)

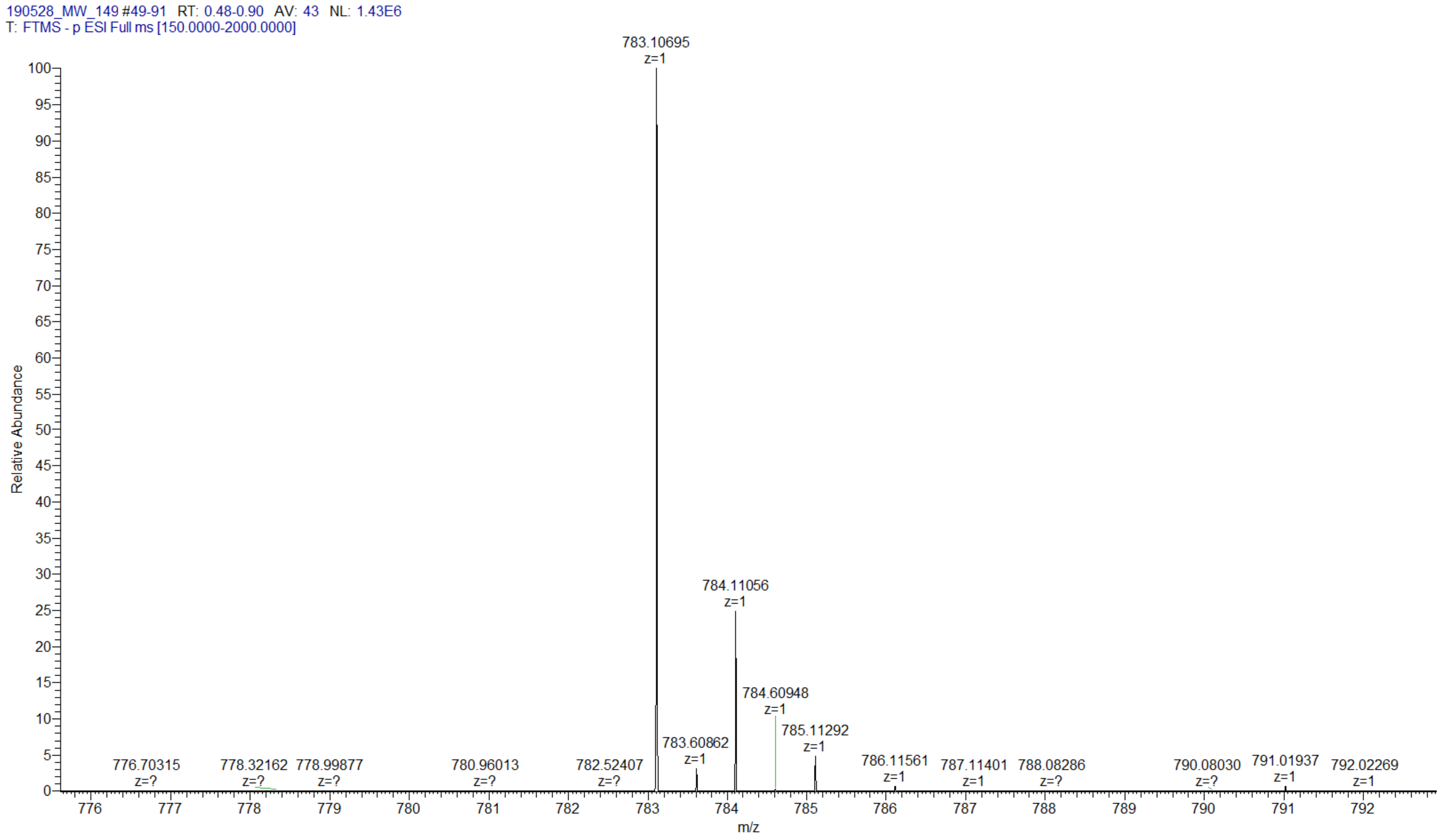

Supplement: gkae763_Supplemental_File [file gkae763_supplemental_file.pdf]
